# Synthesis of 3‑*C*‑Methyl‑d‑Mannopyranoside Derivatives Functionalized at the 3‑Position

**DOI:** 10.1021/acsomega.5c12799

**Published:** 2026-02-19

**Authors:** Shuay Abdullayev, Vikram A. Sarpe, David Crich

**Affiliations:** † Department of Pharmaceutical and Biomedical Sciences, 1355 University of Georgia, 250 West Green Street, Athens, Georgia 30602, United States; ‡ Complex Carbohydrate Research Center, 1355 University of Georgia, 315 Riverbend Road, Athens, Georgia 30602, United States; § Department of Chemistry, 1355 University of Georgia, 302 East Campus Road, Athens, Georgia 30602, United States

## Abstract

3-*C*-Methyl-d-mannopyranose is a rare branched sugar whose derivatives also serve as precursors to the more common d-evalose (6-deoxy-3-*C*-methyl-d-mannose) and, after esterification at O3, as probes for the study of the mechanism of the stereodirecting effect of esters at the 3-position in mannopyranosylation reactions. We describe alternative syntheses of 4,6-*O*-benzylidene-protected 3-*C*-methylmannopyranosyl donors and their 3-*O*-benzoates and an interesting, reversible migration of a 2-*O*-silyl derivative on the attempted esterification at O3. We also describe the reversal of stereoselectivity on application of the Corey–Chaykovsky reaction to a 3-keto sugar based on a 4,6-*O*-benzylidene-protected α-mannopyranoside: the trimethylsulfonium iodide-derived ylid favored axial attack leading to the desired mannoisomer, whereas the more bulky trimethylsulfoxonium iodide-derived ylid gave the altro isomer of the resulting spiroepoxide as a consequence of preferential equatorial attack.

## Graphical abstract

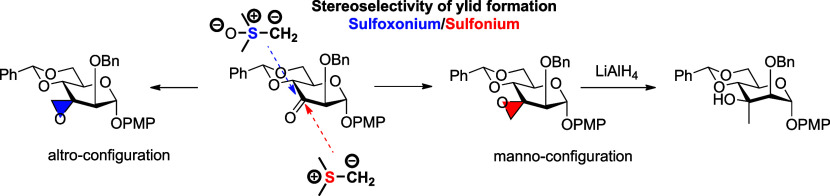

## Introduction

3-*C*-Methyl-d-mannose **2** is a rare branched sugar constituent of the trisaccharide repeating unit from strains D1, D3, and D6 of the Danish *Helicobacter pylori* atypical O-antigen polysaccharide.^1^ Its 6-deoxy derivative 3-*C*-methyl-d-rhamnose **1**, known as d-evalose, has been identified as a component of some members of the orthosomycin^2^ family of bacterial antibiotics: everninomicin B,^3^ flambamycin,^4^ and everninomicin 13–384–1.^5^ As such, 3-*C*-methylation of mannose has attracted the attention of the synthetic community (Figure 1), beginning with Yoshimura and co-workers, who obtained methyl 4,6-*O*-benzylidene-2-*O*-methyl-3-*C*-methyl-α-d-mannopyranoside by epoxidation of methyl 4,6-*O*-benzylidene-2-*O*-methyl-3-*C*-methylene-α-d-*arabino*-hexopyranoside **3** with *m*CPBA, followed by regioselective ring opening with lithium aluminum hydride (LAH).^6^ Subsequently, Žagar and Scharf converted methyl 4,6-*O*-benzylidene-2-*O*-benzyl-3-*C*-methylene-α-d-*arabino*-hexopyranoside **4** to the corresponding 3-*C*-methyl derivative of mannopyranose through dihydroxylation with osmium tetroxide, followed by removal of the primary alcohol by regioselective tosylation and LAH reduction.^7^ In their synthesis of everninomicin 13,384–1, Nicolaou et al. treated the β-glycoside **5** of 4,6-*O*-benzylidene-2-*O*-*tert*-butyldimethylsilyl-d-*arabino*-hexopyranosid-3-ulose with methyllithium in diethyl ether and obtained exclusively the manno-configuration of the resulting alcohol.^8^ Kim and co-workers used Corey-Chaykovsky epoxidation^9^ of ketosugar **6,** followed by regioselective ring opening to afford the desired 3-*C*-methyl-d-mannopyranoside; however, the reaction failed when applied to the corresponding 2′-(benzyloxycarbonyl)­benzyl (BCB) glycoside.^10^ Hydrogen bond-directed epoxidation of 3-*C*-methylene-d-mannopyranosides **7** and **8,** followed by regioselective reductive ring-opening with Bu_3_SnH/NaI/AIBN or Et_3_N·BH_3_/BF_3_·OEt_2_, provided the desired 3-*C*-methyl mannose derivatives.^10,11^ More recently, Minnaard and co-workers reported that diazomethane-mediated epoxidation of 3-ketomannosane **9**, followed by hydrogenolytic epoxide opening and eventual methanolysis, afforded methyl 3-*C*-methyl-α-d-mannopyranoside,^12^ whereas application of the same sequence to methyl 4,6-*O*-benzylidene-2-*O*-methyl-α-d-*arabino*-hexopyranosid-3-ulose gave exclusively the altro-configuration.^13^

**Figure 1:**
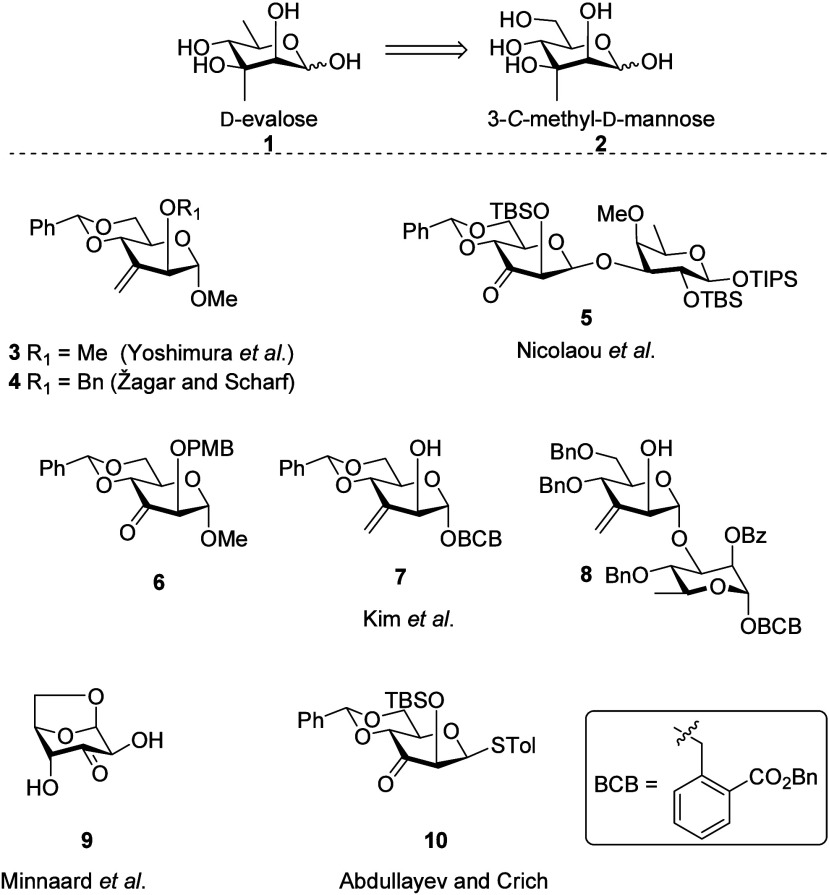
Precursors used for the synthesis of 3-*C*-methyl-d-mannosides.

In our laboratory, driven by the need for *C*-methylated sugars for use as probes in mechanistic studies of glycosylation reactions,^14,15^ we required donors of 3-*O*-benzyl-4,6-*O*-benzylidene-3-*C*-methyl-d-mannopyranosyl units protected at the 2-position as either benzyl or silyl ethers suitable for activation at low temperatures in variable temperature NMR experiments. Thus, building on the efforts of Nicolaou and co-workers with **5** ^8^ we prepared the phenyl β-d-*arabino*-hexothiopyranosid-3-ulose **10** and treated it with methyllithium in THF at −78 °C, resulting in the exclusive formation of the desired manno-configured tertiary alcohol.^15^ Although this reaction was suitable for our purposes, the extra steps required for the preparation of β-thioglycoside **10** compared to the more facile α-isomer prompted further exploration of 3-*C*-methylmannose derivatives, on which we report here.

## Results and Discussion

Our initial attempts at preparation of derivatives of **2** began with *p*-methoxyphenyl α-d-mannopyranoside **11** ^16^ converting it to the 3-*O*-*p*-methoxybenzyl derivative **12** in 78% yield by treatment with dibutyltin oxide^17^ and then *p*-methoxybenzyl chloride, followed by installation of the benzylidene acetal to give **13** in 82% yield under standard conditions (Scheme 1). Silylation with TBSOTf in lutidine then afforded 95% of **14**, from which the PMB group was removed with DDQ to give **15** in 84% yield. Dess-Martin oxidation of **15** afforded the ketone **16** in 91% yield, whose treatment with methyllithium in THF or methylmagnesium chloride in THF gave exclusively the undesired 3-*C*-methyl altro-alcohol **17,** underlining the importance of anomeric configuration in the chemistry of such ulosides and consistent with the work of Sato and Yoshimura.^13^ Drawing inspiration from Kim and co-workers,^10^ ketone **16** was subjected to Tebbe olefination,^18^ affording alkene **18** in 80% yield, from which the silyl group was removed under standard conditions to give **19** in 97% yield. Hydroxy-directed epoxidation with *m*CPBA then gave the manno-configured epoxide **20** in 60% yield, which could be reduced with LAH to the 3-*C*-methyl manno-diol **21** in 87% yield.

**Scheme 1:**
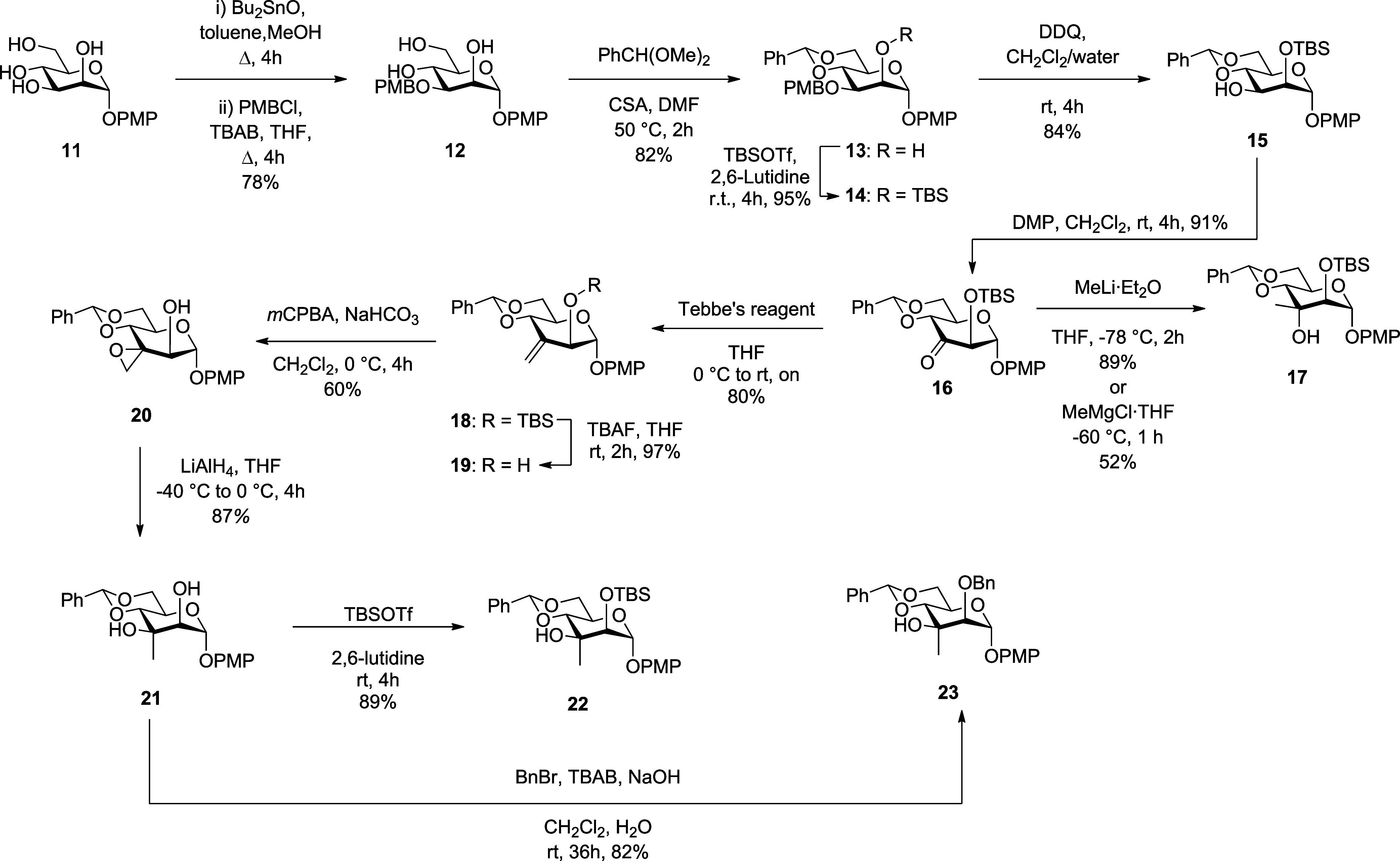
Synthesis of the α-3-*C*-Methyl Mannopyranosyl Diol **21** by H-Bond Directed Epoxidation and Subsequent Derivatization

Alcohol **21** was then converted to the 2-*O*-silyl ether **22** in 89% yield with TBDMSOTf in lutidine, while benzylation of **21** under phase transfer conditions^19^ afforded the 2-*O*-benzyl ether **23** in 82% yield.

Looking for a shorter route to a 2-*O*-benzyl derivative, DMP oxidation of **24** ^20^ gave ketone **25** from which alkene **26** was obtained by Wittig olefination in 72% yield over two steps (Scheme 2). Unfortunately, all attempts at epoxidation of **26** with *m*CPBA, Oxone, magnesium monoperoxyphthalate, hydrogen peroxide/ammonium heptamolybdate, and UHP/disodium phosphate to give the anticipated spirocyclic epoxide **27** or its epimer **28** failed as a consequence of which we turned to the Corey-Chaykovsky reaction. To this end, treatment of crude **25** with trimethylsulfonium iodide and butyllithium afforded the desired epoxide **27** in 42% yield over two steps, while the use of trimethylsulfoxonium iodide with dimsyl sodium as base for the conversion gave the altro-isomer **28** in 43% yield. This change in selectivity on epoxide formation from ketone **25** with axial attack when using trimethylsulfonium iodide and equatorial attack with the trimethylsulfoxonium iodide-derived ylid is consistent with the precedent in cyclohexanones.^9^ Possibly, with the more reactive and less hindered ylid derived from trimethylsulfonium iodide, pseudoaxial attack is preferred for stereoelectronic reasons, while the more bulky trimethylsulfoxonium iodide-derived ylid is forced to follow the less sterically demanding pseudoequatorial attack. Whatever the reason, reduction of **27** with LAH yielded diol **23** in 94% yield.

**Scheme 2:**
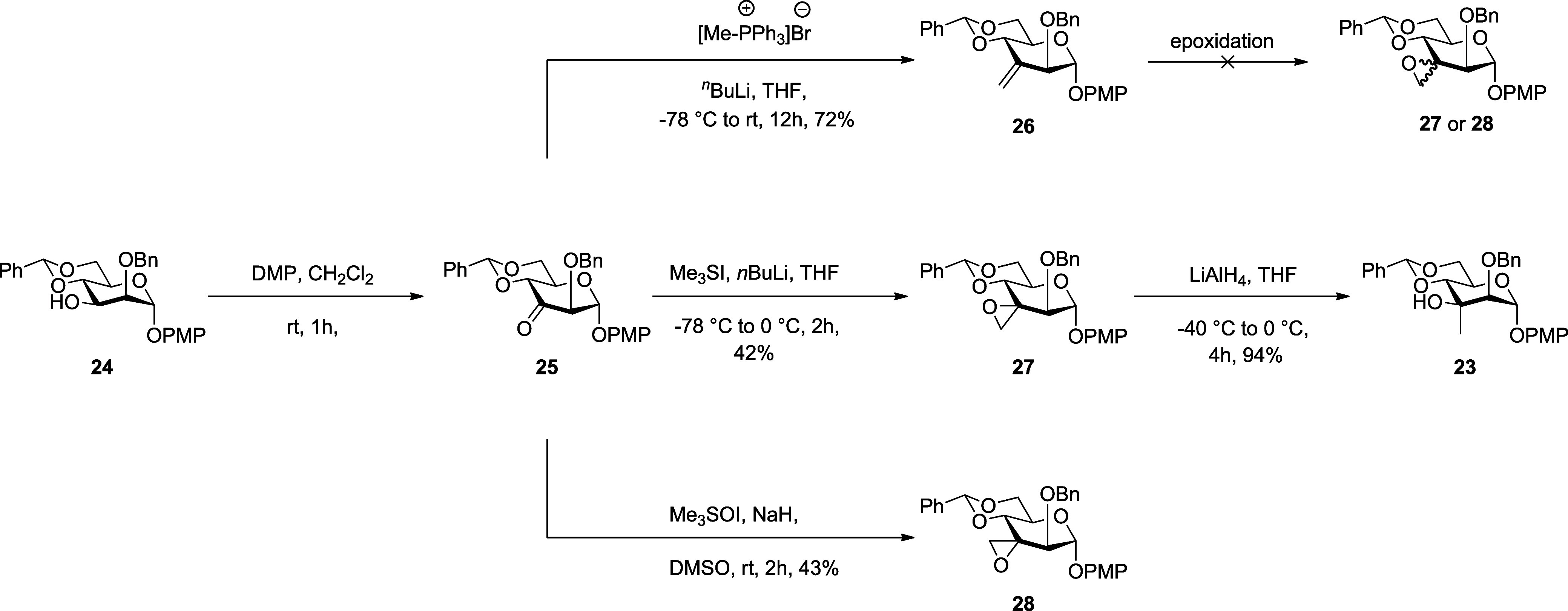
Synthesis of the Tertiary Alcohol **23** by the Corey–Chaykovsky Reaction

With alcohols **22** and **23** in hand, we turned to the installation of a tertiary benzoate ester at the 3-position and the conversion of the PMP glycoside to a thioglycoside suitable for our studies of the mechanism. Attempted benzoylation of **22** with benzoic acid and carbonyl diimidazole (CDI) in the presence of 1,8-diazabicyclo[5.4.0]­undec-7-ene (DBU), conditions previously employed successfully to install a tertiary benzoate on related tertiary alcohols,^14,15^ resulted in silyl migration and benzoylation at the less hindered secondary position to give **29** in 77% yield (Scheme 3). The location of the benzoate group in **29** was apparent from the downfield shift of H2 in the ^1^H NMR spectrum (δ 5.41) and from the strong correlation of H2 with the carbonyl carbon in the HMBC spectrum (see Supporting Information). Treatment of **29** with sodium methoxide in methanol removed the benzoate ester and provoked the reverse migration of the silyl ether, such that **22** was returned in 93% yield.

**Scheme 3:**
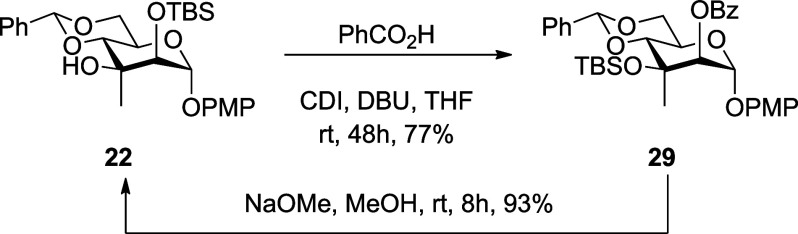
Silyl Migration on Benzoylation and Remigration on Subsequent Saponification

Benzoylation of **23** was achieved smoothly with α-^13^C-benzoic acid and CDI in the presence of DBU and gave the desired tertiary benzoate **30** in 91% yield. Unfortunately, removal of the *p*-methoxyphenyl group from **30** with ceric ammonium nitrate resulted in decomposition, presumably initiated by migration of the tertiary ester to the anomeric position. Fortunately, reversing the reaction sequence and removing the PMP group from **23** with CAN, followed by immediate acetylation with acetic anhydride in pyridine overnight at room temperature, gave a 59% yield of glycosyl acetate **32** as a mixture of anomers, which could be converted with *p*-toluenethiol in the presence of boron trifluoride etherate to thioglycoside **33** in 61% yield. Benzoylation of **33** under the standard conditions with ^13^C-enriched benzoic acid enabled conversion to **34,** presenting a viable alternative to our previous synthesis.^15^ As described previously, the chemical shift of H2 in **34** is shifted unusually downfield (δ 4.95), which we attribute to CH–O hydrogen bonding with the ester carbonyl group,^15^ but this shift is considerably smaller than that noted above for the 2-O-benozyl derivative **23** (δ 5.41), further supporting the location of the benzoate ester in that compound (Scheme 4).

**Scheme 4:**
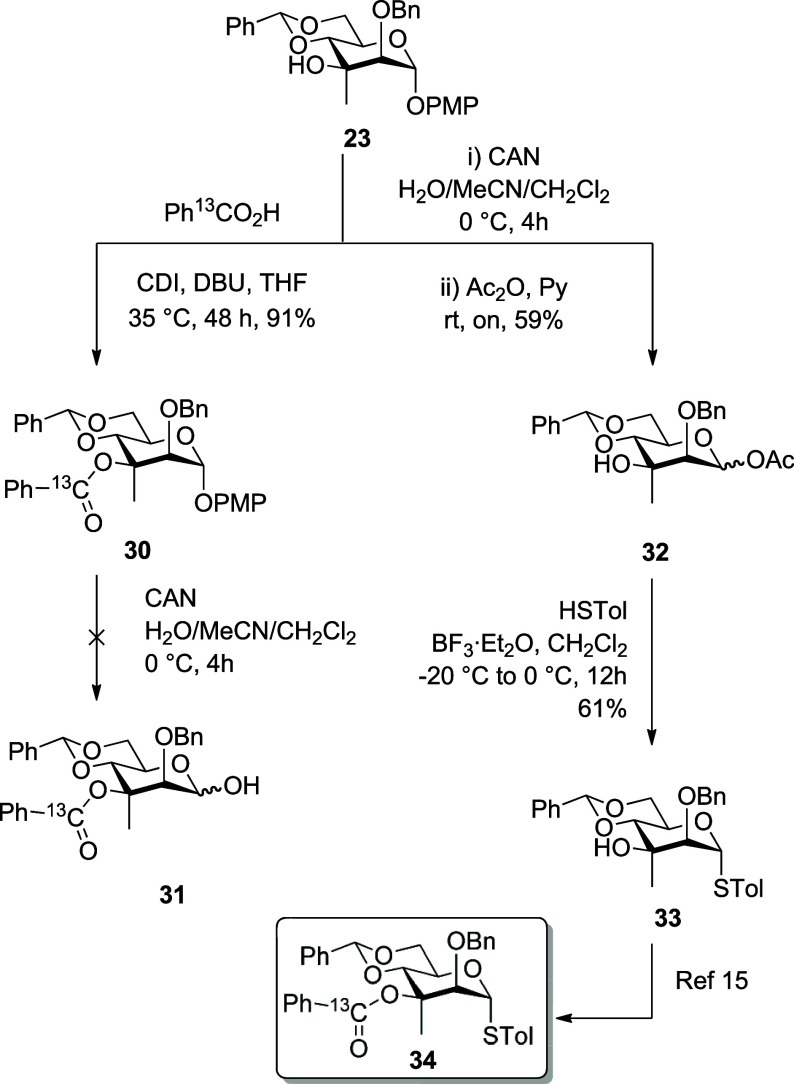
Installation of the Tertiary Benzoate and Conversion to the Glycosyl Sulfide **34**

### Assignment of Configuration and Conformation

The configuration at C3 of the altropyranoside **17** was assigned on the basis of NOE correlations between the methyl group and H’s 2 and 4, whereas those of the isomeric mannopyranoside **22**, and its 2-*O*-benzyl analog **23** follow from NOE correlations between the methyl group and H’s 2 and 5 and the typical ^3^ *J*_H1,H2_, ^3^ *J*_H4,H5_ coupling constants of 1.3 and 9.7 Hz between H’s 1 and 2 and H’s 4 and 5 in all of these compounds are typical for a ^4^C_1_-α-mannopyranosides (Figure 2). Evidently, the 1,3-diaxial interactions present in all of these compounds, while clearly inducing strain are not sufficient to cause significant distortions from the standard ^4^C_1_ chair conformation. The installation of a spiro-epoxide, as in **27** and **28** and related compounds, on the other hand, results in a distortion in the pyranose ring due to the widening of the C2–C3–C4 internal bond angle. Additionally, while the H_a_ proton of the epoxide moiety in both **27** and **28** displays NOE correlations with H’s 4 and 5 and the H_b_ epoxide proton of both compounds with H2, a unique NOE correlation between H_b_ and CH_2_ of the 2-*O*-benzyl group in isomer **28** confirmed its configuration. Ultimately, the configurational assignment of isomeric spirocyclic epoxides **27** and **28** was confirmed by the conversion of **27** to tertiary alcohol **23**.

**Figure 2:**
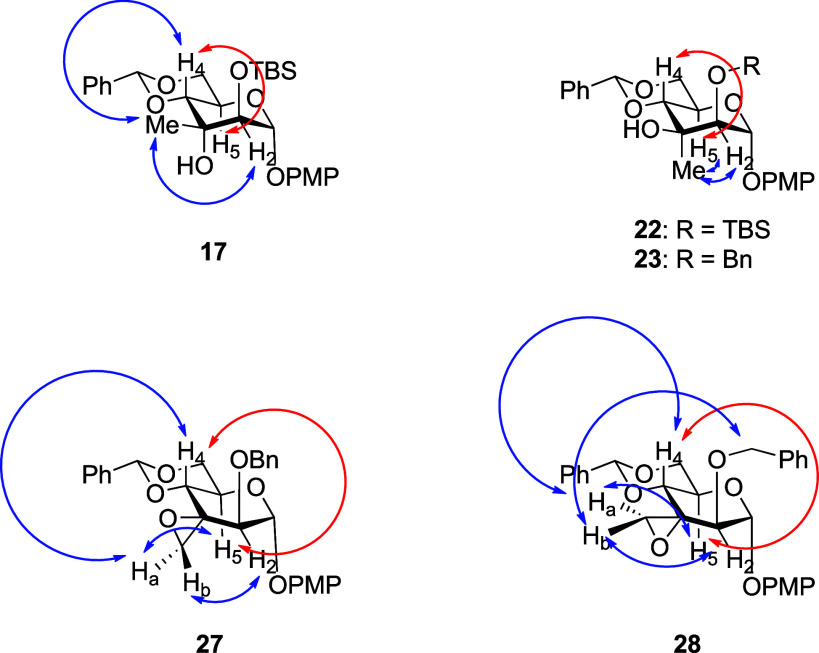
Diagnostic COSY (red) and NOE (blue) correlations for the assignment of configuration at C3.

## Conclusions

An improved route to the 4,6-*O*-benzylidene-protected-3-*C*-mannopyranosyl donors **33** and **34** is described, which should facilitate their use as mechanistic probes for glycosylation reaction mechanisms. An interesting reversal of stereoselectivity was observed on application of the Corey–Chaykovsky reaction to a 3-keto sugar derived from a 4,6-*O*-benzylidene protected α-mannopyranoside, wherein use of trimethylsulfonium iodide as reagent afforded the manno-configured spirocyclic epoxide, while use of the more highly oxidized trimethylsulfoxonium iodide gave the altro-isomer, resulting from the preferential equatorial attack of the ylid on the ketone.

## Experimental Section

### Materials and Methods

All reactions in organic media were performed in standard oven-dried glassware under an argon atmosphere. All reagents were used as supplied without prior purification, unless otherwise stated. Solvents used for column chromatography were analytical grade and purchased from commercial suppliers. For all heated reactions, an appropriately sized, thermostatically controlled heating block was used as a heat source. Reactions were monitored by analytical thin-layer chromatography (TLC) using 250 μm glass backed silica and compounds were visualized with a 254 nm UV lamp or ceric ammonium molybdate solution (100 mL H_2_SO_4_, 900 mL H_2_O, 25 g (NH_4_)_6_Mo_7_O_24_H_2_O, 10 g Ce­(SO_4_)_2_) with spots developed by gentle warming with a heat-gun. Purifications were performed by flash column chromatography using a COMBIFLASH NEXTGEN system with the indicated eluent. Specific rotations [α]_D_ were measured at 589 nm and 22 ± 1 °C in CHCl_3_ or CH_3_OH as stated, with a path length of 10 cm, with an automatic polarimeter. ^1^H NMR and ^13^C NMR spectra were recorded at 600 and 151 MHz, respectively. Proton and carbon chemical shifts (δ) are reported in ppm relative to the chemical shift of residual CD_2_Cl_2_ (in ^1^H 5.32 ppm, in ^13^C 53.84 ppm), CDCl_3_ (in ^1^H 7.26 ppm, in ^13^C 77.16 ppm), and CD_3_OD (in ^1^H, 3.31 ppm and in ^13^C, 49.0 ppm). Selective 1D Nuclear Overhauser Effect Spectroscopy (NOESY) and 2D homonuclear COrrelation Spectroscopy ^1^H–^1^H (COSY), ^1^H–^13^C Heteronuclear Single Quantum Coherence (HSQC), and Heteronuclear Multiple Bond Correlation (HMBC) experiments were used to confirm NMR peak assignments. Coupling constants (*J*) are reported in Hertz (Hz), and the following abbreviations are used for peak multiplicities: singlet (s), broad singlet (br s), doublet (d), doublet of doublets (dd), doublet of doublets of doublets (ddd), triplet (t), doublet of triplets (dt), triplet of doublets (td), triplet of triplets (tt), and multiplet (m). High-resolution electrospray ionization (ESI) mass spectrometry data were recorded with a Thermo Scientific Orbitrap mass analyzer.

### Experimental Procedures for the Preparation of Compounds

#### *p*-Methoxyphenyl 3-*O*-*p*-Methoxybenzyl-α-d-mannopyranoside (**12**)

Using a Dean–Stark apparatus, compound **11** ^16^ (10.1 g, 35.28 mmol, 1 equiv) and dibutyltin oxide (9.22 g, 37.04 mmol, 1.05 equiv) in anhydrous methanol/toluene mixture (v/v–1/5; 120 mL) was refluxed for 4 h. The clear solution was cooled to room temperature and concentrated under reduced pressure. The residue was dissolved in dry THF (150 mL), and tetrabutylammonium bromide (TBAB, 2.9 g, 8.99 mmol, 0.25 equiv) was added, followed by 4-methoxybenzyl chloride (PMBCl, 9.6 mL, 70.49 mmol, 2 equiv). The mixture was refluxed for 4 h, cooled to room temperature, and concentrated in vacuo and purified by flash column chromatography by gradient elution with EtOAc:MeOH (1% MeOH in EtOAc to 10% MeOH/EtOAc) to afford title compound **12** (11.16 g, 27.46 mmol, 78%) as a white solid. [α]_D_^22^ +66.9 (C 1.0, CH_3_OH). ^1^H NMR (600 MHz, CD_3_OD) δ 7.33 (d, *J* = 8.4 Hz, 2H, H_o_-PMB), 7.01 (d, *J* = 9.0 Hz, 2H, H_o_-PMP), 6.91 (d, *J* = 8.4 Hz, 2H, H_m_-PMB), 6.83 (d, *J* = 9.0 Hz, 2H, H_m_-PMP), 5.34 (s, 1H, H-1), 4.71 (d, *J* = 11.3 Hz, 1H, OCH_2_Ar), 4.65 (d, *J* = 11.3 Hz, 1H, OCH_2_Ar), 4.12 (t, *J* = 2.6 Hz, 1H, H-2), 3.86 (t, *J* = 9.6 Hz, 1H, H-4), 3.80–3.76 (m, 5H, H-3, H-6a, OCH_3_–PMB), 3.74–3.71 (m, 4H, H-6b, OCH_3_–PMP), 3.68–3.65 (m, 1H, H-5). ^13^C­{H} NMR (151 MHz, CD_3_OD) δ 160.8 (C_p_-PMB), 156.6 (C_p_-PMP), 151.9 (C_i_-PMP), 131.9 (C_i_-PMB), 130.8 (C_o_-PMB), 119.2 (C_o_-PMP), 115.6 (C_m_-PMP), 114.7 (C_m_-PMB), 101.1 (C-1), 79.8 (C-3), 75.3 (C-5), 72.5 (OCH_2_Ar), 69.1 (C-2), 67.4 (C-4), 62.5 (C-6), 56.0 (OCH_3_–PMP), 55.7 (OCH_3_–PMB). ESI-HRMS: *m*/*z* calcd for C_21_H_26_O_8_ [M + Na]^+^ 429.1520, found 429.1508.

#### *p*-Methoxyphenyl 4,6-*O*-Benzylidene-3-*O*-*p*-methoxybenzyl-α-d-mannopyranoside (**13**)

A round-bottom flask containing a mixture of compound **12** (11.6 g, 27.46 mmol, 1 equiv), benzaldehyde dimethyl acetal (5.7 mL, 37.98 mmol, 1.4 equiv), and (1S)-(+)-10-camphorsulfonic acid (CSA, 1.91 g, 8.22 mmol, 0.3 equiv) in dry DMF (110 mL) was hooked up to rotatory evaporator and stirred under 120 mbar at 50 °C for 2 h and neutralized with triethylamine (10 mL), and concentrated in vacuo. The residue was purified by flash column chromatography by gradient elution of hexane:EtOAc (5% EtOAc in hexane to 30% EtOAc/hexane) to afford the title compound **13** (11.13 g, 22.51 mmol, 82%) as a white foam. [α]_D_^22^ +105.1 (C 1.0, CHCl_3_). ^1^H NMR (600 MHz, CDCl_3_) δ 7.51 (dd, *J* = 8.0, 1.8 Hz, 2H, H_o_-Ph), 7.41–7.35 (m, 3H, H_m_-, H_p_-Ph), 7.34–7.31 (m, 2H, H_o_-PMB), 6.99–6.96 (m, 2H, H_o_-PMP), 6.90–6.88 (m, 2H, H_m_-PMB), 6.85–6.82 (m, 2H, H_m_-PMP), 5.63 (s, 1H, benzylidene-CH), 5.49 (d, *J* = 1.6 Hz, 1H, H-1), 4.86 (d, *J* = 11.3 Hz, 1H, OCH_2_Ar), 4.71 (d, *J* = 11.3 Hz, 1H, OCH_2_Ar), 4.22–4.19 (m, 2H, H-6a, H-2), 4.18–4.15 (m, 1H, H-3), 4.10 (dd, *J* = 9.6, 3.4 Hz, 1H, H-4), 3.99 (td, *J* = 9.8, 4.8 Hz, 1H, H-5), 3.84 (t, *J* = 10.3 Hz, 1H, H-6b), 3.80 (s, 3H, OCH_3_-PMB), 3.78 (s, 3H, OCH_3_-PMP), 2.88 (s, 1H, OH). ^13^C­{H} NMR (151 MHz, CDCl_3_) δ 159.6 (C_p_-PMB), 155.2 (C_p_-PMP), 149.9 (C_i_-PMP), 137.6 (C_i_-Ph), 130.1 (C_i_-PMB), 129.7 (C_o_-PMB), 129.1 (C_p_-Ph), 128.3 (C_m_-Ph), 126.2 (C_o_-Ph), 117.9 (C_o_-PMP), 114.8 (C_m_-PMP), 114.0 (C_m_-PMB), 101.7 (benzylidene-CH), 98.9 (C-1), 78.9 (C-3), 75.3 (C-4), 73.1 (OCH_2_Ar), 70.1 (C-2), 68.8 (C-6), 64.0 (C-5), 55.8 (OCH_3_–PMP), 55.4 (OCH_3_–PMB). ESI-HRMS: *m*/*z* calcd for C_28_H_30_O_8_ [M + Na]^+^ 517.1833, found 517.1830.

#### *p*-Methoxyphenyl 4,6-*O*-Benzylidene-3-*O*-*p*-methoxybenzyl-2-*O*-*tert*-butyldimethylsilyl-α-d-mannopyranoside (**14**)

To a solution of compound **13** (7.3 g, 14.76 mmol, 1 equiv) in dry 2,6-lutidine (60 mL) was added *tert*-butyldimethylsilyl trifluoromethanesulfonate (TBSOTf, 5.1 mL, 22.21 mmol, 1.5 equiv), and the reaction mixture was allowed to stir vigorously at room temperature for 4 h. The reaction mixture was quenched with MeOH (10 mL) and concentrated in vacuo. The residue was purified by flash column chromatography by gradient elution of hexane:EtOAc (1% EtOAc in hexane to 15% EtOAc/hexane) to afford compound **14** (8.58 g, 14.09 mmol, 95%) as a white foam. [α]_D_^22^ +35.5 (C 1.0, CHCl_3_). ^1^H NMR (600 MHz, CDCl_3_) δ 7.50–7.49 (m, 2H, H_o_-Ph), 7.38–7.34 (m, 3H, H_p_-, H_m_-Ph), 7.32–7.30 (m, 2H, H_o_-PMB), 6.97–6.94 (m, 2H, H_o_-PMP), 6.87–6.84 (m, 2H, H_m_-PMB), 6.84–6.82 (m, 2H, H_m_-PMP), 5.63 (s, 1H, benzylidene-CH), 5.25 (d, *J* = 1.8 Hz, 1H, H-1), 4.79 (d, *J* = 11.5 Hz, 1H, OCH_2_Ar), 4.69 (d, *J* = 11.5 Hz, 1H, OCH_2_Ar), 4.21 (dd, *J* = 3.1, 1.8 Hz, 1H, H-2), 4.19–4.16 (m, 2H, H-6a, H-3), 4.03 (dd, *J* = 9.8, 3.0 Hz, 1H, H-4), 3.92 (td, *J* = 9.8, 4.7 Hz, 1H, H-5), 3.83–3.79 (m, 4H, H-6b, OCH_3_-PMB), 3.78 (s, 3H, OCH_3_-PMP), 0.92 (s, 9H, Si–C­(CH_3_)_3_), 0.11 (s, 3H, Si-CH_3_), 0.08 (s, 3H, Si-CH_3_). ^13^C­{H} NMR (151 MHz, CDCl_3_) δ 159.2 (C_p_-PMB), 155.2 (C_p_-PMP), 150.1 (C_i_-PMP), 137.9 (C_i_-Ph), 130.9 (C_i_-PMB), 129.6 (C_o_-PMB), 128.9 (C_p_-Ph), 128.3 (C_m_-Ph), 126.3 (C_o_-Ph), 117.9 (C_o_-PMP), 114.8 (C_m_-PMP), 113.7 (C_m_-PMB), 101.7 (benzylidene-CH), 100.6 (C-1), 79.3 (C-3), 75.4 (C-4), 72.9 (OCH_2_Ar), 71.5 (C-2), 69.0 (C-6), 65.1 (C-5), 55.8 (OCH_3_–PMP), 55.4 (OCH_3_–PMB), 26.0 (Si–C­(*C*H_3_)_3_), 18.4 (Si-*C*(CH_3_)_3_), −4.2 (Si-CH_3_), −4.9 (Si-CH_3_). ESI-HRMS: *m*/*z* calcd for C_34_H_44_O_8_Si [M + Na]^+^ 631.2698, found 631.2675.

#### *p*-Methoxyphenyl 4,6-*O*-Benzylidene-2-*O*-*tert*-butyldimethylsilyl-α-d-mannopyranoside (**15**)

To a solution of compound **14** (6.1 g, 10.02 mmol, 1 equiv) in DCM (50 mL) was added distilled water (DCM/water: v/v:10/1; 5 mL), followed by 2,3-dichloro-5,6-dicyano-1,4-benzoquinone (DDQ, 2.73 g, 12.03 mmol, 1.2 equiv) at room temperature. The reaction mixture was stirred for 4 h and quenched by a saturated aqueous solution of NaHCO_3_ (30 mL). The organic layer was extracted, diluted in DCM (80 mL), washed with 2 M NaOH (80 mL) and brine (80 mL), dried over anhydrous Na_2_SO_4,_ and concentrated in vacuo. The residue was purified by flash column chromatography by gradient elution with hexane:EtOAc (1% EtOAc in hexane to 30% EtOAc/hexane) to afford compound **15** (4.1 g, 8.39 mmol, 84%) as a white foam. [α]_D_^22^ +56.3 (C 1.0, CHCl_3_). ^1^H NMR (600 MHz, CDCl_3_) δ 7.52–7.50 (m, 2H, H_o_-Ph), 7.38–7.33 (m, 3H, H_p_-, H_m_-Ph), 7.00–6.97 (m, 2H, H_o_-PMP), 6.86–6.84 (m, 2H, H_m_-PMP), 5.59 (s, 1H, benzylidene-CH), 5.30 (s, 1H, H-1), 4.22–4.18 (m, 3H, H-2, H-6a, H-3), 3.95 (td, *J* = 9.7, 4.6 Hz, 1H, H-5), 3.90 (t, *J* = 9.2 Hz, 1H, H-4), 3.80–3.77 (m, 4H, H-6b, OCH_3_-PMP), 2.19 (br. s, 1H, OH), 0.97 (s, 9H, Si–C­(CH_3_)_3_), 0.18 (s, 3H, Si-CH_3_), 0.15 (s, 3H, Si-CH_3_). ^13^C­{H} NMR (151 MHz, CDCl_3_) δ 155.3 (C_p_-PMP), 150.1 (C_i_-PMP), 137.5 (C_i_-Ph), 129.2 (C_p_-Ph), 128.4 (C_m_-Ph), 126.5 (C_o_-Ph), 117.9 (C_o_-PMP), 114.8 (C_m_-PMP), 102.4 (benzylidene-CH), 100.0 (C-1), 79.5 (C-4), 72.5 (C-2), 68.9 (C-6), 68.8 (C-3), 64.4 (C-5), 55.8 (OCH_3_–PMP), 25.9 (Si–C­(*C*H_3_)_3_), 18.3 (Si-*C*(CH_3_)_3_), −4.5 (Si-CH_3_), −4.6 (Si-CH_3_). ESI-HRMS: *m*/*z* calcd for C_26_H_36_O_7_Si [M + Na]^+^ 511.2122, found 511.2104.

#### *p*-Methoxyphenyl 4,6-*O*-Benzylidene-2-*O*-*tert*-butyldimethylsilyl-α-d-*arabino*-hexopyranosid-3-ulose (**16**)

To a solution of compound **15** (4.1 g, 8.39 mmol, 1 equiv) in DCM (60 mL) was added Dess–Martin periodinane (DMP, 5.4 g, 12.73 mmol, 1.5 equiv) at room temperature. The reaction mixture was stirred for 4 h and quenched by adding a saturated aqueous solution of Na_2_S_2_O_3_ (60 mL) and stirred for an additional 15 min. The organic layer was extracted with DCM (200 mL), washed with saturated aqueous NaHCO_3_ (150 mL) and brine (150 mL), dried over anhydrous Na_2_SO_4,_ and concentrated in vacuo. The residue was purified by flash column chromatography by gradient elution with hexane: EtOAc (1% EtOAc in hexane to 15% EtOAc/hexane) to afford compound **16** (3.72 g, 7.64 mmol, 91%) as a white foam. [α]_D_^22^ +87.1 (C 1.0, CHCl_3_). ^1^H NMR (600 MHz, CDCl_3_) δ 7.55–7.53 (m, 2H, H_o_-Ph), 7.39–7.36 (m, 3H, H_p_-, H_m_-Ph), 7.02–6.99 (m, 2H, H_o_-PMP), 6.87–6.85 (m, 2H, H_m_-PMP), 5.65 (s, 1H, benzylidene-CH), 5.55 (d, *J* = 1.6 Hz, 1H, H-1), 4.96 (d, *J* = 9.7 Hz, 1H, H-4), 4.32–4.24 (m, 3H, H-6a, H-2, H-5), 3.98 (t, *J* = 10.0 Hz, 1H, H-6b), 3.77 (s, 3H, OCH_3_-PMP), 0.98 (s, 9H, Si–C­(CH_3_)_3_), 0.19 (s, 3H, Si-CH_3_), 0.16 (s, 3H, Si-CH_3_). ^13^C­{H} NMR (151 MHz, CDCl_3_) δ 197.7 (C=O), 155.3 (C_p_-PMP), 149.3 (C_i_-PMP), 136.6 (C_i_-Ph), 129.3 (C_p_-Ph), 128.3 (C_m_-Ph), 126.5 (C_o_-Ph), 117.5 (C_o_-PMP), 114.8 (C_m_-PMP), 102.2 (benzylidene-CH), 102.1 (C-1), 80.4 (C-4), 72.3 (C-2), 69.3 (C-6), 66.9 (C-5), 55.6 (OCH_3_–PMP), 25.7 (Si–C­(*C*H_3_)_3_), 18.1 (Si-*C*(CH_3_)_3_), −4.8 (Si-CH_3_), −5.0 (Si-CH_3_). ESI-HRMS: *m*/*z* calcd for C_26_H_34_O_7_Si [M + Na]^+^ 509.1966, found 509.1960.

#### *p*-Methoxyphenyl 4,6-*O*-Benzylidene-2-*O*-*tert*-butyldimethylsilyl-3-*C*-methyl-α-d-altropyranoside (**17**)

##### *C*-3 Methylation with Methylithium

To a solution of compound **16** (50 mg, 0.102 mmol, 1 equiv) in anhydrous THF (5 mL) was added a 1.6 M solution of methyllithium in THF (150 μL, 0.240 mmol, 2.4 equiv) at −78 °C under an argon atmosphere. The reaction mixture was stirred for 1 h at the same temperature, quenched with saturated aqueous NH_4_Cl (1 mL), and diluted in EtOAc (20 mL). The organic layer was extracted, washed with brine (10 mL), dried over anhydrous Na_2_SO_4,_ and concentrated in vacuo. The residue was purified by flash column chromatography by gradient elution with hexane:EtOAc (1% EtOAc in hexane to 20% EtOAc/hexane) to afford compound **17** (46 mg, 0.091 mmol, 89%) as a white foam.

##### *C*-3 Methylation with Methylmagnesium Chloride

To a solution of compound **16** (15 mg, 0.031 mmol, 1 equiv) in anhydrous THF (1 mL) was added a 3.0 M solution of methylmagnesium chloride in THF (50 μL, 0.150 mmol, 4.8 equiv) at −60 °C under an argon atmosphere. The reaction mixture was stirred for 1 h at the same temperature, quenched with saturated aqueous NH_4_Cl (1 mL), and diluted in EtOAc (5 mL). The organic layer was extracted, washed with brine (3 mL), dried over anhydrous Na_2_SO_4,_ and concentrated in vacuo. The residue was purified by flash column chromatography by gradient elution with hexane:EtOAc (1% EtOAc in hexane to 20% EtOAc/hexane) to afford compound **17** (8 mg, 0.016 mmol, 52%) as a white foam. [α]_D_^22^ +47.9 (C 2.0, CHCl_3_). ^1^H NMR (600 MHz, CDCl_3_) δ 7.53–7.51 (m, 2H, H_o_-Ph), 7.38–7.33 (m, 3H, H_p_-, H_m_-Ph), 7.03–7.00 (m, 2H, H_o_-PMP), 6.87–6.84 (m, 2H, H_m_-PMP), 5.62 (s, 1H, benzylidene-CH), 5.24 (d, *J* = 1.3 Hz, 1H, H-1), 4.30–4.26 (m, 2H, H-6a, H-5), 3.86 (d, *J* = 1.3 Hz, 1H, H-2), 3.83–3.73 (m, 5H, H-4, H-6b, OCH_3_–PMP), 3.41 (br. s, 1H, OH), 1.39 (s, 3H, HO–C–C*H*_3_), 0.96 (s, 9H, Si–C­(CH_3_)_3_), 0.16 (s, 3H, Si-CH_3_), 0.13 (s, 3H, Si-CH_3_). ^13^C­{H} NMR (151 MHz, CDCl_3_) δ 155.7 (C_p_-PMP), 149.9 (C_i_-PMP), 137.7 (C_i_-Ph), 129.0 (C_p_-Ph), 128.3 (C_m_-Ph), 126.4 (C_o_-Ph), 118.6 (C_o_-PMP), 114.9 (C_m_-PMP), 102.2 (benzylidene-CH), 101.4 (C-1), 79.8 (C-4), 74.7 (C-2), 71.9 (C-3), 69.2 (C-6), 60.7 (C-5), 55.8 (OCH_3_–PMP), 25.9 (Si–C­(*C*H_3_)_3_), 22.1 (HO-C-*C*H_3_), 18.0 (Si-*C*(CH_3_)_3_), −4.5 (Si-CH_3_), −4.6 (Si-CH_3_). ESI-HRMS: *m*/*z* calcd for C_27_H_38_O_7_Si [M + Na]^+^ 525.2279, found 525.2272.

#### *p*-Methoxyphenyl 4,6-O-Benzylidene-2-O-*tert*-butyldimethylsilyl-3-deoxy-3-C-methylene-α-d-*arabino*-hexopyranoside (**18**)

To a solution of compound **16** (3.72 g, 7.64 mmol, 1 equiv) in anhydrous THF (50 mL) at 0 °C was added a 0.5 M solution of Tebbe’s reagent in THF (30 mL, 15 mmol, 2 equiv) under an argon atmosphere. The reaction mixture was stirred overnight at room temperature, then cooled to 0 °C, quenched by dropwise addition of 10% aqueous NaOH (20 mL), and filtered over a pad of Celite. The filtrate was diluted in EtOAc (100 mL), and the organic layer was washed with brine solution (100 mL), dried over anhydrous Na_2_SO_4,_ and concentrated in vacuo. The residue was purified by flash column chromatography by gradient elution with hexane:EtOAc (1% EtOAc in hexane to 10% EtOAc/hexane) to afford compound **18** (2.96 g, 6.11 mmol, 80%) as a white foam. [α]_D_^22^ +88.6 (C 1.0, CHCl_3_). ^1^H NMR (600 MHz, CDCl_3_) δ 7.55 (dd, *J* = 7.8, 1.8 Hz, 2H, H_o_-Ph), 7.41–7.36 (m, 3H, H_p_-, H_m_-Ph), 7.02–6.99 (m, 2H, H_o_-PMP), 6.86–6.83 (m, 2H, H_m_-PMP), 5.70 (s, 1H, benzylidene-CH), 5.35 (t, *J* = 1.7 Hz, 1H, C=CH_2_), 5.31 (s, 1H, H-1), 5.21 (t, *J* = 1.7 Hz, 1H, C=CH_2_) 4.51 (dt, *J* = 9.4, 2.0 Hz, 1H, H-4), 4.43 (s, 1H, H-2), 4.19 (dd, *J* = 10.0, 4.6 Hz, 1H, H-6a), 3.94 (td, *J* = 9.8, 4.6 Hz, 1H, H-5), 3.86 (t, *J* = 10.2 Hz, 1H, H-6b), 3.78 (s, 3H, OCH_3_-PMP), 0.95 (s, 9H, Si–C­(CH_3_)_3_), 0.15 (s, 3H, Si-CH_3_), 0.14 (s, 3H, Si-CH_3_). ^13^C­{H} NMR (151 MHz, CDCl_3_) δ 155.1 (C_p_-PMP), 150.2 (C_i_-PMP), 141.7 (*C*=CH_2_), 137.8 (C_i_-Ph), 129.2 (C_p_-Ph), 128.4 (C_m_-Ph), 126.4 (C_o_-Ph), 118.0 (C_o_-PMP), 114.7 (C_m_-PMP), 110.3 (C=*C*H_2_), 101.9 (benzylidene-CH), 100.5 (C-1), 76.9 (C-4), 75.3 (C-2), 69.5 (C-6), 67.2 (C-5), 55.8 (OCH_3_–PMP), 25.9 (Si–C­(*C*H_3_)_3_), 18.3 (Si-*C*(CH_3_)_3_), −4.4 (Si-CH_3_), −4.8 (Si-CH_3_). ESI-HRMS: *m*/*z* calcd for C_27_H_36_O_6_Si [M + Na]^+^ 507.2173, found 507.2165.

#### *p*-Methoxyphenyl 4,6-*O*-Benzylidene-3-deoxy-3-*C*-methylene-α-d-*arabino*-hexopyranoside (**19**)

To a solution of compound **18** (2.96 g, 6.11 mmol, 1 equiv) in anhydrous THF (50 mL) was added a 1 M solution of tetrabutylammonium fluoride (TBAF, 12 mL, 12 mmol, 2 equiv) in THF at room temperature. The reaction mixture was stirred for 2 h and then concentrated in vacuo. The residue was purified by flash column chromatography by gradient elution with hexane:EtOAc (10% EtOAc in hexane to 60% EtOAc/hexane) to afford compound **19** (2.2 g, 5.94 mmol, 97%) as a white foam. [α]_D_^22^ +136.0 (C 1.0, CHCl_3_). ^1^H NMR (600 MHz, CDCl_3_) δ 7.53 (dd, *J* = 7.6, 2.0 Hz, 2H, H_o_-Ph), 7.40–7.36 (m, 3H, H_p_-, H_m_-Ph), 7.01–6.98 (m, 2H, H_o_-PMP), 6.84–6.82 (m, 2H, H_m_-PMP), 5.67 (s, 1H, benzylidene-CH), 5.44 (s, 1H, H-1), 5.39 (t, *J* = 1.7 Hz, 1H, C=CH_2_), 5.29 (t, *J* = 1.7 Hz, 1H, C=CH_2_) 4.55 (dt, *J* = 9.6, 2.1 Hz, 1H, H-4), 4.46 (s, 1H, H-2), 4.20 (dd, *J* = 10.3, 4.8 Hz, 1H, H-6a), 3.98 (td, *J* = 9.9, 4.8 Hz, 1H, H-5), 3.86 (t, *J* = 10.3 Hz, 1H, H-6b), 3.77 (s, 3H, OCH_3_-PMP), 2.32 (s, 1H, OH). ^13^C­{H} NMR (151 MHz, CDCl_3_) δ 155.2 (C_p_-PMP), 150.0 (C_i_-PMP), 141.1 (*C*=CH_2_), 137.6 (C_i_-Ph), 129.2 (C_p_-Ph), 128.4 (C_m_-Ph), 126.4 (C_o_-Ph), 118.0 (C_o_-PMP), 114.8 (C_m_-PMP), 111.8 (C=*C*H_2_), 101.9 (benzylidene-CH), 99.5 (C-1), 76.5 (C-4), 74.6 (C-2), 69.3 (C-6), 66.8 (C-5), 55.8 (OCH_3_–PMP). ESI-HRMS: *m*/*z* calcd for C_21_H_22_O_6_ [M + Na]^+^ 393.1309, found 393.1297.

#### *p*-Methoxyphenyl 3,3′-Anhydro-4,6-*O*-benzylidene-3-*C*-hydroxymethyl-α-d-*manno-*hexopyranoside (**20**)

To a solution of compound **19** (2.2 g, 5.94 mmol, 1 equiv) in DCM (40 mL) at 0 °C was added sodium bicarbonate (0.75 g, 8.9 mmol, 1.5 equiv), followed by 3-chloroperbenzoic acid (*m*CPBA, 2.66 g, 11.87 mmol, 2 equiv). The reaction mixture was stirred for 4 h at the same temperature and quenched with saturated aqueous NaHCO_3_ (20 mL). The organic layer was extracted with DCM (40 mL), washed with saturated aqueous NaHCO_3_ (3 × 20 mL) and brine (20 mL), dried over anhydrous Na_2_SO_4,_ and concentrated in vacuo. The residue was purified by flash column chromatography by gradient elution with hexane:EtOAc (10% EtOAc in hexane to 50% EtOAc/hexane) to afford the title compound **20** (1.38 g, 3.58 mmol, 60%) as a white foam. [α]_D_^22^ +64.8 (C 1.0, CHCl_3_). ^1^H NMR (600 MHz, CDCl_3_) δ 7.45–7.42 (m, 2H, H_o_-Ph), 7.35–7.33 (m, 3H, H_p_-, H_m_-Ph), 7.01–6.98 (m, 2H, H_o_-PMP), 6.86–6.83 (m, 2H, H_m_-PMP), 5.58 (s, 1H, benzylidene-CH), 5.53 (d, *J* = 1.0 Hz, 1H, H-1), 4.44 (d, *J* = 9.7 Hz, 1H, H-4), 4.25 (dd, *J* = 10.3, 4.9 Hz, 1H, H-6a), 4.17 (td, *J* = 9.9, 4.9 Hz, 1H, H-5), 3.89 (t, *J* = 10.3 Hz, 1H, H-6b), 3.78 (s, 3H, OCH_3_-PMP), 3.76 (d, *J* = 1.0 Hz, 1H, H-2), 3.44 (d, *J* = 5.0 Hz, 1H, epoxide CH_2_), 3.01 (d, *J* = 5.0 Hz, 1H, epoxide CH_2_), 2.59 (s, 1H, OH). ^13^C­{H} NMR (151 MHz, CDCl_3_) δ 155.4 (C_p_-PMP), 149.8 (C_i_-PMP), 137.2 (C_i_-Ph), 129.3 (C_p_-Ph), 128.3 (C_m_-Ph), 126.3 (C_o_-Ph), 118.0 (C_o_-PMP), 114.9 (C_m_-PMP), 101.9 (benzylidene-CH), 99.6 (C-1), 74.6 (C-2), 72.9 (C-4), 69.0 (C-6), 65.0 (C-5), 59.5 (C-3), 55.8 (OCH_3_–PMP), 50.2 (epoxide CH_2_). ESI-HRMS: *m*/*z* calcd for C_21_H_22_O_7_ [M + Na]^+^ 409.1258, found 409.1248.

#### *p*-Methoxyphenyl 4,6-*O*-Benzylidene-3-*C*-methyl-α-d-mannopyranoside (**21**)

To a solution of compound **20** (1.37 g, 3.54 mmol, 1 equiv) in anhydrous THF (50 mL) was added a 1.0 M solution of lithium aluminum hydride (LAH, 14 mL, 14 mmol, 4 equiv) in THF at −40 °C under an argon atmosphere. The reaction mixture was stirred for 4 h at 0 °C, then quenched with saturated aquoeus ammonium chloride (10 mL), and diluted in EtOAc (80 mL). The organic layer was washed with brine (40 mL), dried over anhydrous Na_2_SO_4,_ and concentrated in vacuo. The residue was purified by flash column chromatography by gradient elution with hexane:EtOAc (10% EtOAc in hexane to 60% EtOAc/hexane) to afford the title compound **21** (1.2 g, 3.09 mmol, 87%) as a white foam. [α]_D_^22^ +121.8 (C 0.5, CH_3_OH). ^1^H NMR (600 MHz, CDCl_3_) δ 7.51–7.47 (m, 2H, H_o_-Ph), 7.40–7.35 (m, 3H, H_p_-, H_m_-Ph), 7.00–6.97 (m, 2H, H_o_-PMP), 6.86– 6.83 (m, 2H, H_m_-PMP), 5.58 (s, 1H, benzylidene-CH), 5.51 (d, *J* = 1.3 Hz, 1H, H-1), 4.22 (dd, *J* = 10.4, 4.3 Hz, 1H, H-6a), 3.99–3.93 (m, 2H, H-4, H-5), 3.87 (d, *J* = 1.3 Hz, 1H, H-2), 3.80–3.76 (m, 4H, H-6b, OCH_3_-PMP), 3.26 (br. s, 1H, CH_3_–C-O*H*), 2.69 (br. s, 1H, HC-OH), 1.63 (s, 3H, HO–C–C*H*_3_). ^13^C­{H} NMR (151 MHz, CDCl_3_) δ 155.3 (C_p_-PMP), 150.1 (C_i_-PMP), 137.5 (C_i_-Ph), 129.4 (C_p_-Ph), 128.5 (C_m_-Ph), 126.5 (C_o_-Ph), 118.0 (C_o_-PMP), 114.8 (C_m_-PMP), 102.4 (benzylidene-CH), 99.3 (C-1), 81.0 (C-4), 75.2 (C-2), 71.5 (C-3), 69.1 (C-6), 62.9 (C-5), 55.8 (OCH_3_–PMP), 20.0 (HO-C-*C*H_3_). ESI-HRMS: *m*/*z* calcd for C_21_H_24_O_7_ [M + Na]^+^ 411.1414, found 411.1406.

#### *p*-Methoxyphenyl 4,6-*O*-Benzylidene-2-*O*-*tert*-butyldimethylsilyl-3-*C*-methyl-α-d-mannopyranoside (**22**)

(1) To a solution of compound **21** (1.43 g, 3.68 mmol, 1 equiv) in dry 2,6-lutidine (40 mL) was added TBSOTf (1.3 mL, 5.66 mmol, 1.5 equiv) at 0 °C under an argon atmosphere, after which the reaction mixture was stirred vigorously at room temperature for 4 h then was quenched with MeOH (10 mL) and concentrated in vacuo. The residue was dissolved in EtOAc (100 mL) and washed with 0.2 M HCl (100 mL), dried over anhydrous Na_2_SO_4,_ and concentrated in vacuo. The residue was purified by flash column chromatography by gradient elution with hexane:EtOAc (1% EtOAc in hexane to 15% EtOAc/hexane) to afford compound **22** (1.66 g, 3.3 mmol, 89%) as a colorless viscous oil.

(2) To a solution of **29** (30 mg, 0.049 mmol, 1 equiv) in dry MeOH (1.5 mL) was added a 0.1 M solution of NaOMe (250 μL, 0.025 mmol, 0.5 equiv) in MeOH at room temperature under an argon atmosphere. After 8 h stirring, the reaction mixture was neutralized by the addition of Amberlite 120 H^+^ resin, and filtered over a pad of Celite, and the filtrate was concentrated in vacuo. The residue was purified by flash column chromatography by gradient elution with hexane:EtOAc (1% EtOAc in hexane to 15% EtOAc/hexane) to afford the title compound **22** (23 mg, 0.046 mmol, 93%) as a colorless viscous oil. [α]_D_^22^ +73.0 (C 0.5, CHCl_3_). ^1^H NMR (600 MHz, CDCl_3_) δ 7.52–7.50 (m, 2H, H_o_-Ph), 7.38–7.34 (m, 3H, H_p_-, H_m_-Ph), 6.99–6.96 (m, 2H, H_o_-PMP), 6.87–6.84 (m, 2H, H_m_-PMP), 5.60 (s, 1H, benzylidene-CH), 5.33 (d, *J* = 1.0 Hz, 1H, H-1), 4.20 (dd, *J* = 10.2, 5.0 Hz, 1H, H-6a), 3.96 (td, *J* = 9.9, 4.9 Hz, 1H, H-5), 3.84 (d, *J* = 1.0 Hz, 1H, H-2), 3.83–3.81 (d, *J* = 9.7 Hz, 1H, H-4), 3.79 (s, 3H, OCH_3_–PMP), 3.74 (t, *J* = 10.2 Hz, 1H, H-6b), 2.65 (s, 1H, OH), 1.60 (s, 3H, HO–C–C*H*_3_), 0.98 (s, 9H, Si–C­(CH_3_)_3_), 0.19 (s, 3H, Si-CH_3_), 0.17 (s, 3H, Si-CH_3_). ^13^C­{H} NMR (151 MHz, CDCl_3_) δ 155.4 (C_p_-PMP), 150.1 (C_i_-PMP), 137.7 (C_i_-Ph), 129.1 (C_p_-Ph), 128.3 (C_m_-Ph), 126.4 (C_o_-Ph), 118.2 (C_o_-PMP), 114.9 (C_m_-PMP), 102.2 (benzylidene-CH), 100.5 (C-1), 82.1 (C-4), 76.9 (C-2), 70.9 (C-3), 69.2 (C-6), 63.4 (C-5), 55.8 (OCH_3_–PMP), 25.9 (Si–C­(*C*H_3_)_3_), 18.4 (HO-C-*C*H_3_), 18.2 (Si-*C*(CH_3_)_3_), −4.5 (Si-CH_3_), −4.6 (Si-CH_3_). ESI-HRMS: *m*/*z* calcd for C_27_H_38_O_7_Si [M + Na]^+^ 525.2279, found 525.2267.

#### *p*-Methoxyphenyl 2-*O*-Benzyl-4,6-*O*-benzylidene-3-*C*-methyl-α-d-mannopyranoside (**23**)

To the mixture of compound **21** (0.88 g, 2.27 mmol, 1 equiv), TBAB (0.04 g, 0.12 mmol, 0.05 equiv), and benzyl bromide (0.55 mL, 4.62 mmol, 2 equiv) in DCM (12 mL) was added 10% aqueous NaOH (12 mL, v/v:1/1 with DCM) at room temperature. The reaction mixture was stirred for 36 h, and the organic layer was diluted in DCM (30 mL), washed with brine (30 mL), dried over anhydrous Na_2_SO_4,_ and concentrated in vacuo. The residue was purified by flash column chromatography by gradient elution with hexane:EtOAc (5% EtOAc in hexane to 25% EtOAc/hexane) to afford compound **23** (0.89 g, 1.86 mmol, 82%) as a white foam. [α]_D_^22^ +55.5 (C 1.0, CHCl_3_). ^1^H NMR (600 MHz, CDCl_3_) δ 7.54–7.52 (m, 2H, H_o_-Ph), 7.42–7.35 (m, 8H, H_p_-, H_m_-Ph; H_o_-, H_p_- H_m_-OBn), 6.98–6.95 (m, 2H, H_o_-PMP), 6.88–6.85 (m, 2H, H_m_-PMP), 5.60 (s, 1H, benzylidene-CH), 5.50 (d, *J* = 1.3 Hz, 1H, H-1), 4.79 (d, *J* = 11.4 Hz, 1H, OCH_2_Ph), 4.73 (d, *J* = 11.4 Hz, 1H, OCH_2_Ph), 4.24 (dd, *J* = 10.3, 5.0 Hz, 1H, H-6a), 4.00 (td, *J* = 9.9, 5.0 Hz, 1H, H-5), 3.90 (d, *J* = 9.7 Hz, 1H, H-4), 3.82–3.76 (m, 4H, H-6b, OCH_3_–PMP), 3.68 (d, *J* = 1.0 Hz, 1H, H-2), 3.02 (br. s, 1H, OH) 1.66 (s, 3H, HO–C–C*H*_3_). ^13^C­{H} NMR (151 MHz, CDCl_3_) δ 155.3 (C_p_-PMP), 150.1 (C_i_-PMP), 137.6 (C_i_-Ph), 137.1 (C_i_-OBn), 129.1 (C_p_-Ph), 128.8 (C_m_-Ph), 128.4 (C_p_-OBn), 128.2 (C_m_-OBn), 128.2 (C_o_-OBn), 126.4 (C_o_-Ph), 118.0 (C_o_-PMP), 114.8 (C_m_-PMP), 102.1 (benzylidene-CH), 97.7 (C-1), 83.2 (C-2), 82.0 (C-4), 74.3 (OCH_2_Ph), 70.9 (C-3), 69.0 (C-6), 63.2 (C-5), 55.7 (OCH_3_–PMP), 28.8 (HO-C-*C*H_3_). ESI-HRMS: *m*/*z* calcd for C_28_H_30_O_7_ [M + Na]^+^ 501.1884, found 501.1871.

#### *p*-Methoxyphenyl 2-O-Benzyl-4,6-O-benzylidene-3-deoxy-3-C-methylene-α-d-*arabino*-hexopyranoside (**26**)

To a solution of compound **24** ^**20**^ (0.65 g, 1.4 mmol, 1 equiv) in DCM (7 mL) was added DMP (0.89 g, 2.1 mmol, 1.5 equiv) at room temperature. The reaction mixture was stirred for 1 h, quenched by adding saturated aqueous Na_2_S_2_O_3_ (5 mL), and stirred for an additional 15 min. The organic layer was diluted with diethyl ether (3 × 10 mL), washed with saturated aqueous NaHCO_3_ (10 mL) and brine (10 mL), dried over anhydrous Na_2_SO_4,_ and concentrated in vacuo to afford crude **25** as a white foam.

To a solution of methyltriphenylphosphonium bromide (650 mg, 1.82 mmol, 1.3 equiv) in THF (6 mL) at −78 °C was added a 1.6 M solution of ^*n*^BuLi in hexane (1.05 mL, 1.68 mmol, 1.2 equiv). The resulting mixture was stirred at the same temperature for 0.5 h min and then allowed to warm to 0 °C. The reaction mixture was cooled back to −78 °C before the addition of a solution of crude **25** in THF (6 mL). After stirring for 0.5 h at −78 °C, the reaction mixture was allowed to warm to room temperature and stirred for an additional 12 h. The reaction was quenched with H_2_O (5 mL) and extracted with EtOAc (3 × 7 mL). The organic layer was washed with brine (2 × 5 mL), dried over anhydrous Na_2_SO_4,_ and concentrated in vacuo. The residue was purified by flash column chromatography by gradient elution with hexane:EtOAc (5% EtOAc in hexane to 30% EtOAc/hexane) to afford compound **26** (468 mg, 72%) as white foam. [α]_D_^22^ +88.5 (C 1.0, CHCl_3_). ^1^H NMR (600 MHz, CDCl_3_) δ 7.56 (dd, *J* = 7.7, 1.8 Hz, 2H, H_o_-Ph), 7.42–7.36 (m, 7H, H_p_-, H_m_-Ph; H_o_-, H_m_-OBn), 7.34–7.21 (m, 1H, H_p_-OBn), 6.99–6.96 (m, 2H, H_o_-PMP), 6.84–6.81 (m, 2H, H_m_-PMP), 5.70 (s, 1H, benzylidene-CH), 5.55 (t, *J* = 1.7 Hz, 1H, C=CH_2_), 5.52 (d, *J* = 1.0 Hz, 1H, H-1), 5.29 (t, *J* = 1.7 Hz, 1H, C=CH_2_), 4.74 (d, *J* = 12.1 Hz, 1H, OCH_2_Ph), 4.51–4.46 (m, 2H, H-4; OCH_2_Ph), 4.20 (dd, *J* = 10.2, 4.8 Hz, 1H, H-6a), 4.15 (d, *J* = 1.0 Hz, 1H, H-2), 4.00 (td, *J* = 9.9, 4.7 Hz, 1H, H-5), 3.88 (t, *J* = 10.3 Hz, 1H, H-6b) 3.77 (s, 3H, OCH_3_–PMP). ^13^C­{H} NMR (151 MHz, CDCl_3_) δ 155.1 (C_p_-PMP), 150.1 (C_i_-PMP), 138.2 (C_i_-Ph), 137.7 (*C*=CH_2_), 137.6 (C_i_-OBn), 129.2 (C_p_-Ph), 128.7 (C_m_-Ph), 128.4 (C_m_-OBn), 128.2 (C_o_-OBn), 128.0 (C_p_-OBn), 126.4 (C_o_-Ph), 117.9 (C_o_-PMP), 114.7 (C_m_-PMP), 113.4 (C=*C*H_2_), 101.9 (benzylidene-CH), 99.0 (C-1), 80.1 (C-2), 76.9 (C-4), 69.6 (OCH_2_Ph), 69.4 (C-6), 67.1 (C-5), 55.8 (OCH_3_–PMP). ESI-HRMS: *m*/*z* calcd for C_28_H_28_O_6_ [M + Na]^+^ 483.1778, found 483.1767.

#### *p*-Methoxyphenyl 3,3′-Anhydro-2-O-benzyl-4,6-O-benzylidene-3-C-hydroxymethyl-α-d-*manno-*hexopyranoside (**27**)

To a solution of compound **24** (175 mg, 0.38 mmol, 1 equiv) in DCM (4 mL) was added DMP (240 mg, 0.57 mmol, 1.5 equiv) at room temperature. The reaction mixture was stirred for 1 h, quenched with saturated aqueous Na_2_S_2_O_3_ (10 mL), and stirred for an additional 15 min. The organic layer was diluteded with diethyl ether (3 × 10 mL), washed with saturated aqueous NaHCO_3_ (10 mL) and brine (10 mL), dried over anhydrous Na_2_SO_4,_ and concentrated in vacuo to afford crude **25** as a white foam.

To a solution of trimethylsulfonium iodide (175 mg, 0.49 mmol, 1.3 equiv) in THF (2.0 mL) at −78 °C was added a 1.6 M solution of ^*n*^BuLi (283 μL, 0.45 mmol, 1.2 equiv) in THF under an argon atmosphere. The reaction mixture was stirred at the same temperature for 10 min and allowed to warm to 0 °C. The reaction mixture was cooled back to −78 °C before the addition of a solution of crude **25** in THF (4 mL). After being stirred for 2 h at −78 °C, the reaction mixture was allowed to warm to room temperature, quenched with H_2_O (5 mL), and extracted with EtOAc (3 × 10 mL). The combined organic layer was washed with brine (10 mL), dried over anhydrous Na_2_SO_4,_ and concentrated in vacuo. The residue was purified by flash column chromatography by gradient elution with hexane:EtOAc (1% EtOAc in hexane to 25% EtOAc/hexane) to afford compound **27** (78 mg, 0.16 mmol, 42%) as a white foam. [α]_D_^22^ +79.2 (C 2.0, CHCl_3_). ^1^H NMR (600 MHz, CDCl_3_) δ 7.46–7.44 (m, 2H, H_o_-Ph), 7.42 (d, *J* = 7.1 Hz, 2H, H_o_-OBn), 7.38–7.30 (m, 6H, H_p_-, H_m_-Ph; H_p_-, H_m_-OBn), 6.96–6.93 (m, 2H, H_o_-PMP), 6.84–6.82 (m, 2H, H_m_-PMP), 5.61 (s, 1H, benzylidene-CH), 5.49 (d, *J* = 1.2 Hz, 1H, H-1), 4.99 (d, *J* = 12.0 Hz, 1H, OCH_2_Ph), 4.76 (d, *J* = 12.0 Hz, 1H, OCH_2_Ph), 4.49 (d, *J* = 9.5 Hz, 1H, H-4), 4.23 (dd, *J* = 10.3, 4.8 Hz, 1H, H-6a), 4.14 (td, *J* = 9.9, 4.8 Hz, 1H, H-5), 3.91 (t, *J* = 10.3 Hz, 1H, H-6b), 3.78 (s, 3H, OCH_3_-PMP), 3.53 (d, *J* = 1.2 Hz, 1H, H-2), 3.30 (d, *J* = 5.2 Hz, 1H, epoxide CH_2_), 2.87 (d, *J* = 5.2 Hz, 1H, epoxide CH_2_). ^13^C­{H} NMR (151 MHz, CDCl_3_) δ 155.3 (C_p_-PMP), 149.9 (C_i_-PMP), 137.7 (C_i_-Ph), 137.2 (C_i_-Ph), 129.2 (C_p_-Ph), 128.6 (C_m_-Ph), 128.3 (C_m_-OBn), 128.2 (C_o_-OBn), 128.1 (C_p_-OBn), 126.4 (C_o_-Ph), 117.8 (C_o_-PMP), 114.8 (C_m_-PMP), 101.8 (benzylidene-CH), 99.3 (C-1), 80.2 (C-2), 73.3 (C-4), 72.7 (OCH_2_–Ph), 69.0 (C-6), 65.7 (C-5), 58.5 (C-3), 55.8 (OCH_3_–PMP), 47.0 (epoxide CH_2_). ESI-HRMS: *m*/*z* calcd for C_28_H_28_O_7_ [M + Na]^+^ 499.1727, found 499.1721.

#### *p*-Methoxyphenyl 3,3′-Anhydro-2-*O*-benzyl-4,6-*O*-benzylidene-3-*C*-hydroxymethyl-α-d-*allo-*hexopyranoside (**28**)

To a solution of compound **24** (250 mg, 0.54 mmol, 1 equiv) in DCM (6 mL) was added DMP (342 mg, 0.81 mmol, 1.5 equiv) at room temperature. The reaction mixture was stirred for 1 h, quenched with saturated aqueous Na_2_S_2_O_3_ (15 mL), and stirred for an additional 15 min. The organic layer was diluted with diethyl ether (3 × 15 mL), washed with saturated aqueous NaHCO_3_ (15 mL) and brine (15 mL), dried over anhydrous Na_2_SO_4,_ and concentrated in vacuo to afford crude **25** as a white foam.

To a solution of trimethylsulfoxonium iodide (136 mg, 0.62 mmol, 1.15 equiv) in dry DMSO (1.5 mL) at room temperature was added NaH (60% dispersion in mineral oil; 24 mg, 0.56 mmol, 1.11 equiv) while maintaining a continuous flow of argon through the reaction mixture. The reaction mixture was stirred for 0.5 h before the addition of a solution of crude **25** in THF (2 mL). After being stirred for an additional 2 h, the reaction was quenched with ice-cold water H_2_O (2 mL) and extracted with ether (3 × 10 mL). The combined organic layer was washed with brine (10 mL), dried over anhydrous Na_2_SO_4,_ and concentrated in vacuo. The residue was purified by flash column chromatography by gradient elution with hexane:EtOAc (1% EtOAc in hexane to 25% EtOAc/hexane) to afford compound **28** (110 mg, 0.23 mmol, 43%) as a white foam. [α]_D_^22^ +51.8 (C 1.0, CHCl_3_). ^1^H NMR (600 MHz, CDCl_3_) δ 7.45–7.43 (m, 2H, H_o_-Ph), 7.40–7.32 (m, 8H, H_o_-, H_p_-, H_m_-OBn; H_p_-, H_m_-Ph), 7.03–7.00 (m, 2H, H_o_-PMP), 6.85–6.82 (m, 2H, H_m_-PMP), 5.60 (s, 1H, benzylidene-CH), 5.50 (d, *J* = 1.1 Hz, 1H, H-1), 4.73–4.69 (m, 2H, OCH_2_Ph), 4.37–4.34 (m, 2H, H-4, H-5), 4.27–4.25 (m, 1H, H-6a), 3.86–3.82 (m, 1H, H-6b), 3.78 (s, 3H, OCH_3_-PMP), 3.46 (d, *J* = 1.1 Hz, 1H, H-2), 3.19 (d, *J* = 5.0 Hz, 1H, epoxide CH_2_), 2.64 (d, *J* = 5.0 Hz, 1H, epoxide CH_2_). ^13^C­{H} NMR (151 MHz, CDCl_3_) δ 155.2 (C_p_-PMP), 150.1 (C_i_-PMP), 137.3 (C_i_-Ph), 137.1 (C_i_-Ph), 129.2 (C_p_-Ph), 128.8 (C_m_-Ph), 128.5 (C_p_-OBn), 128.3 (C_m_-OBn), 128.1 (C_o_-OBn), 126.4 (C_o_-Ph), 118.1 (C_o_-PMP), 114.7 (C_m_-PMP), 102.1 (benzylidene-CH), 97.9 (C-1), 79.9 (C-2), 72.5 (C-4), 72.4 (OCH_2_–Ph), 69.3 (C-6), 62.8 (C-5), 55.8 (C-3), 55.8 (OCH_3_–PMP), 46.5 (epoxide CH_2_). ESI-HRMS: *m*/*z* calcd for C_28_H_28_O_7_ [M + Na]^+^ 499.1727, found 499.1717.

#### *p*-Methoxyphenyl 2-*O*-Benzoyl-4,6-*O*-benzylidene-3-*O*-*tert*-butyldimethylsilyl-3-*C*-methyl-α-d-mannopyranoside (**29**)

A mixture of benzoic acid (50 mg, 0.409 mmol, 5.1 equiv) and 1,1’-carbonyl diimidazole (CDI, 66 mg, 0.407 mmol, 5.1 equiv) in THF (1.5 mL) was stirred for 1 h at room temperature under an argon atmosphere. To this reaction mixture was added a solution of compound **22** (40 mg, 0.079 mmol, 1 equiv) in THF (1 mL), followed by 1,8-diazabicyclo[5.4.0]­undec-7-ene (DBU, 25 μL, 0.167 mmol, 2.1 equiv). After stirring for 24 h at room temperature, an additional amount of DBU (25 μL, 0.167 mmol, 2.1 equiv) was added to the reaction mixture, and stirring continued for another 24 h. The reaction mixture was diluted in EtOAc (15 mL) and washed with saturated aqueous NaHCO_3_ (3 × 10 mL) and brine (10 mL). The organic layer was dried over anhydrous Na_2_SO_4_ and concentrated in vacuo. The residue was purified by flash column chromatography by gradient elution with hexane:EtOAc (1% EtOAc in hexane to 20% EtOAc/hexane) to afford compound **29** (37 mg, 0.061 mmol, 77%) as a white foam. [α]_D_^22^ +27.0 (C 0.5, CHCl_3_). ^1^H NMR (600 MHz, CDCl_3_) δ 8.13–8.11 (m, 2H, H_o_-OBz), 7.61–7.58 (m, 1H, H_p_-OBz), 7.54–7.52 (m, 2H, H_o_-Ph), 7.48 (t, *J* = 7.7 Hz, 2H, H_m_-OBz), 7.41–7.36 (m, 3H, H_p_-, H_m_-Ph), 7.02–6.98 (m, 2H, H_o_-PMP), 6.85–6.82 (m, 2H, H_m_-PMP), 5.67 (s, 1H, benzylidene-CH), 5.44 (d, *J* = 1.4 Hz, 1H, H-1), 5.41 (d, *J* = 1.4 Hz, 1H, H-2), 4.29 (dd, *J* = 10.3, 4.1 Hz, 1H, H-6a), 4.11–4.06 (m, 2H, H-4, H-5), 3.88–3.85 (m, 1H, H-6b), 3.78 (s, 3H, OCH_3_–PMP), 1.76 (s, 3H, TBSO–C–CH_3_), 0.64 (s, 9H, Si–C­(CH_3_)_3_), 0.00 (s, 3H, Si-CH_3_), 0.00 (s, 3H, Si-CH_3_). ^13^C­{H} NMR (151 MHz, CDCl_3_) δ 165.6 (C=O), 155.4 (C_p_-PMP), 150.3 (C_i_-PMP), 137.5 (C_i_-Ph), 133.4 (C_p_-OBz), 130.1 (C_i_-OBz), 130.1 (C_o_-OBz), 129.3 (C_p_-Ph), 128.5 (C_m_-OBz), 128.4 (C_m_-Ph), 126.5 (C_o_-Ph), 118.2 (C_o_-PMP), 114.8 (C_m_-PMP), 102.4 (benzylidene-CH), 98.9 (C-1), 82.2 (C-4), 76.3 (C-2), 73.4 (C-3), 69.3 (C-6), 63.6 (C-5), 55.8 (OCH_3_–PMP), 25.7 (Si–C­(*C*H_3_)_3_), 20.6 (HO-C-*C*H_3_), 18.2 (Si-*C*(CH_3_)_3_), −2.5 (Si-CH_3_), −2.6 (Si-CH_3_). ESI-HRMS: *m*/*z* calcd for C_34_H_42_O_8_Si [M + Na]^+^ 629.2541, found 629.2529.

#### *p*-Methoxyphenyl 3-*O*-(Benzoyl-α-^**13**^C)-2-*O*-benzyl-4,6-*O*-benzylidene-3-*C*-methyl-α-d-mannopyranoside (**30**)

A mixture of benzoic acid-α-^13^C (193 mg, 1.567 mmol, 5 equiv) and CDI (254 mg, 1.566 mmol, 5 equiv) in THF (1.5 mL) was stirred for 1 h at room temperature under an argon atmosphere. To this reaction mixture was added a solution of compound **23** (150 mg, 0.313 mmol, 1 equiv) in THF (1 mL), followed by DBU (95 μL, 0.64 mmol, 2 equiv). After stirring for 24 h at 35 °C, an additional amount of DBU (95 μL, 0.64 mmol, 2 equiv) was added to the reaction mixture, and stirring continued for another 24 h. The reaction mixture was diluted in EtOAc (10 mL) and washed with saturated aqueous NaHCO_3_ (3 × 5 mL) and brine (5 mL). The organic layer was dried over anhydrous Na_2_SO_4_ and concentrated in vacuo. The residue was purified by flash column chromatography by gradient elution with hexane:EtOAc (1% EtOAc in hexane to 20% EtOAc/hexane) to afford compound **30** (166 mg, 0.096 mmol, 91%) as white foam. [α]_D_^22^ −2.1 (C 2.0, CHCl_3_). ^1^H NMR (600 MHz, CDCl_3_) δ 8.08 (dd, *J* = 7.3, 4.1 Hz, 2H, H_o_-OBz), 7.59–7.57 (m, 3H, H_p_-OBz; H_o_-Ph), 7.46–7.38 (m, 5H, H_m_-OBz; H_p_-, H_m_-Ph), 7.22–7.16 (m, 5H, H_o_-, H_m_-, H_p_-OBn), 7.02–6.99 (m, 2H, H_o_-PMP), 6.90–6.87 (m, 2H, H_m_-PMP), 5.76 (s, 1H, benzylidene-CH), 5.50 (s, 1H, H-1), 4.91 (s, 1H, H-2), 4.65 (d, *J* = 11.0 Hz, 1H, OCH_2_Ph), 4.61 (d, *J* = 11.0 Hz, 1H, OCH_2_Ph), 4.38 (d, *J* = 9.8 Hz, 1H, H-4), 4.31 (dd, *J* = 10.3, 5.0 Hz, 1H, H-6a), 4.17 (dt, *J* = 10.0, 5.1 Hz, 1H, H-5), 3.90 (t, *J* = 10.2 Hz, 1H, H-6b), 3.81 (s, 3H, OCH_3_-PMP), 2.12 (s, 3H, α-^13^C-BzO–C–CH_3_). ^13^C­{H} NMR (151 MHz, CDCl_3_) δ 166.2 (^13^C=O), 155.3 (C_p_-PMP), 150.3 (C_i_-PMP), 137.7 (C_i_-Ph), 137.5 (C_i_-OBn), 132.9 (C_p_-OBz), 131.4 (d, *J* = 74.5 Hz; C_i_-OBz), 129.8 (d, *J* = 2.5 Hz; C_o_-OBz), 129.1 (C_p_-Ph), 128.4 (C_m_-Ph; C_m_-OBz), 128.3 (C_m_-OBn), 128.2 (C_o_-OBn), 127.9 (C_p_-OBn), 126.3 (C_o_-Ph), 118.1 (C_o_-PMP), 114.8 (C_m_-PMP), 101.8 (benzylidene-CH), 97.9 (C-1), 81.3 (d, *J* = 2.7 Hz; C-3), 80.3 (C-2), 79.7 (d, *J* = 3.7 Hz; C-4), 73.8 (OCH_2_Ph), 69.1 (C-6), 62.3 (C-5), 55.8 (OCH_3_–PMP), 16.7 (α-^13^C-BzO-C-*C*H_3_). ESI-HRMS: *m*/*z* calcd for C_34_^13^CH_34_O_8_ [M + Na]^+^ 606.2179, found 606.2152.

#### Acetyl 2-*O*-Benzyl-4,6-*O*-benzylidene-3-*C*-methyl-d-mannopyranose (**32**)

To a solution of **23** (1.2 g, 2.51 mmol, 1 equiv) in an ACN/DCM/H_2_O mixture (v/v/v–2/1/1; 40 mL) at 0 °C was added cerium ammonium nitrate (CAN, 2.1 g, 3.76 mmol, 1.5 equiv). The resulting mixture was stirred for 4 h before additional CAN (1.38 g, 2.51 mmol, 1 equiv) was added and stirring continued for 1 h. The reaction mixture was quenched by the addition of a saturated aqueous NaHCO_3_ (20 mL), and the organic layer was diluted with EtOAc (3 × 60 mL), washed with brine (50 mL), dried over anhydrous Na_2_SO_4,_ and concentrated in vacuo. The residue was dissolved in pyridine (16 mL) and acetic anhydride (6 mL) and stirred overnight at room temperature before the reaction mixture was concentrated under reduced pressure. The residue was purified by flash column chromatography by gradient elution with hexane:EtOAc (5% EtOAc in hexane to 25% EtOAc/hexane) to afford compound **32** (0.61 g, 1.47 mmol, 59%, α:β mixture of anomers ∼ 2.55:1) as a colorless solid. ^1^H NMR (600 MHz, CDCl_3_) δ 7.51–7.48 (m, 4H, H_o_-Ph-α, H_o_-Ph-β), 7.44 (d, *J* = 7.2 Hz, 2H, H_o_-OBn-β), 7.41–7.33 (m, 14H,; H_p_-, H_m_-Ph-α; H_p_-, H_m_-Ph-β; H_o_-, H_m_-, H_p_-OBn-α; H_m_-, H_p_-OBn-β), 6.27 (s, 1H, H-1-α), 5.98 (s, 1H, H-1-β), 5.57 (s, 1H, benzylidene-CH-α), 5.54 (s, 1H, benzylidene-CH-β), 5.02 (d, *J* = 11.3 Hz, 1H, OCH_2_Ph-β), 4.83 (d, *J* = 11.3 Hz, 1H, OCH_2_Ph-α), 4.71 (d, *J* = 11.3 Hz, 1H, OCH_2_Ph-β), 4.64 (d, *J* = 11.3 Hz, 1H, OCH_2_Ph-α), 4.35 (dd, *J* = 10.4, 4.9 Hz, 1H, H-6a-β), 4.30 (dd, *J* = 10.3, 4.8 Hz, 1H, H-6a-β), 3.91 (td, *J* = 9.8, 4.8 Hz, 1H, H-5-α), 3.87–3.83 (m, 2H, H-4-α, H-4-β), 3.80 (t, *J* = 10.1 Hz, 1H, H-6b-β), 3.76 (t, *J* = 10.1 Hz, 1H, H-6b-α), 3.62–3.58 (m, 2H, H-2-β, H-5-β), 3.46 (s, 1H, H-2-α), 3.00 (s, 1H, OH-α), 2.89 (s, 1H, OH-β), 2.15 (s, 3H, HO–C–C*H*_3_-α), 2.14 (s, 3H, HO–C–C*H*_3_-β), 1.51 (s, 3H, CH_3_CO_2_-α), 1.43 (s, 3H, CH_3_CO_2_-β). ^13^C­{H} NMR (151 MHz, CDCl_3_) δ 169.0 (C=O-α), 168.8 (C=O-β), 137.6 (C_i_-Ph-β), 137.6 (C_i_-Ph-α), 137.3 (C_i_-OBn-β), 136.8 (C_i_-OBn-α), 129.1 (C_p_-Ph-β), 129.1 (C_p_-Ph-α), 128.7 (C_m_-Ph-α), 128.7 (C_m_-Ph-β), 128.4 (C_m_-OBn-β), 128.4 (C_p_-OBn-α), 128.3 (C_p_-OBn-β), 128.2 (C_m_-OBn-β), 128.2 (C_o_-OBn-α), 128.2 (C_o_-OBn-α), 126.3 (C_o_-Ph-α; C_o_-Ph-β), 102.1 (benzylidene-CH-β), 102.1 (benzylidene-CH-α), 92.4 (C-1-β), 91.7 (C-1-α), 83.1 (C-2-β), 82.0 (C-2-α), 81.4 (C-4-β), 81.4 (C-4-α), 76.5 (OCH_2_Ph-β), 73.9 (OCH_2_Ph-α), 72.1 (C-3-β), 70.3 (C-3-α), 68.8 (C-6-α), 68.7 (C-6-β), 66.9 (C-5-β), 64.8 (C-5-α), 21.2 (HO-C-*C*H_3_-α), 21.0 (HO-C-*C*H_3_-β), 18.4 (*C*H_3_CO_2_-α), 18.1 (*C*H_3_CO_2_-β). ESI-HRMS: *m*/*z* calcd for C_23_H_26_O_7_ [M + Na]^+^ 437.1571, found 437.1562.

#### *p*-Methylphenyl 2-*O*-Benzyl-4,6-*O*-benzylidene-3-*C*-methyl-thio-α-d-mannopyranoside (**33**)

To a mixture of **32** (100 mg, 0.241 mmol, 1 equiv) and *p*-methoxybenzenethiol (60 mg, 0.483 mmol, 2 equiv) in DCM (10 mL) was added boron trifluoride diethyl etherate (BF_3_·OEt_2_, 60 μL, 0.486 mmol, 2 equiv) at −20 °C under an argon atmosphere. The reaction mixture was stirred for 6 h at 0 °C, quenched by the addition of Et_3_N (0.1 mL), and concentrated in vacuo. The residue was purified by flash column chromatography by gradient elution with hexane:EtOAc (1% EtOAc in hexane to 20% EtOAc/hexane) to afford compound **33** (71 mg, 0.148 mmol, 61%) as a white foam. [α]_D_^22^ +99.2 (C 0.5, CHCl_3_). ^1^H NMR (600 MHz, CDCl_3_) δ 7.55 (dd, *J* = 7.8, 1.8 Hz, 2H, H_o_-Ph), 7.41–7.36 (m, 10H, H_p_-, H_m_-Ph; H_o_-, H_m_-, H_p_-OBn; H_o_-STol), 7.17 (d, *J* = 8.0 Hz, 2H, H_m_-STol), 5.60 (s, 1H, benzylidene-CH), 5.54 (s, 1H, H-1), 4.74 (d, *J* = 11.3 Hz, 1H, OCH_2_Ph), 4.62 (d, *J* = 11.3 Hz, 1H, OCH_2_Ph), 4.33–4.28 (m, 2H, H-6a; H-5), 3.90 (d, *J* = 9.4 Hz, 1H, H-4), 3.84–3.80 (m, 1H, H-6b), 3.78 (s, 1H, H-2), 3.12 (s, 1H, OH), 2.38 (s, 3H, CH_3_-STol), 1.64 (s, 3H, HO–C–CH_3_). ^13^C­{H} NMR (151 MHz, CDCl_3_) δ 138.2 (C_p_-STol), 137.6 (C_i_-Ph), 136.9 (C_i_-OBn), 132.4 (C_o_-STol), 131.1 (C_i_-STol), 130.1 (C_m_-STol), 129.1 (C_p_-Ph), 128.8 (C_m_-Ph), 128.4 (C_p_-OBn), 128.3 (C_m_-OBn), 128.3 (C_o_-OBn), 126.5 (C_o_-Ph), 102.1 (benzylidene-CH), 86.6 (^1^ *J*_CH_ = 165 Hz; C-1), 85.4 (C-2), 82.4 (C-4), 73.5 (OCH_2_Ph), 70.8 (C-3), 68.9 (C-6), 63.4 (C-5), 21.2 (CH_3_–STol) 18.9 (HO-C-*C*H_3_). ESI-HRMS: *m*/*z* calcd for C_28_H_30_O_5_S [M + Na]^+^ 501.1706, found 501.1701.

## Supplementary Material



## Data Availability

The data underlying this study are available in the published article and its Supporting Information.

## Notes

Copies of ^1^H, ^13^C­{H}, ^1^H–^1^H COSY, ^1^H–^13^C HSQC, and ^1^H–^13^C HMBC NMR spectra for all new compounds (PDF)
