# Supplementary material for: Synthesis of 3‑C‑Methyl‑d‑Mannopyranoside Derivatives Functionalized at the 3‑Position
Source: ACS Omega. 2026 Feb 19;11(8):13957–66. doi: 10.1021/acsomega.5c12799 (PMC12961483; doi:10.1021/acsomega.5c12799)

Supporting Information

for

Synthesis of 3-*C*-Methyl-D-Mannopyranoside Derivatives Functionalized at the 3-Position

Shuay Abdullayev,[a,b] Vikram A. Sarpe[a,b] and David Crich[a,b,c,*]

a) Department of Pharmaceutical and Biomedical Sciences, University of Georgia, 250 West Green Street, Athens, GA 30602, USA

b) Complex Carbohydrate Research Center, University of Georgia, 315 Riverbend Road, Athens, GA 30602, USA

c) Department of Chemistry, University of Georgia, 302 East Campus Road, Athens, GA 30602, USA

Email: David.Crich@uga.edu

Table of Contents

1H, 13C{1H}, COSY, HSQC, HMBC NMR Spectra of Compounds S2

^{1}H, ^{13}C{^{1}H}, COSY, HSQC, HMBC NMR Spectra of Compounds

Figure S1. ^{1}H NMR (600 MHz, CD_3OD) spectrum of *p*-methoxyphenyl 3-*O*-*p*-methoxybenzyl-α-D-mannopyranoside **12**:

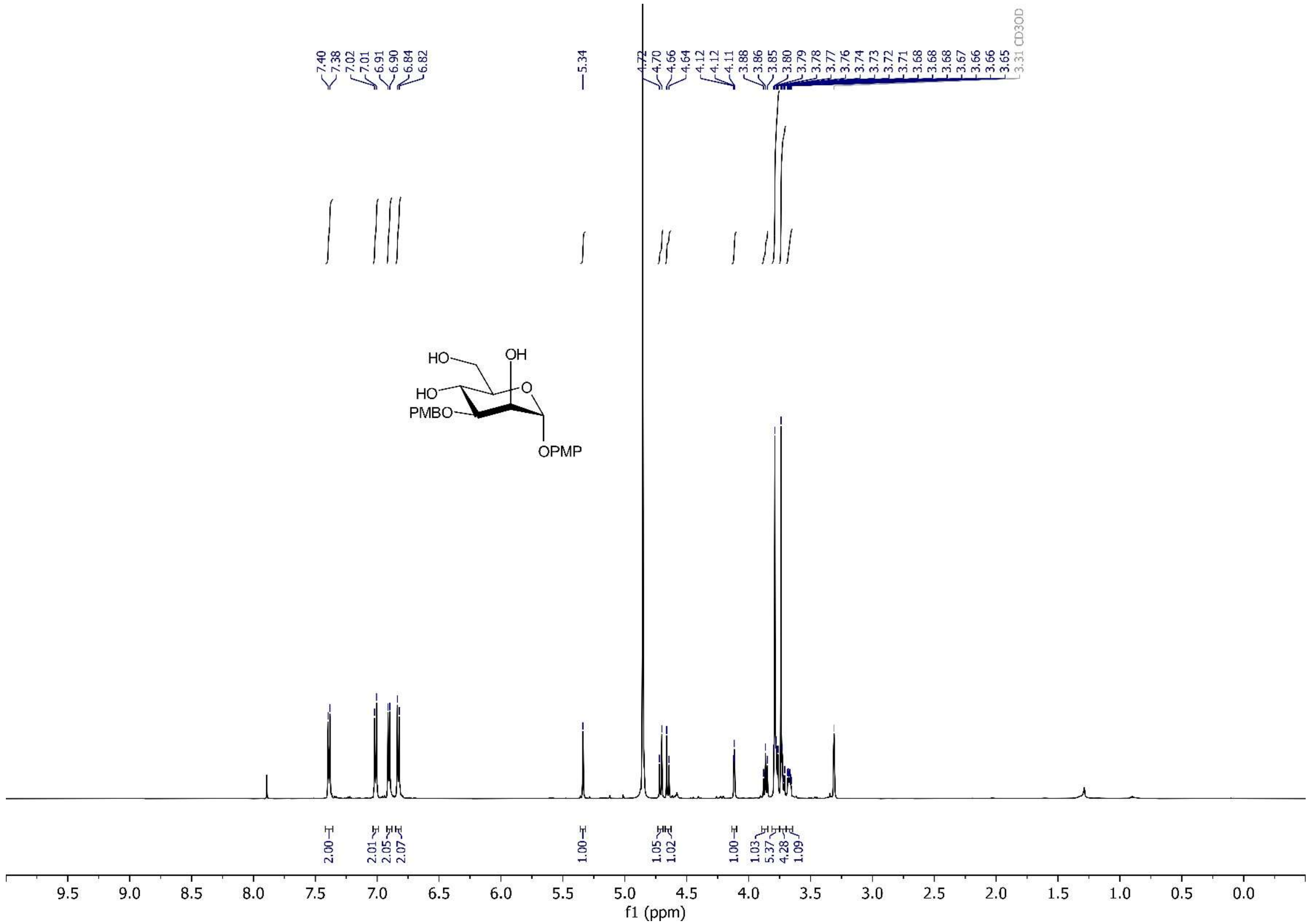

Figure S2. COSY NMR (600 MHz, CD_3OD) spectrum of *p*-methoxyphenyl 3-*O*-*p*-methoxybenzyl-α-D-mannopyranoside **12**:

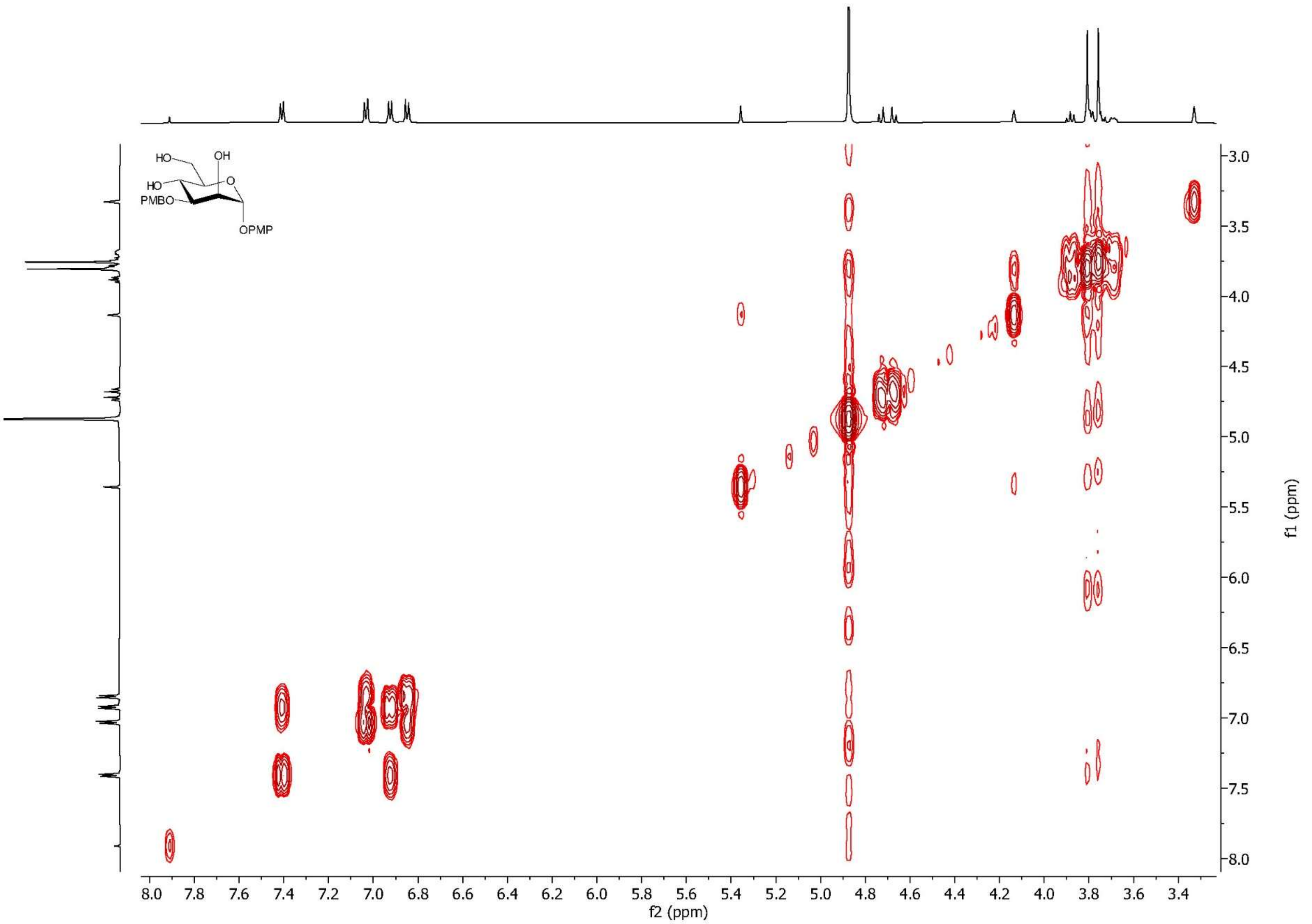

Figure S3. $^{13}C\{H\}$ NMR (151 MHz, CD_3OD) spectrum of *p*-methoxyphenyl 3-*O*-*p*-methoxybenzyl-α-D-mannopyranoside **12**:

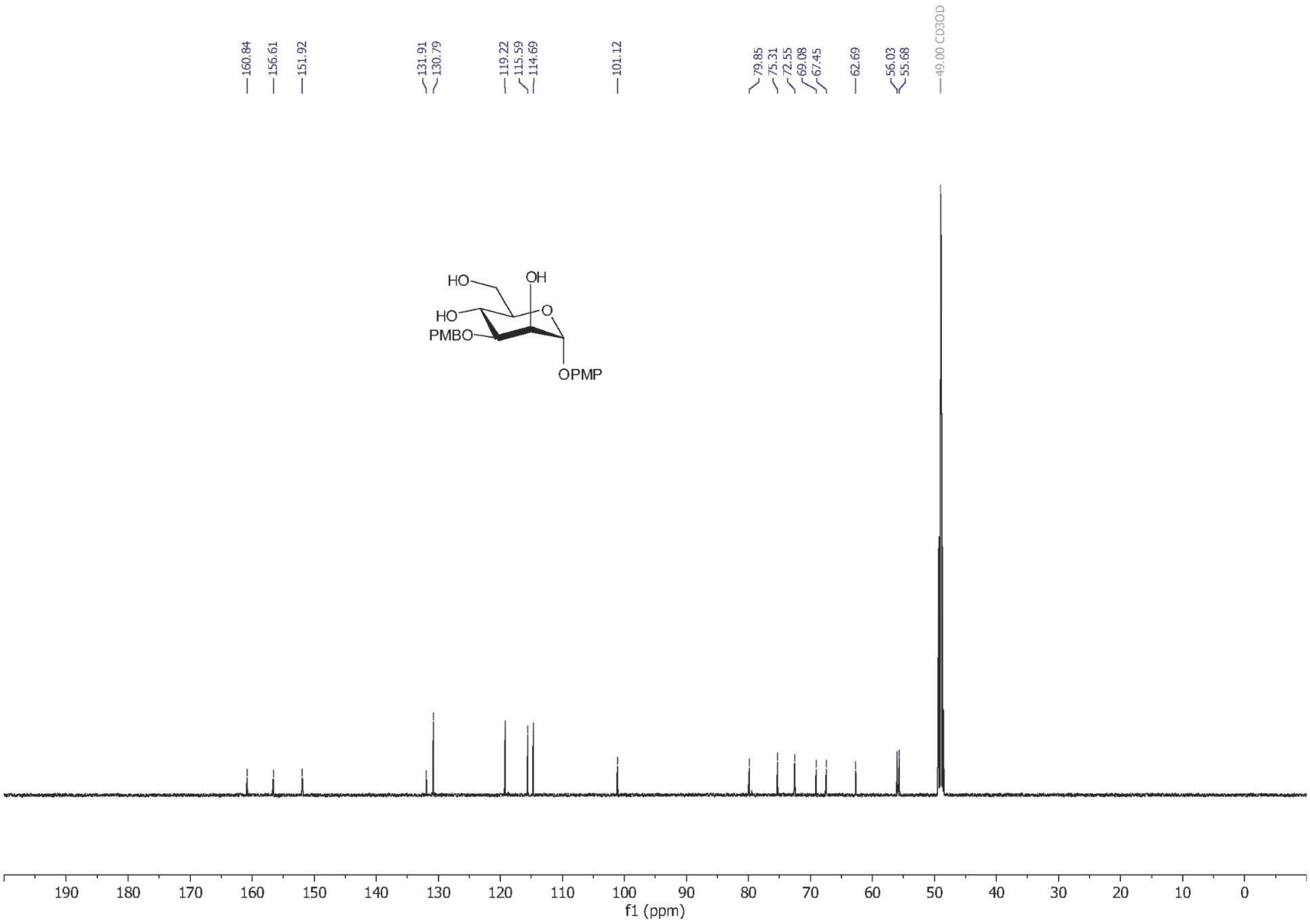

Figure S4. HSQC NMR (600 MHz, CD_3OD) spectrum of *p*-methoxyphenyl 3-*O*-*p*-methoxybenzyl-α-D-mannopyranoside **12**:

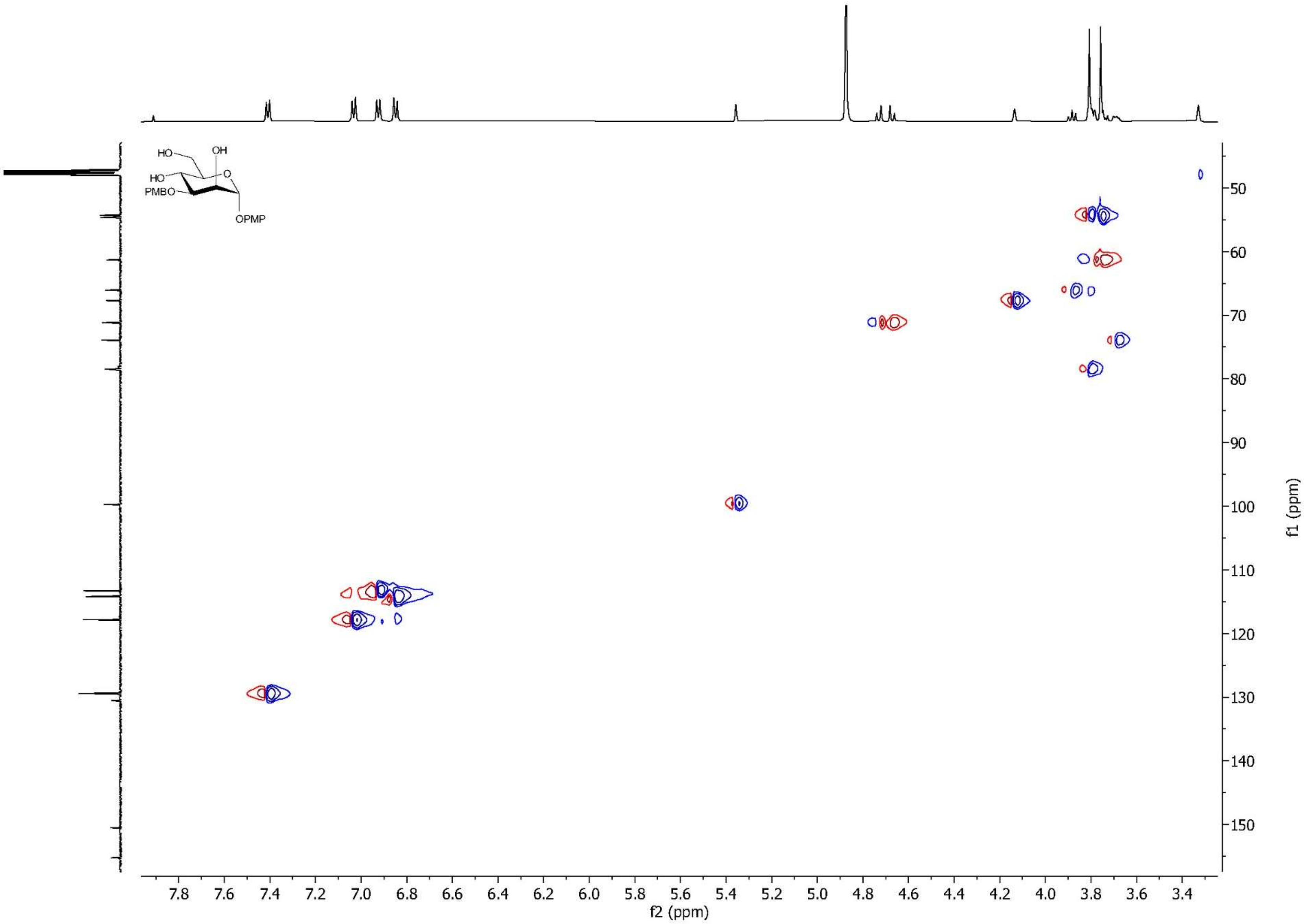

Figure S5. HMBC NMR (600 MHz, CD_3OD) spectrum of *p*-methoxyphenyl 3-O-*p*-methoxybenzyl-α-D-mannopyranoside **12**:

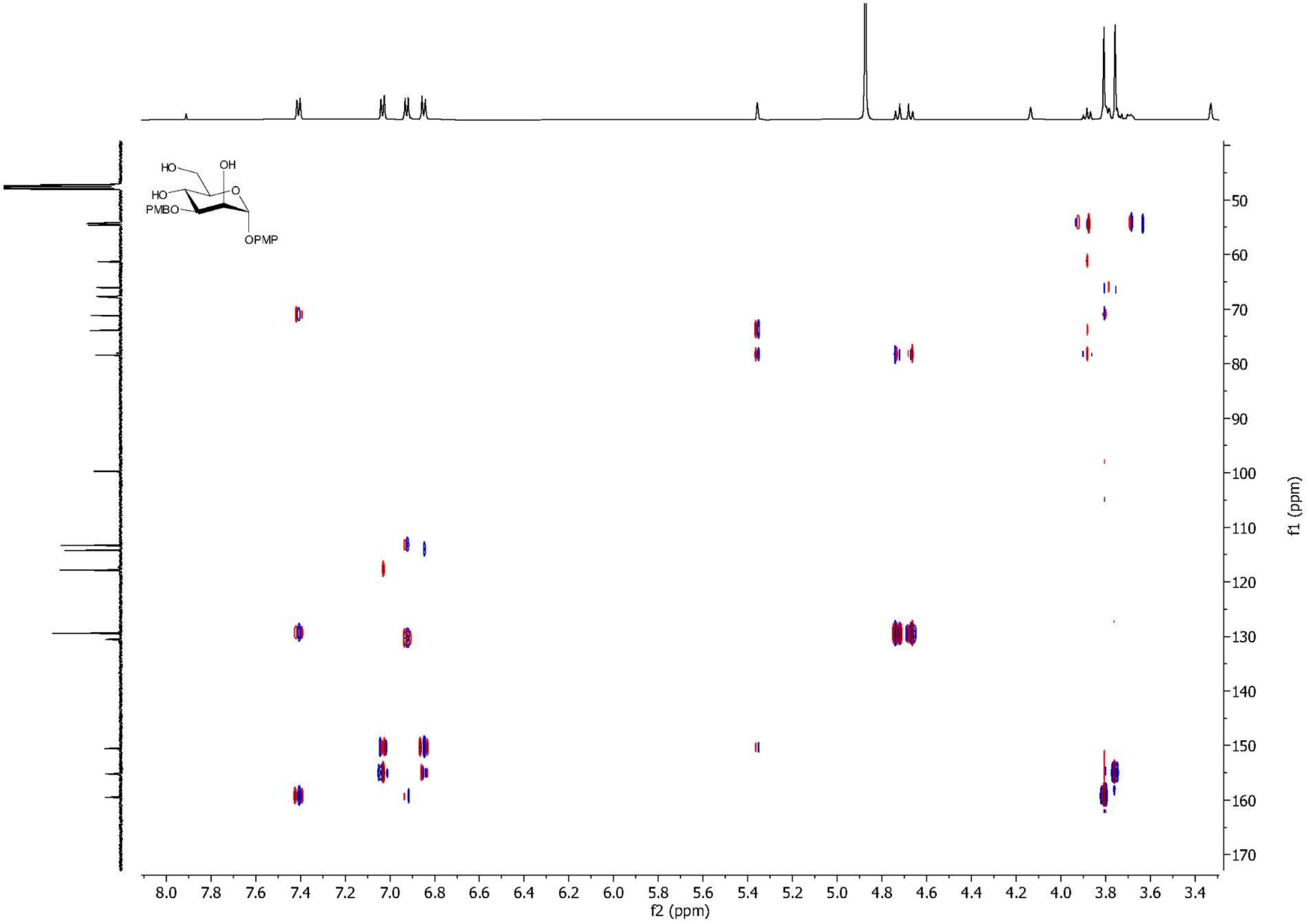

Figure S6. ^{1}H NMR (600 MHz, $CDCl_3$) spectrum of *p*-methoxyphenyl 4,6-*O*-benzylidene-3-*O*-*p*-methoxybenzyl-α-D-mannopyranoside **13**:

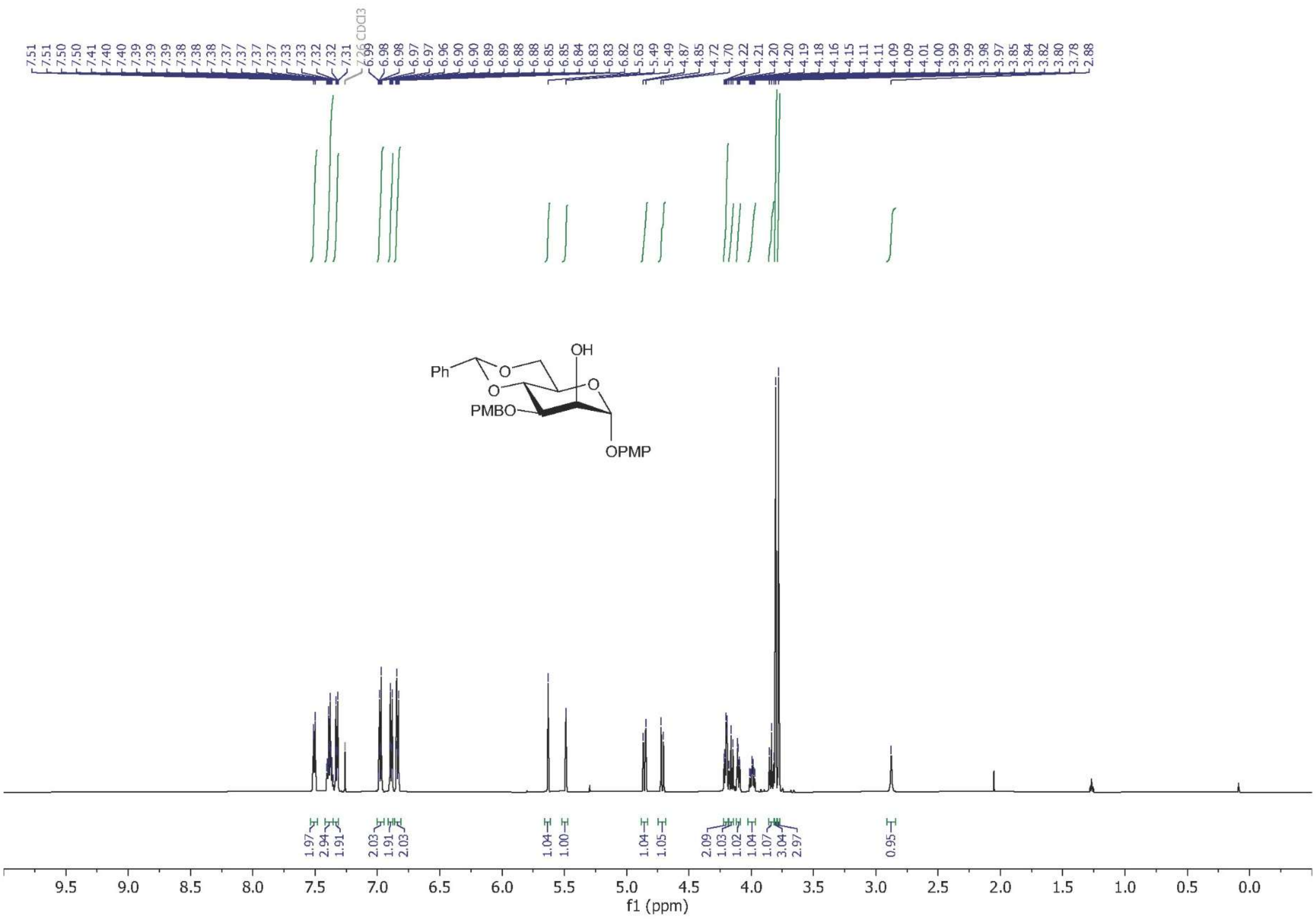

Figure S7. COSY NMR (600 MHz, $CDCl_3$) spectrum of *p*-methoxyphenyl 4,6-*O*-benzylidene-3-*O*-*p*-methoxybenzyl-α-D-mannopyranoside **13**:

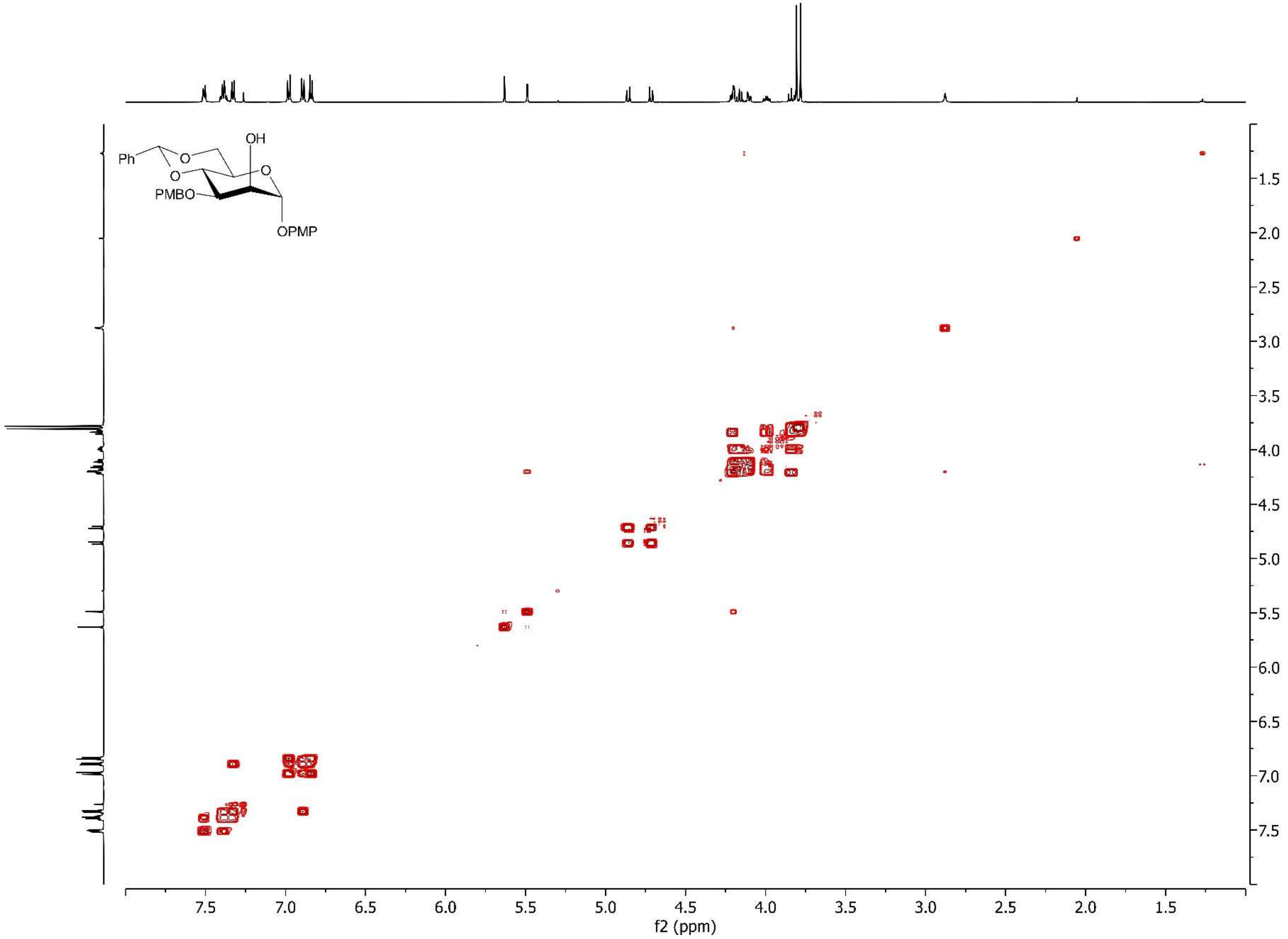

Figure S8. ^{13}C{H} NMR (151 MHz, $CDCl_3$) spectrum of *p*-methoxyphenyl 4,6-*O*-benzylidene-3-*O*-*p*-methoxybenzyl-α-D-mannopyranoside **13**:

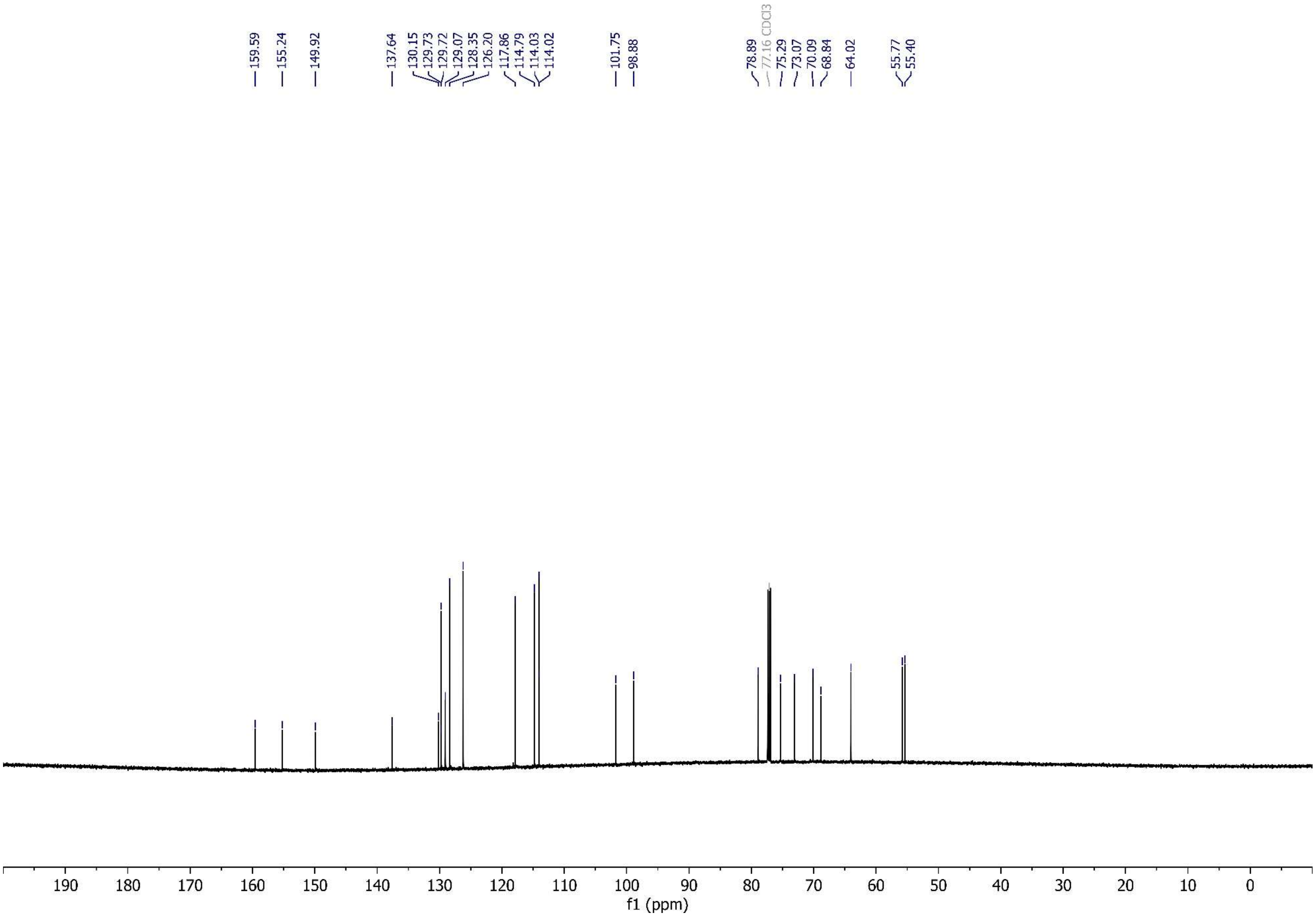

Figure S9. HSQC NMR (600 MHz, $CDCl_3$) spectrum of *p*-methoxyphenyl 4,6-*O*-benzylidene-3-*O*-*p*-methoxybenzyl-α-D-mannopyranoside **13**:

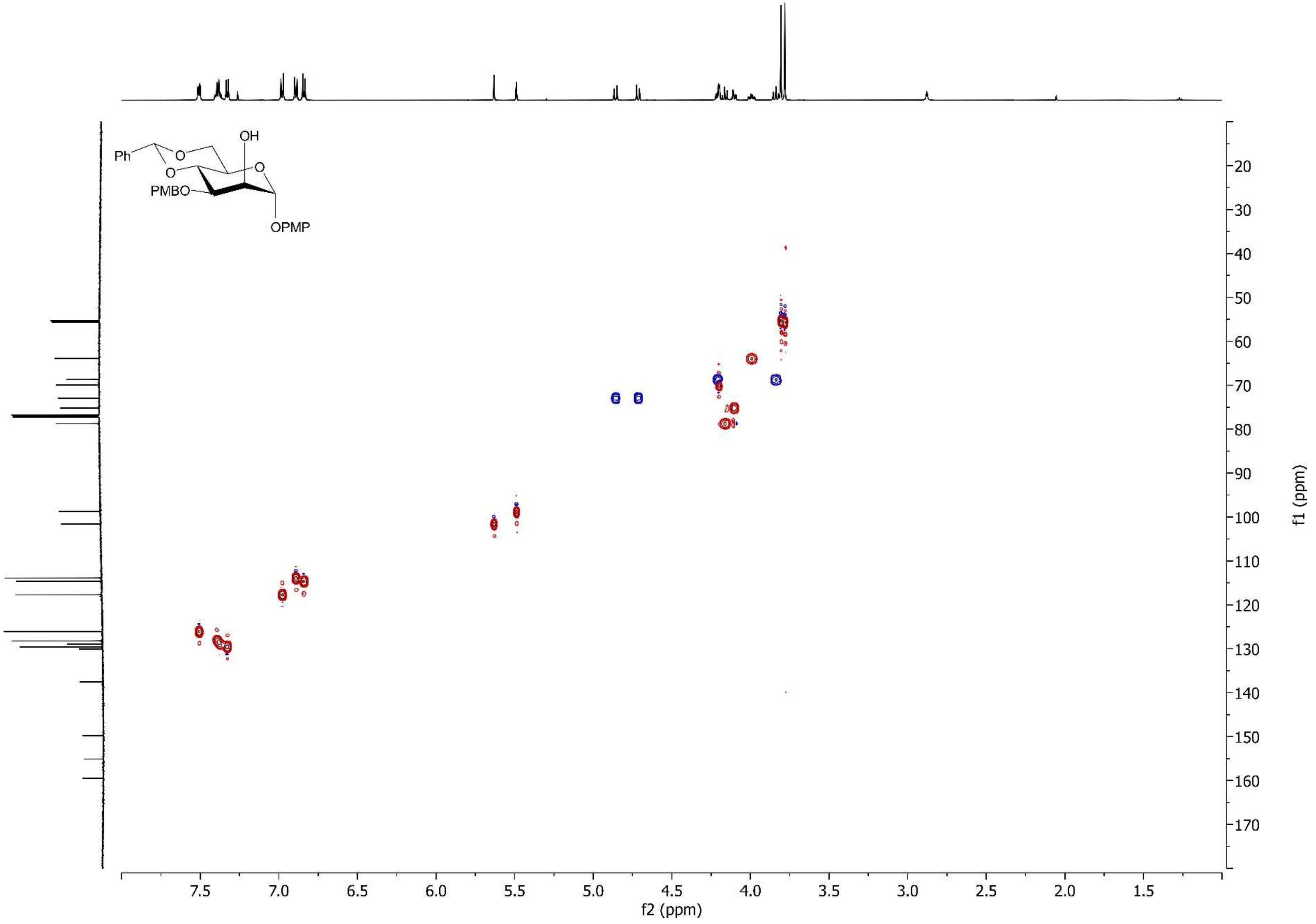

Figure S10. HMBC NMR (600 MHz, $CDCl_3$) spectrum of *p*-methoxyphenyl 4,6-*O*-benzylidene-3-*O*-*p*-methoxybenzyl-α-D-mannopyranoside **13**:

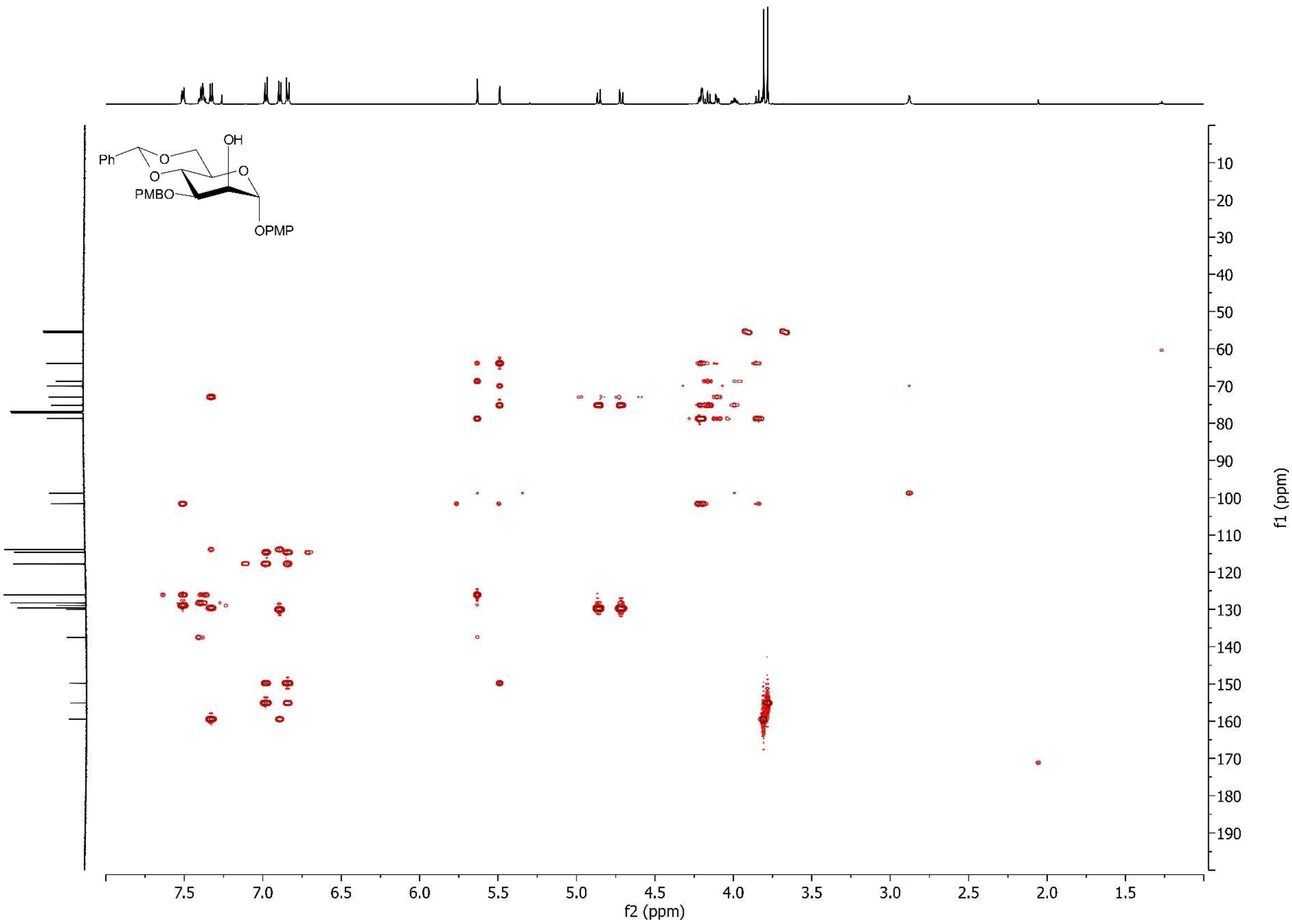

Figure S11. ^{1}H NMR (600 MHz, $CDCl_3$) spectrum of *p*-methoxyphenyl 4,6-*O*-benzylidene-3-*O*-*p*-methoxybenzyl-2-*O*-*tert*-butyldimethylsilyl-α-D-mannopyranoside **14**:

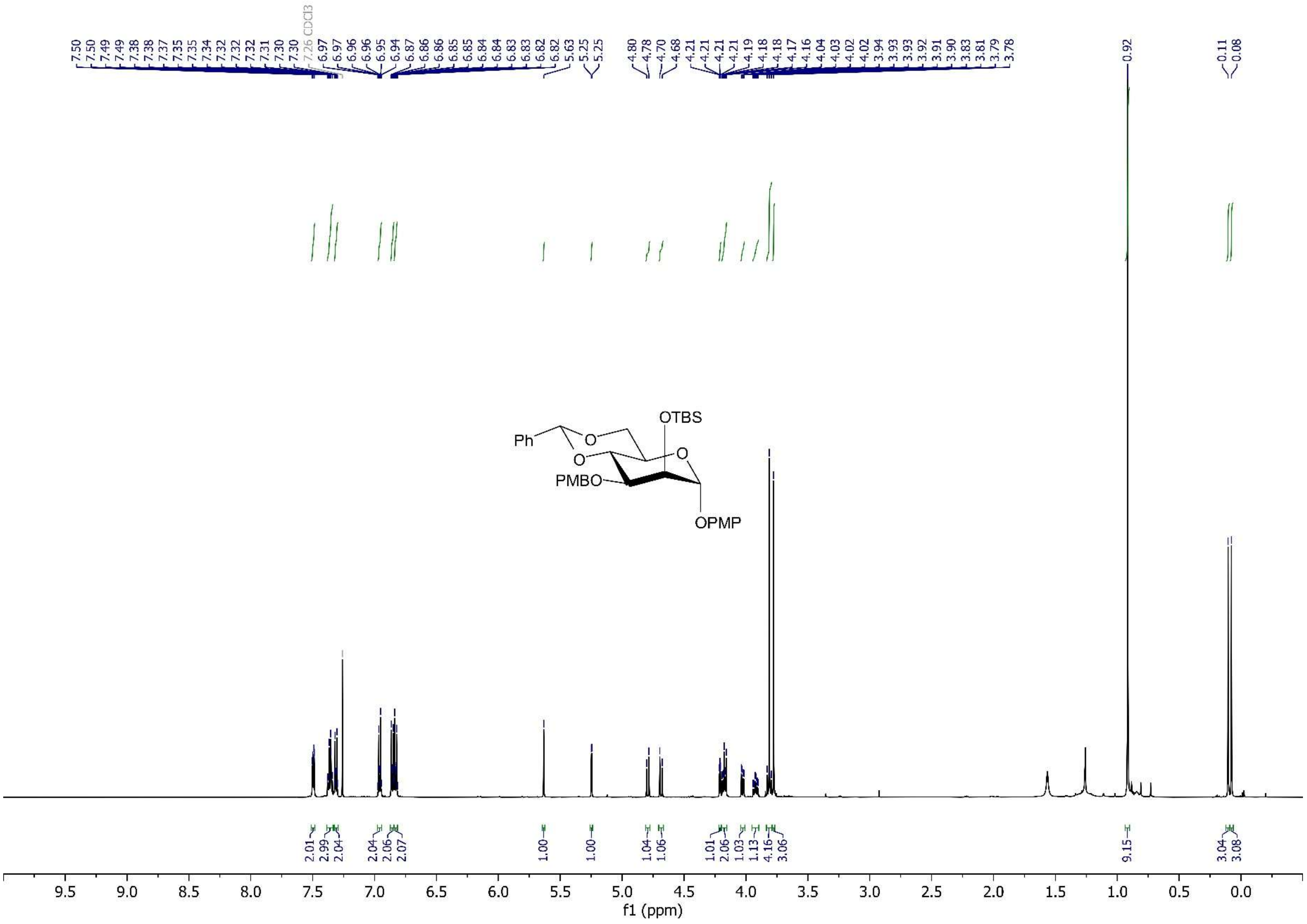

Figure S12. COSY NMR (600 MHz, $CDCl_3$) spectrum of *p*-methoxyphenyl 4,6-*O*-benzylidene-3-*O*-*p*-methoxybenzyl-2-*O*-*tert*-butyldimethylsilyl-α-D-mannopyranoside **14**:

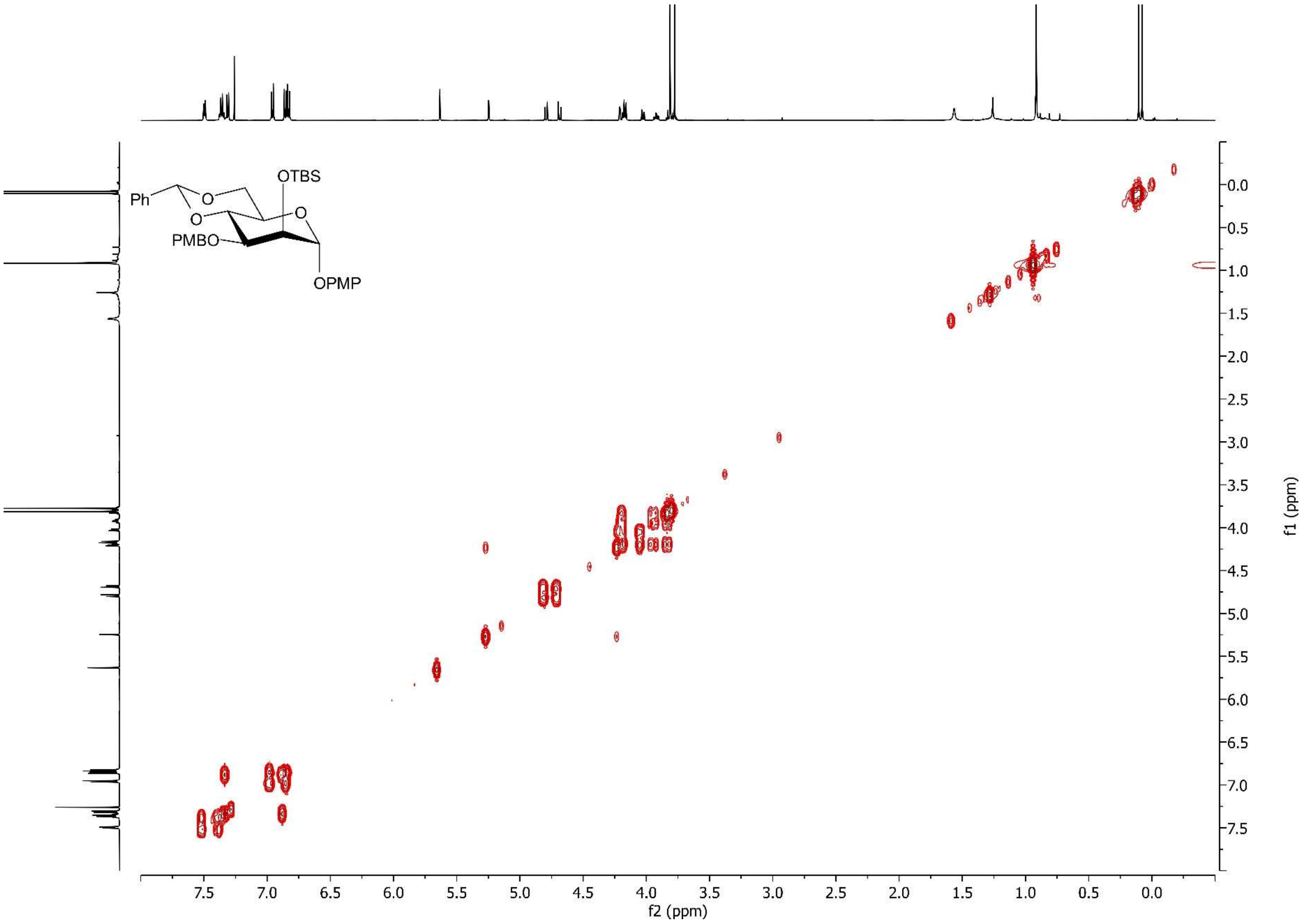

Figure S13. ^{13}C{H} NMR (151 MHz, $CDCl_3$) spectrum of *p*-methoxyphenyl 4,6-*O*-benzylidene-3-*O*-*p*-methoxybenzyl-2-*O*-*tert*-butyldimethylsilyl-α-D-mannopyranoside **14**:

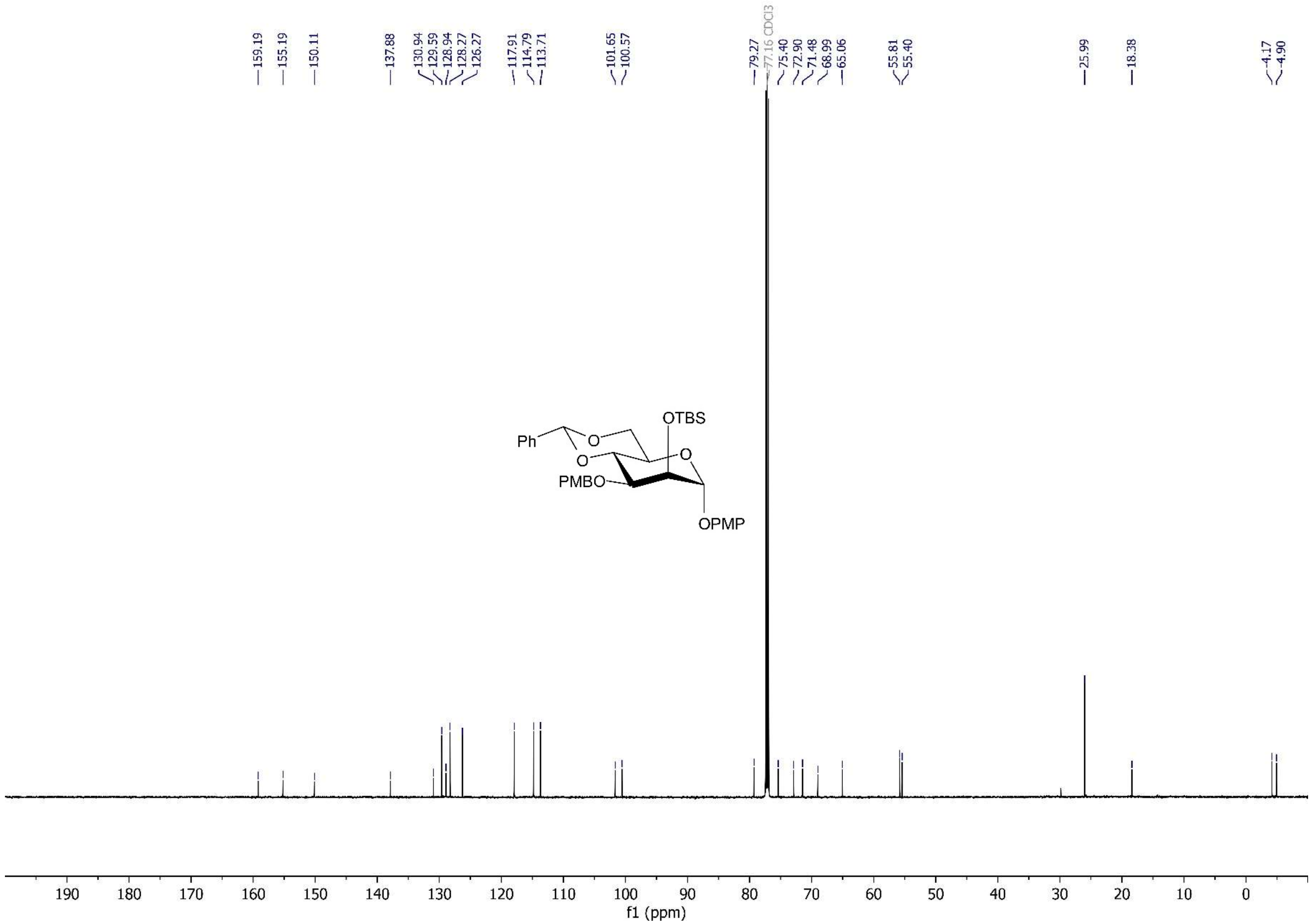

Figure S14. HSQC NMR (600 MHz, $CDCl_3$) spectrum of *p*-methoxyphenyl 4,6-*O*-benzylidene-3-*O*-*p*-methoxybenzyl-2-*O*-*tert*-butyldimethylsilyl-α-D-mannopyranoside **14**:

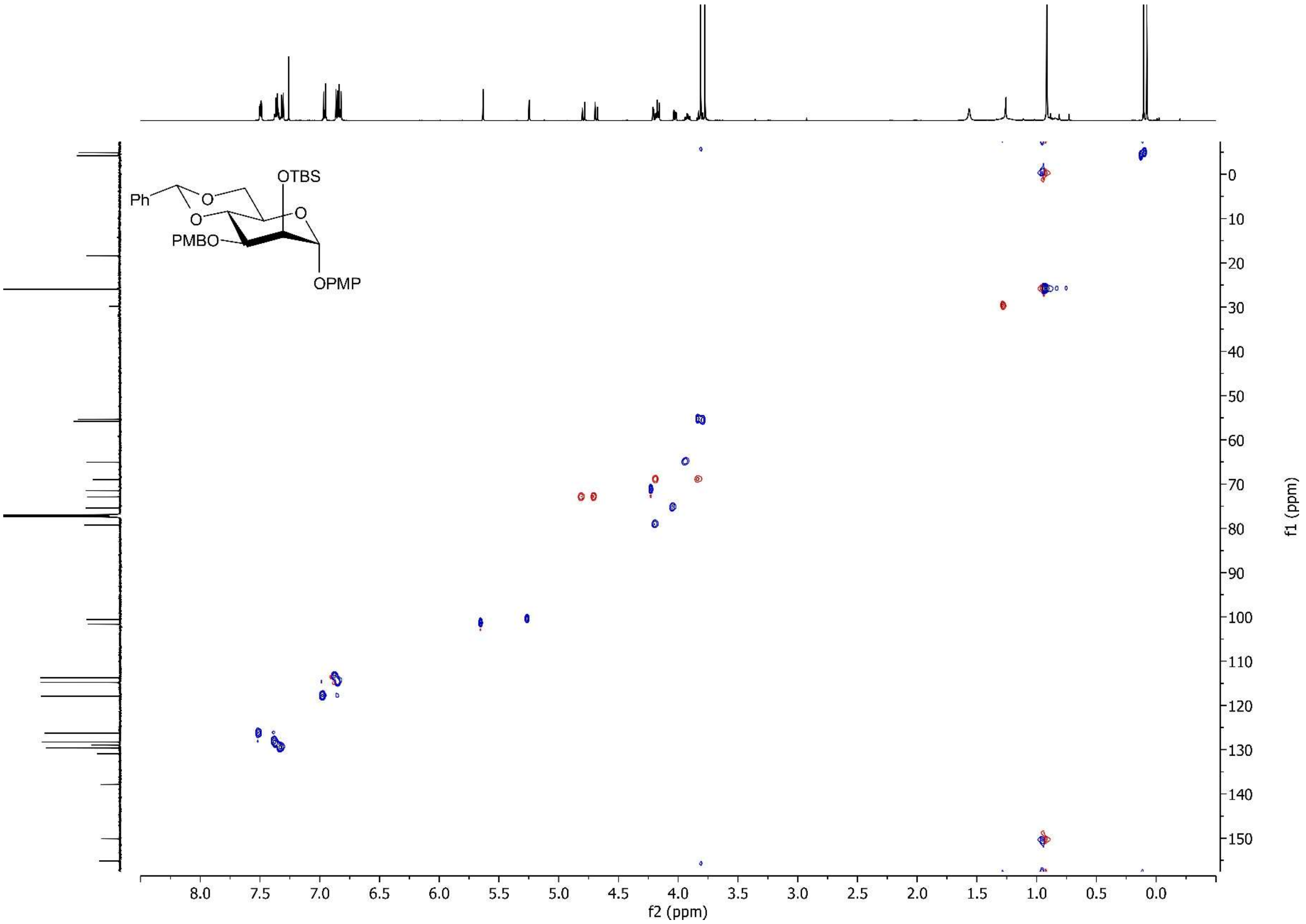

Figure S15. HMBC NMR (600 MHz, $CDCl_3$) spectrum of *p*-methoxyphenyl 4,6-*O*-benzylidene-3-*O*-*p*-methoxybenzyl-2-*O*-*tert*-butyldimethylsilyl-α-D-mannopyranoside **14**:

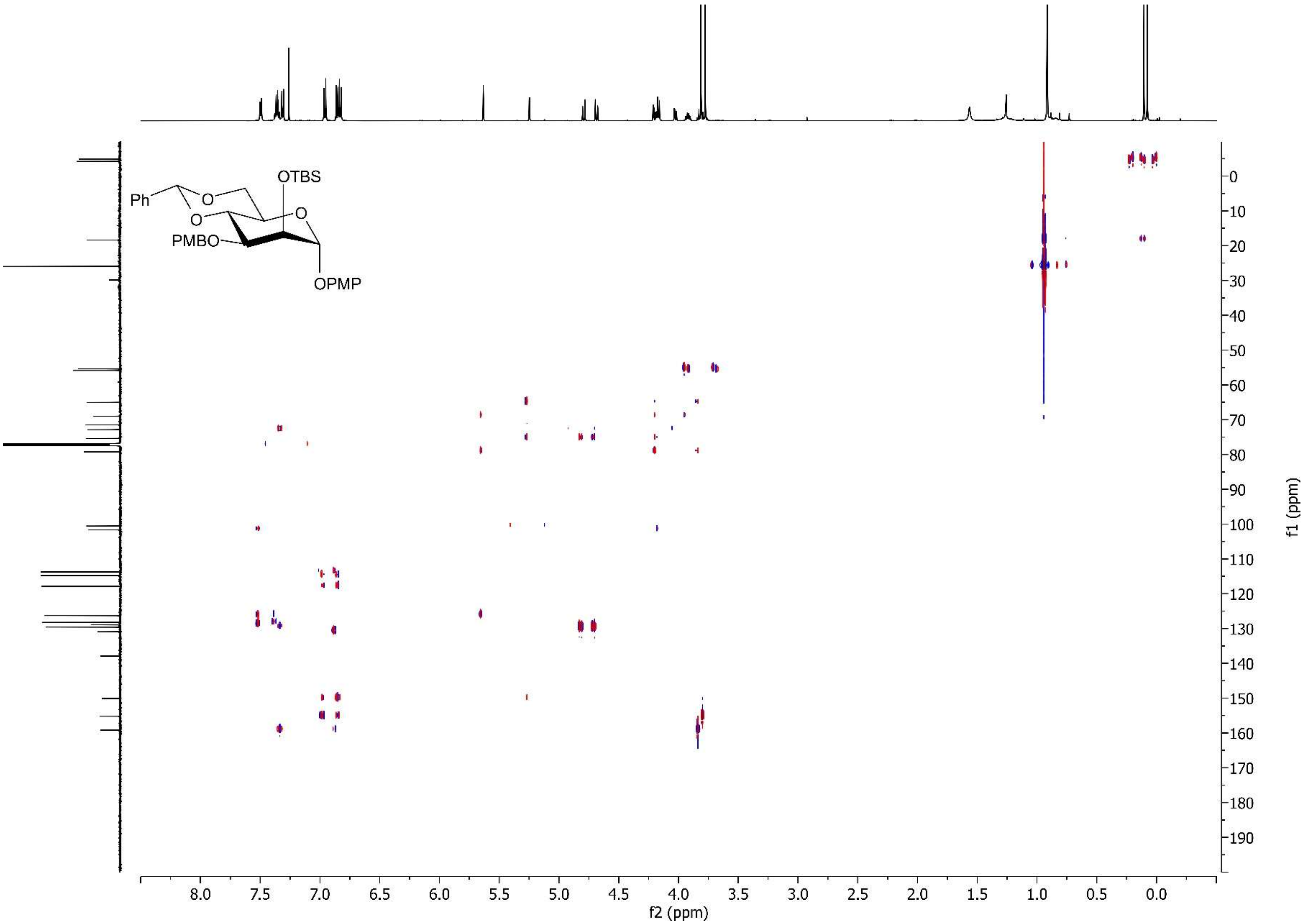

Figure S16. ^{1}H NMR (600 MHz, $CDCl_3$) spectrum of *p*-methoxyphenyl 4,6-*O*-benzylidene-2-*O*-*tert*-butyldimethylsilyl-α-D-mannopyranoside **15**:

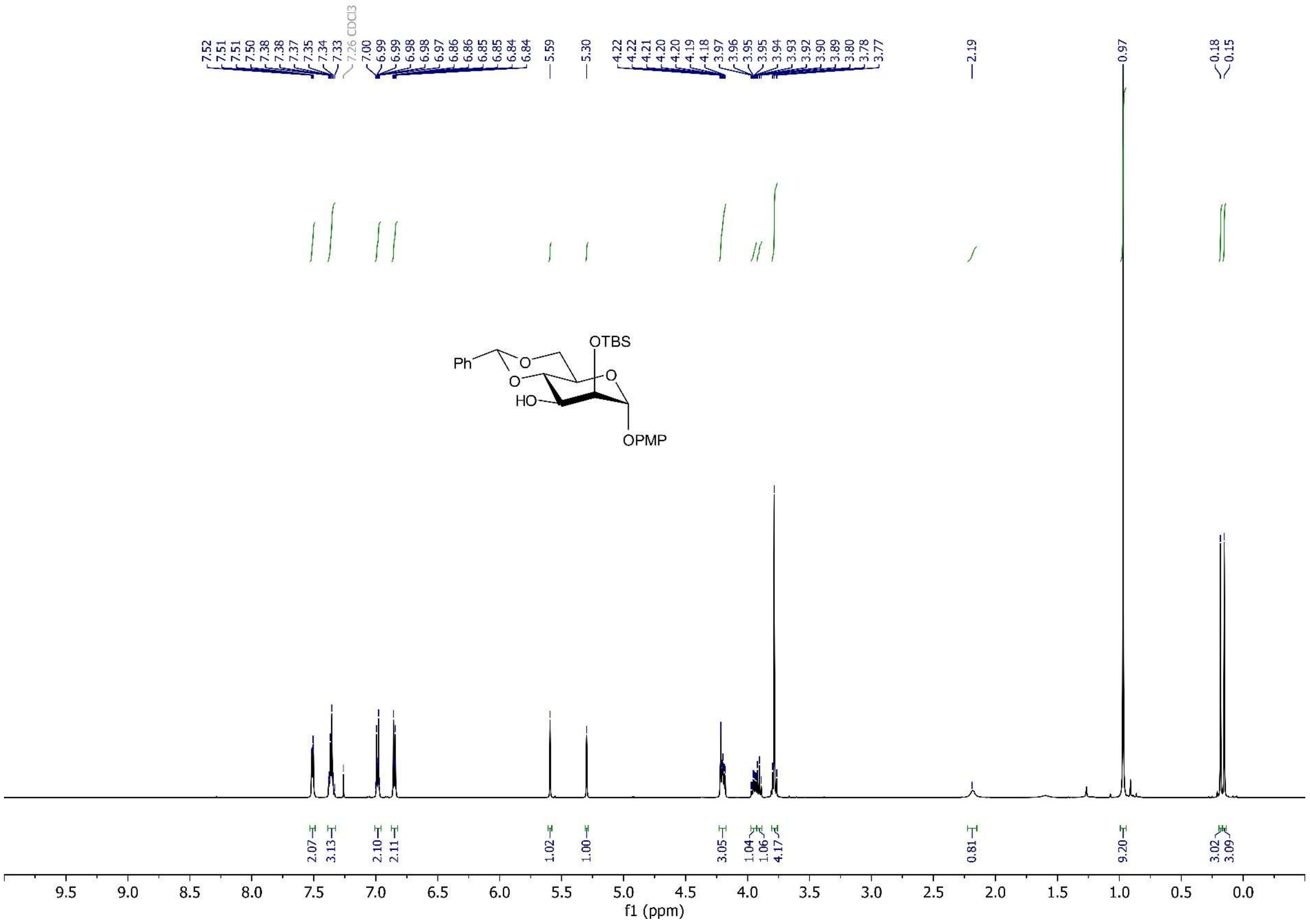

Figure S17. COSY NMR (600 MHz, $CDCl_3$) spectrum of *p*-methoxyphenyl 4,6-*O*-benzylidene-2-*O*-*tert*-butyldimethylsilyl-α-D-mannopyranoside **15**:

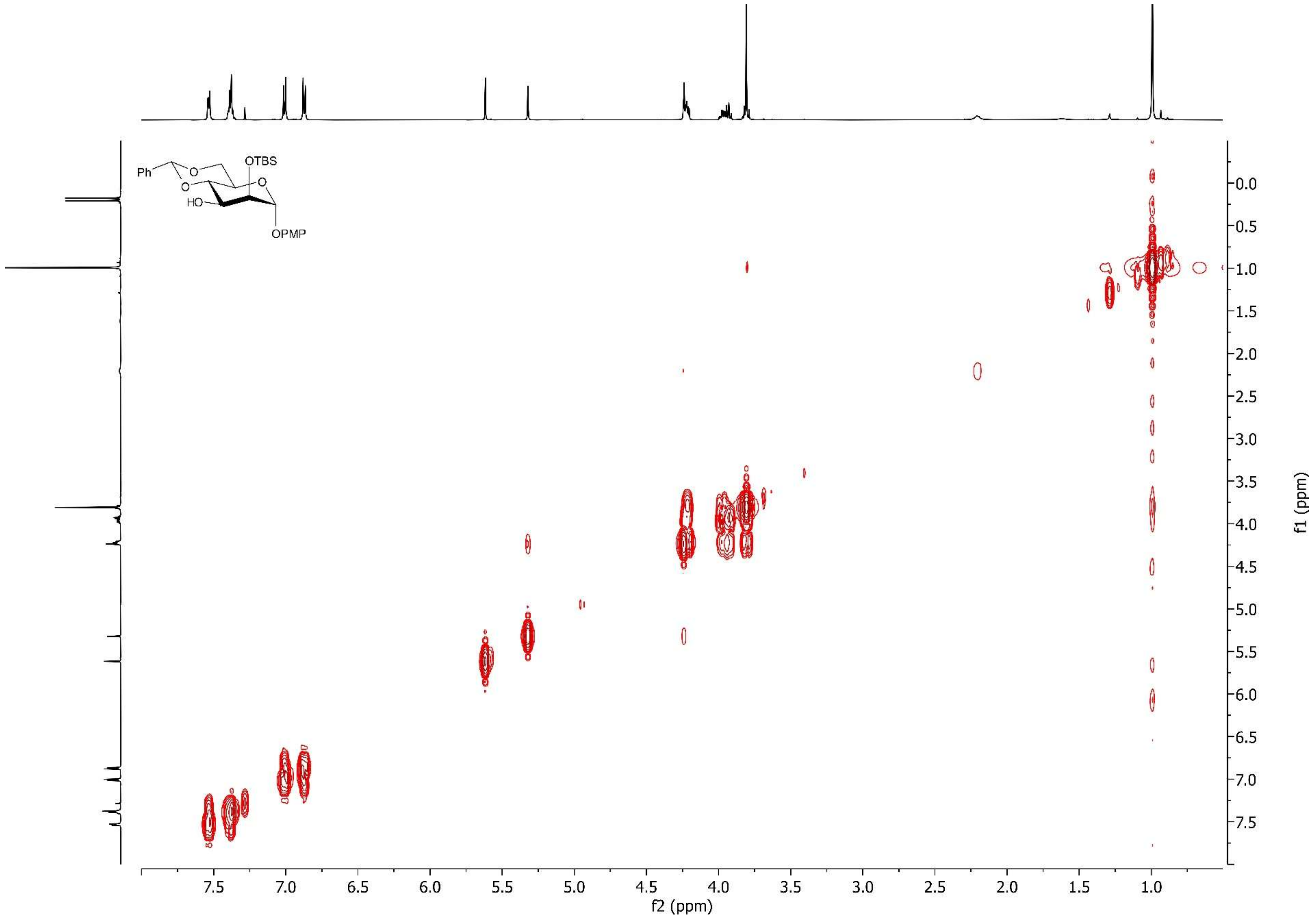

Figure S18. $^{13}C\{H\}$ NMR (151 MHz, $CDCl_3$) spectrum of *p*-methoxyphenyl 4,6-*O*-benzylidene-2-*O*-*tert*-butyldimethylsilyl-α-D-mannopyranoside **15**:

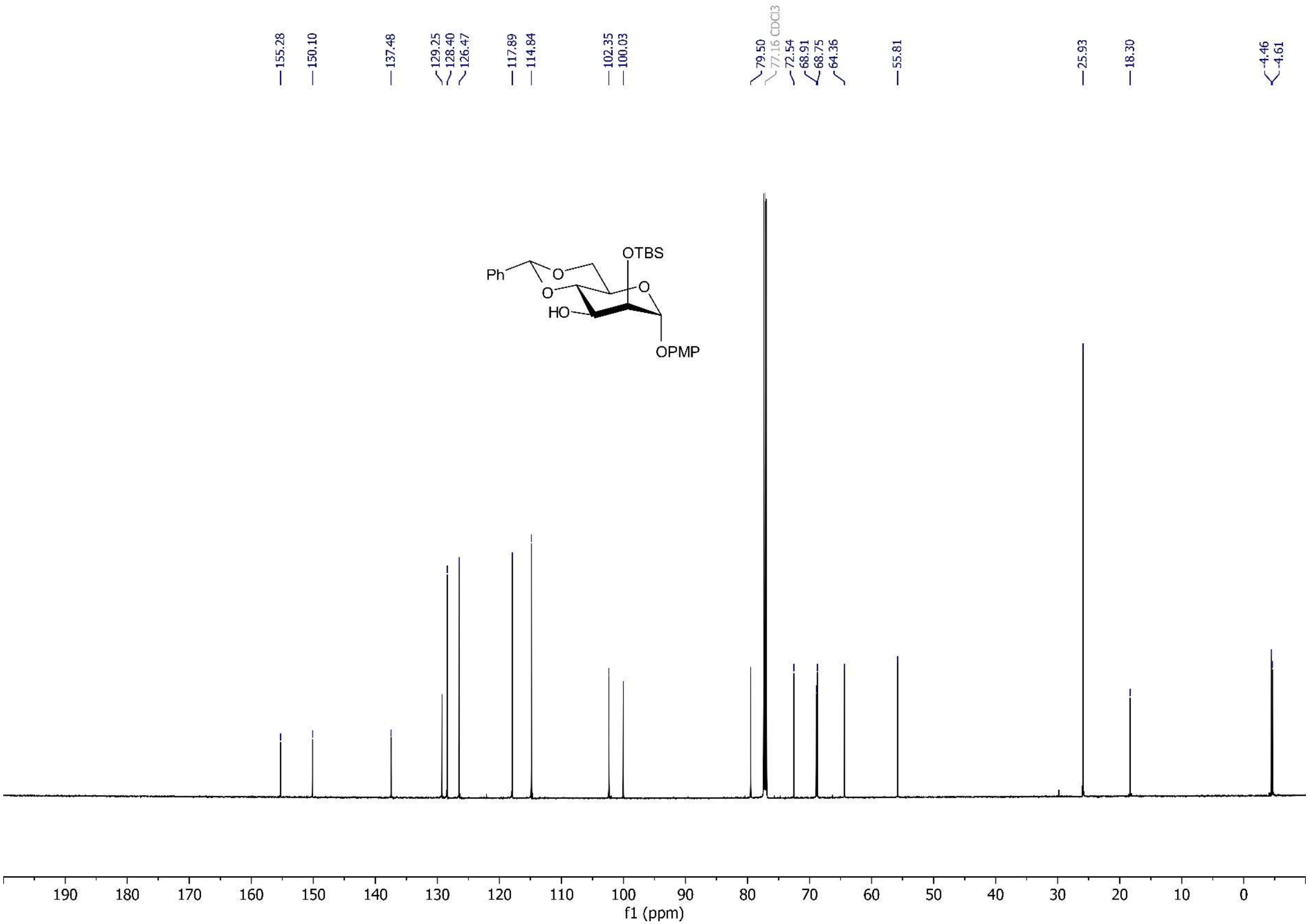

Figure S19. HSQC NMR (600 MHz, $CDCl_3$) spectrum of *p*-methoxyphenyl 4,6-*O*-benzylidene-2-*O*-*tert*-butyldimethylsilyl-α-D-mannopyranoside **15**:

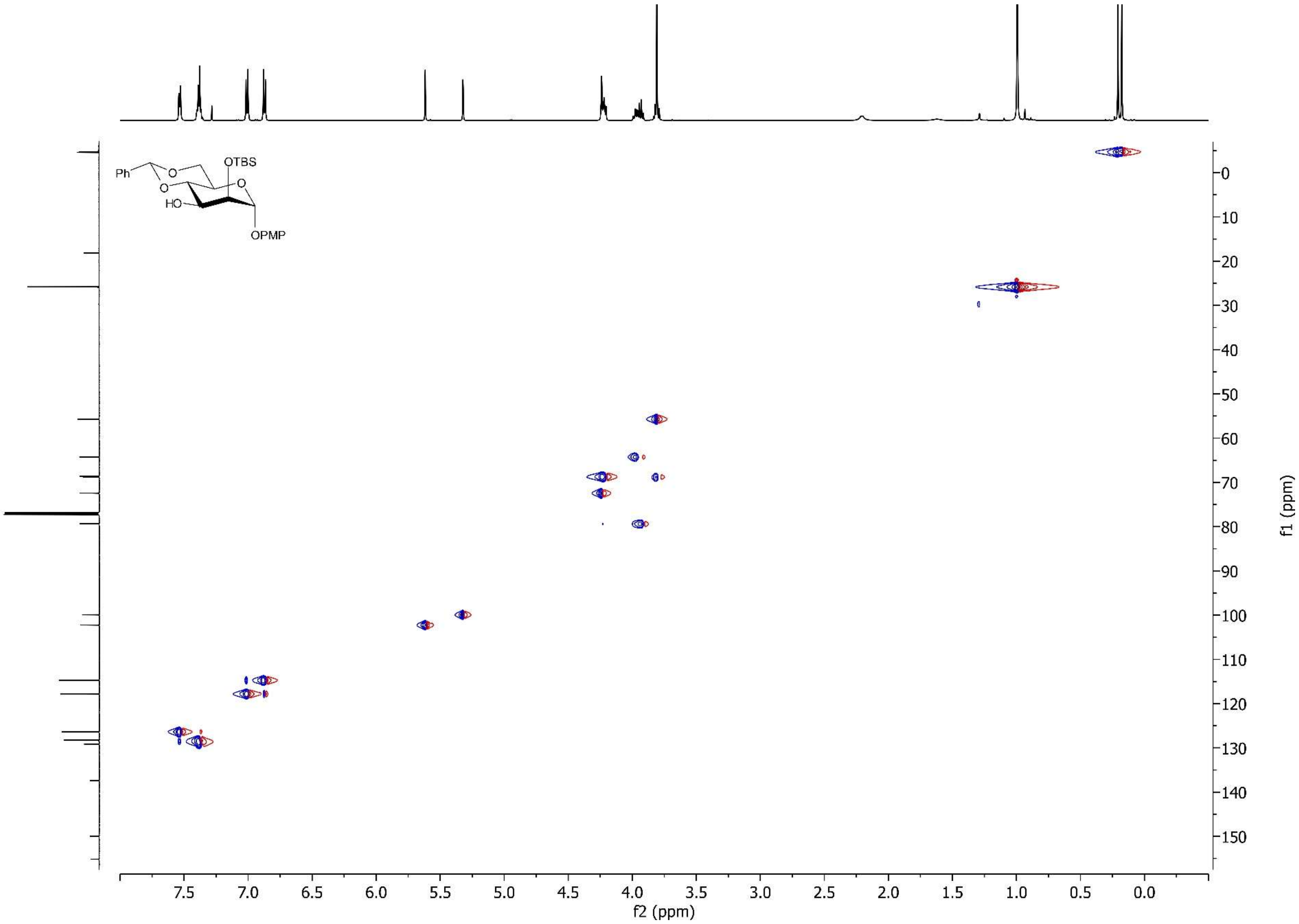

Figure S20. HMBC NMR (600 MHz, $CDCl_3$) spectrum of *p*-methoxyphenyl 4,6-*O*-benzylidene-2-*O*-*tert*-butyldimethylsilyl-α-D-mannopyranoside **15**:

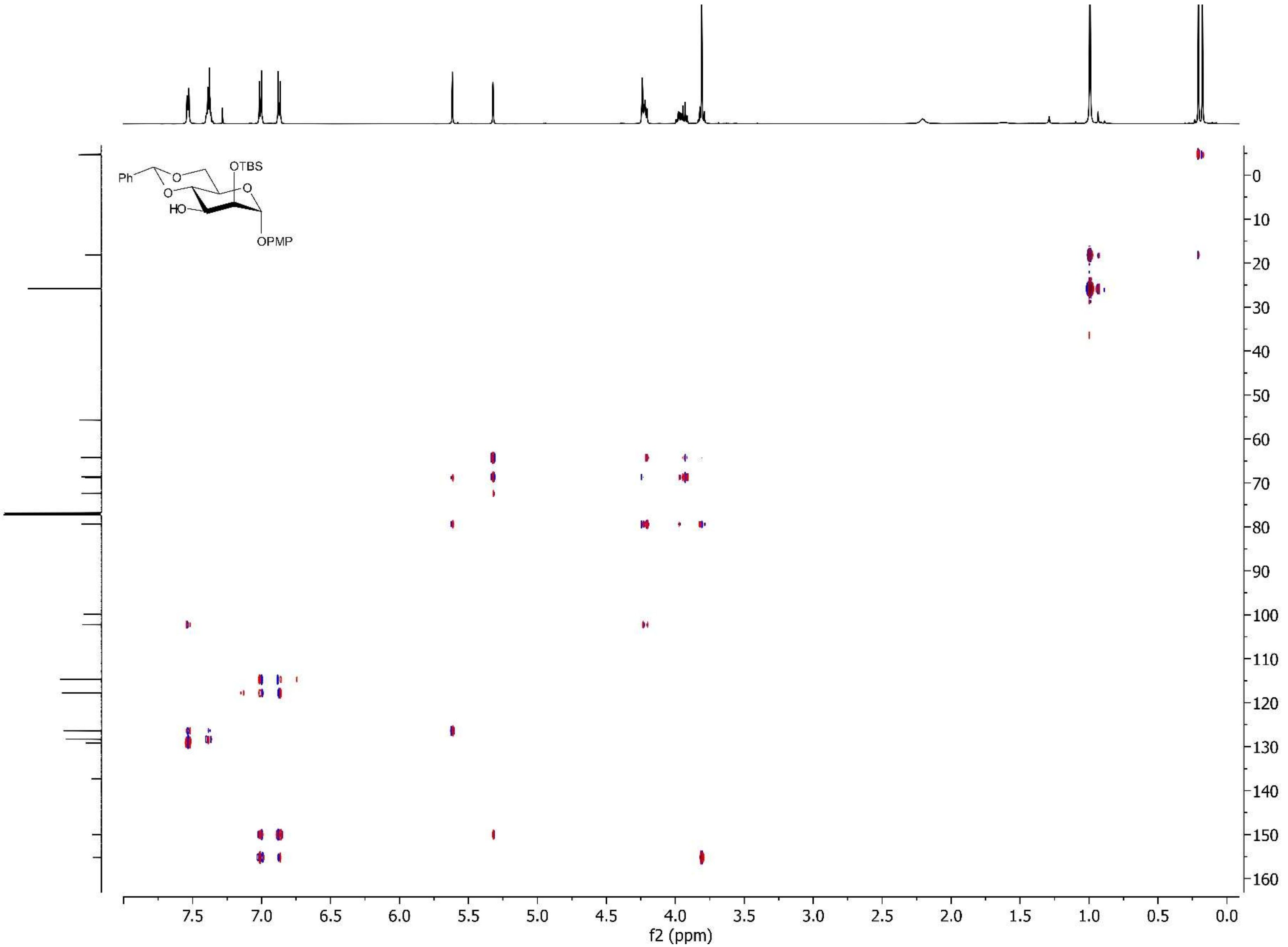

Figure S21. ^{1}H NMR (600 MHz, $CDCl_3$) spectrum of *p*-methoxyphenyl 4,6-*O*-benzylidene-2-*O*-*tert*-butyldimethylsilyl-α-D-*arabino*-hexopyranosid-3-ulose **16**:

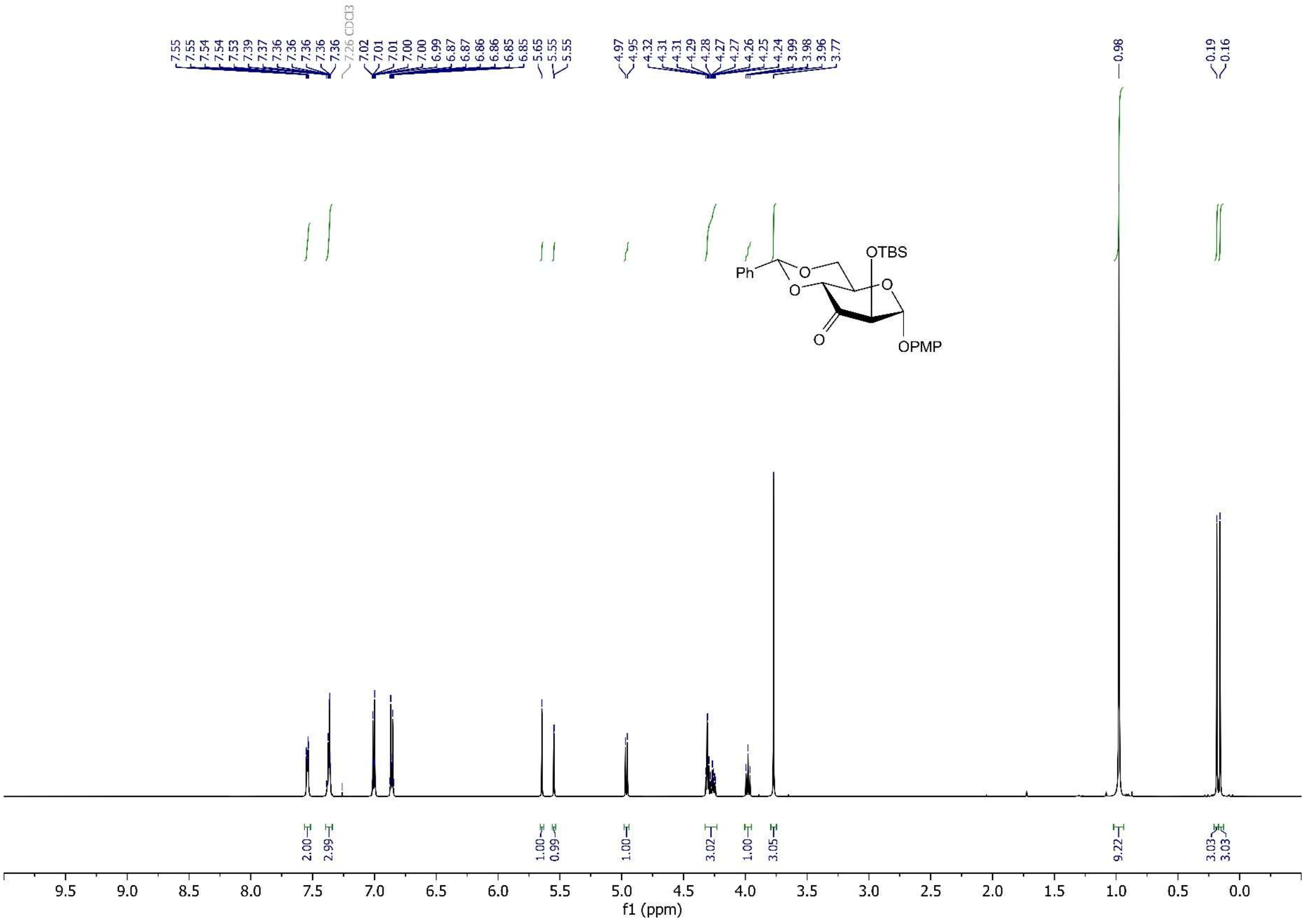

Figure S22. COSY NMR (600 MHz, $CDCl_3$) spectrum of *p*-methoxyphenyl 4,6-*O*-benzylidene-2-*O*-*tert*-butyldimethylsilyl-α-D-*arabino*-hexopyranosid-3-ulose **16**:

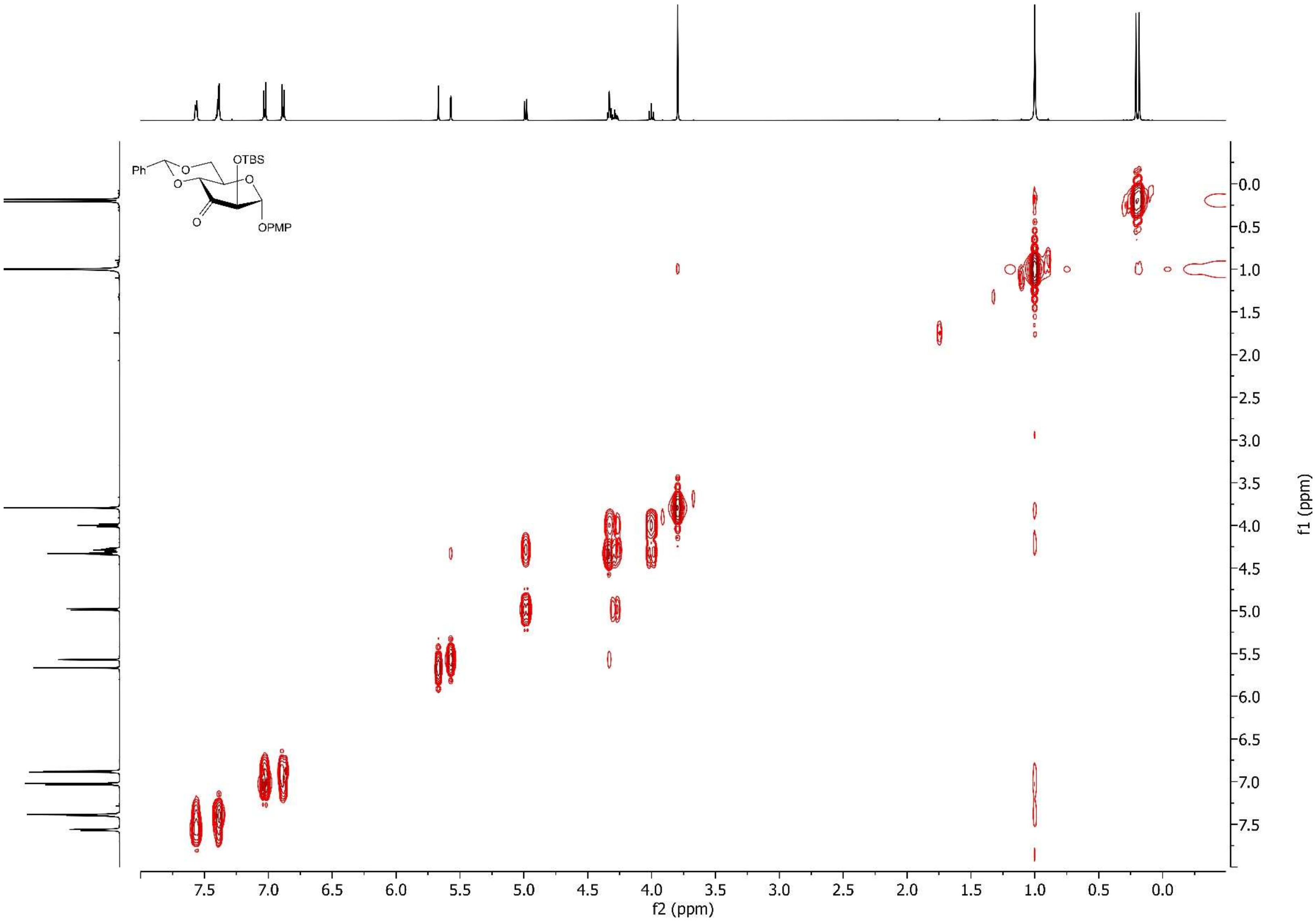

Figure S23. $^{13}C\{H\}$ NMR (151 MHz, $CDCl_3$) spectrum of *p*-methoxyphenyl 4,6-*O*-benzylidene-2-*O*-*tert*-butyldimethylsilyl-α-D-*arabino*-hexopyranosid-3-ulose **16**:

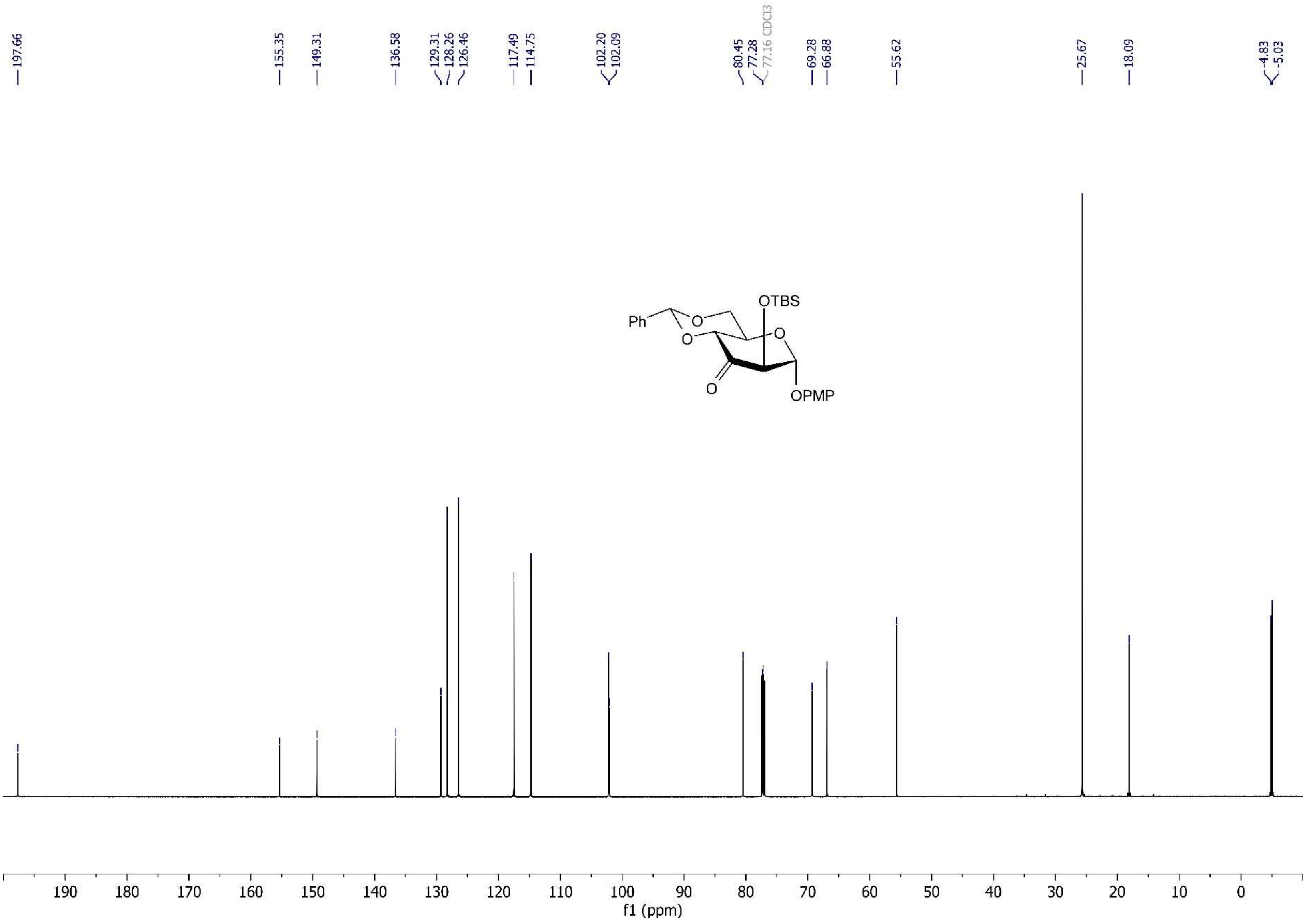

Figure S24. HSQC NMR (600 MHz, $CDCl_3$) spectrum of *p*-methoxyphenyl 4,6-*O*-benzylidene-2-*O*-*tert*-butyldimethylsilyl-α-D-*arabino*-hexopyranosid-3-ulose **16**:

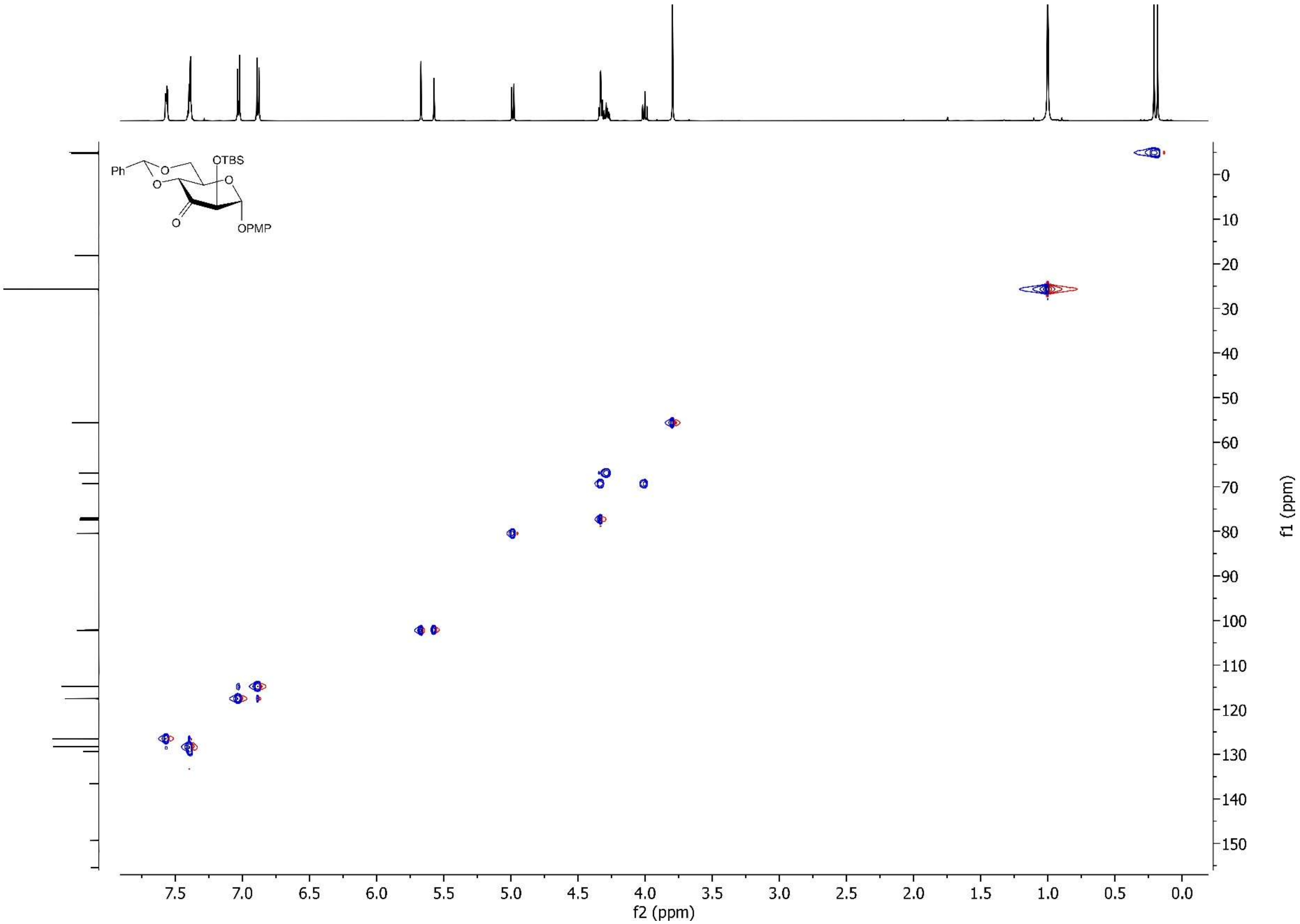

Figure S25. HMBC NMR (600 MHz, $CDCl_3$) spectrum of *p*-methoxyphenyl 4,6-*O*-benzylidene-2-*O*-*tert*-butyldimethylsilyl-α-D-*arabino*-hexopyranosid-3-ulose **16**:

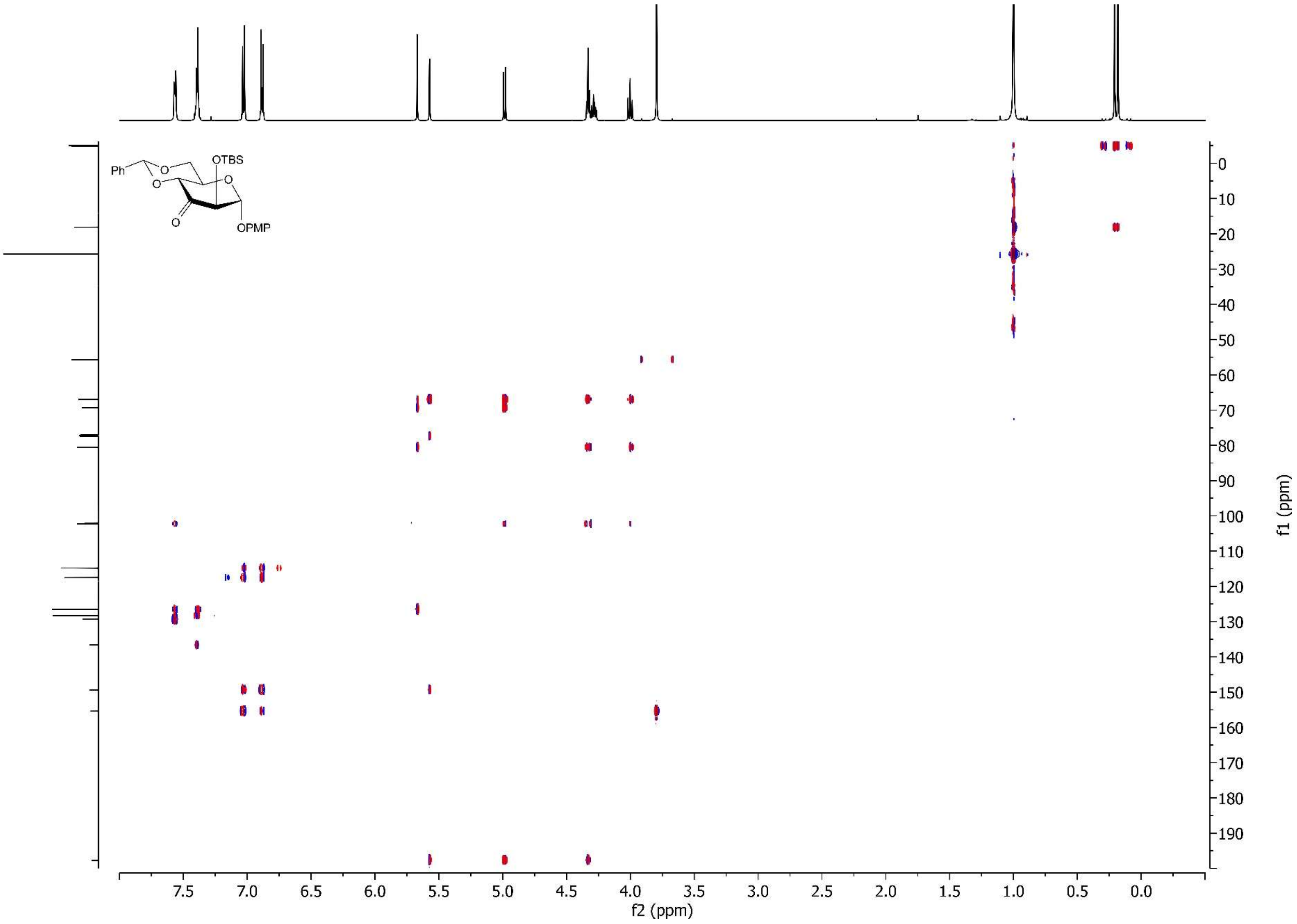

Figure S26. ^{1}H NMR (600 MHz, $CDCl_3$) spectrum of *p*-methoxyphenyl 4,6-*O*-benzylidene-2-*O*-*tert*-butyldimethylsilyl-3-*C*-methyl-α-D-altropyranoside **17**:

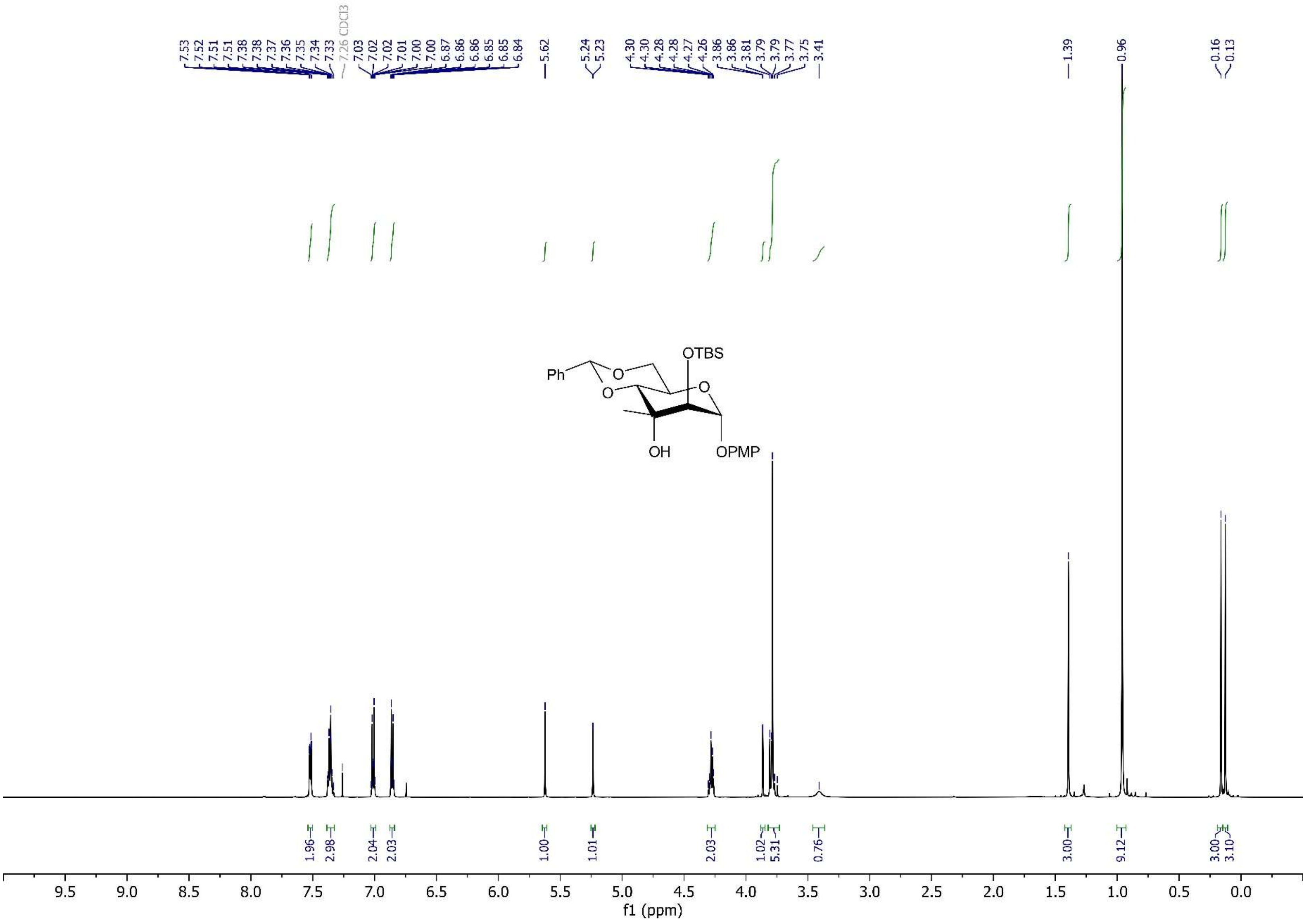

Figure S27. COSY NMR (600 MHz, $CDCl_3$) spectrum of *p*-methoxyphenyl 4,6-*O*-benzylidene-2-*O*-*tert*-butyldimethylsilyl-3-*C*-methyl-α-D-altropyranoside **17**:

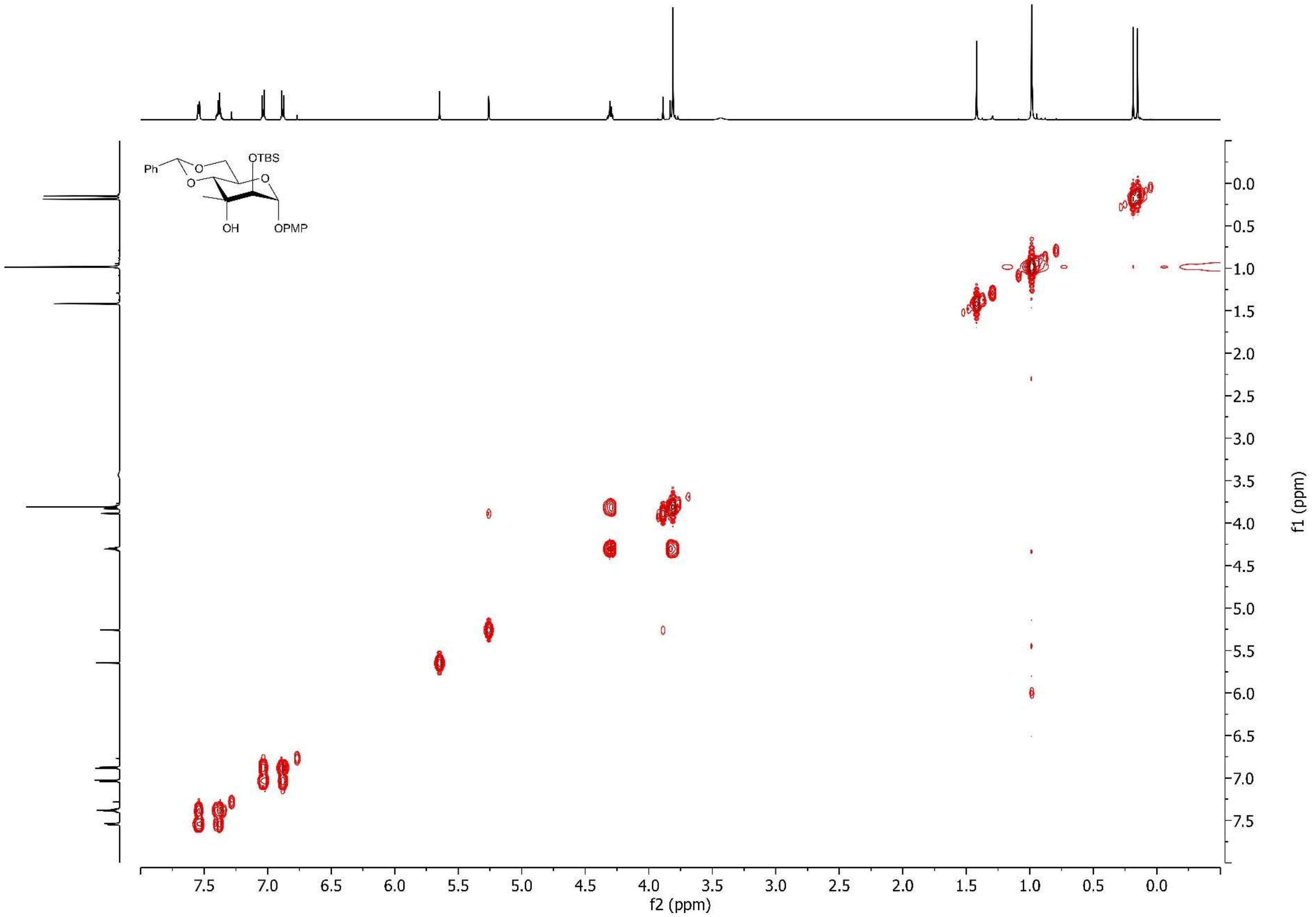

Figure S28. ^{1}H (NOE) NMR (600 MHz, $CDCl_3$) spectrum of *p*-methoxyphenyl 4,6-*O*-benzylidene-2-*O*-*tert*-butyldimethylsilyl-3-*C*-methyl-α-D-altropyranoside **17**:

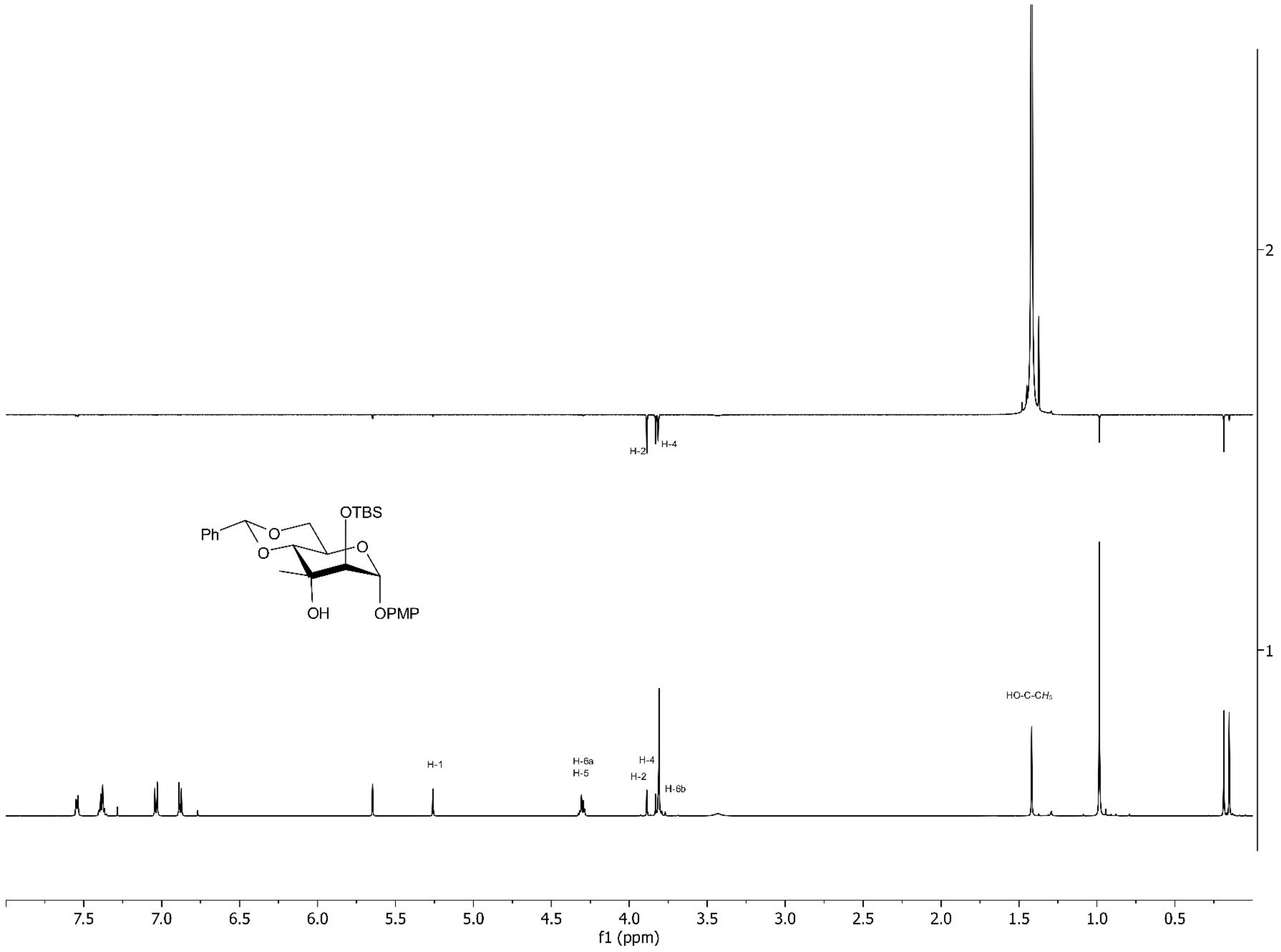

Figure S29. $^{13}C\{H\}$ NMR (151 MHz, $CDCl_3$) spectrum of *p*-methoxyphenyl 4,6-*O*-benzylidene-2-*O*-*tert*-butyldimethylsilyl-3-*C*-methyl-α-D-altropyranoside **17**:

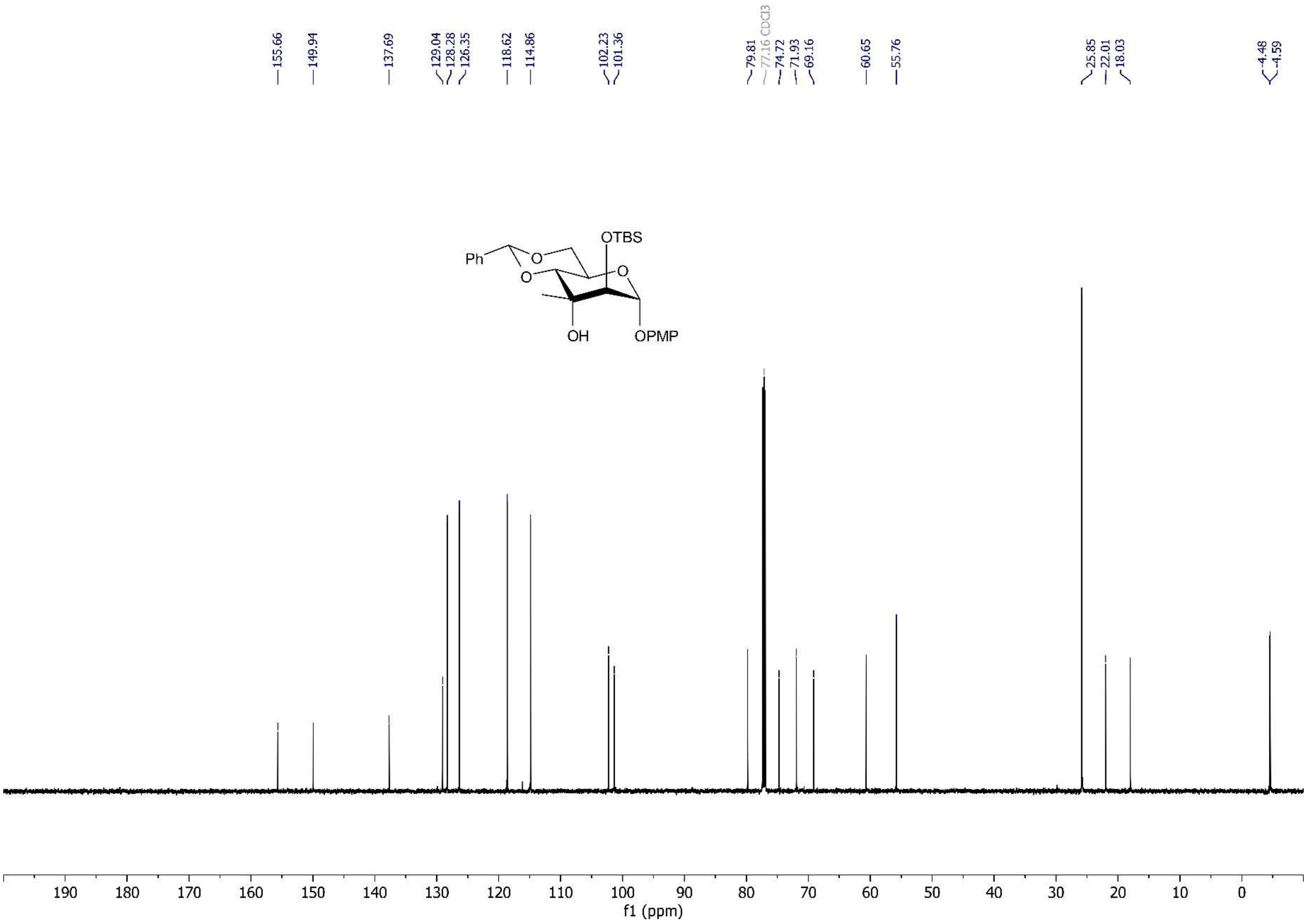

Figure S30. HSQC NMR (600 MHz, $CDCl_3$) spectrum of *p*-methoxyphenyl 4,6-*O*-benzylidene-2-*O*-*tert*-butyldimethylsilyl-3-*C*-methyl-α-D-altropyranoside **17**:

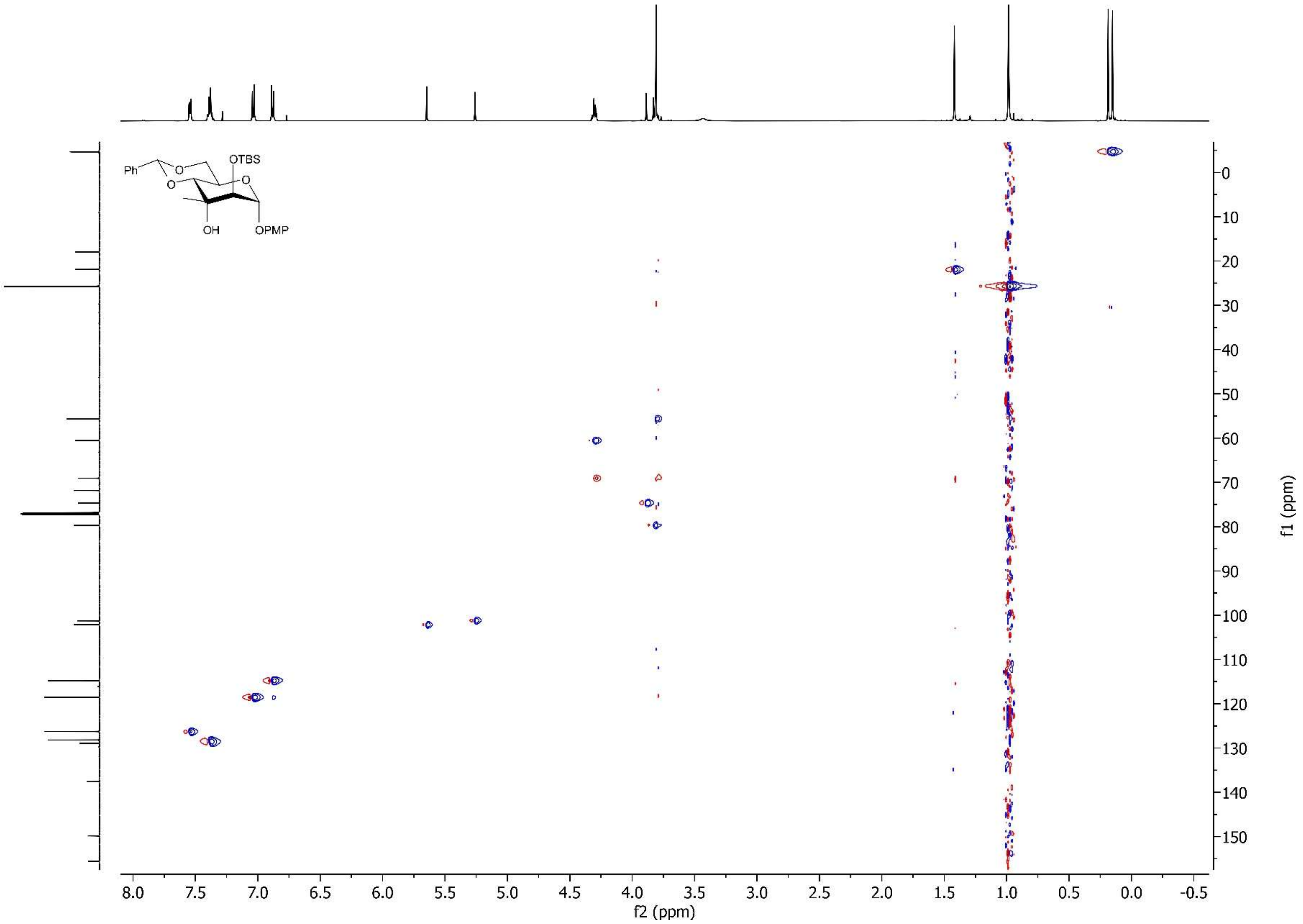

Figure S31. HMBC NMR (600 MHz, $CDCl_3$) spectrum of *p*-methoxyphenyl 4,6-*O*-benzylidene-2-*O*-*tert*-butyldimethylsilyl-3-*C*-methyl-α-D-altropyranoside **17**:

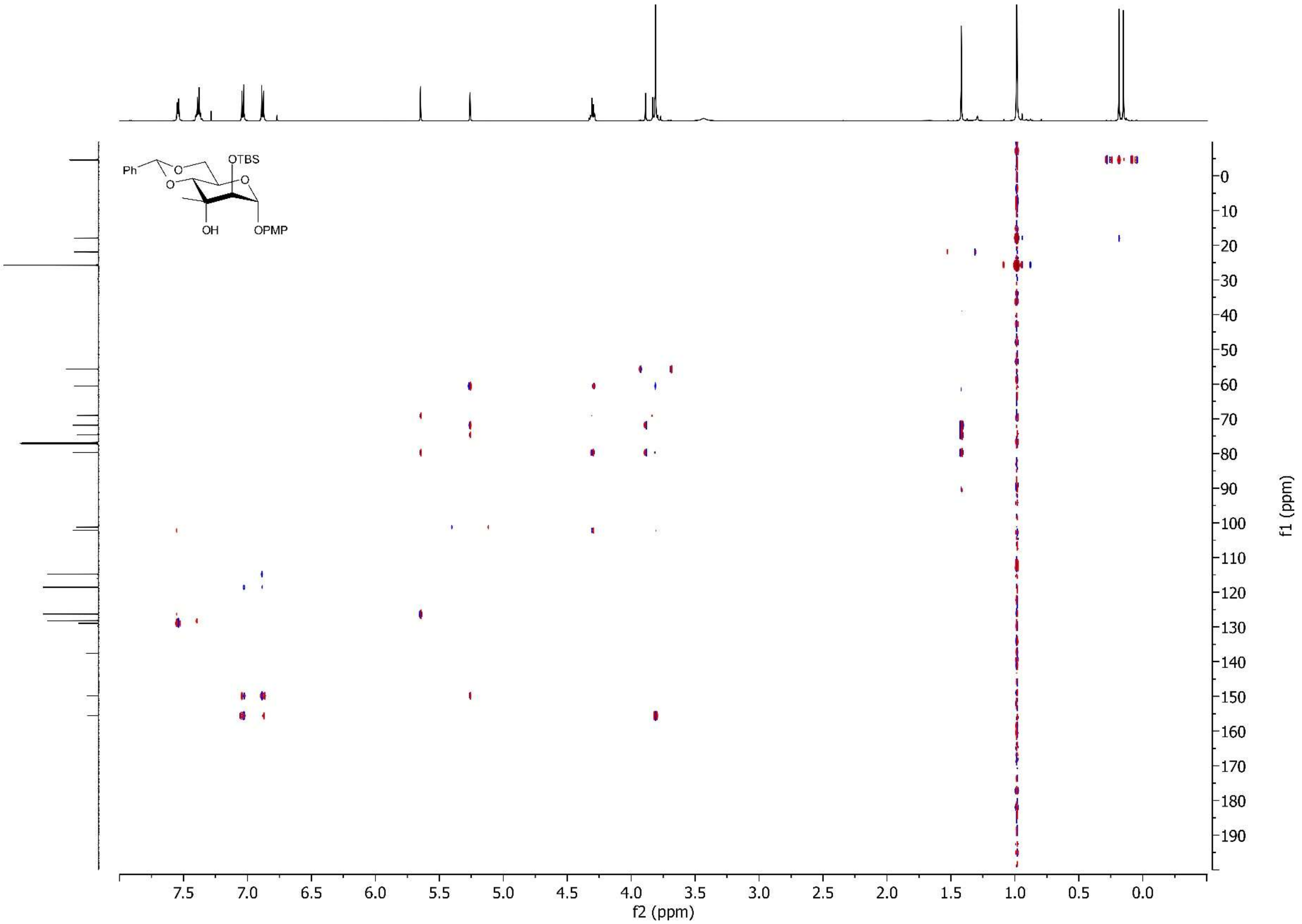

Figure S32. ^{1}H NMR (600 MHz, $CDCl_3$) spectrum of *p*-methoxyphenyl 4,6-*O*-benzylidene-2-*O*-*tert*-butyldimethylsilyl-3-deoxy-3-methylene-α-D-*arabino*-hexopyranoside **18**:

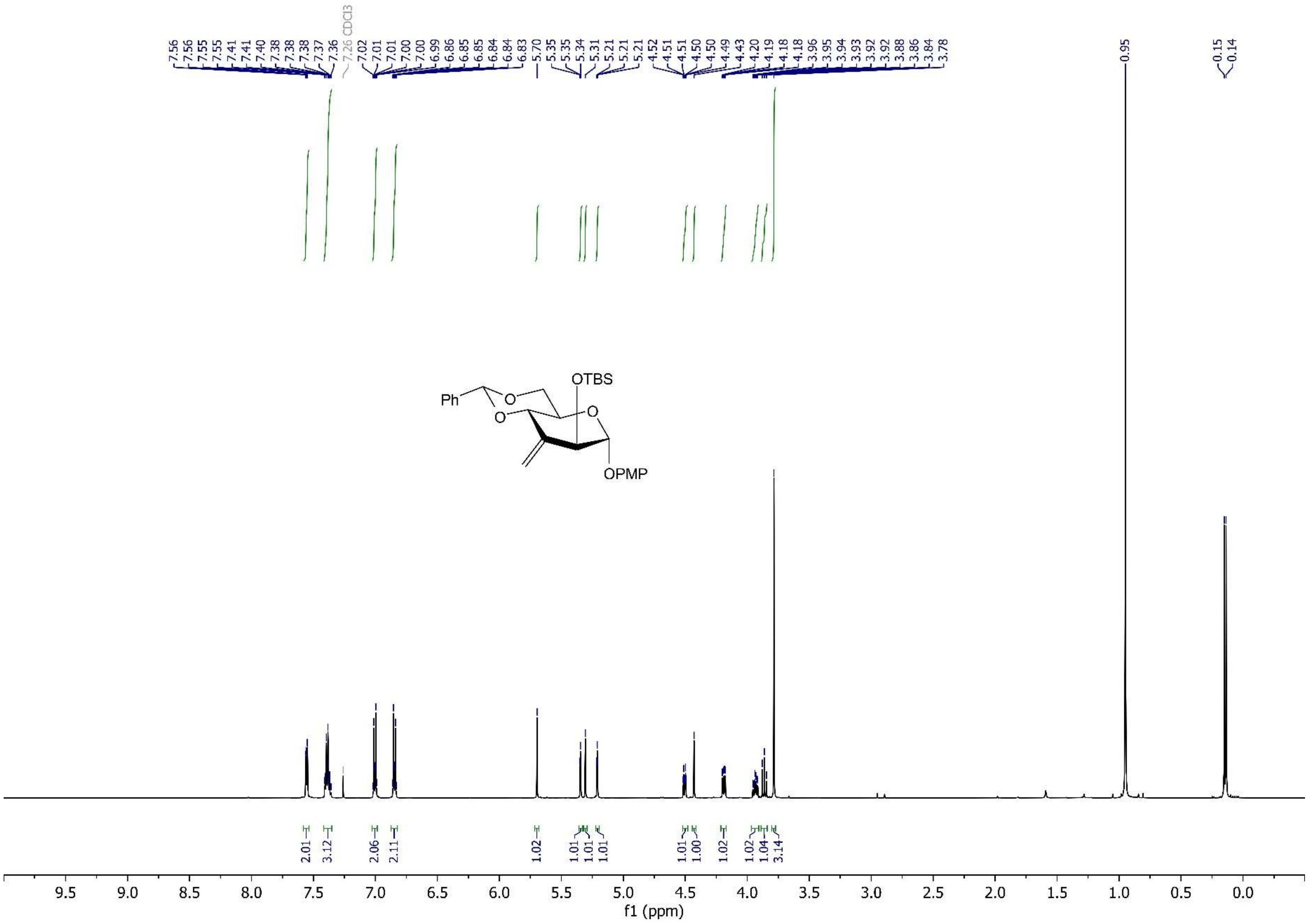

Figure S33. COSY NMR (600 MHz, $CDCl_3$) spectrum of *p*-methoxyphenyl 4,6-*O*-benzylidene-2-*O*-*tert*-butyldimethylsilyl-3-deoxy-3-methylene-α-D-*arabino*-hexopyranoside **18**:

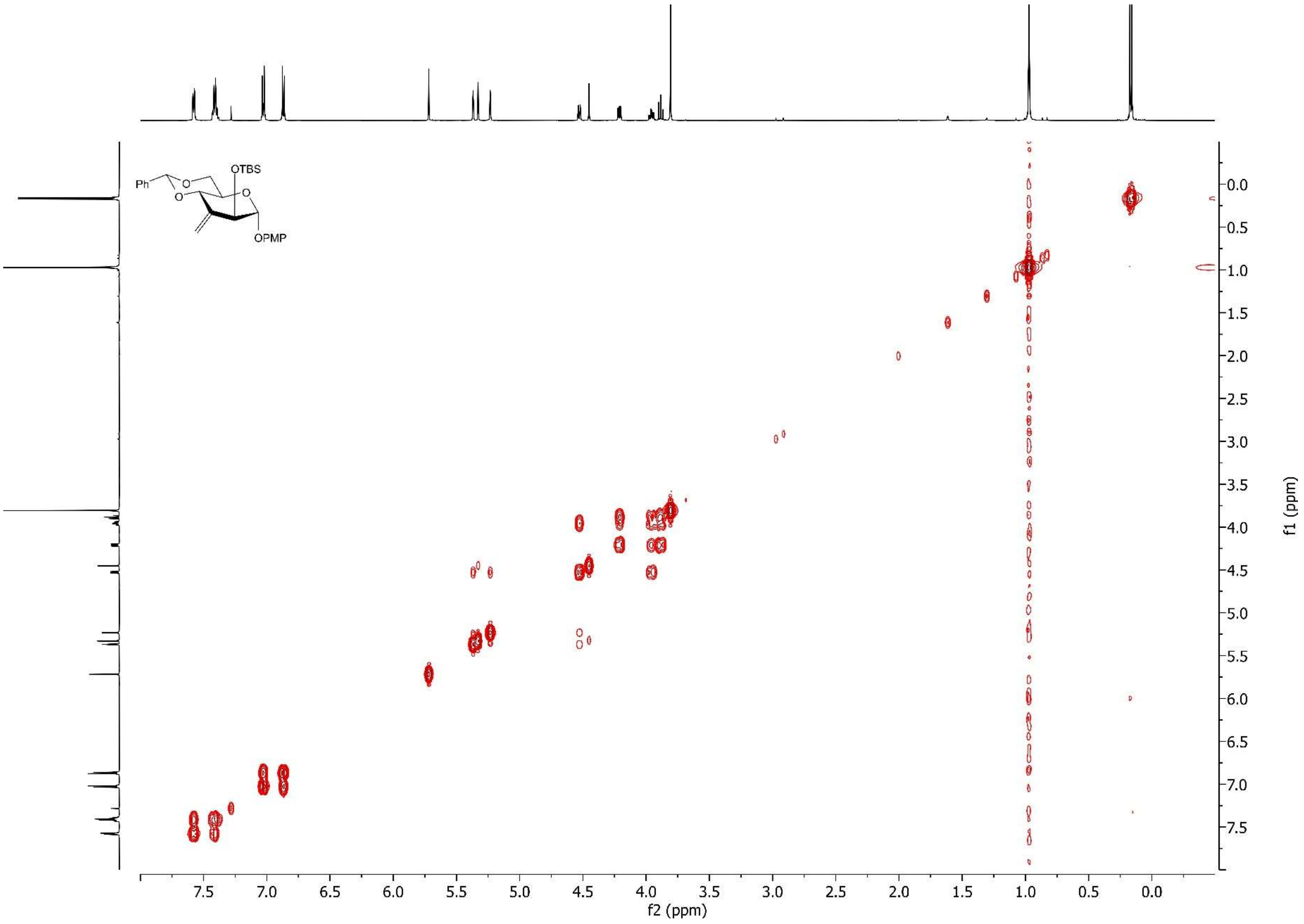

Figure S34. ^{13}C{H} NMR (151 MHz, $CDCl_3$) spectrum of *p*-methoxyphenyl 4,6-*O*-benzylidene-2-*O*-*tert*-butyldimethylsilyl-3-deoxy-3-methylene-α-D-*arabino*-hexopyranoside **18**:

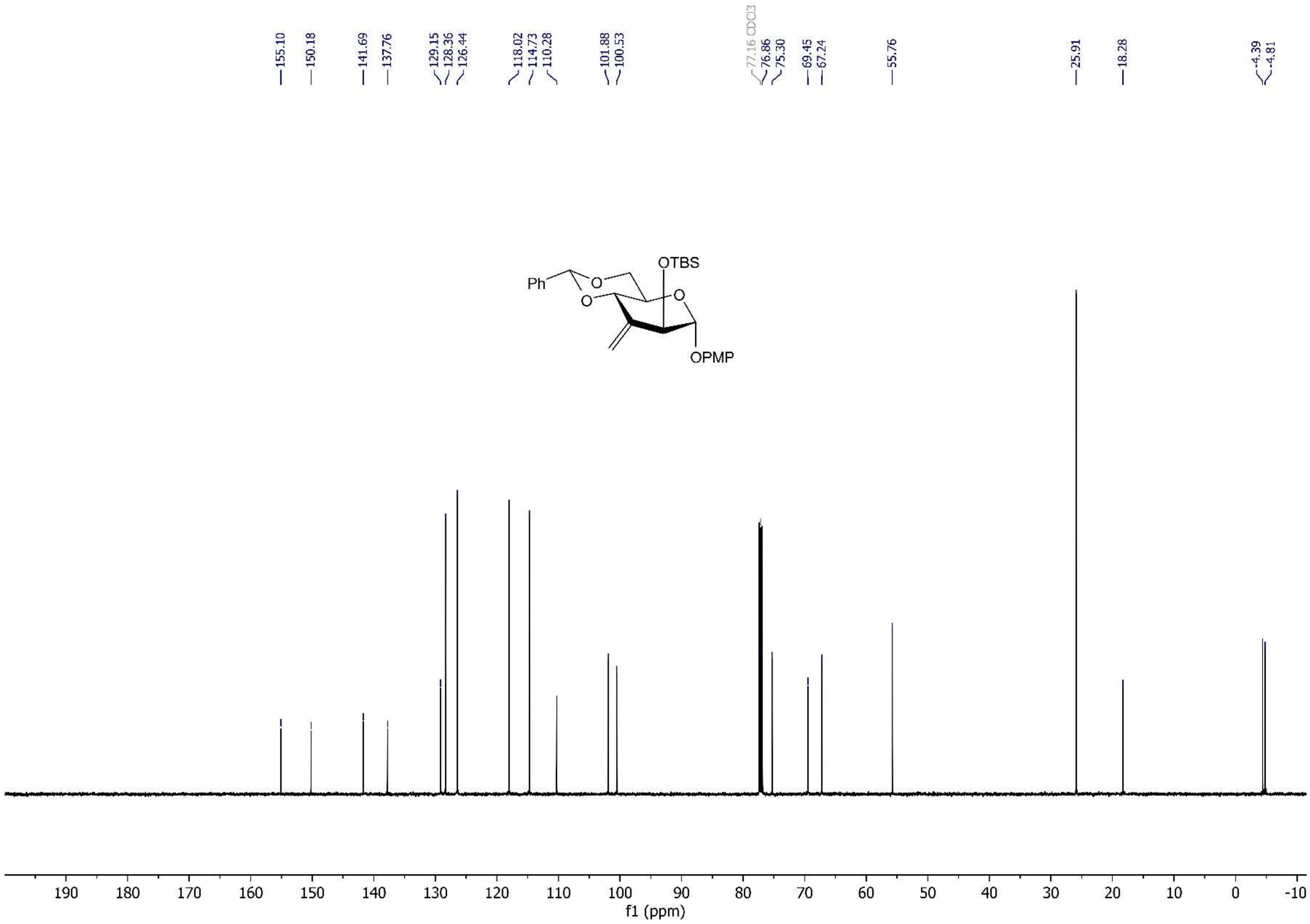

Figure S35. HSQC NMR (600 MHz, $CDCl_3$) spectrum of *p*-methoxyphenyl 4,6-*O*-benzylidene-2-*O*-*tert*-butyldimethylsilyl-3-deoxy-3-methylene-α-D-*arabino*-hexopyranoside **18**:

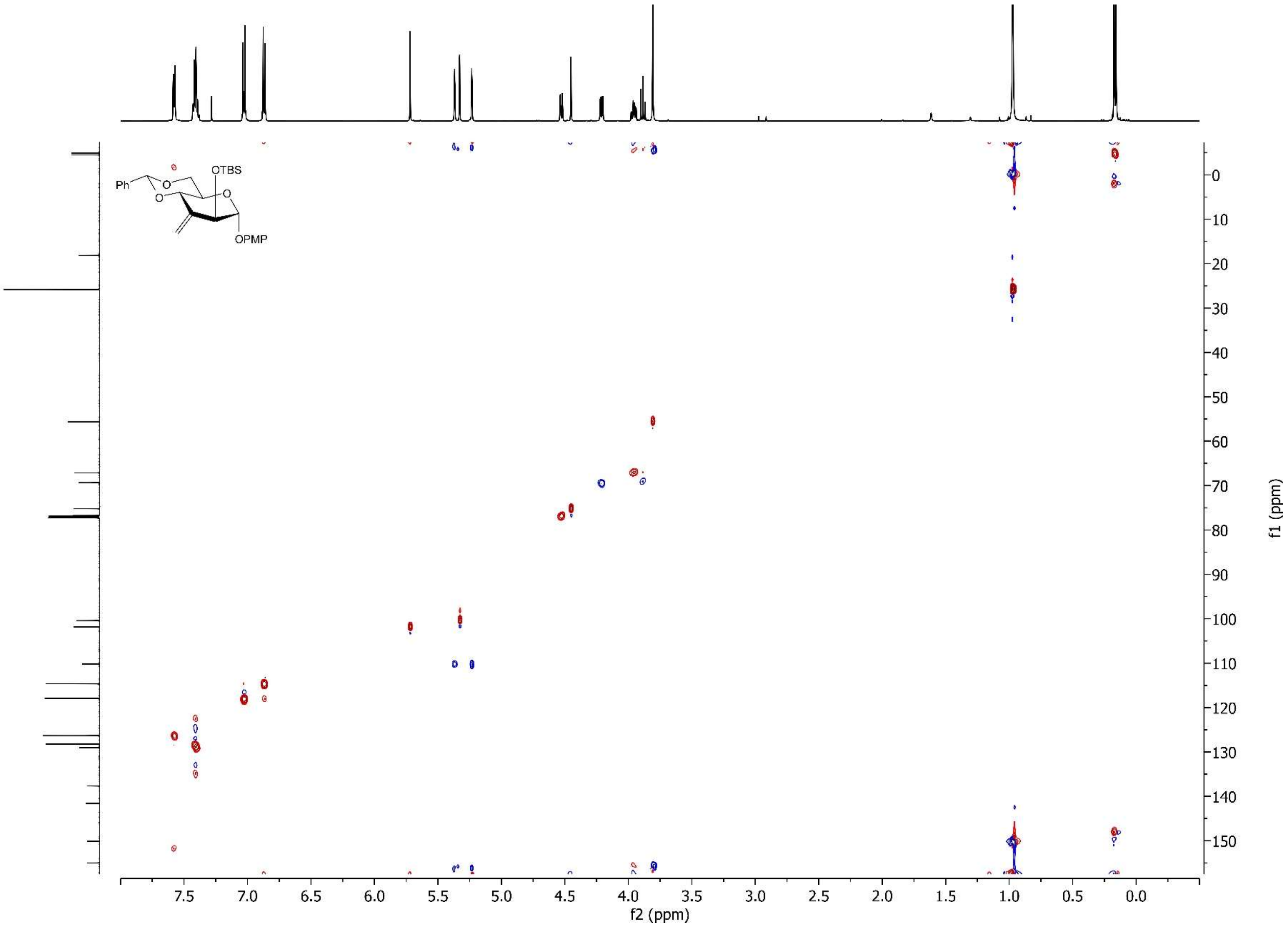

Figure S36. HMBC NMR (600 MHz, $CDCl_3$) spectrum of *p*-methoxyphenyl 4,6-*O*-benzylidene-2-*O*-*tert*-butyldimethylsilyl-3-deoxy-3-methylene-α-D-*arabino*-hexopyranoside **18**:

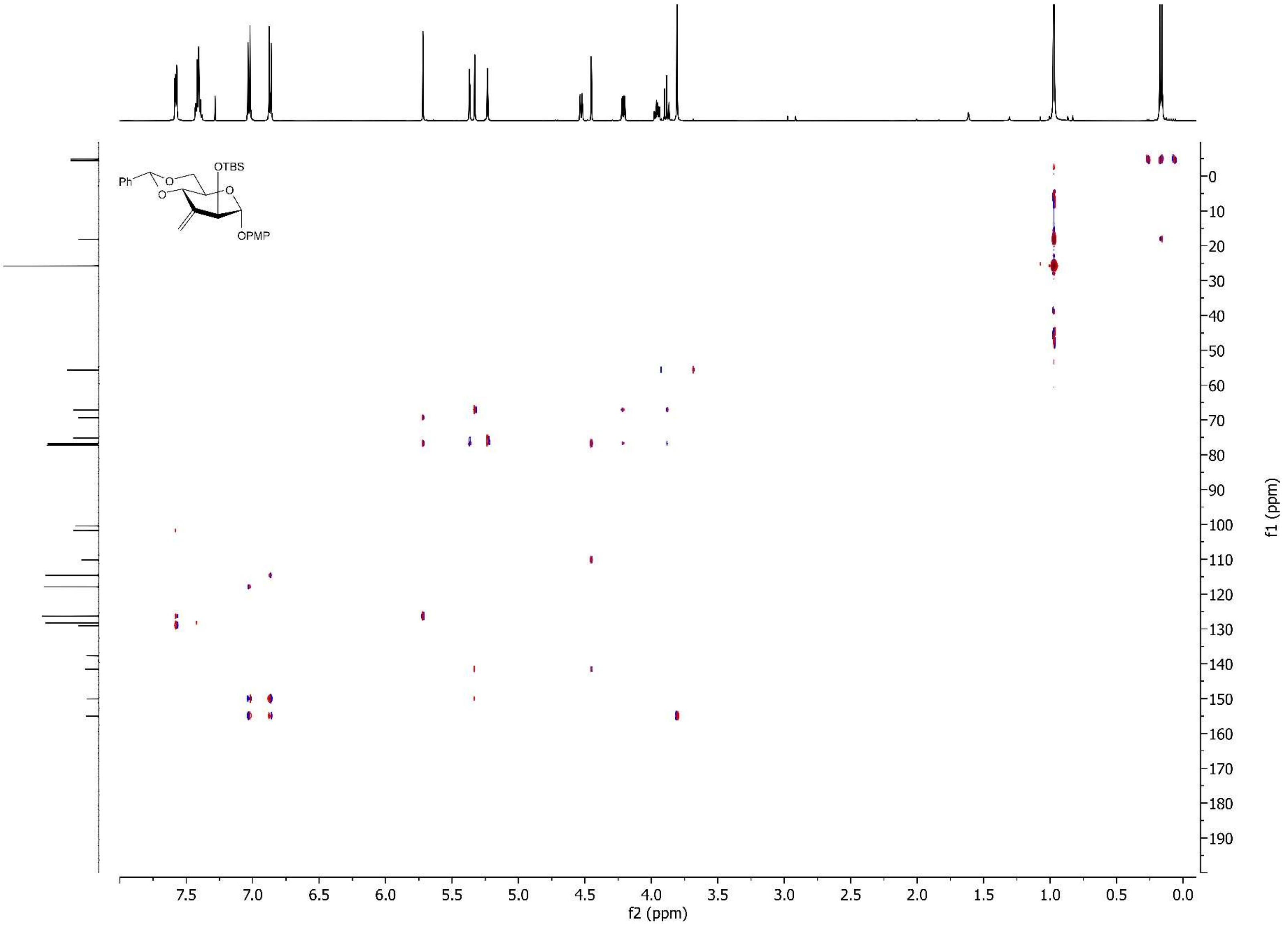

Figure S37. ^{1}H NMR (600 MHz, $CDCl_3$) spectrum of *p*-methoxyphenyl 4,6-*O*-benzylidene-3-deoxy-3-methylene-α-D-*arabino*-hexopyranoside **19**:

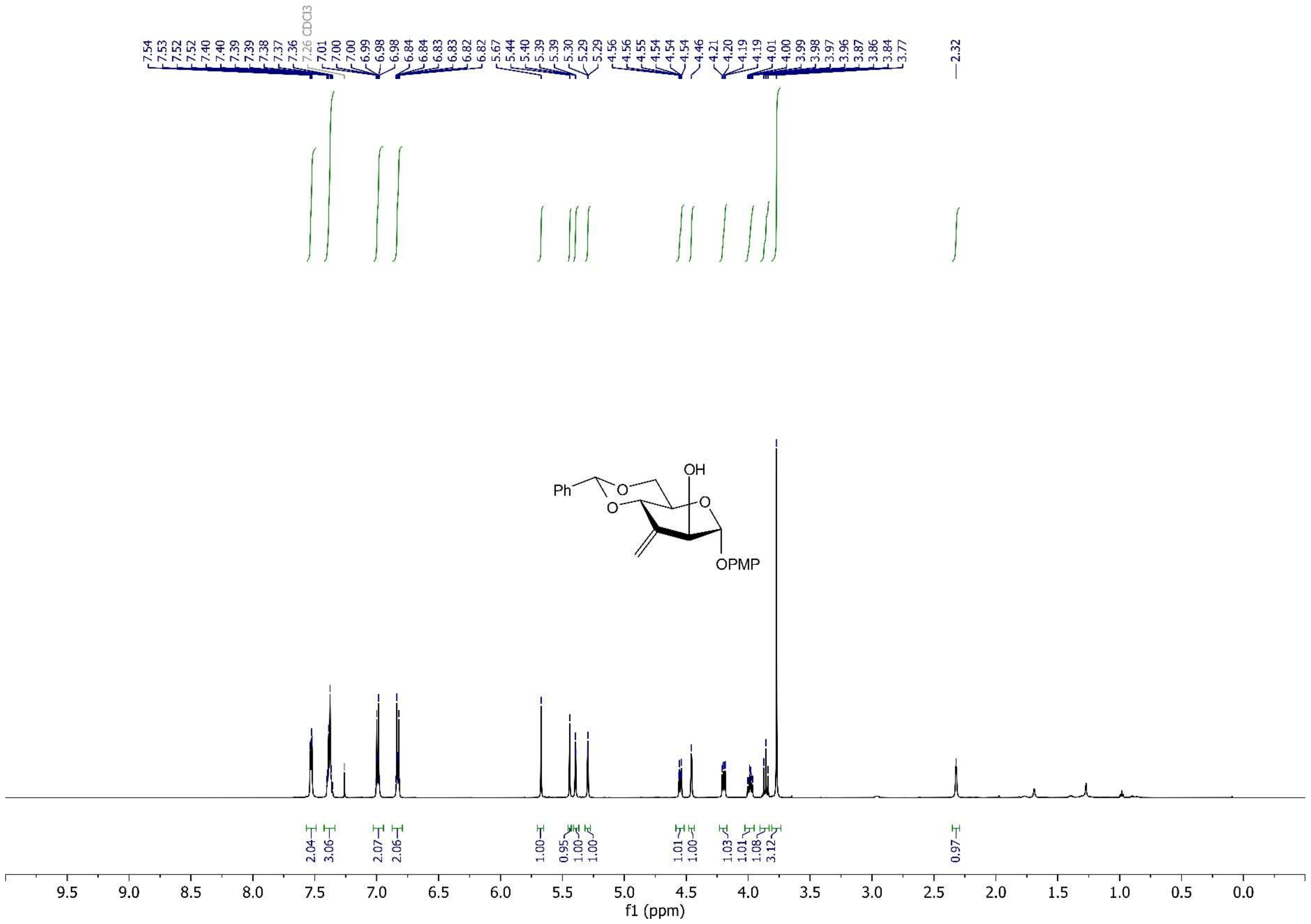

Figure S38. COSY NMR (600 MHz, $CDCl_3$) spectrum of *p*-methoxyphenyl 4,6-*O*-benzylidene-3-deoxy-3-methylene-α-D-*arabino*-hexopyranoside **19:**

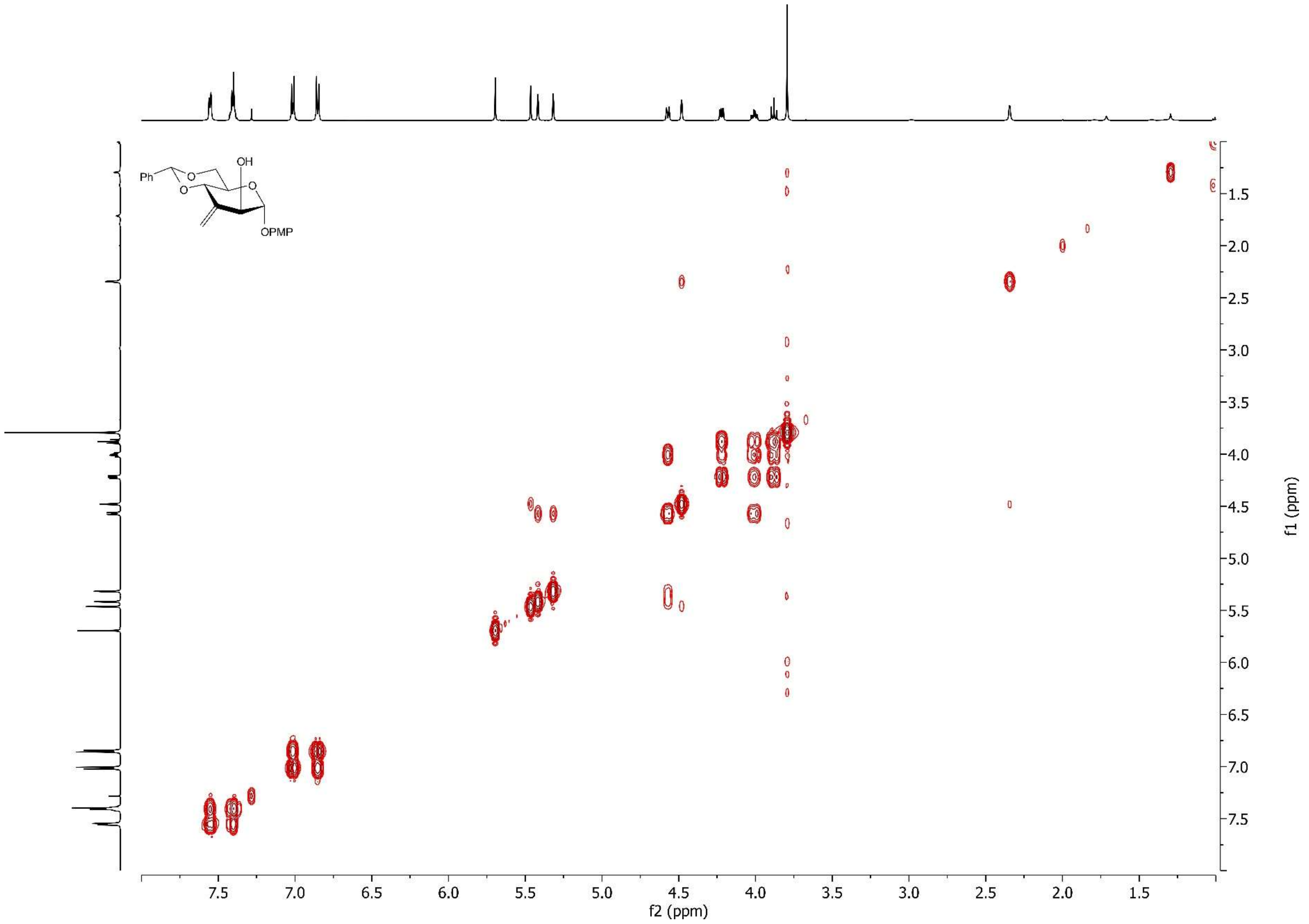

Figure S39. $^{13}C\{H\}$ NMR (151 MHz, $CDCl_3$) spectrum of *p*-methoxyphenyl 4,6-*O*-benzylidene-3-deoxy-3-methylene-α-D-*arabino*-hexopyranoside **19**:

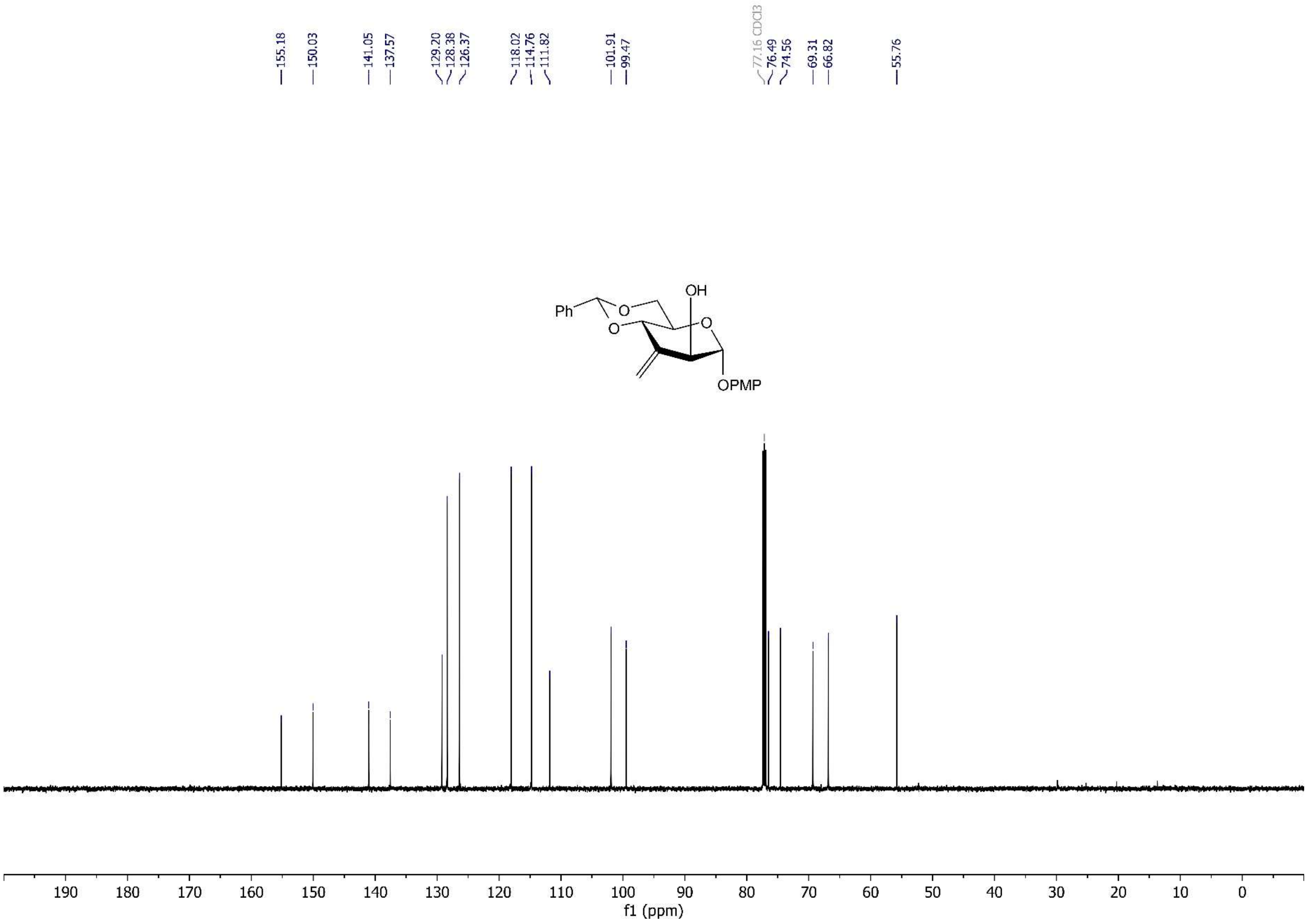

Figure S40. HSQC NMR (600 MHz, $CDCl_3$) spectrum of *p*-methoxyphenyl 4,6-*O*-benzylidene-3-deoxy-3-methylene-α-D-*arabino*-hexopyranoside **19**:

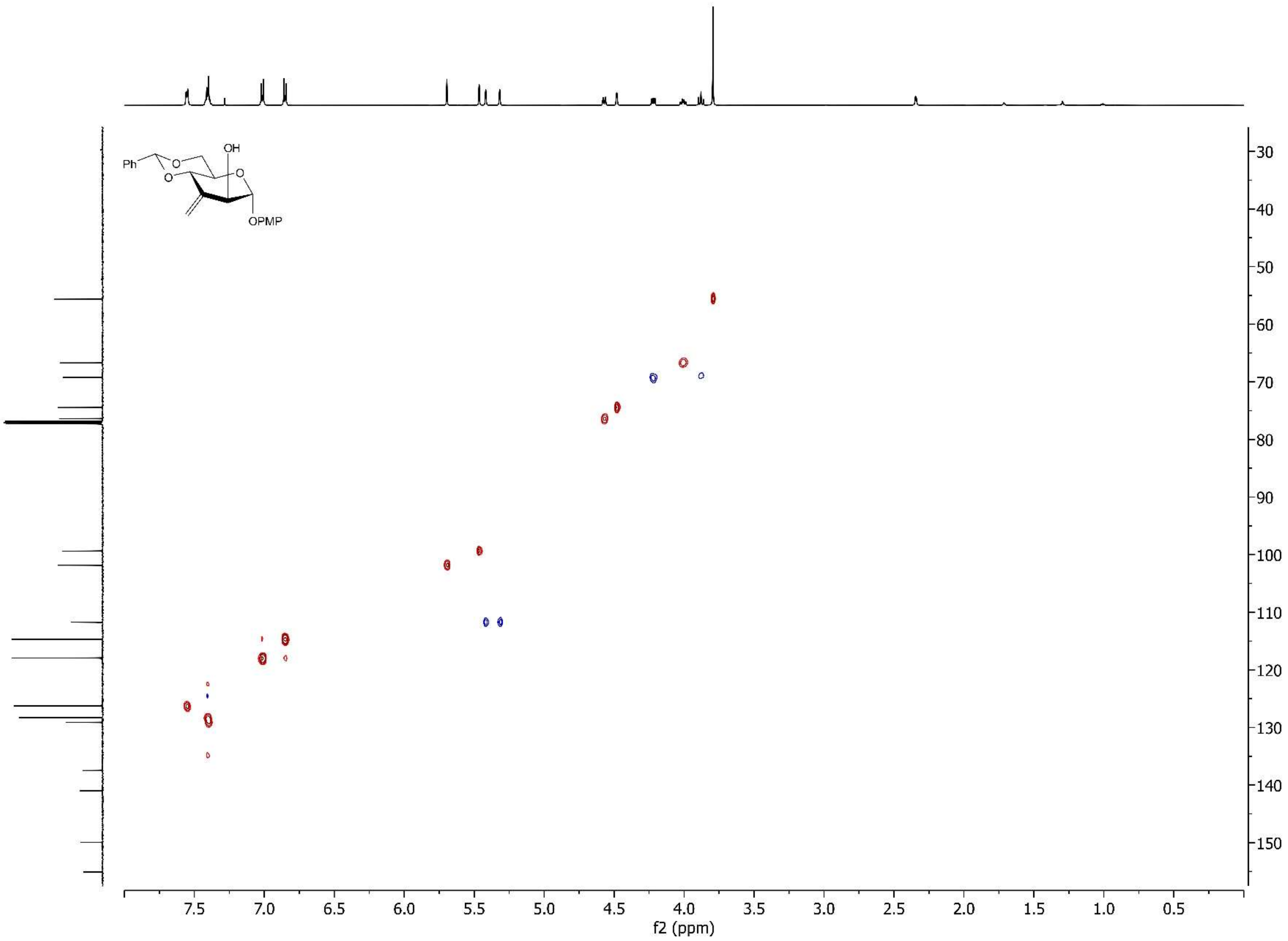

Figure S41. HMBC NMR (600 MHz, $CDCl_3$) spectrum of *p*-methoxyphenyl 4,6-*O*-benzylidene-3-deoxy-3-methylene-α-D-*arabino*-hexopyranoside **19**:

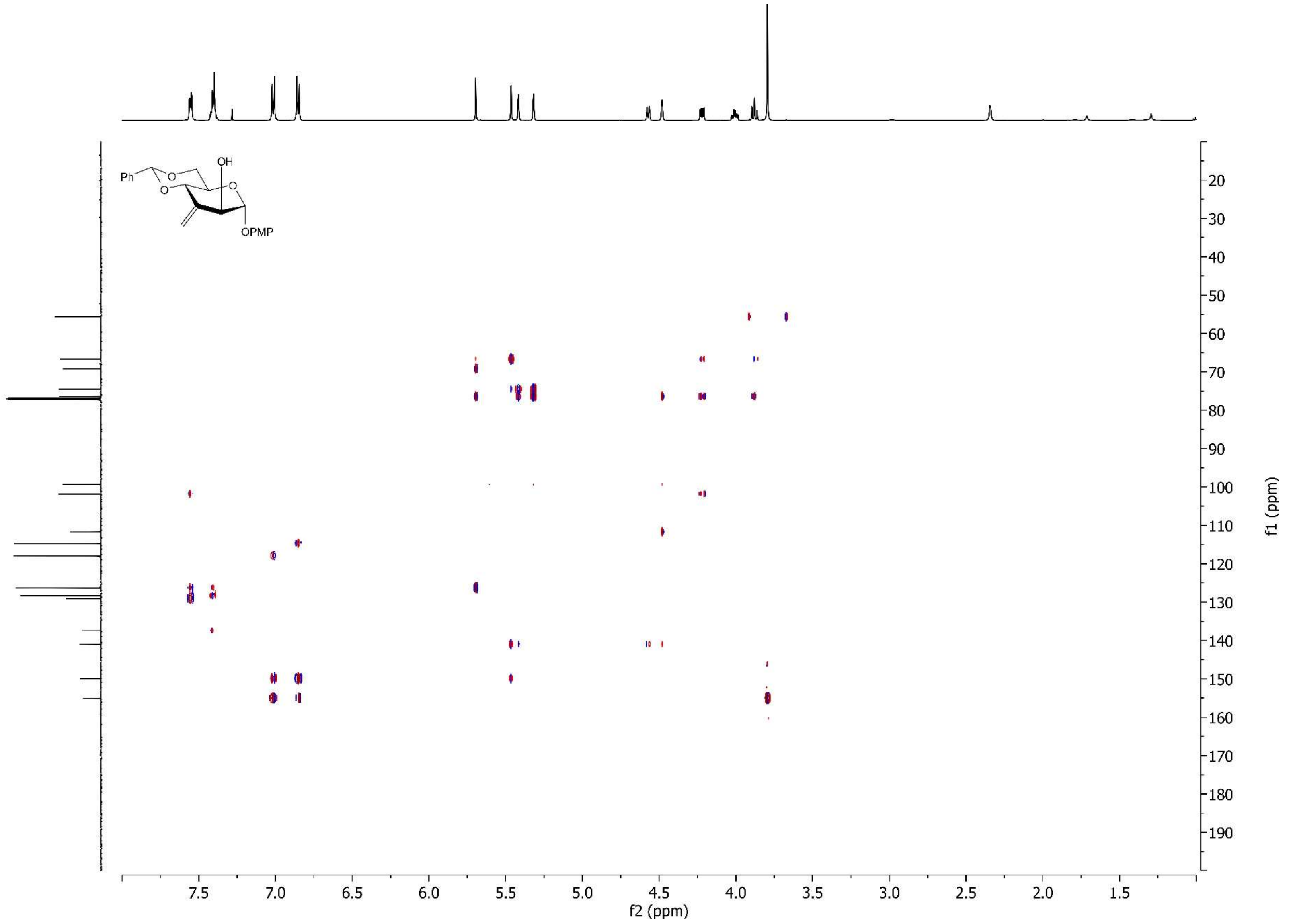

Figure S42. ^{1}H NMR (600 MHz, $CDCl_3$) spectrum of *p*-methoxyphenyl 3,3′-anhydro-4,6-*O*-benzylidene-3-*C*-hydroxymethyl-α-D-*manno*-hexopyranoside **20**:

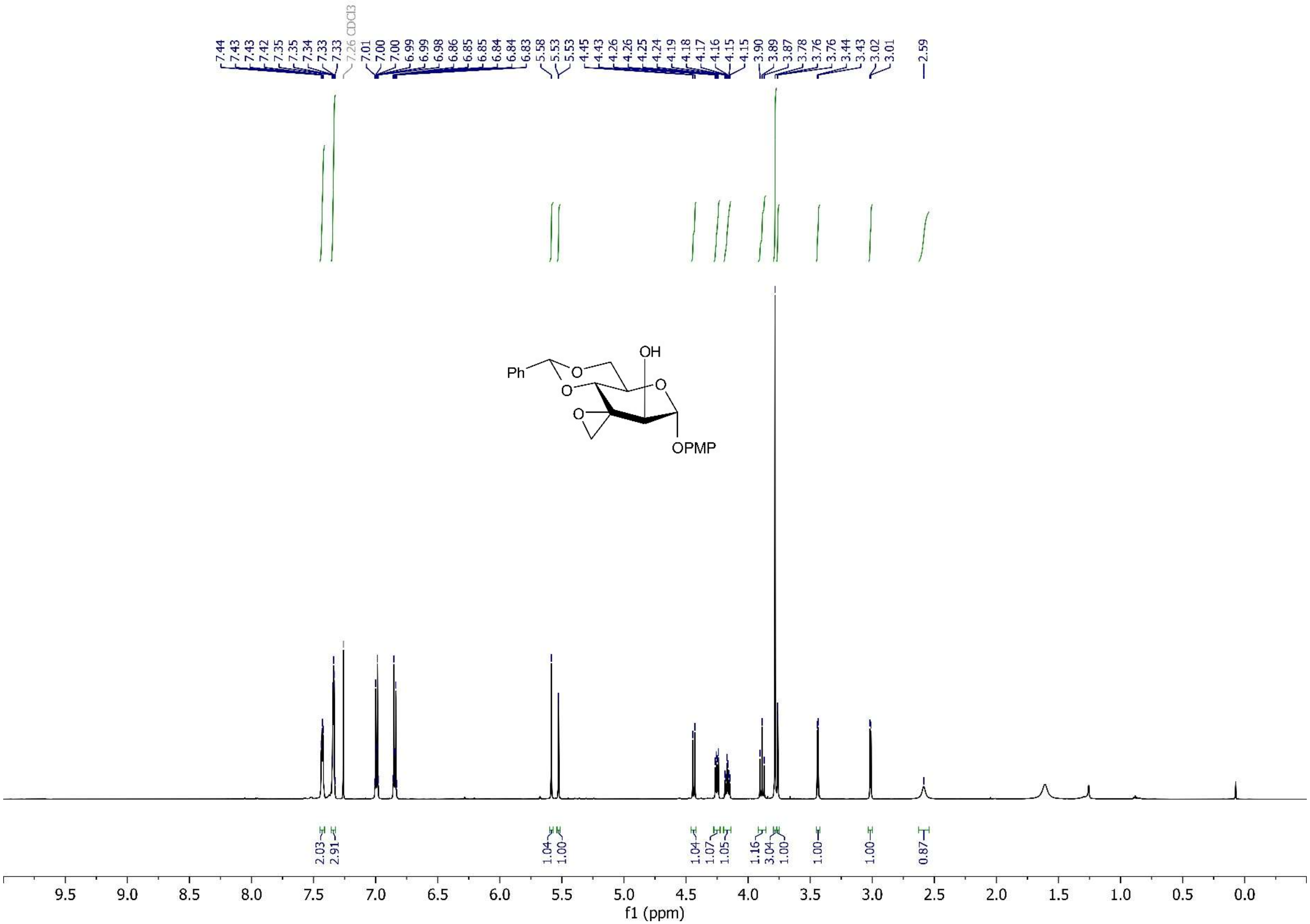

Figure S43. COSY NMR (600 MHz, $CDCl_3$) spectrum of *p*-methoxyphenyl 3,3'-anhydro-4,6-*O*-benzylidene-3-*C*-hydroxymethyl-α-D-*manno*-hexopyranoside **20**:

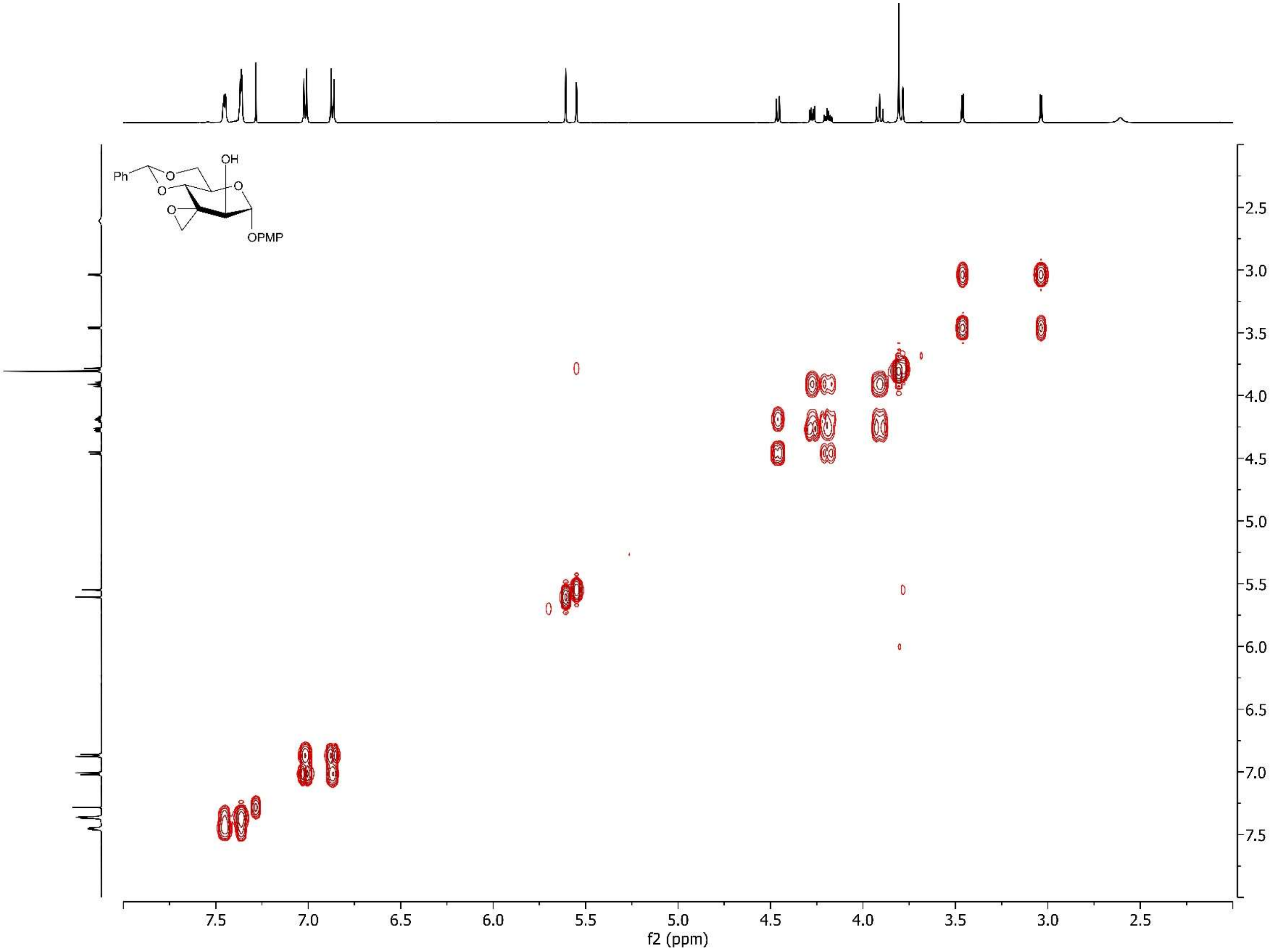

Figure S44. $^{13}C\{H\}$ NMR (151 MHz, $CDCl_3$) spectrum of *p*-methoxyphenyl 3,3'-anhydro-4,6-*O*-benzylidene-3-*C*-hydroxymethyl-α-D-*manno*-hexopyranoside **20**:

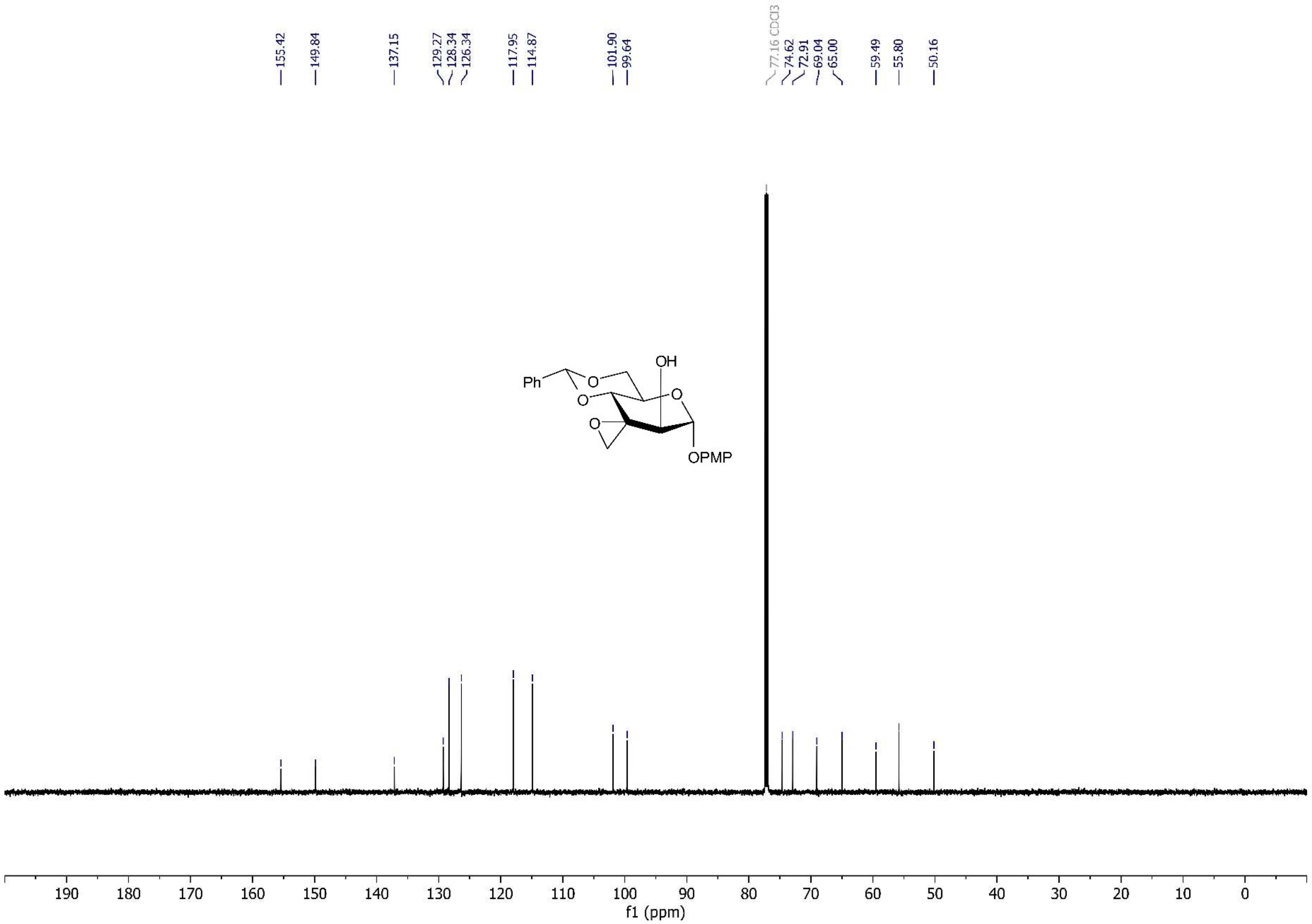

Figure S45. HSQC NMR (600 MHz, $CDCl_3$) spectrum of *p*-methoxyphenyl 3,3'-anhydro-4,6-*O*-benzylidene-3-*C*-hydroxymethyl-α-D-*manno*-hexopyranoside **20**:

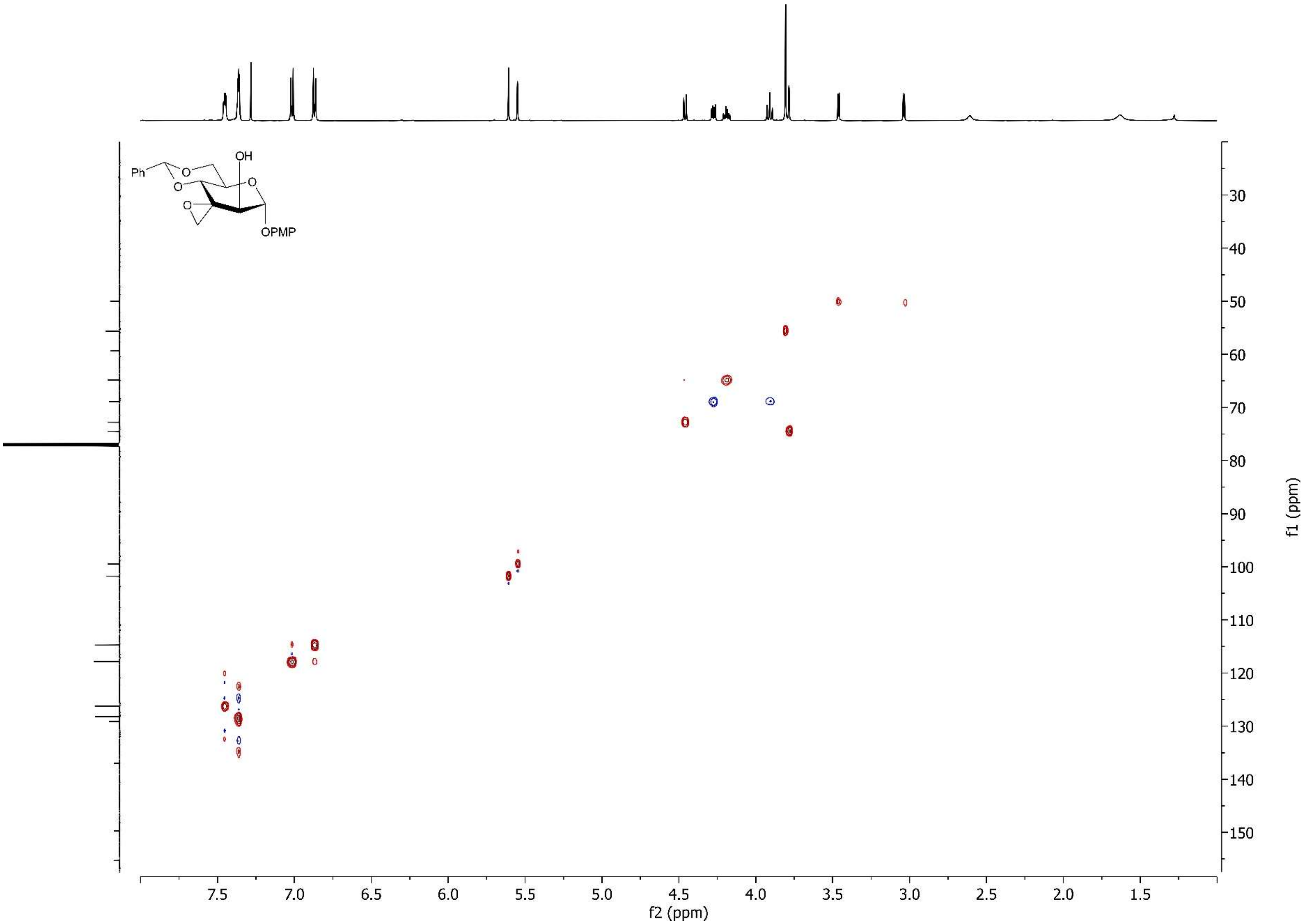

Figure S46. HMBC NMR (600 MHz, $CDCl_3$) spectrum of *p*-methoxyphenyl 3,3'-anhydro-4,6-*O*-benzylidene-3-*C*-hydroxymethyl-α-D-*manno*-hexopyranoside **20**:

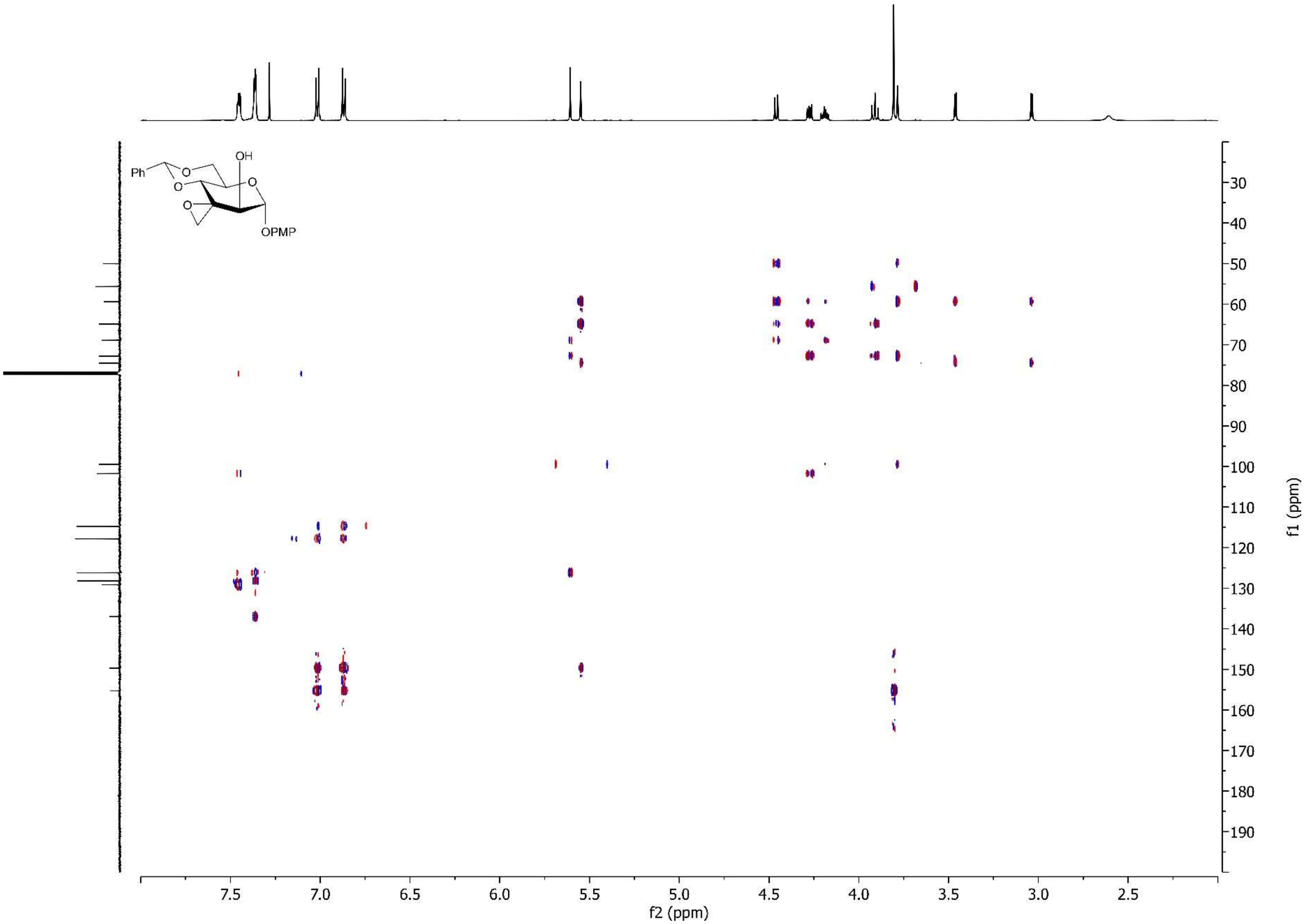

Figure S47. ^{1}H NMR (600 MHz, $CDCl_3$) spectrum of *p*-methoxyphenyl 4,6-*O*-benzylidene-3-*C*-methyl-α-D-mannopyranoside **21**:

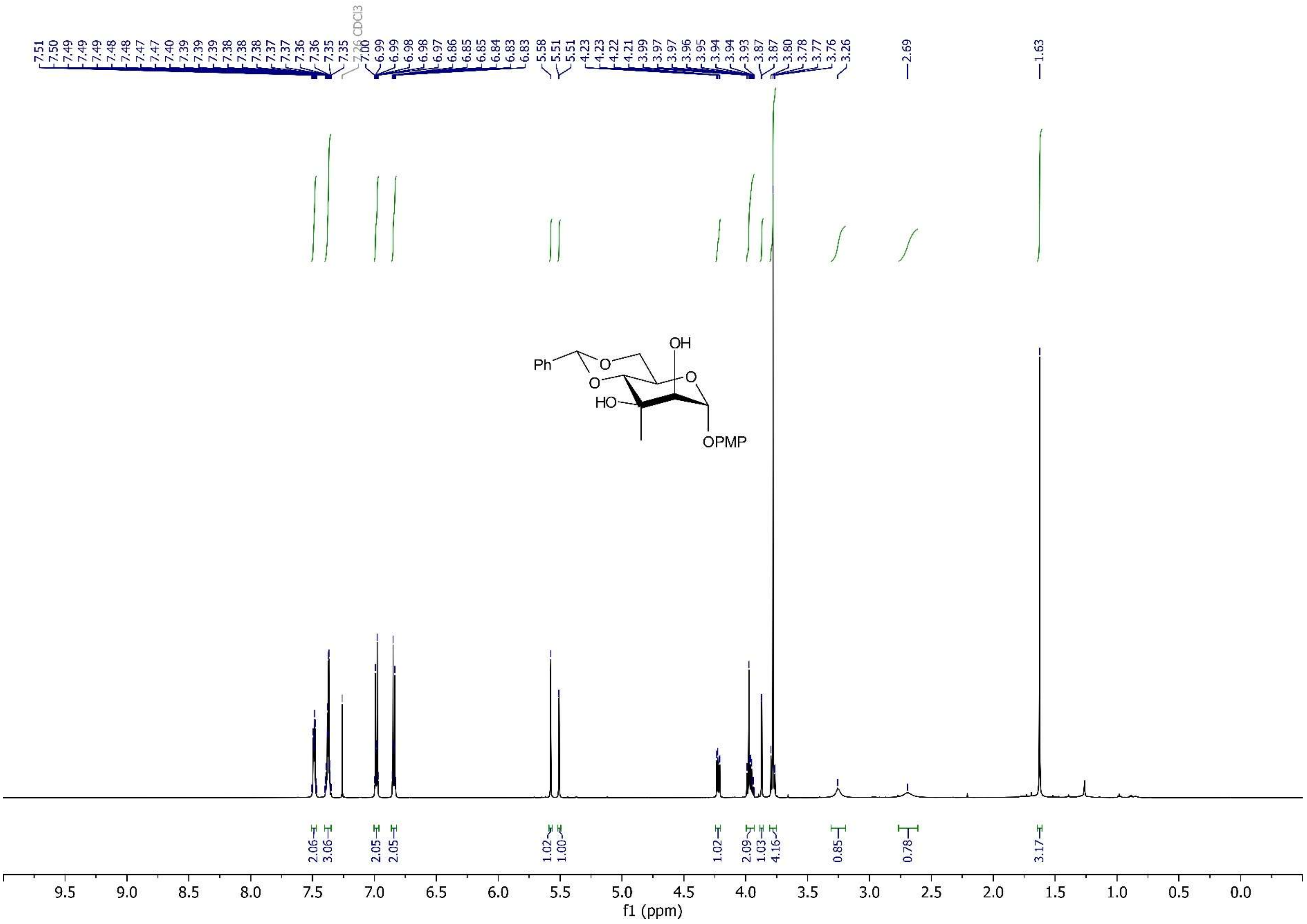

Figure S48. COSY NMR (600 MHz, $CDCl_3$) spectrum of *p*-methoxyphenyl 4,6-*O*-benzylidene-3-*C*-methyl-α-D-mannopyranoside **21**:

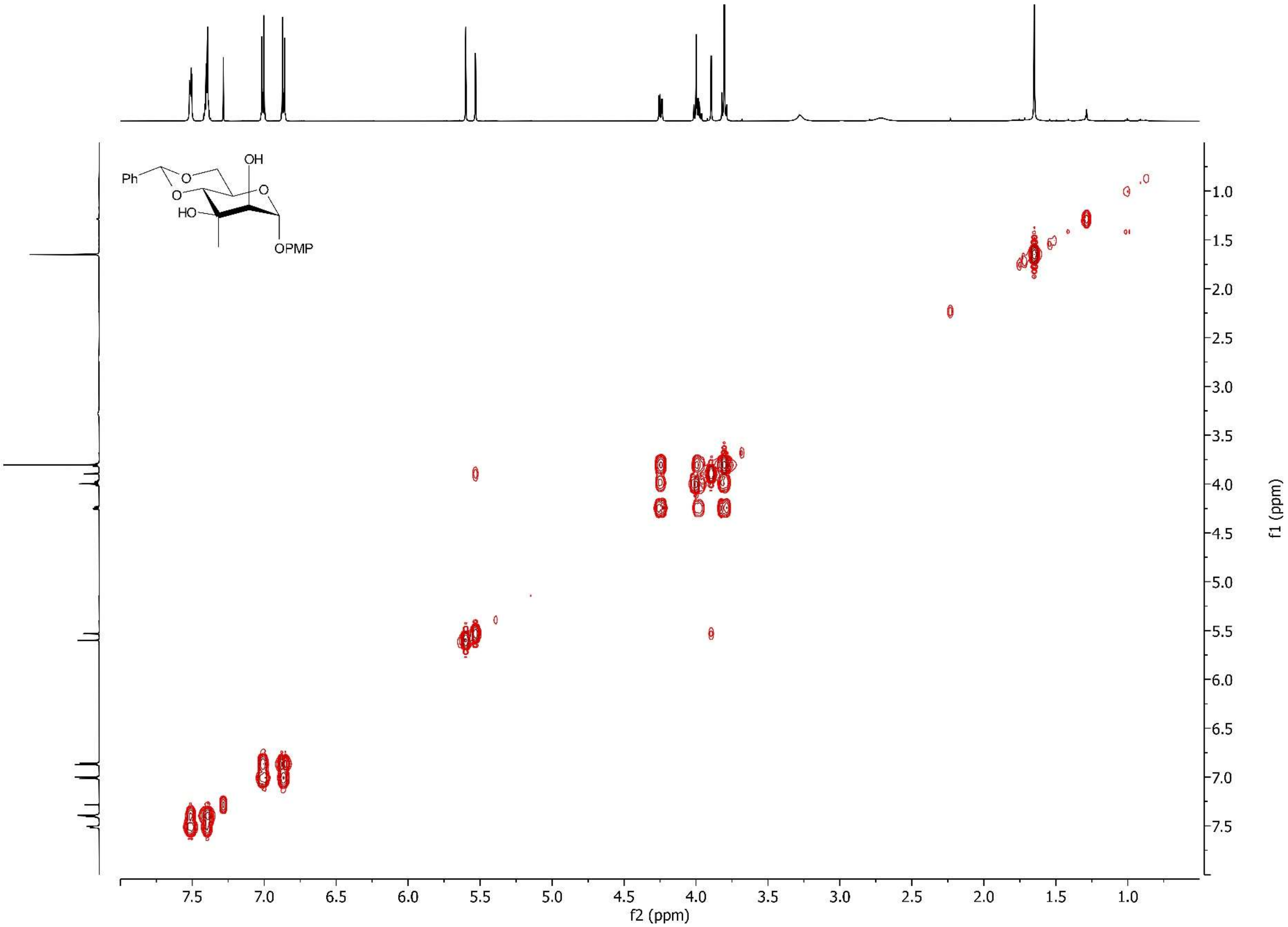

Figure S49. $^{13}C\{H\}$ NMR (151 MHz, $CDCl_3$) spectrum of *p*-methoxyphenyl 4,6-*O*-benzylidene-3-*C*-methyl-α-D-mannopyranoside **21**:

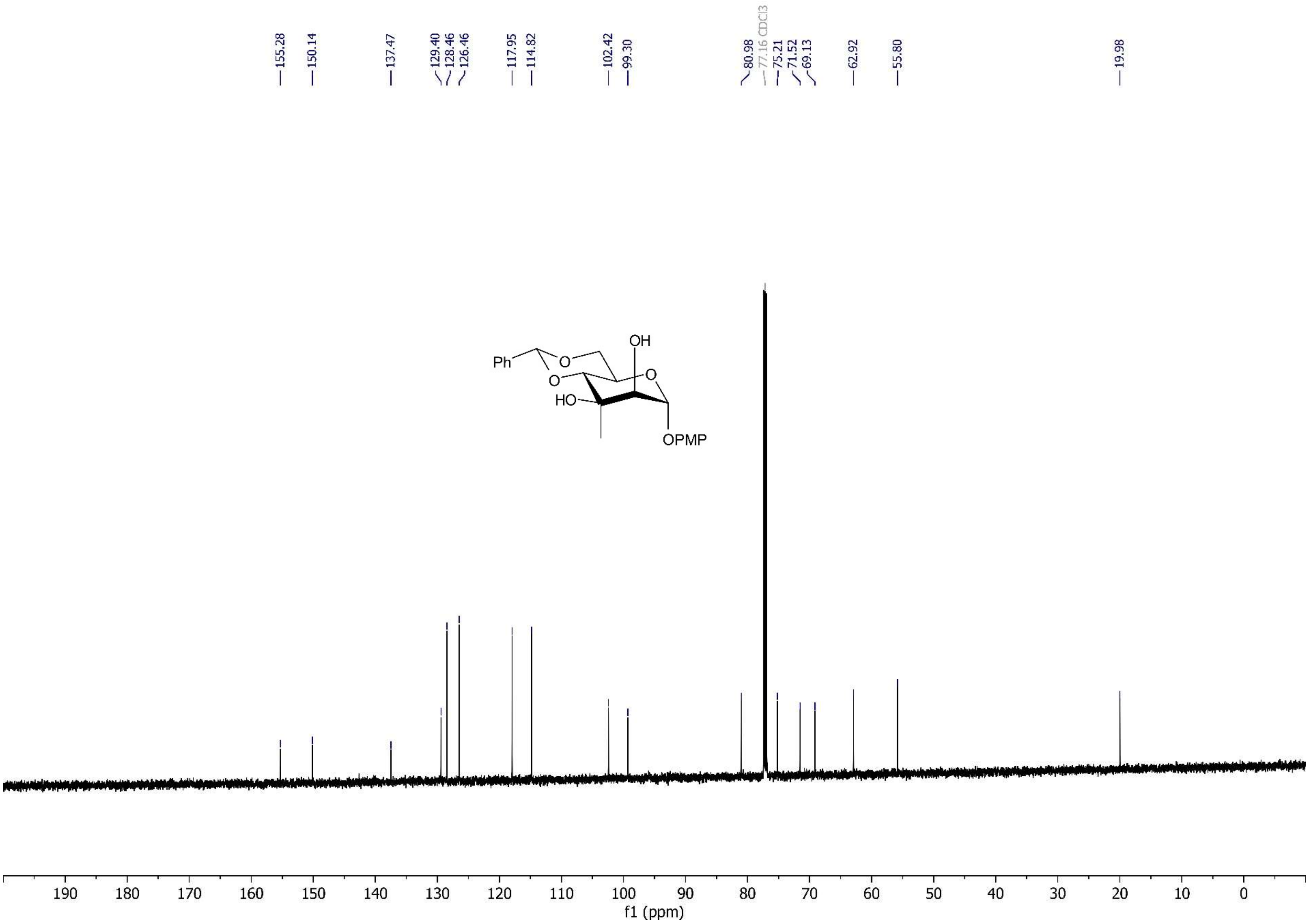

Figure S50. HSQC NMR (600 MHz, $CDCl_3$) spectrum of *p*-methoxyphenyl 4,6-*O*-benzylidene-3-*C*-methyl-α-D-mannopyranoside **21**:

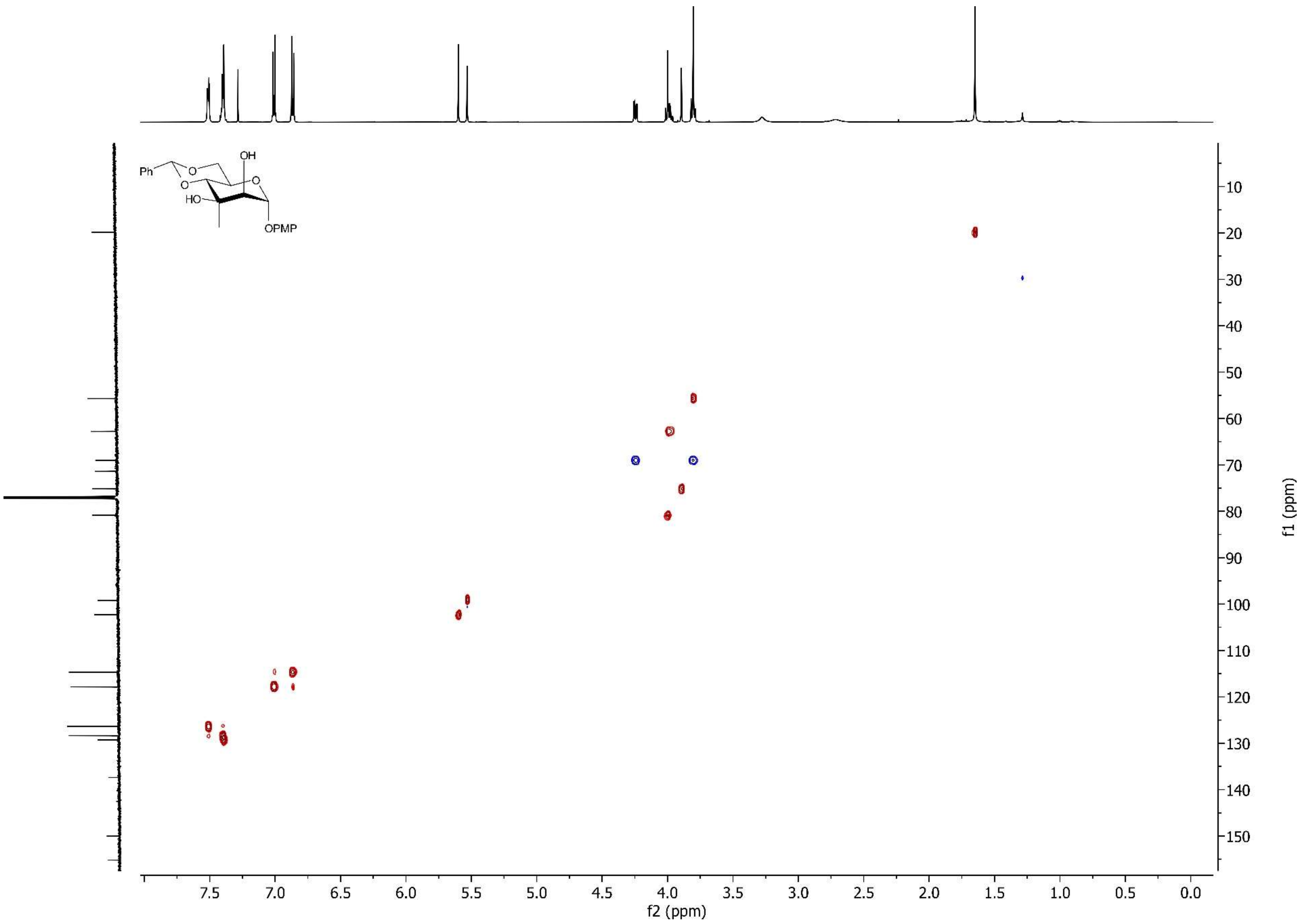

Figure S51. HMBC NMR (600 MHz, $CDCl_3$) spectrum of *p*-methoxyphenyl 4,6-*O*-benzylidene-3-*C*-methyl-α-D-mannopyranoside **21**:

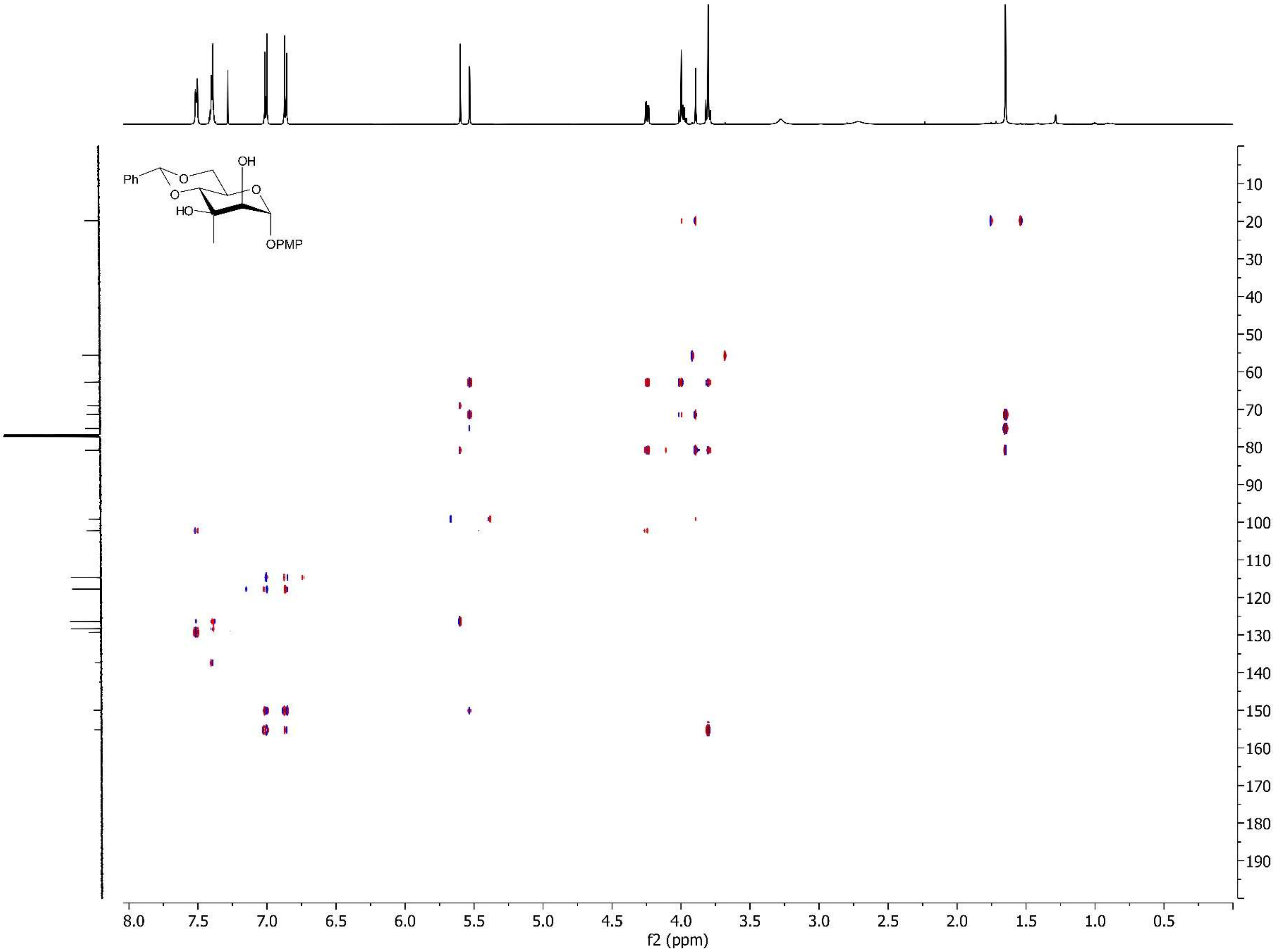

Figure S52. ^{1}H NMR (600 MHz, $CDCl_3$) spectrum of *p*-methoxyphenyl 4,6-*O*-benzylidene-2-*O*-*tert*-butyldimethylsilyl-3-*C*-methyl-α-D-mannopyranoside **22**:

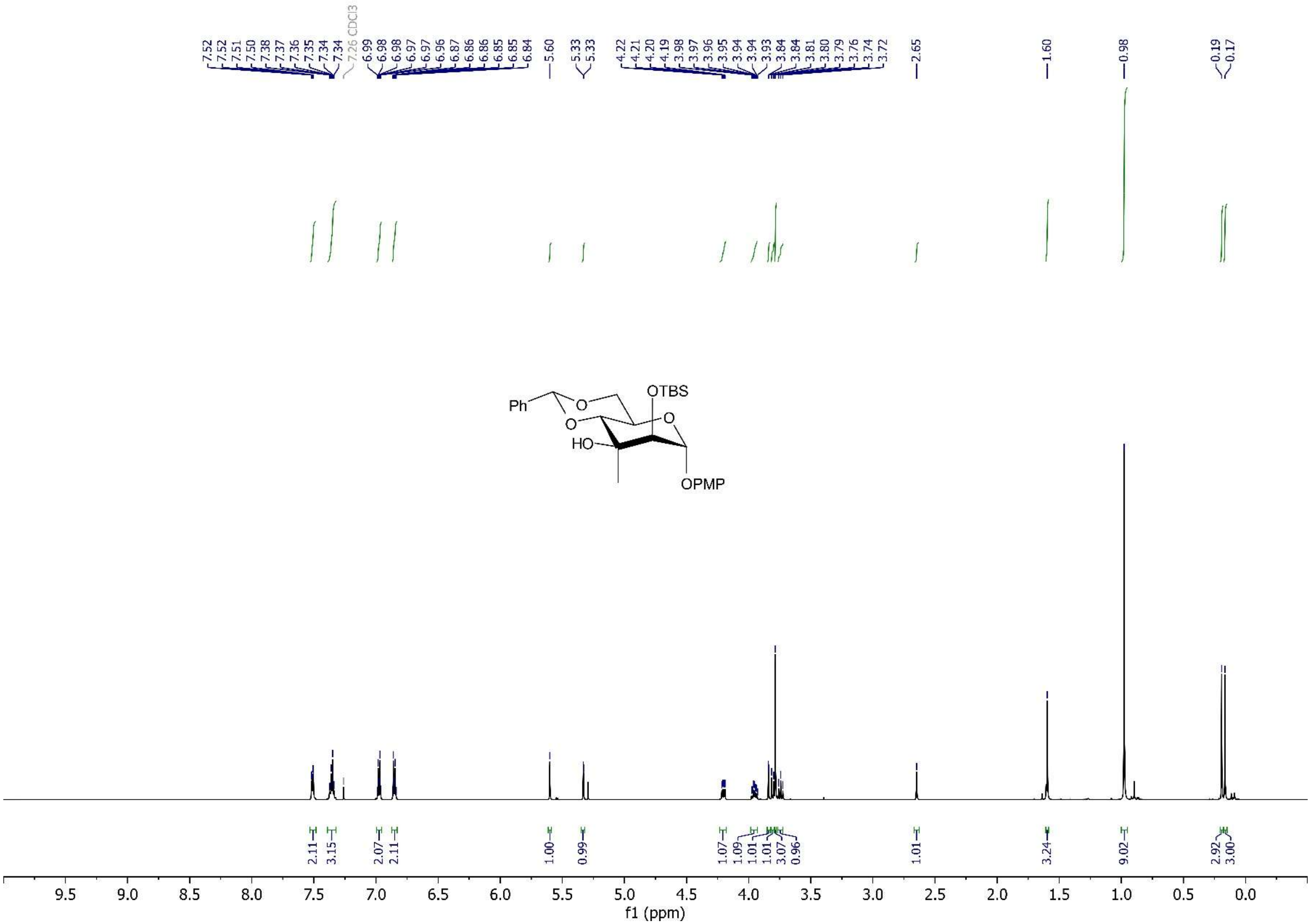

Figure S53. COSY NMR (600 MHz, $CDCl_3$) spectrum of *p*-methoxyphenyl 4,6-*O*-benzylidene-2-*O*-*tert*-butyldimethylsilyl-3-*C*-methyl-α-D-mannopyranoside **22**:

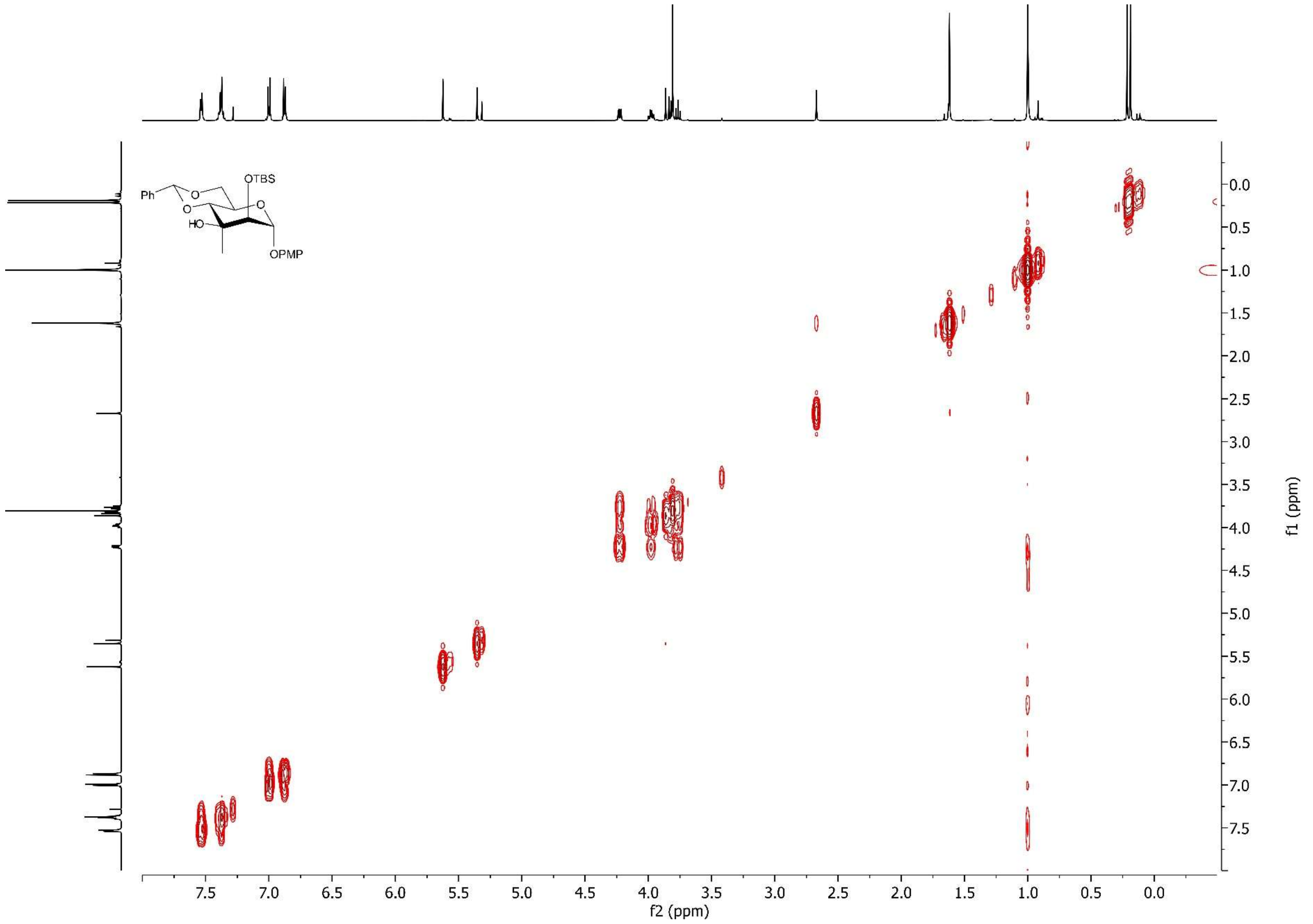

Figure S54. ^{13}C{H} NMR (151 MHz, CDCl$_3$) spectrum of *p*-methoxyphenyl 4,6-*O*-benzylidene-2-*O*-*tert*-butyldimethylsilyl-3-*C*-methyl-α-D-mannopyranoside **22**:

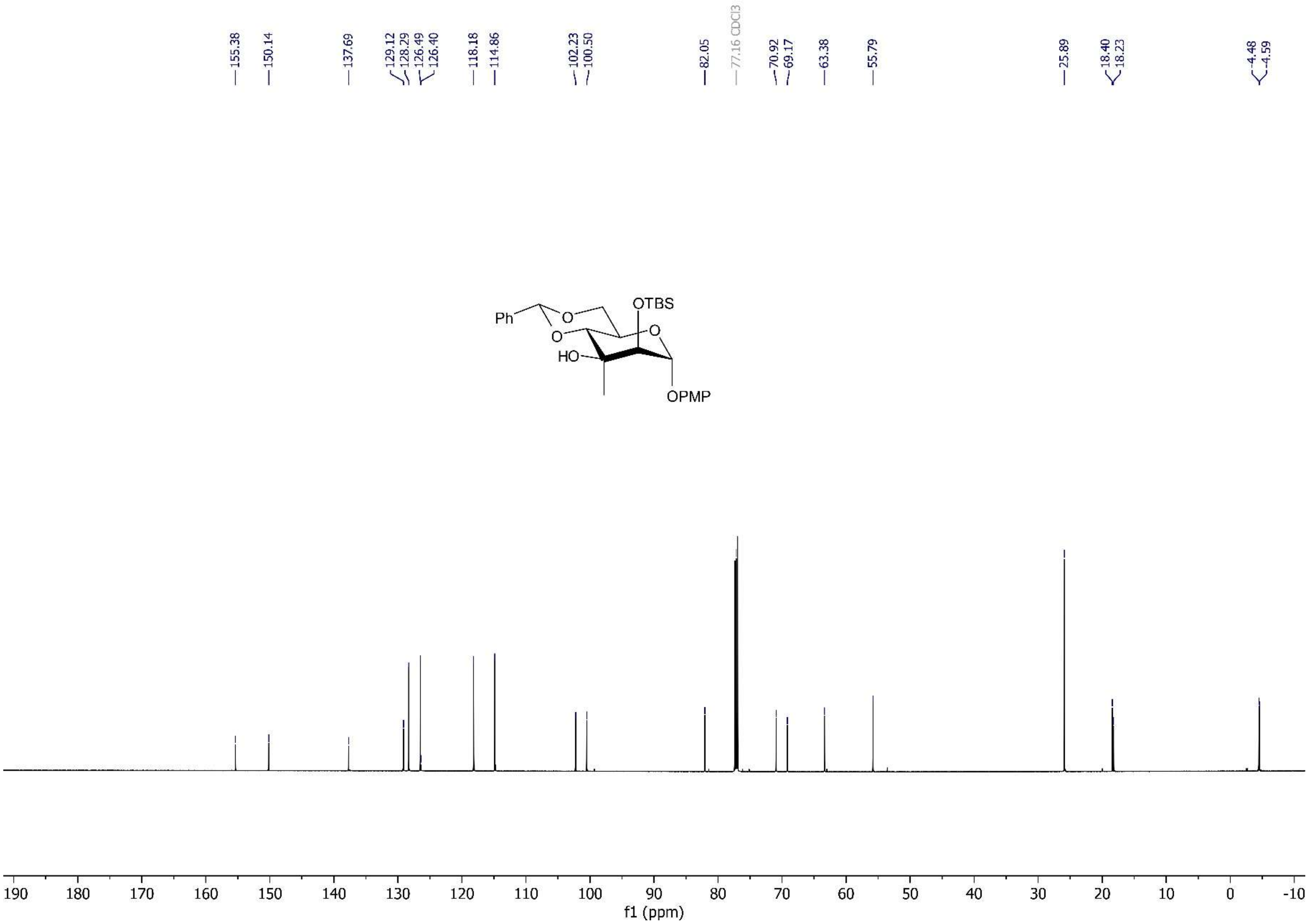

Figure S55. HSQC NMR (600 MHz, $CDCl_3$) spectrum of *p*-methoxyphenyl 4,6-*O*-benzylidene-2-*O*-*tert*-butyldimethylsilyl-3-*C*-methyl-α-D-mannopyranoside **22**:

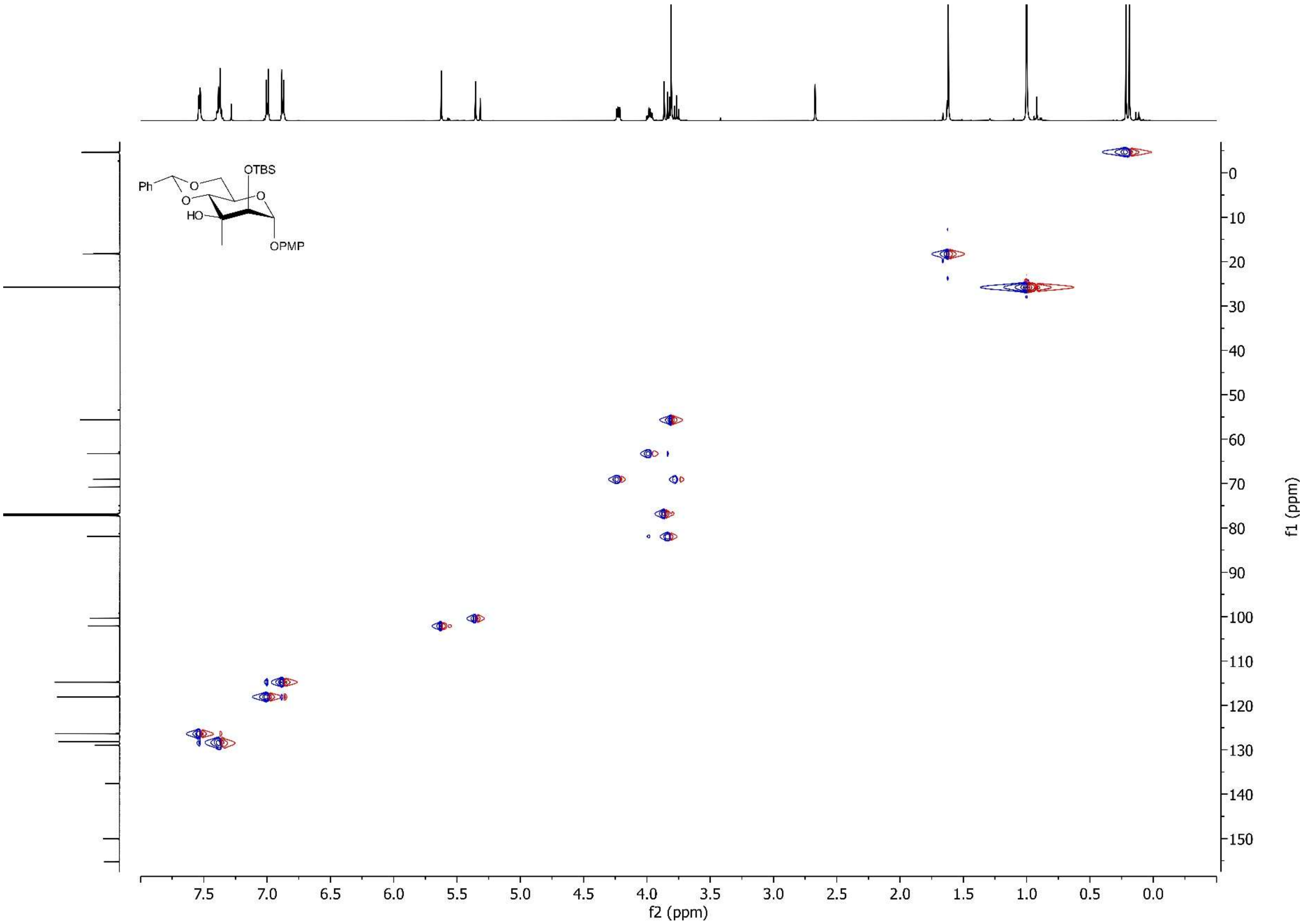

Figure S56. HMBC NMR (600 MHz, $CDCl_3$) spectrum of *p*-methoxyphenyl 4,6-*O*-benzylidene-2-*O*-*tert*-butyldimethylsilyl-3-*C*-methyl-α-D-mannopyranoside **22**:

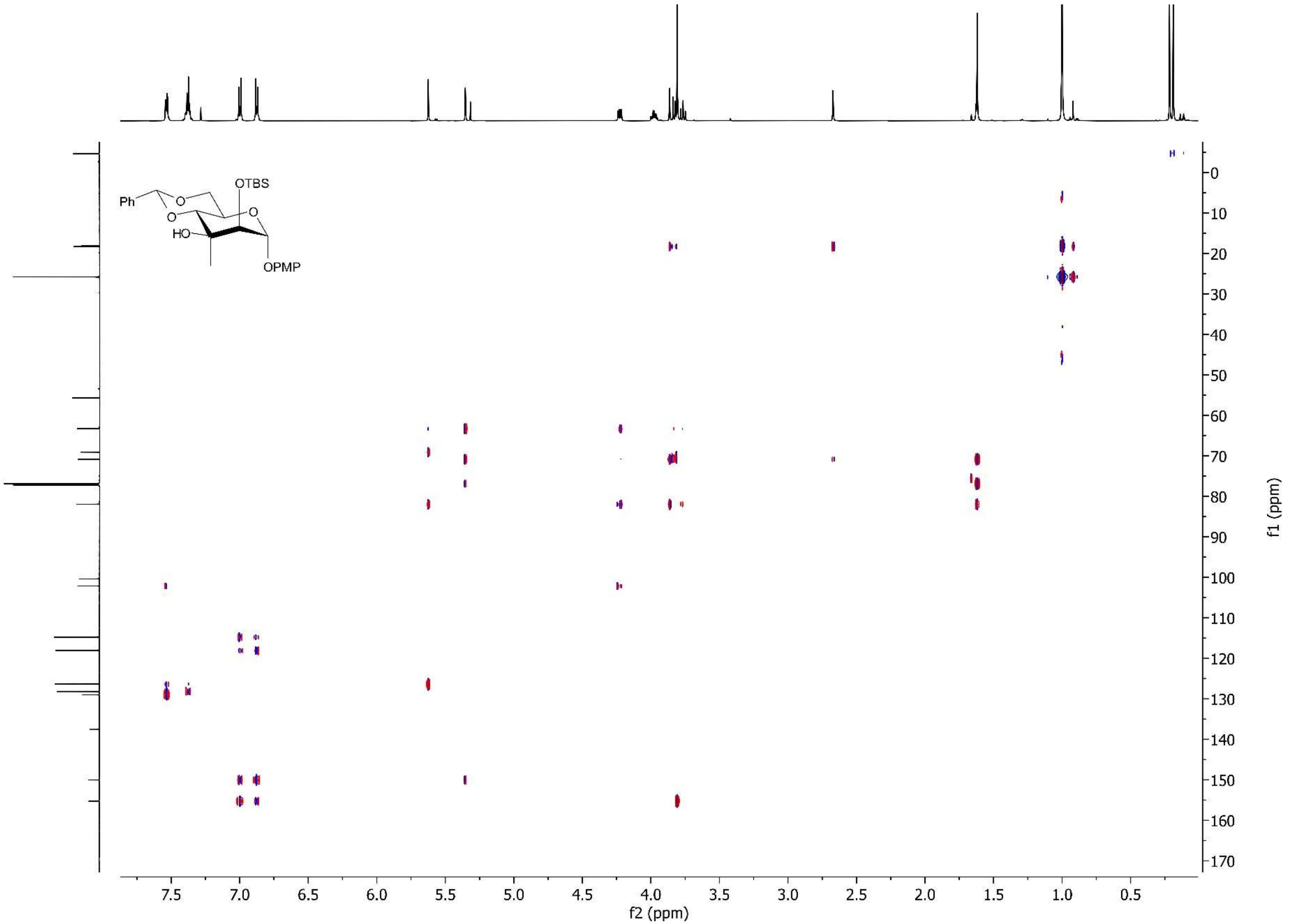

Figure S57. ^{1}H (NOE) NMR (600 MHz, $CDCl_3$) spectrum of *p*-methoxyphenyl 4,6-*O*-benzylidene-2-*O*-*tert*-butyldimethylsilyl-3-*C*-methyl-α-D-mannopyranoside **22**:

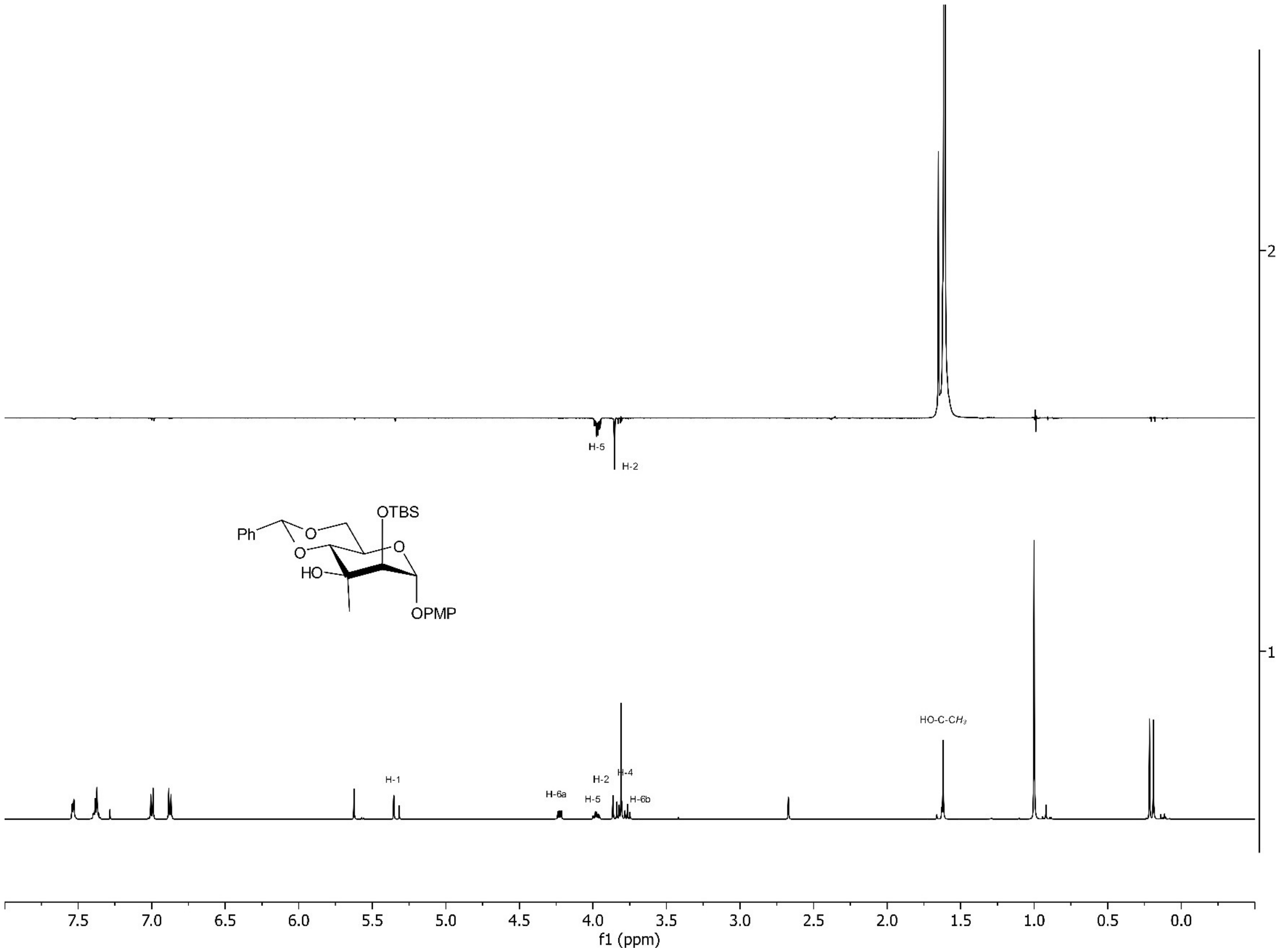

Figure S58. Comparison of ^{1}H NMR (600 MHz, $CDCl_3$) spectra of *p*-methoxyphenyl 4,6-*O*-benzylidene-2-*O*-*tert*-butyldimethylsilyl-3-*C*-methyl-α-D-altropyranoside **17** and *p*-methoxyphenyl 4,6-*O*-benzylidene-2-*O*-*tert*-butyldimethylsilyl-3-*C*-methyl-α-D-mannopyranoside **22**:

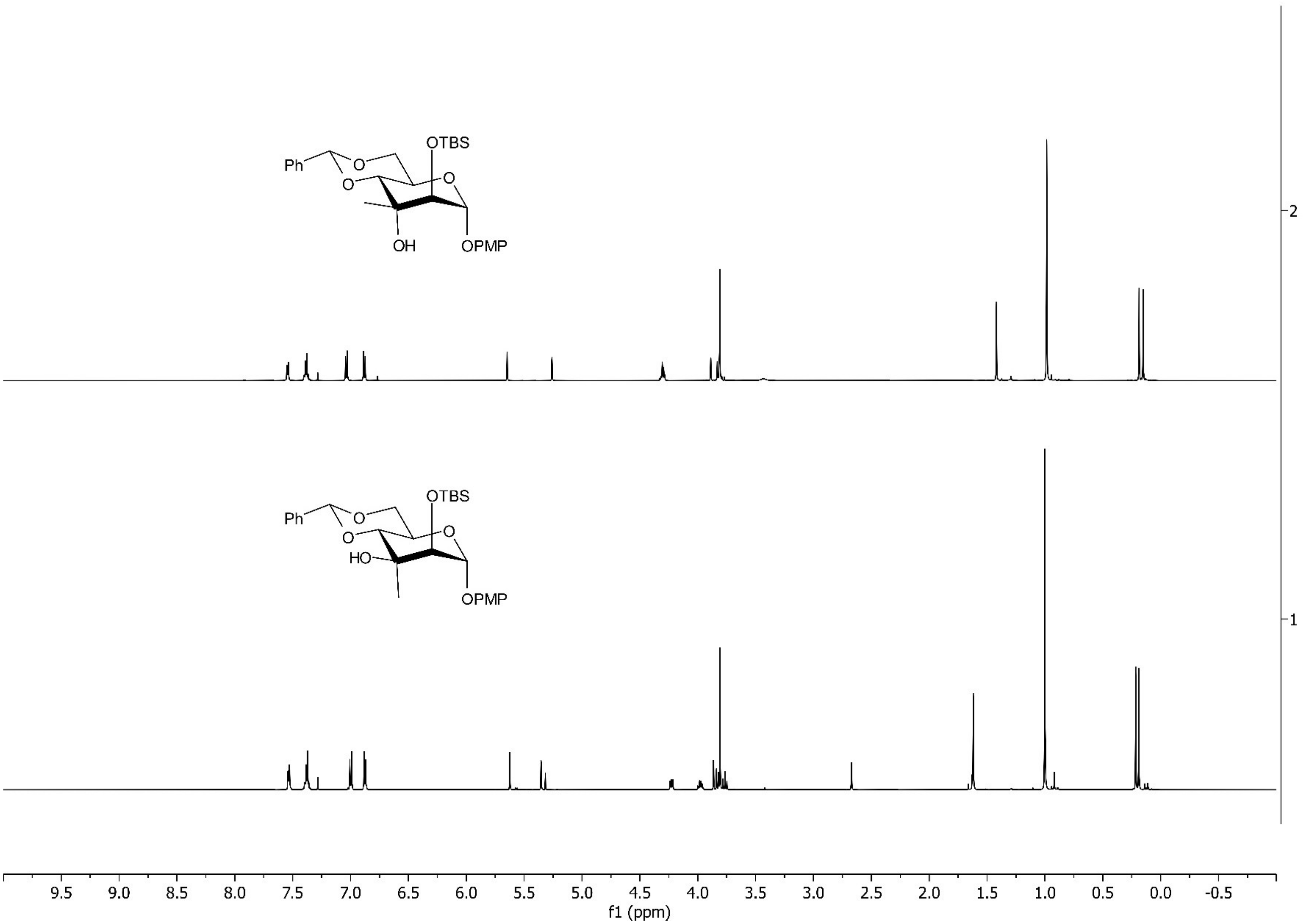

Figure S59. Comparison of $^{13}C\{H\}$ NMR (151 MHz, $CDCl_3$) spectra of *p*-methoxyphenyl 4,6-*O*-benzylidene-2-*O*-*tert*-butyldimethylsilyl-3-*C*-methyl-α-D-altropyranoside **17** and *p*-methoxyphenyl 4,6-*O*-benzylidene-2-*O*-*tert*-butyldimethylsilyl-3-*C*-methyl-α-D-mannopyranoside **22**:

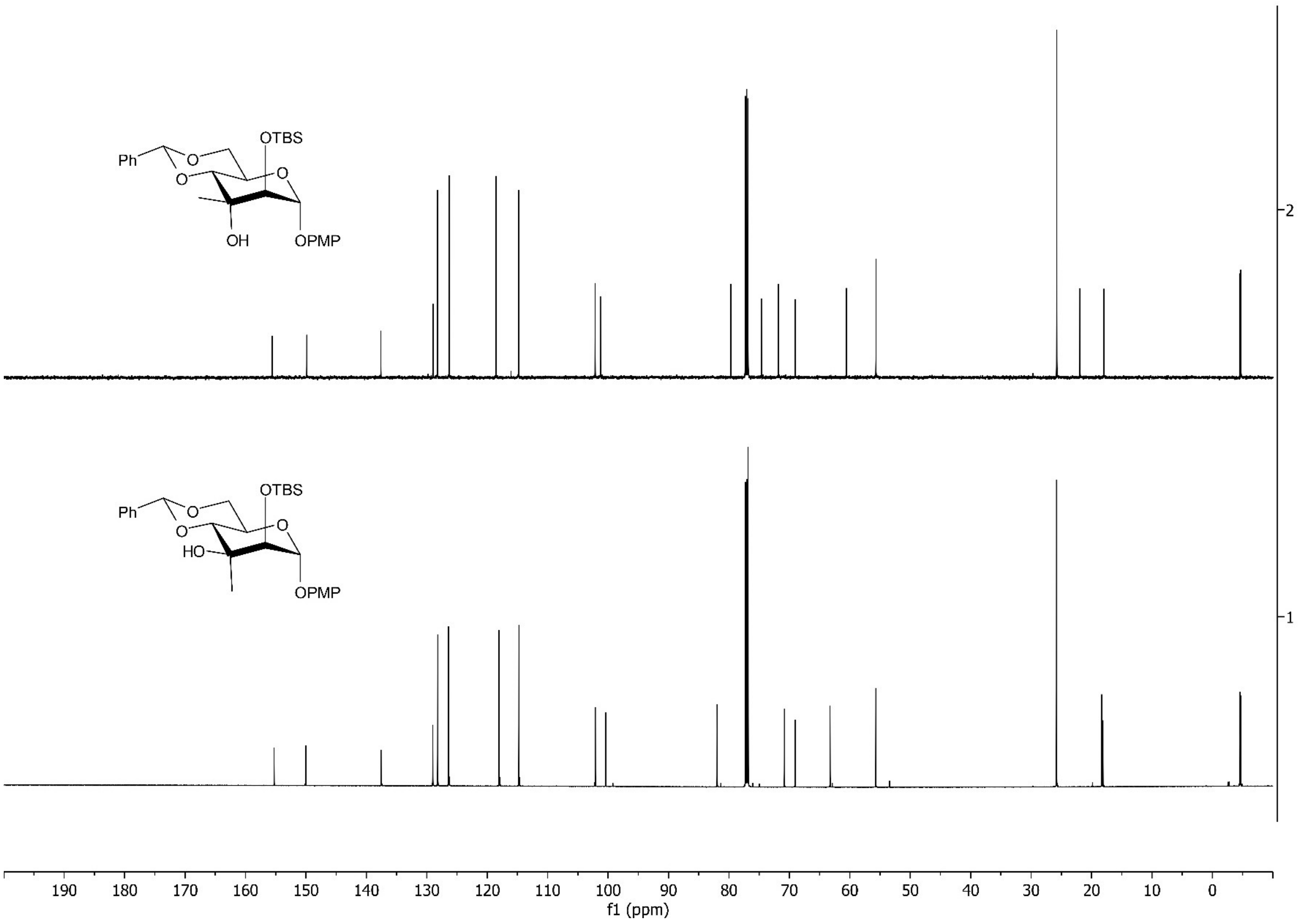

Figure S60. ^{1}H NMR (600 MHz, $CDCl_3$) spectrum of *p*-methoxyphenyl 2-*O*-benzyl-4,6-*O*-benzylidene-3-*C*-methyl-α-D-mannopyranoside **23**:

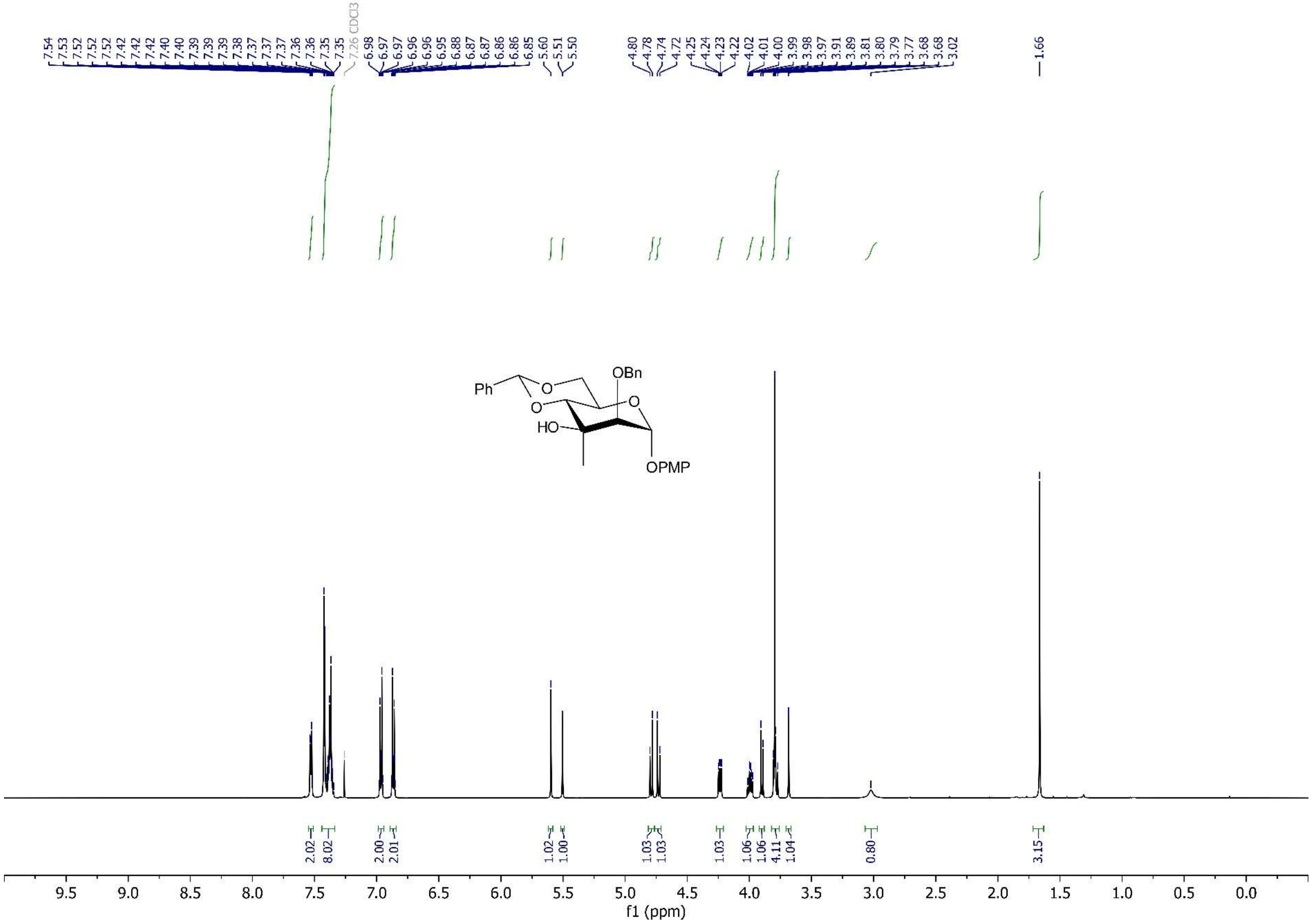

Figure S61. COSY NMR (600 MHz, $CDCl_3$) spectrum of *p*-methoxyphenyl 2-*O*-benzyl-4,6-*O*-benzylidene-3-*C*-methyl-α-D-mannopyranoside **23**:

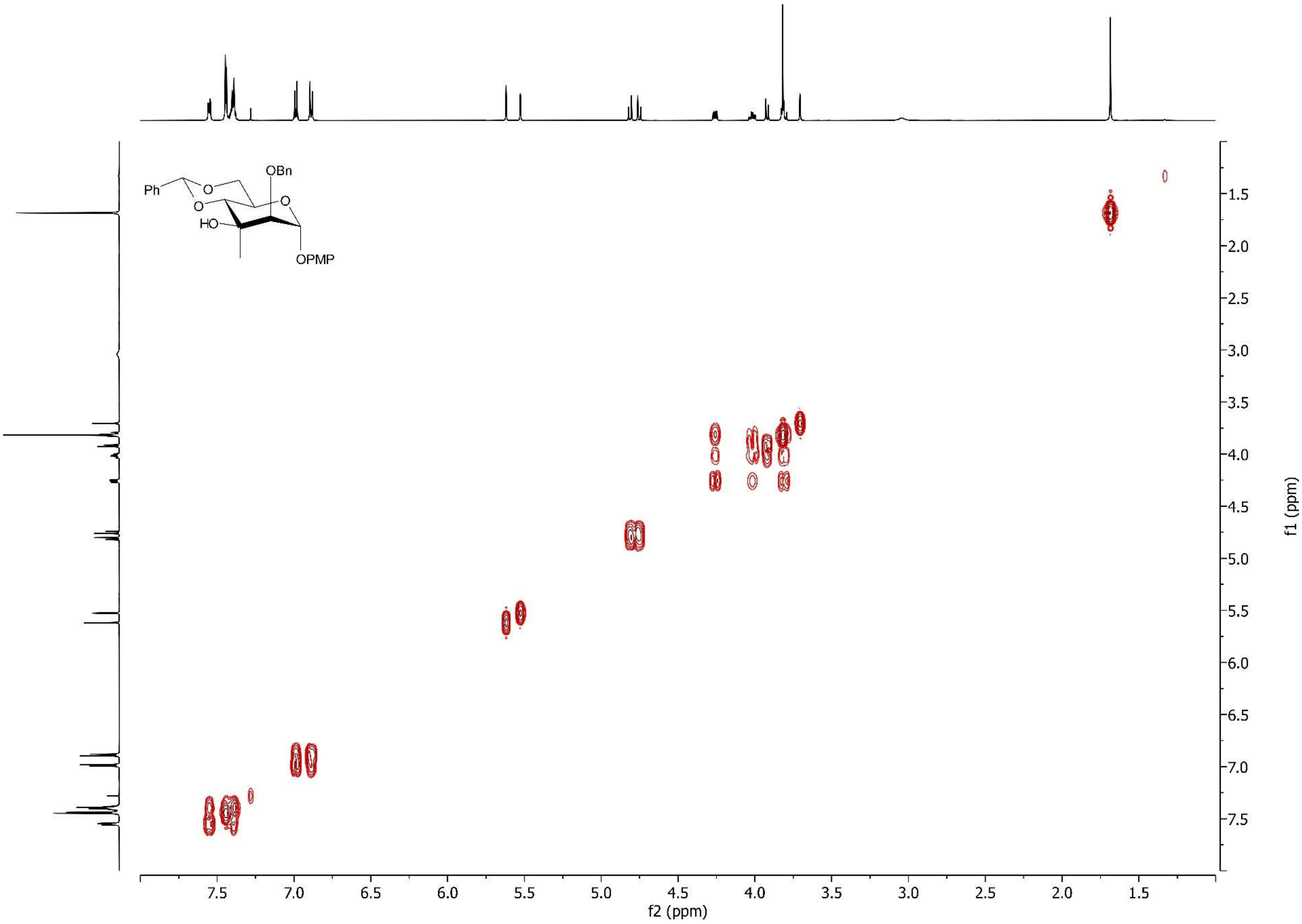

Figure S62. ^{13}C{H} NMR (151 MHz, $CDCl_3$) spectrum of *p*-methoxyphenyl 2-*O*-benzyl-4,6-*O*-benzylidene-3-*C*-methyl-α-D-mannopyranoside **23**:

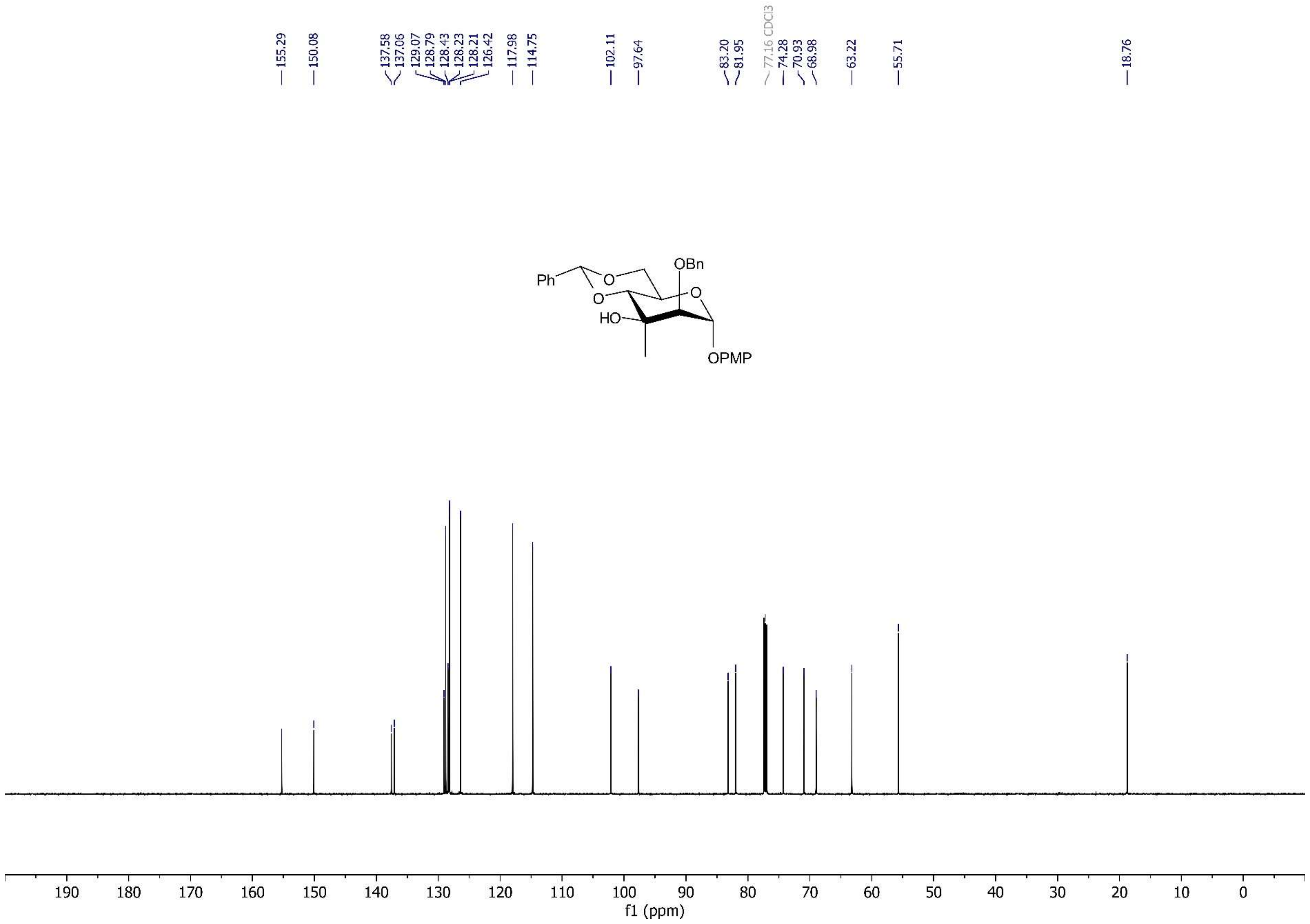

Figure S63. HSQC NMR (600 MHz, $CDCl_3$) spectrum of *p*-methoxyphenyl 2-*O*-benzyl-4,6-*O*-benzylidene-3-*C*-methyl-α-D-mannopyranoside **23**:

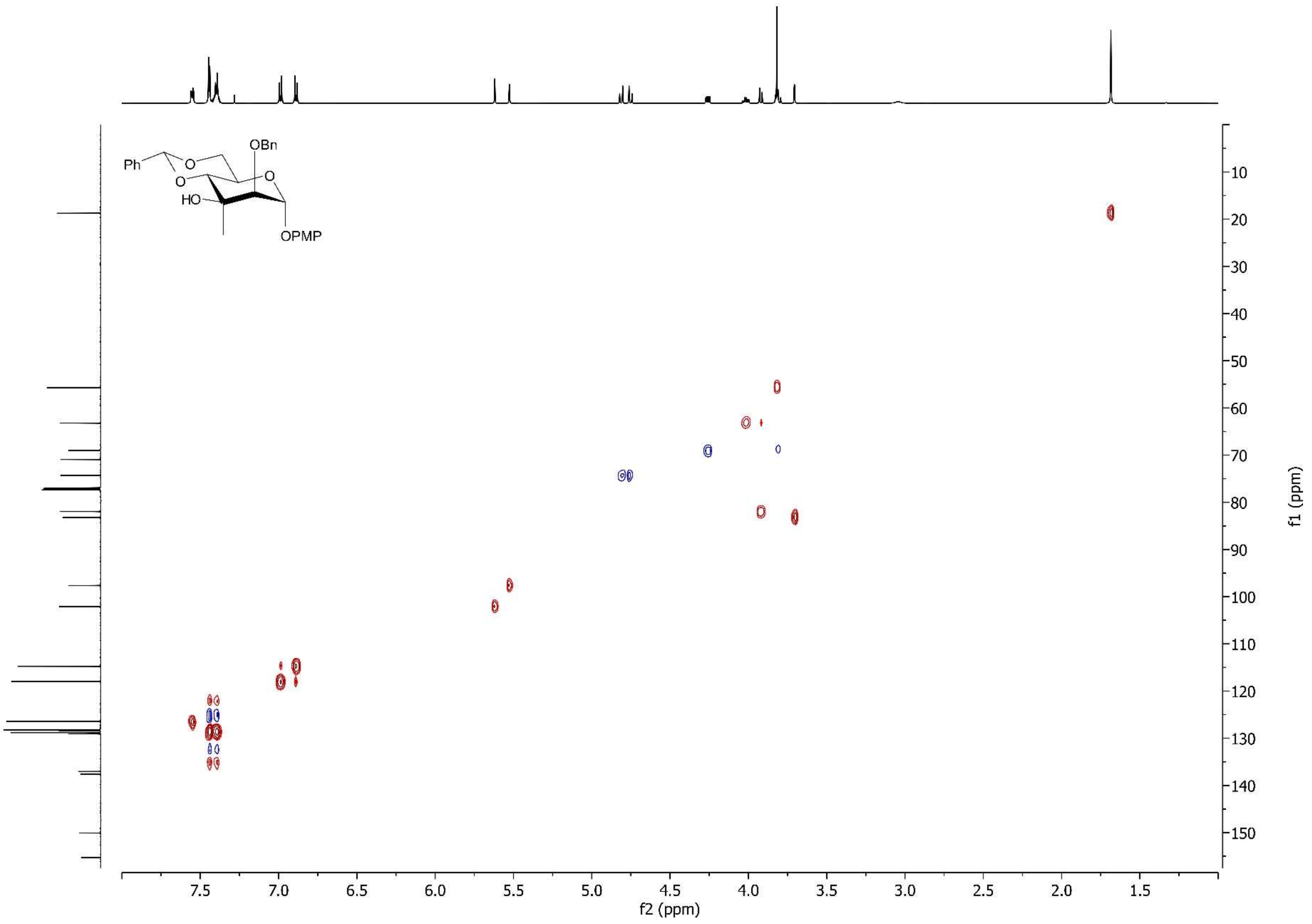

Figure S64. HMBC NMR (600 MHz, $CDCl_3$) spectrum of *p*-methoxyphenyl 2-*O*-benzyl-4,6-*O*-benzylidene-3-*C*-methyl-α-D-mannopyranoside **23**:

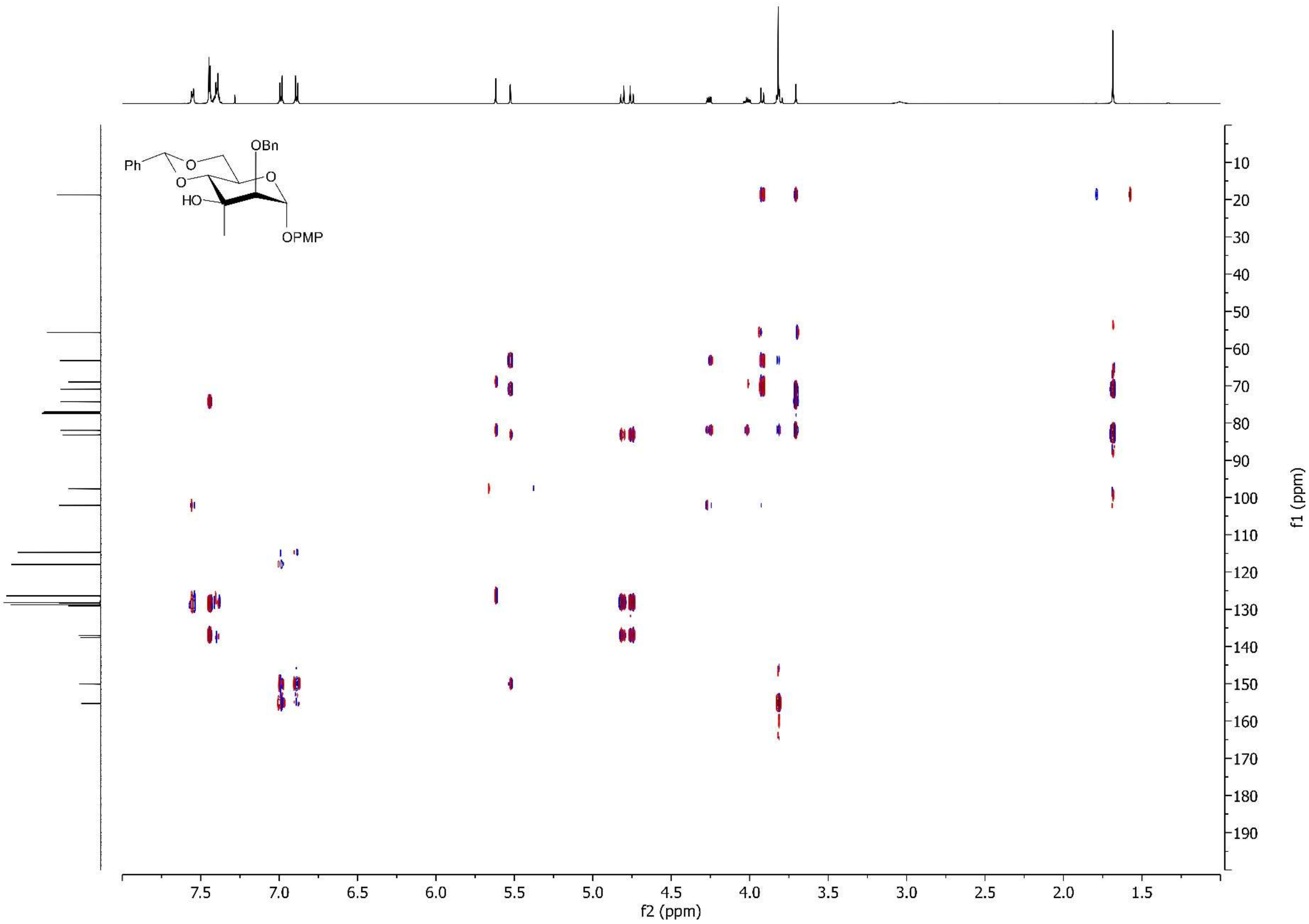

Figure S65. ^{1}H (NOE) NMR (600 MHz, $CDCl_3$) spectrum of *p*-methoxyphenyl 2-*O*-benzyl-4,6-*O*-benzylidene-3-*C*-methyl-α-D-mannopyranoside **23**:

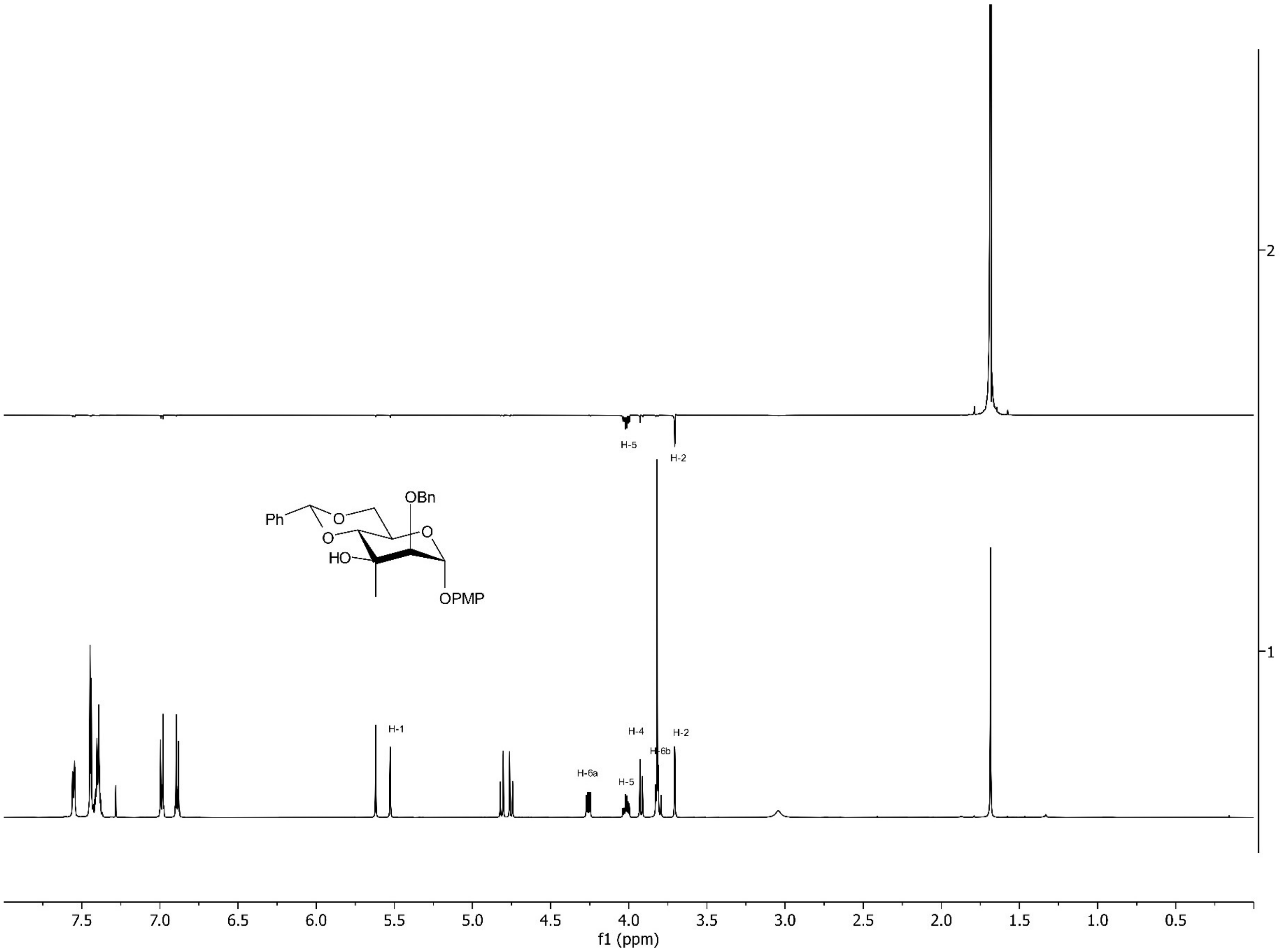

Figure S66. ^{1}H NMR (600 MHz, $CDCl_3$) spectrum of *p*-methoxyphenyl 2-*O*-benzyl-4,6-*O*-benzylidene-3-deoxy-3-*C*-methylene-α-D-*arabino*-hexopyranoside **26**:

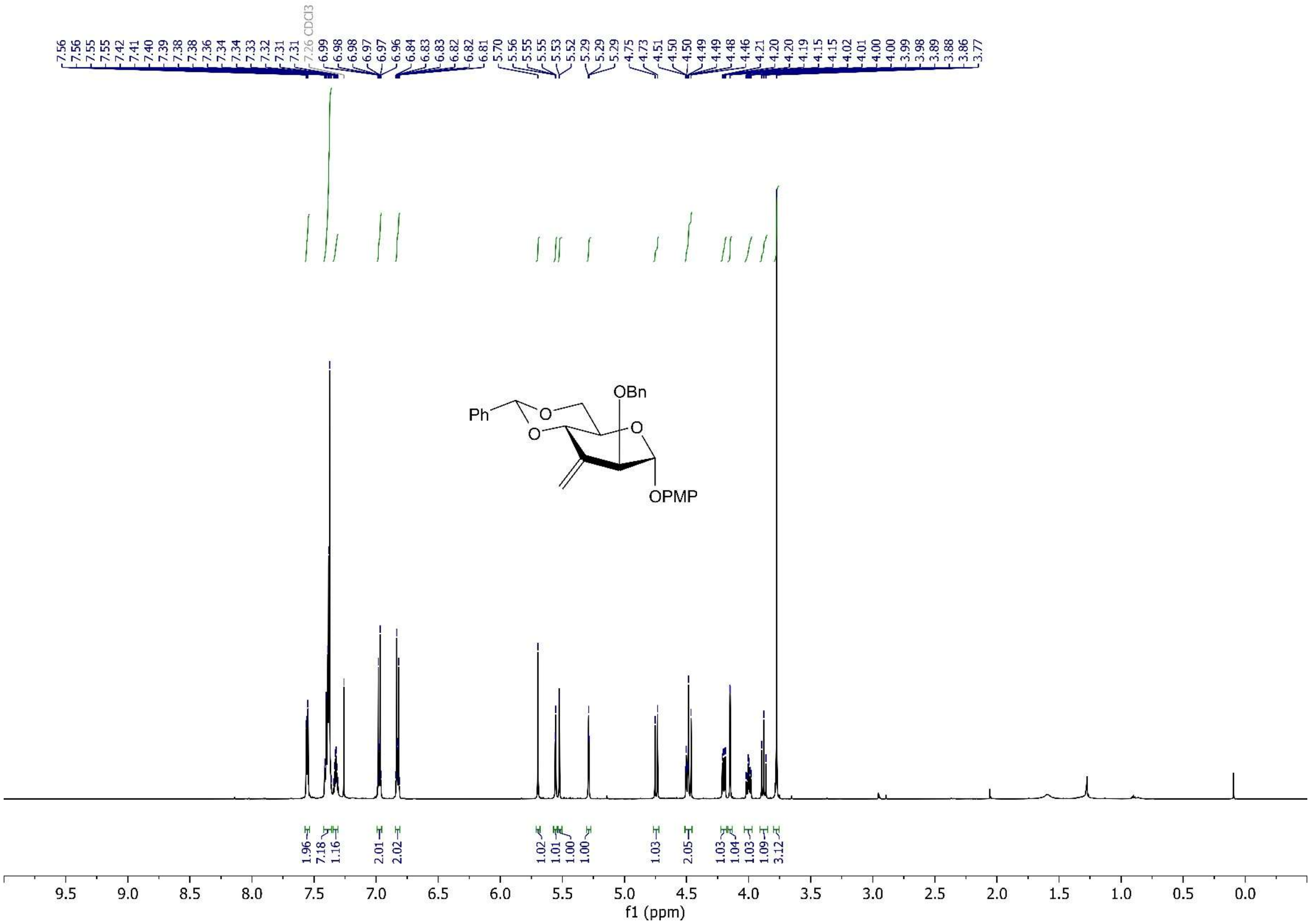

Figure S67. COSY NMR (600 MHz, $CDCl_3$) spectrum of *p*-methoxyphenyl 2-*O*-benzyl-4,6-*O*-benzylidene-3-deoxy-3-*C*-methylene-α-D-*arabino*-hexopyranoside **26**:

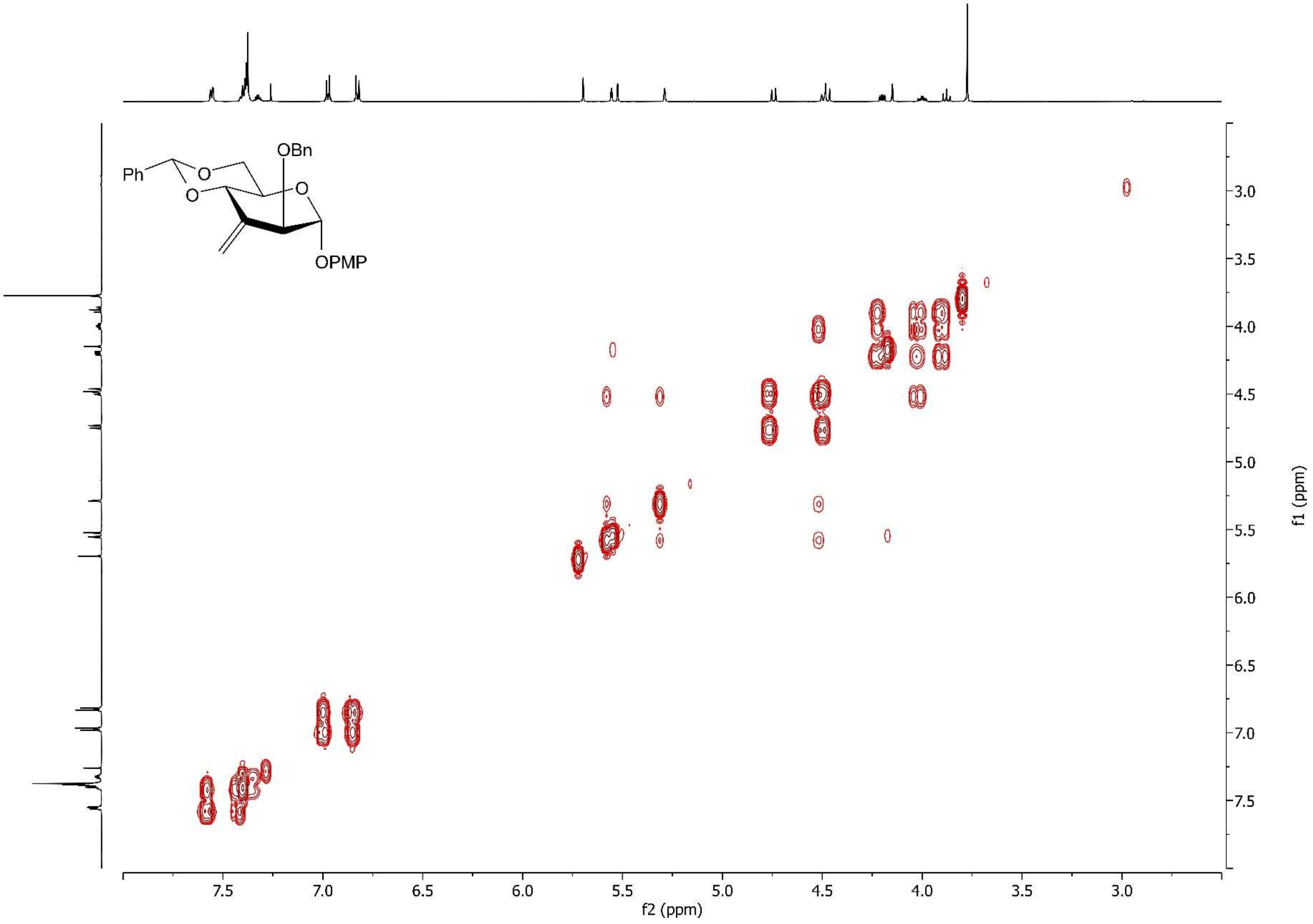

Figure S68. $^{13}C\{H\}$ NMR (151 MHz, $CDCl_3$) spectrum of *p*-methoxyphenyl 2-*O*-benzyl-4,6-*O*-benzylidene-3-deoxy-3-*C*-methylene-α-D-*arabino*-hexopyranoside **26**:

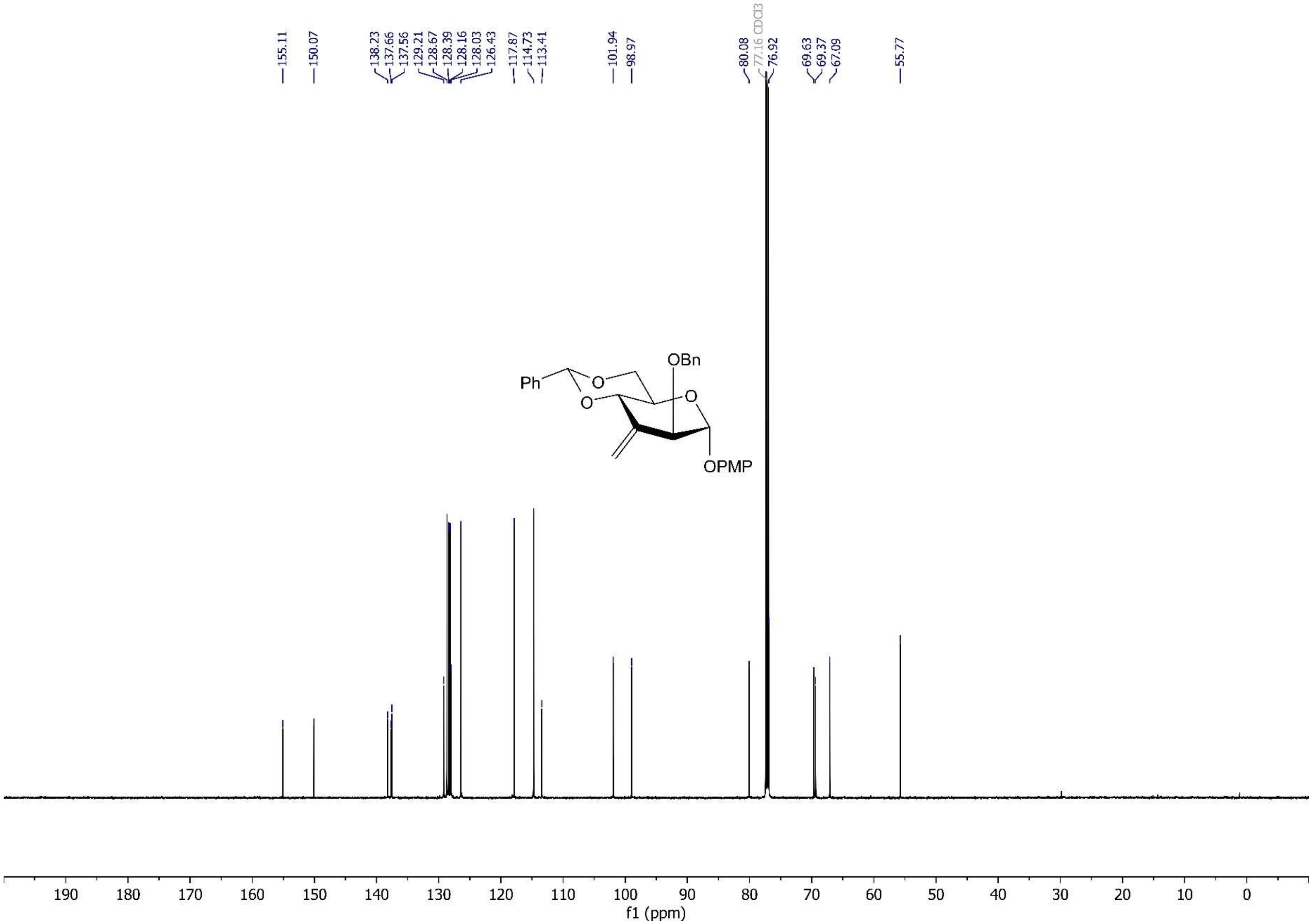

Figure S69. HSQC NMR (600 MHz, $CDCl_3$) spectrum of *p*-methoxyphenyl 2-*O*-benzyl-4,6-*O*-benzylidene-3-deoxy-3-*C*-methylene-α-D-*arabino*-hexopyranoside **26**:

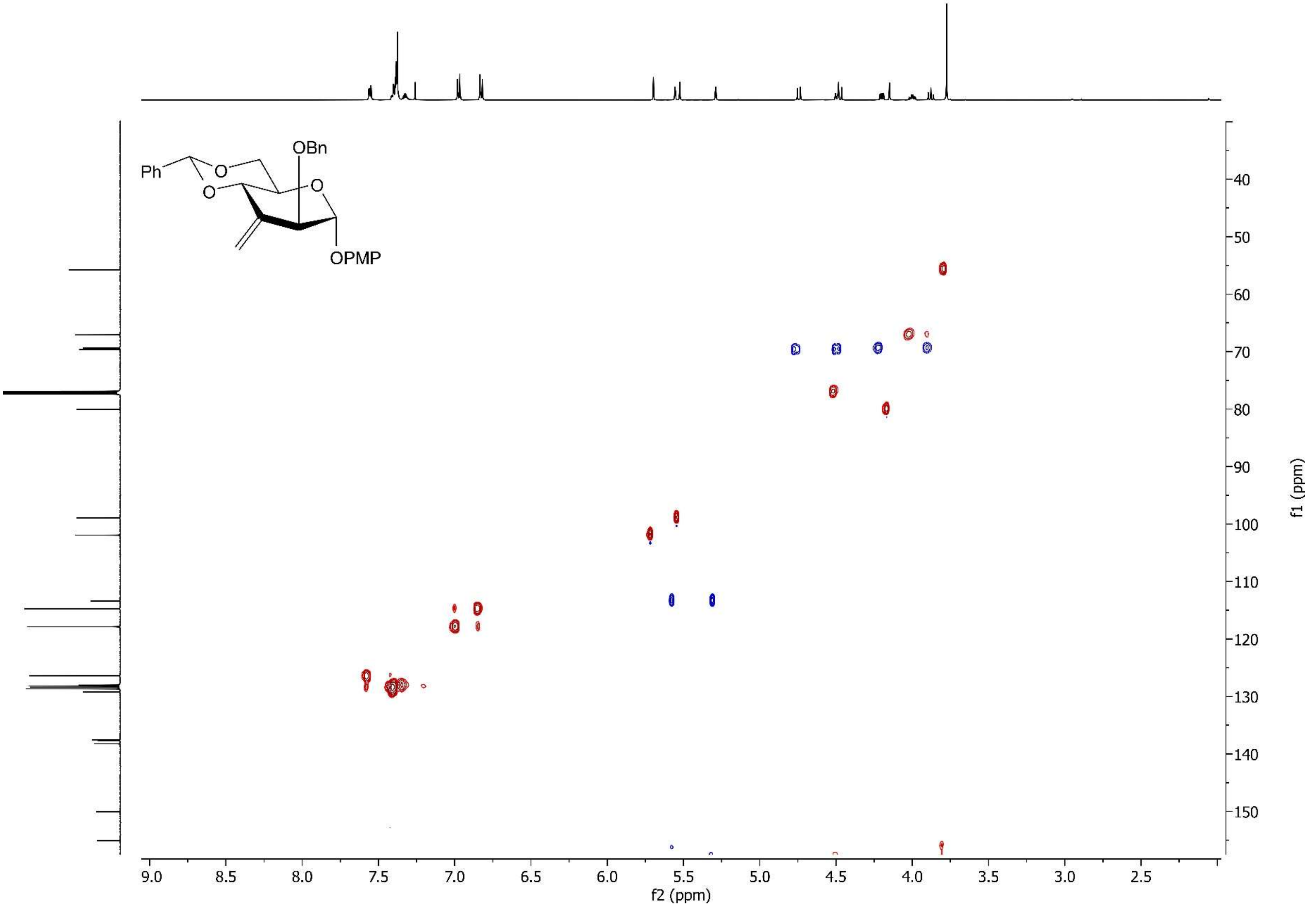

Figure S70. HMBC NMR (600 MHz, $CDCl_3$) spectrum of *p*-methoxyphenyl 2-*O*-benzyl-4,6-*O*-benzylidene-3-deoxy-3-*C*-methylene-α-D-*arabino*-hexopyranoside **26**:

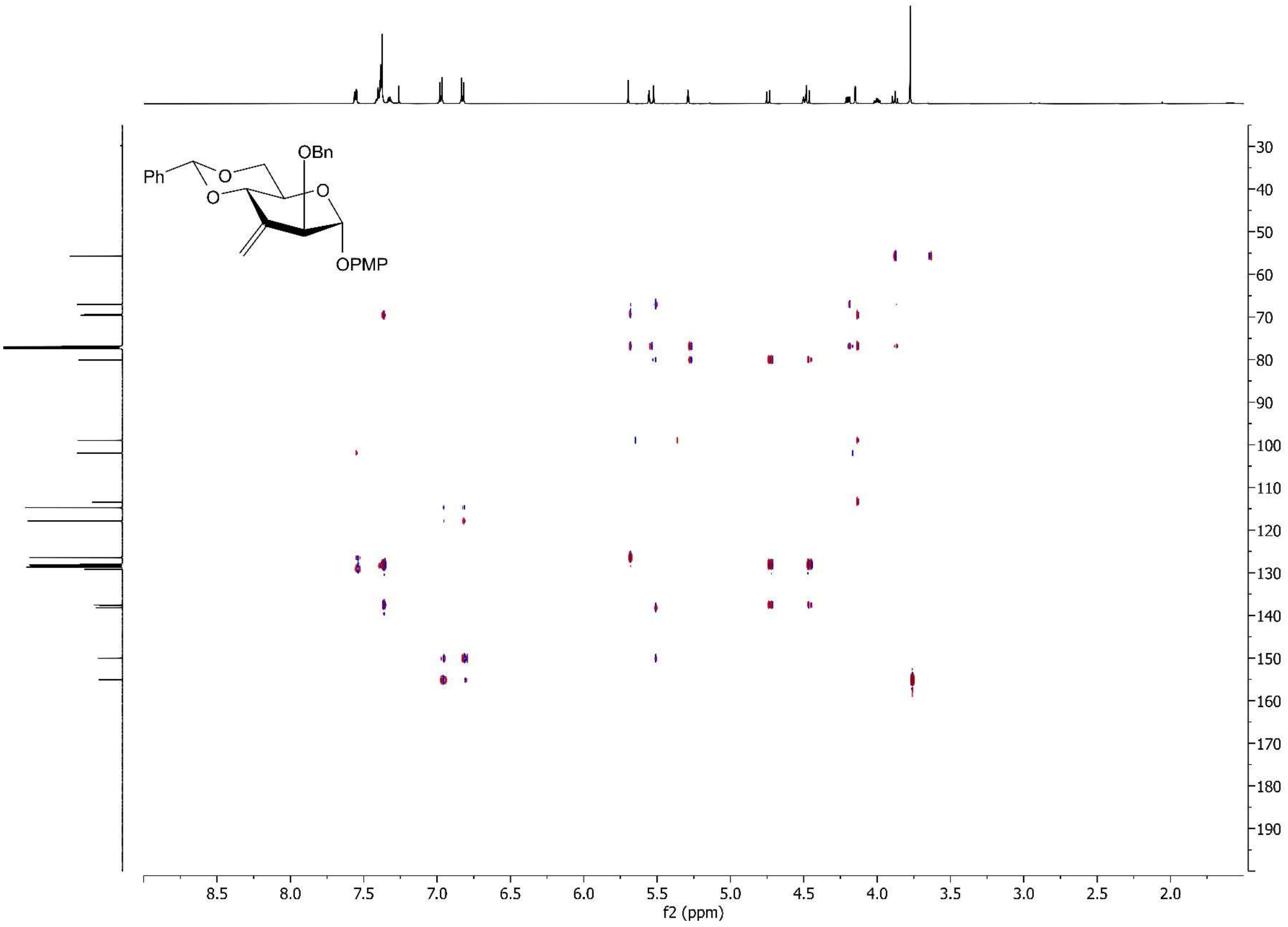

Figure S71. ^{1}H NMR (600 MHz, $CDCl_3$) spectrum of *p*-methoxyphenyl 3,3'-anhydro-2-*O*-benzyl-4,6-*O*-benzylidene-3-*C*-hydroxymethyl-α-D-*manno*-hexopyranoside **27**:

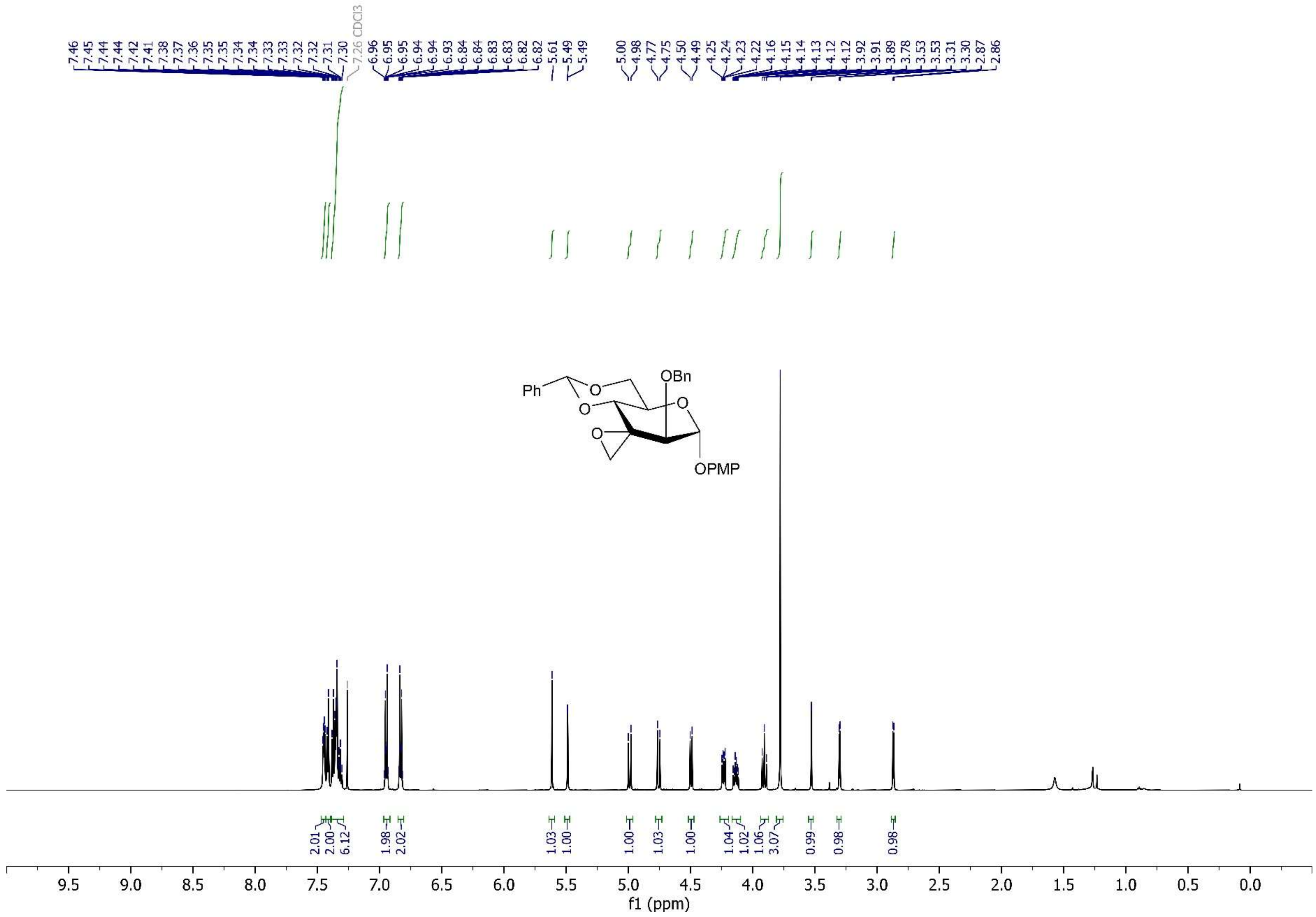

Figure S72. COSY NMR (600 MHz, $CDCl_3$) spectrum of *p*-methoxyphenyl 3,3'-anhydro-2-*O*-benzyl-4,6-*O*-benzylidene-3-*C*-hydroxymethyl-α-D-*manno*-hexopyranoside **27**:

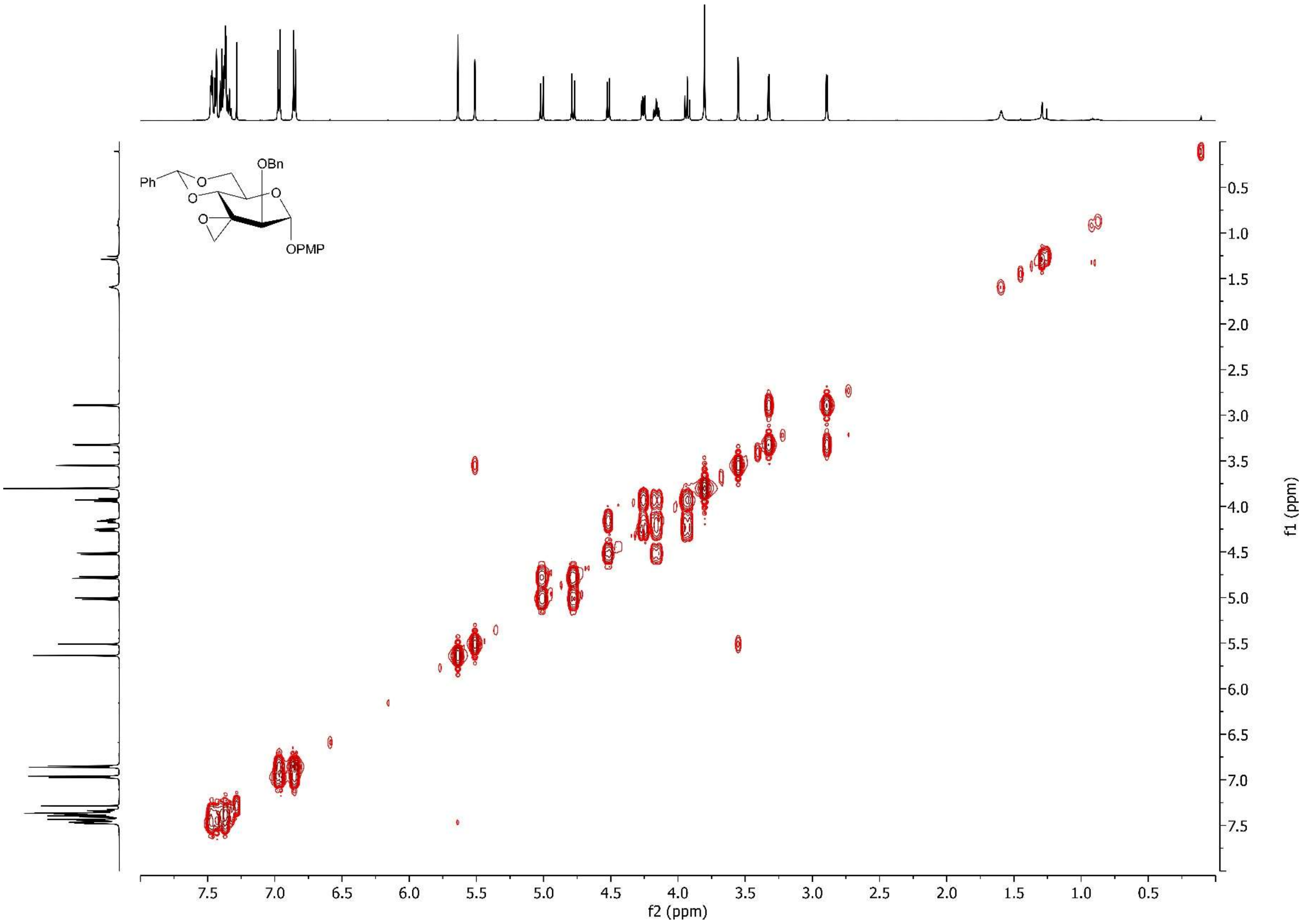

Figure S73. $^{13}C\{H\}$ NMR (151 MHz, $CDCl_3$) spectrum of *p*-methoxyphenyl 3,3’-anhydro-2-*O*-benzyl-4,6-*O*-benzylidene-3-*C*-hydroxymethyl-α-D-*manno*-hexopyranoside **27**:

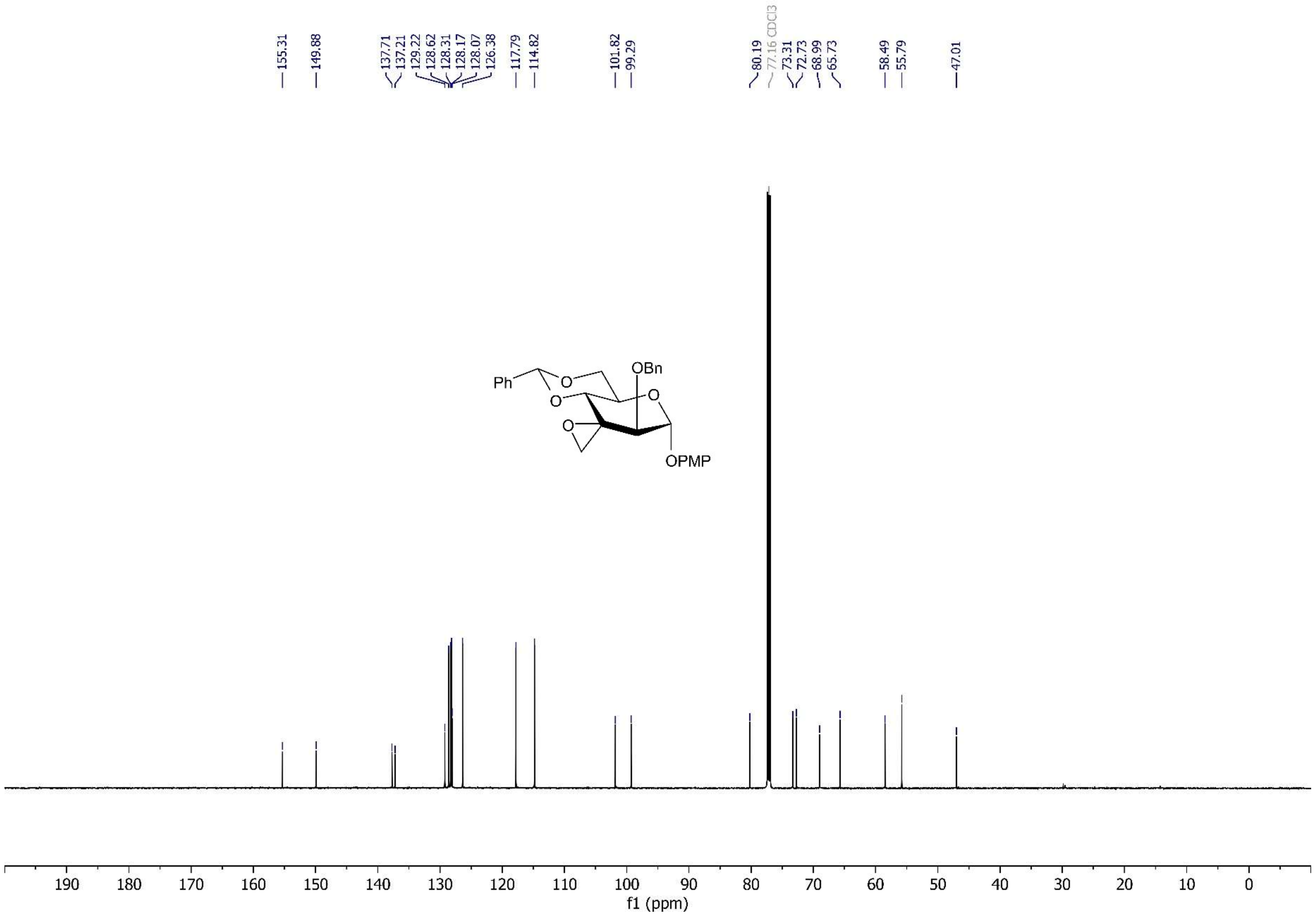

Figure S74. HSQC NMR (600 MHz, $CDCl_3$) spectrum of *p*-methoxyphenyl 3,3'-anhydro-2-*O*-benzyl-4,6-*O*-benzylidene-3-*C*-hydroxymethyl-α-D-*manno*-hexopyranoside **27**:

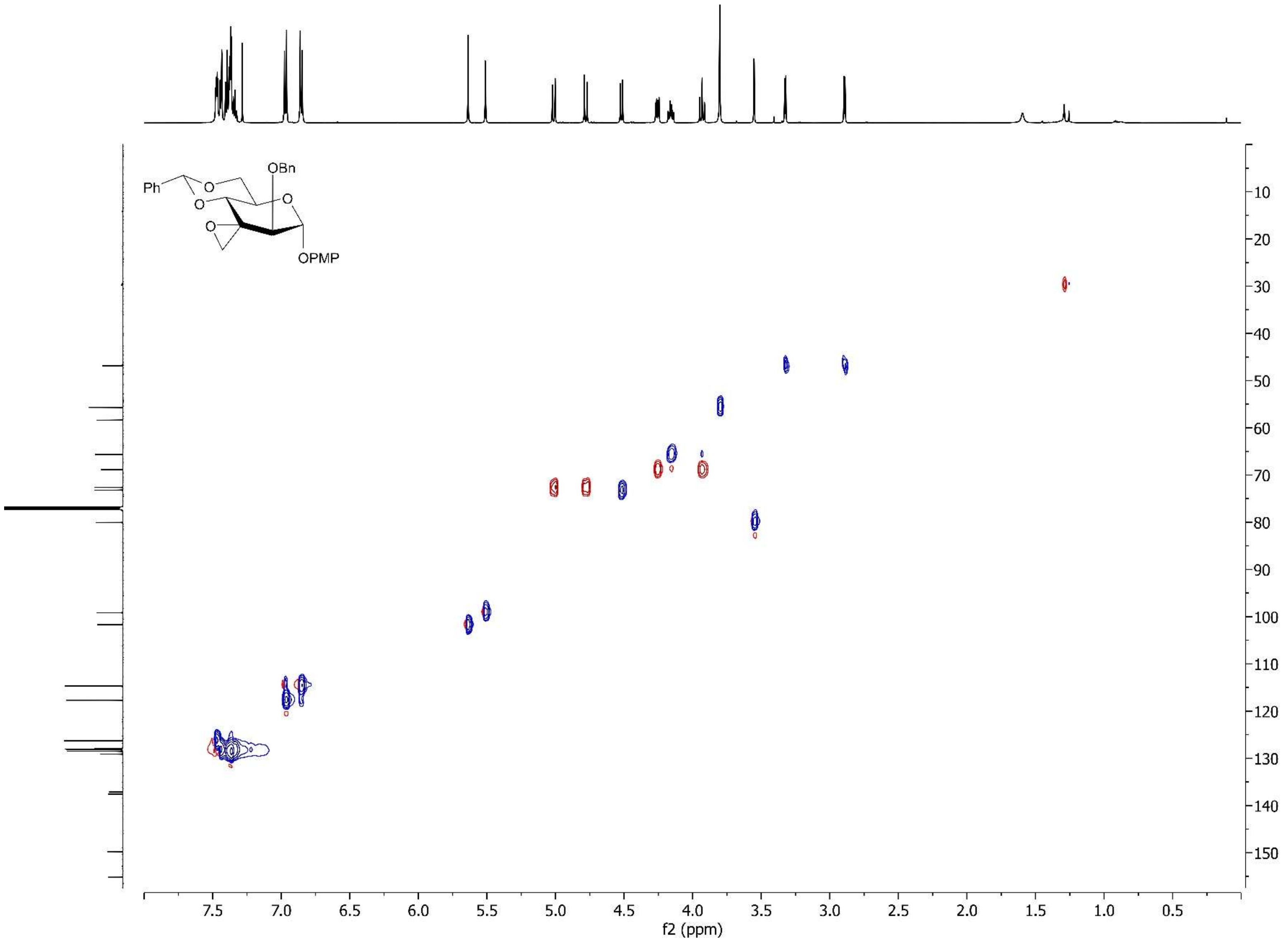

Figure S75. HMBC NMR (600 MHz, $CDCl_3$) spectrum of *p*-methoxyphenyl 3,3'-anhydro-2-*O*-benzyl-4,6-*O*-benzylidene-3-*C*-hydroxymethyl-α-D-*manno*-hexopyranoside **27**:

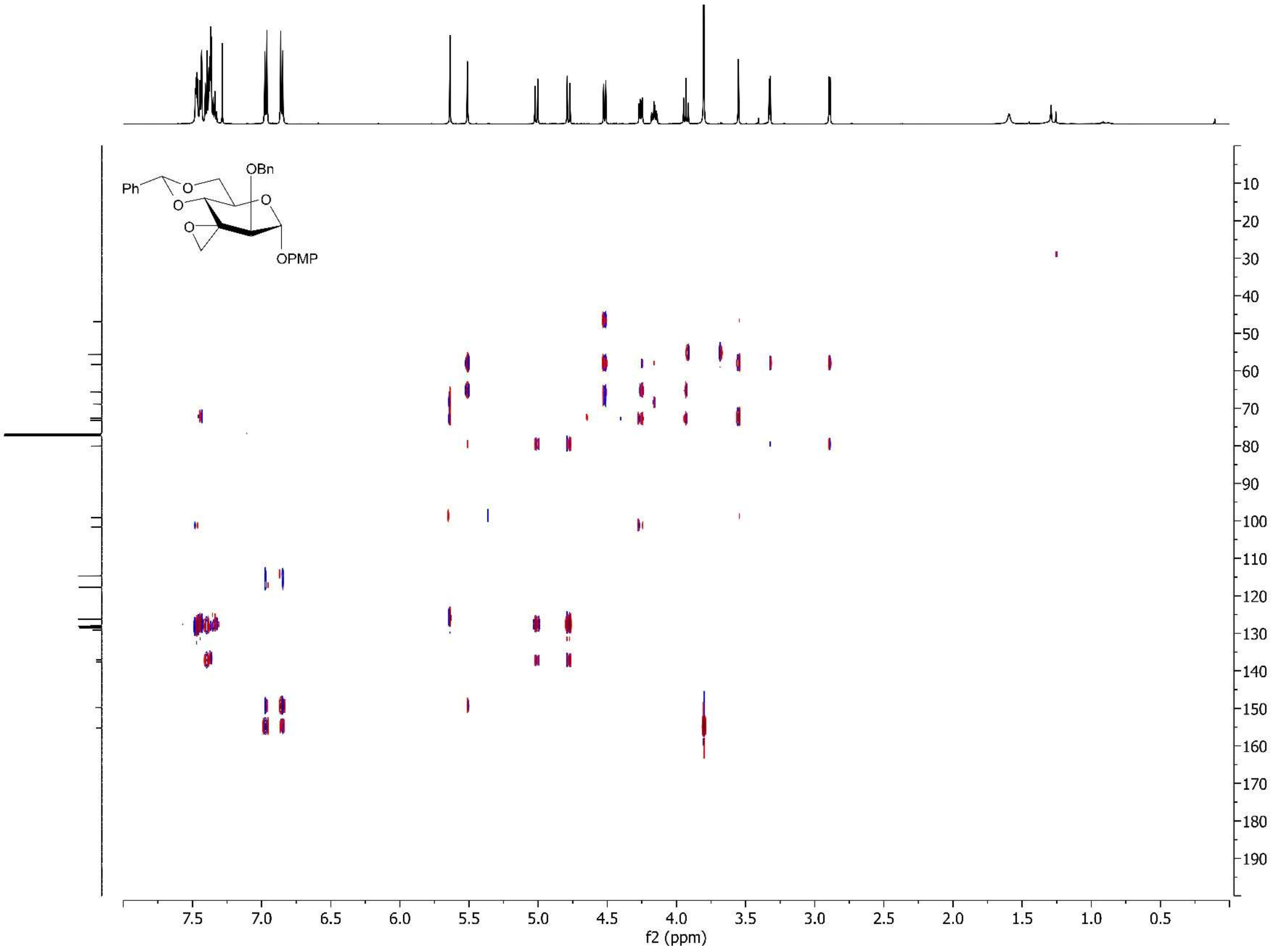

Figure S76. ^{1}H (NOE) NMR (600 MHz, $CDCl_3$) spectrum of *p*-methoxyphenyl 3,3'-anhydro-2-*O*-benzyl-4,6-*O*-benzylidene-3-*C*-hydroxymethyl-α-D-*manno*-hexopyranoside **27**:

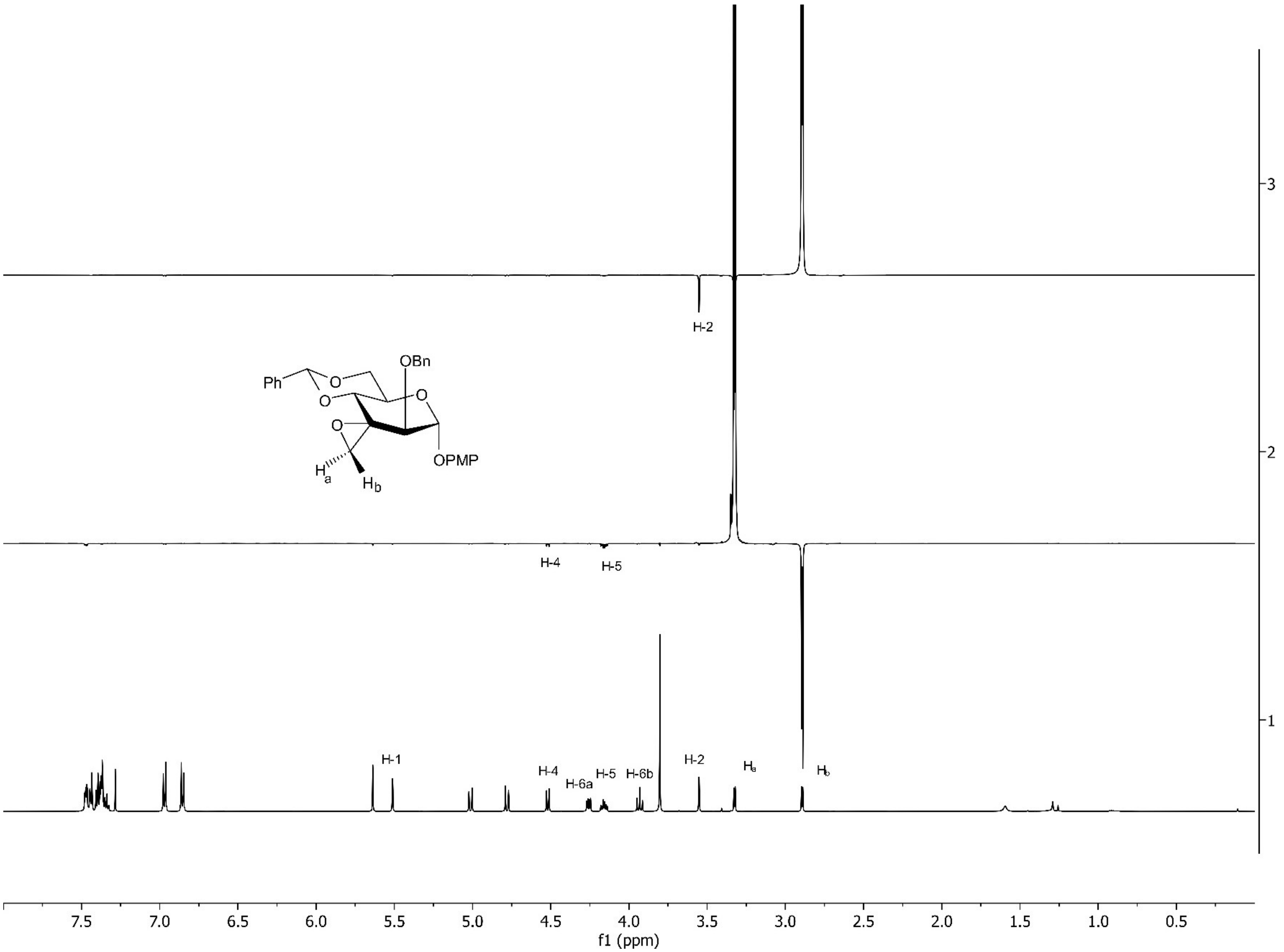

Figure S77. ^{1}H NMR (600 MHz, $CDCl_3$) spectrum of *p*-methoxyphenyl 3,3'-anhydro-2-*O*-benzyl-4,6-*O*-benzylidene-3-*C*-hydroxymethyl-α-D-*allo*-hexopyranoside **28**:

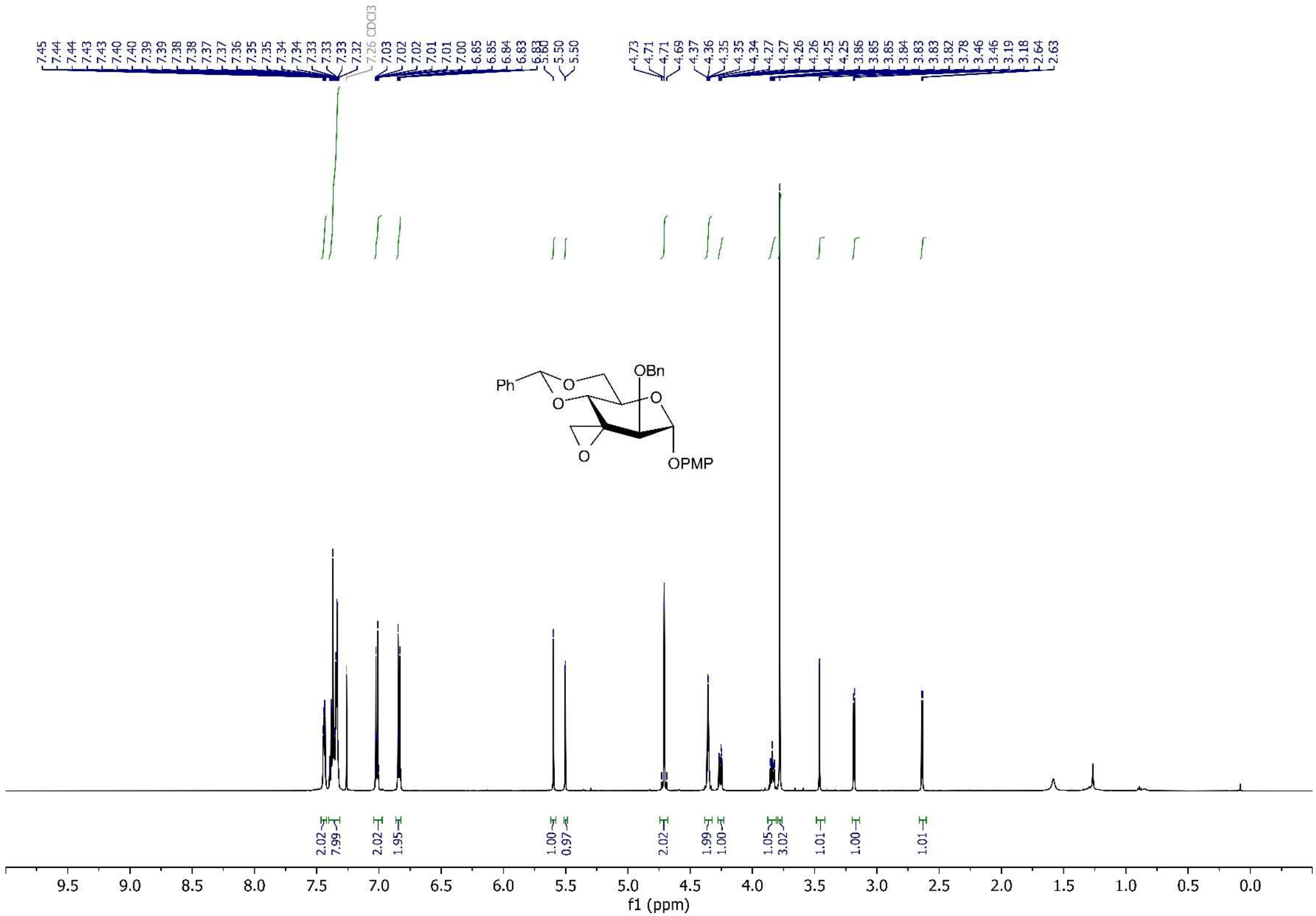

Figure S78. COSY NMR (600 MHz, $CDCl_3$) spectrum of *p*-methoxyphenyl 3,3'-anhydro-2-*O*-benzyl-4,6-*O*-benzylidene-3-*C*-hydroxymethyl-α-D-*allo*-hexopyranoside **28**:

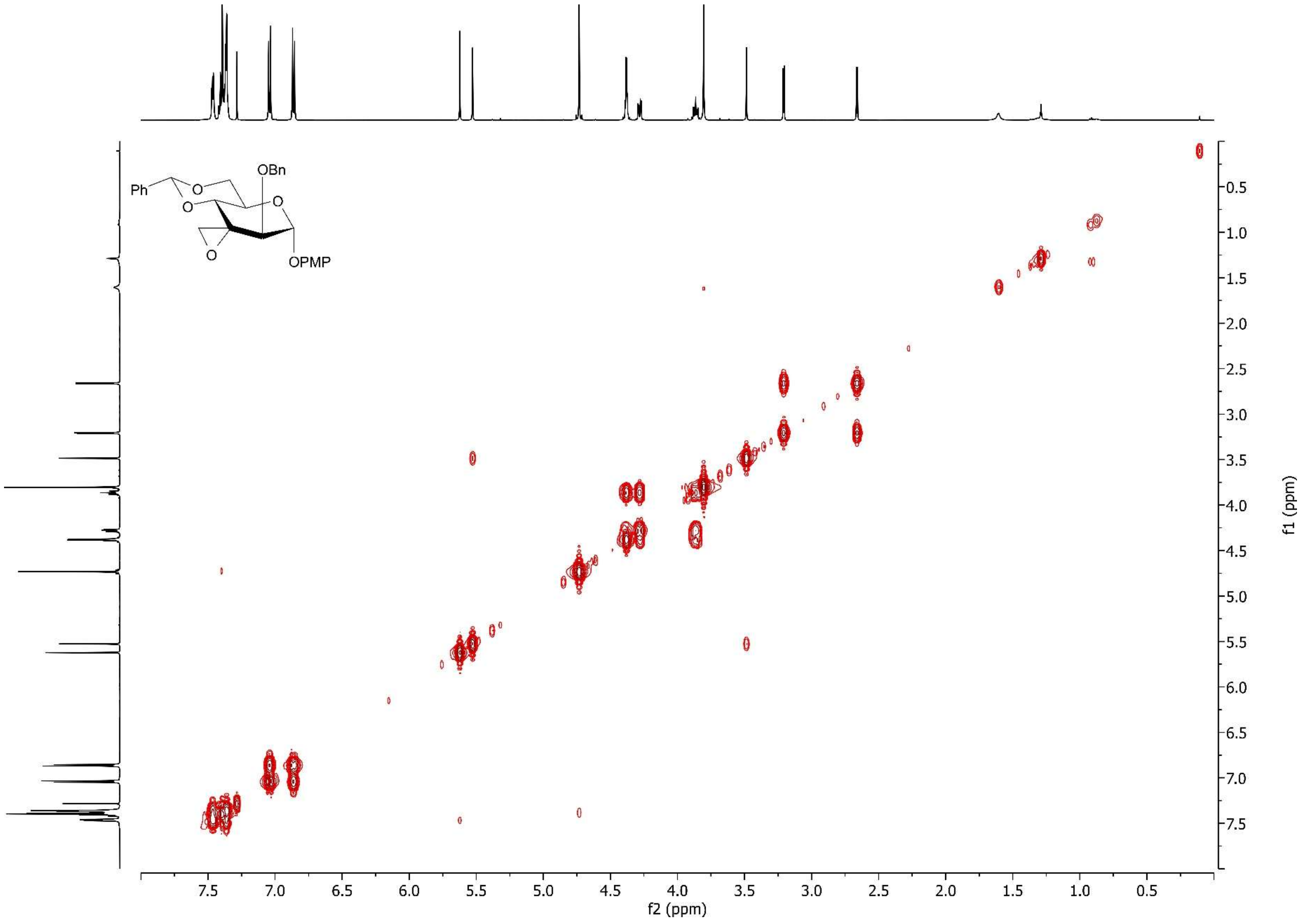

Figure S79. $^{13}C\{H\}$ NMR (151 MHz, $CDCl_3$) spectrum of *p*-methoxyphenyl 3,3'-anhydro-2-*O*-benzyl-4,6-*O*-benzylidene-3-*C*-hydroxymethyl-α-D-*allo*-hexopyranoside **28**:

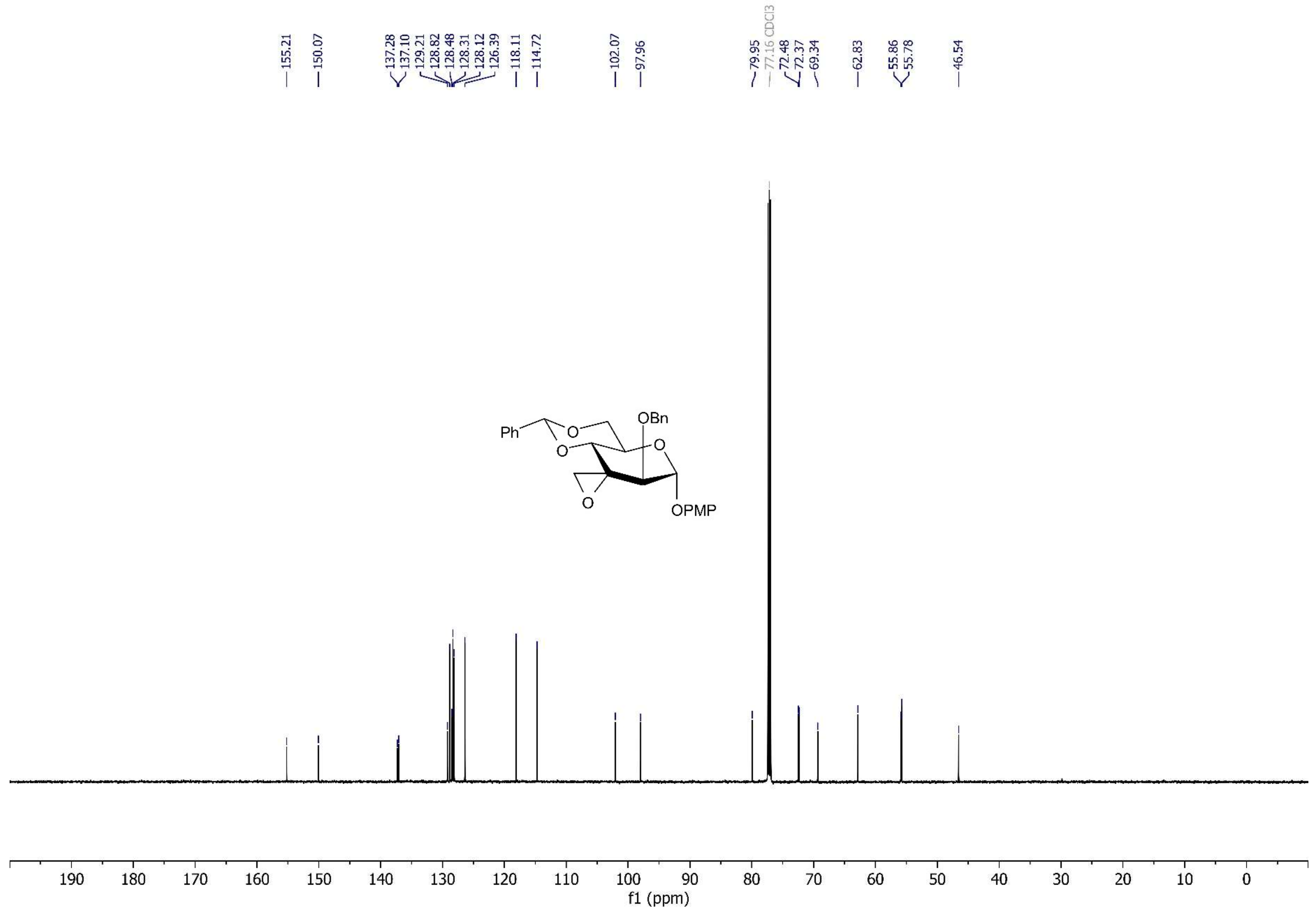

Figure S80. HSQC NMR (600 MHz, $CDCl_3$) spectrum of *p*-methoxyphenyl 3,3'-anhydro-2-*O*-benzyl-4,6-*O*-benzylidene-3-*C*-hydroxymethyl-α-D-*allo*-hexopyranoside **28**:

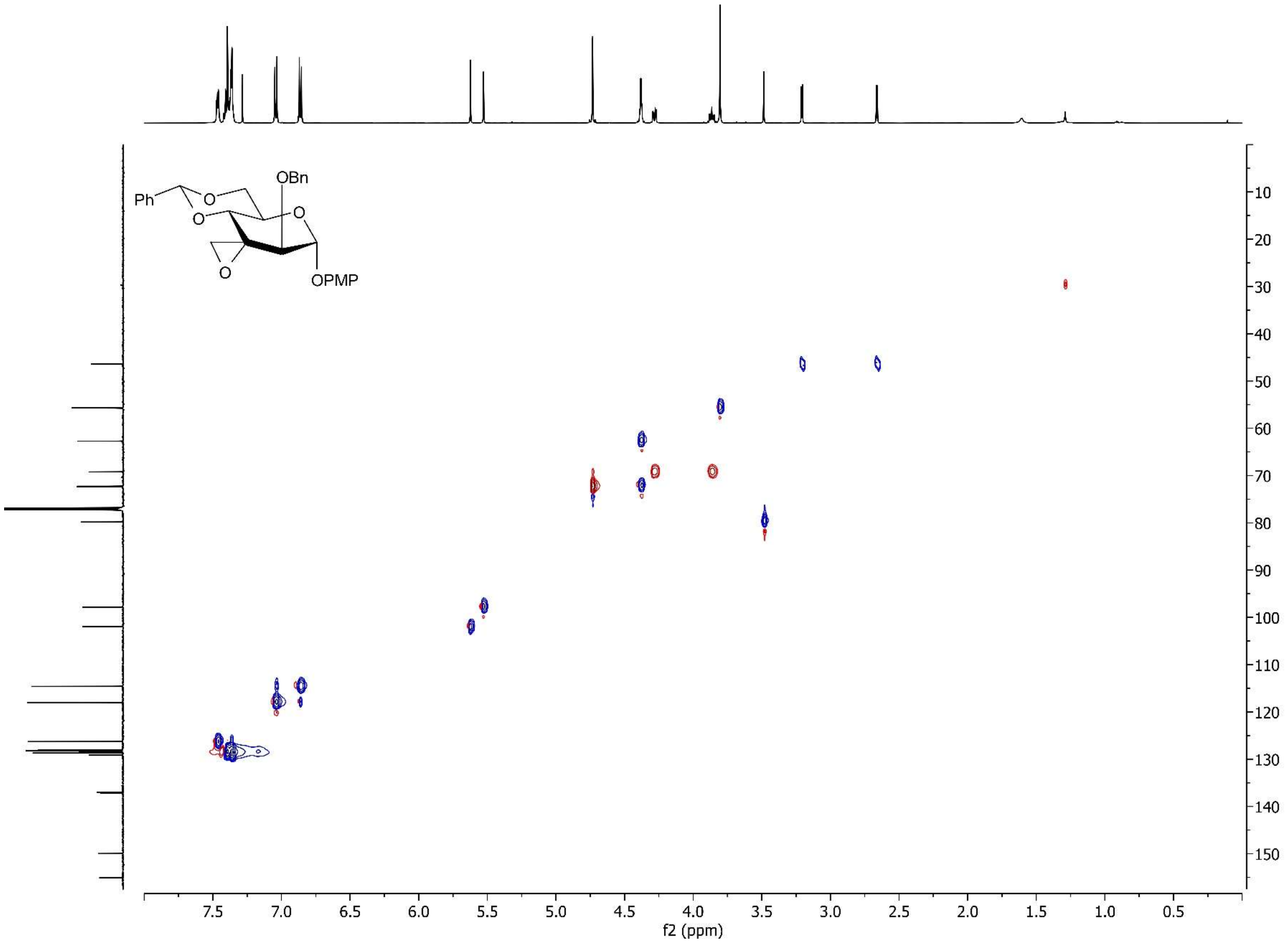

Figure S81. HMBC NMR (600 MHz, $CDCl_3$) spectrum of *p*-methoxyphenyl 3,3'-anhydro-2-*O*-benzyl-4,6-*O*-benzylidene-3-*C*-hydroxymethyl-α-D-*allo*-hexopyranoside **28**:

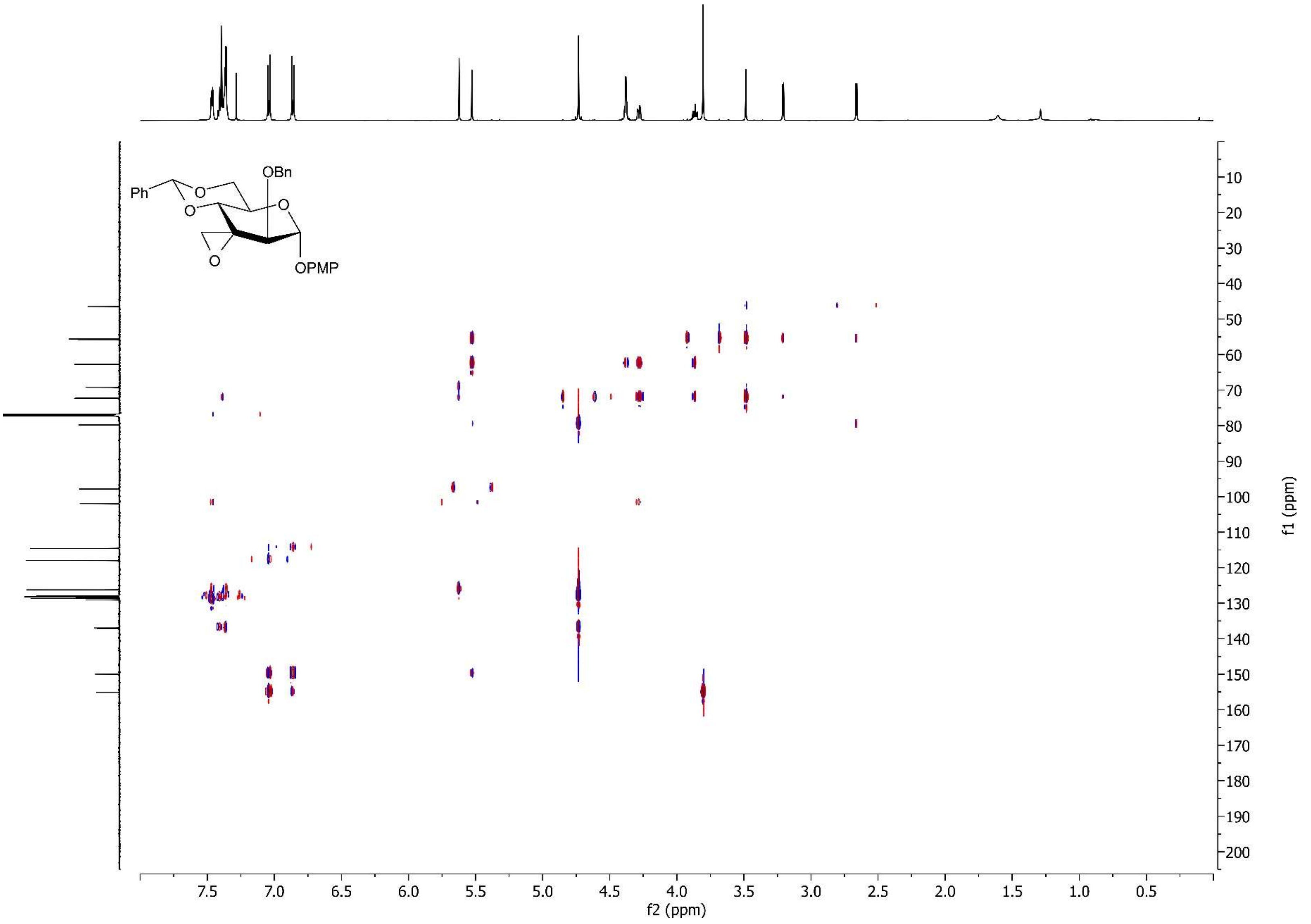

Figure S82. ^{1}H (NOE) NMR (600 MHz, $CDCl_3$) spectrum of *p*-methoxyphenyl 3,3'-anhydro-2-*O*-benzyl-4,6-*O*-benzylidene-3-*C*-hydroxymethyl-α-D-*allo*-hexopyranoside **28**:

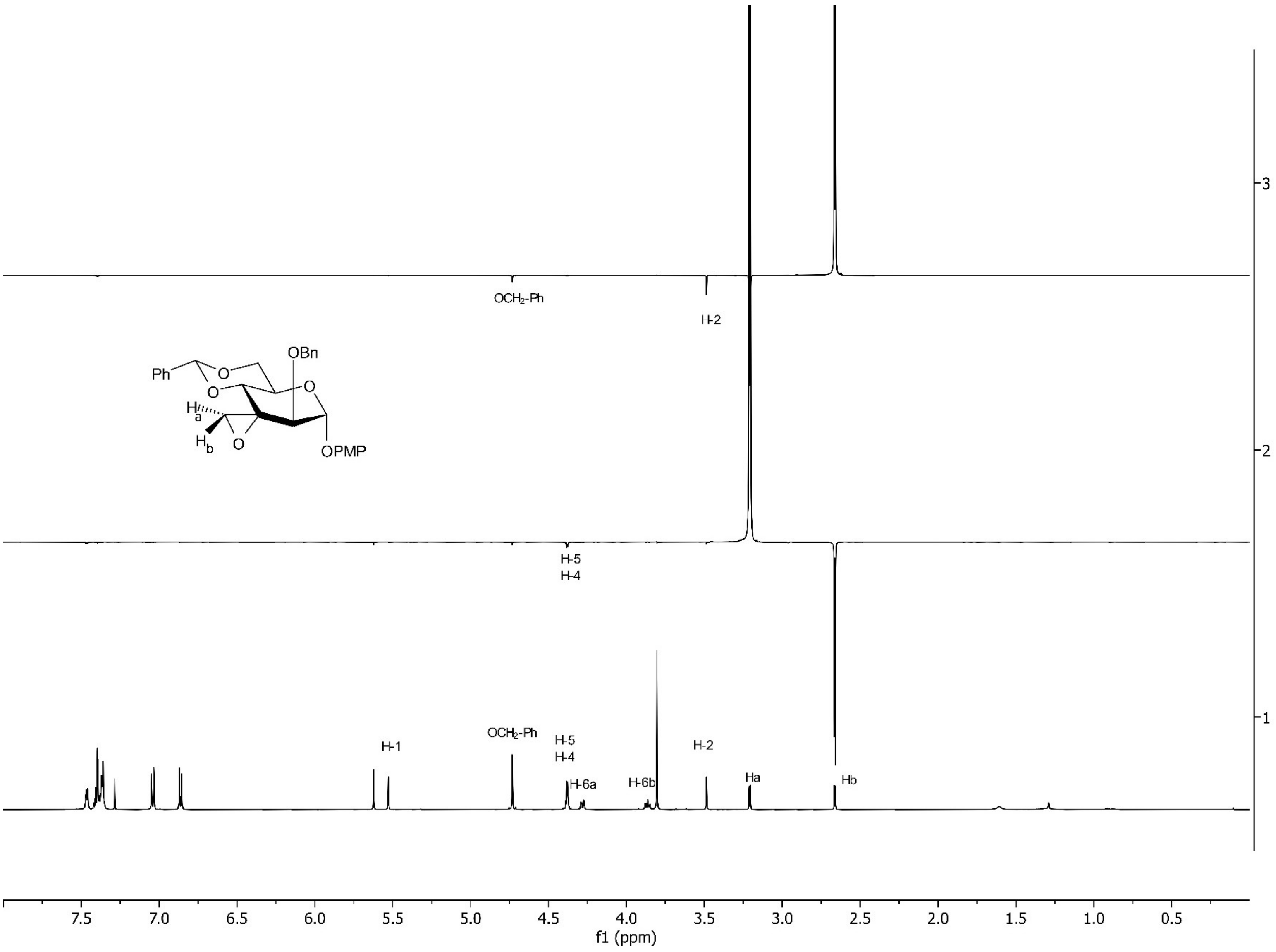

Figure S83. Comparison of ^{1}H NMR (600 MHz, $CDCl_3$) spectra of *p*-methoxyphenyl 3,3'-anhydro-2-*O*-benzyl-4,6-*O*-benzylidene-3-*C*-hydroxymethyl-α-D-*manno*-hexopyranoside **27** and *p*-methoxyphenyl 3,3'-anhydro-2-*O*-benzyl-4,6-*O*-benzylidene-3-*C*-hydroxymethyl-α-D-*allo*-hexopyranoside **28:**

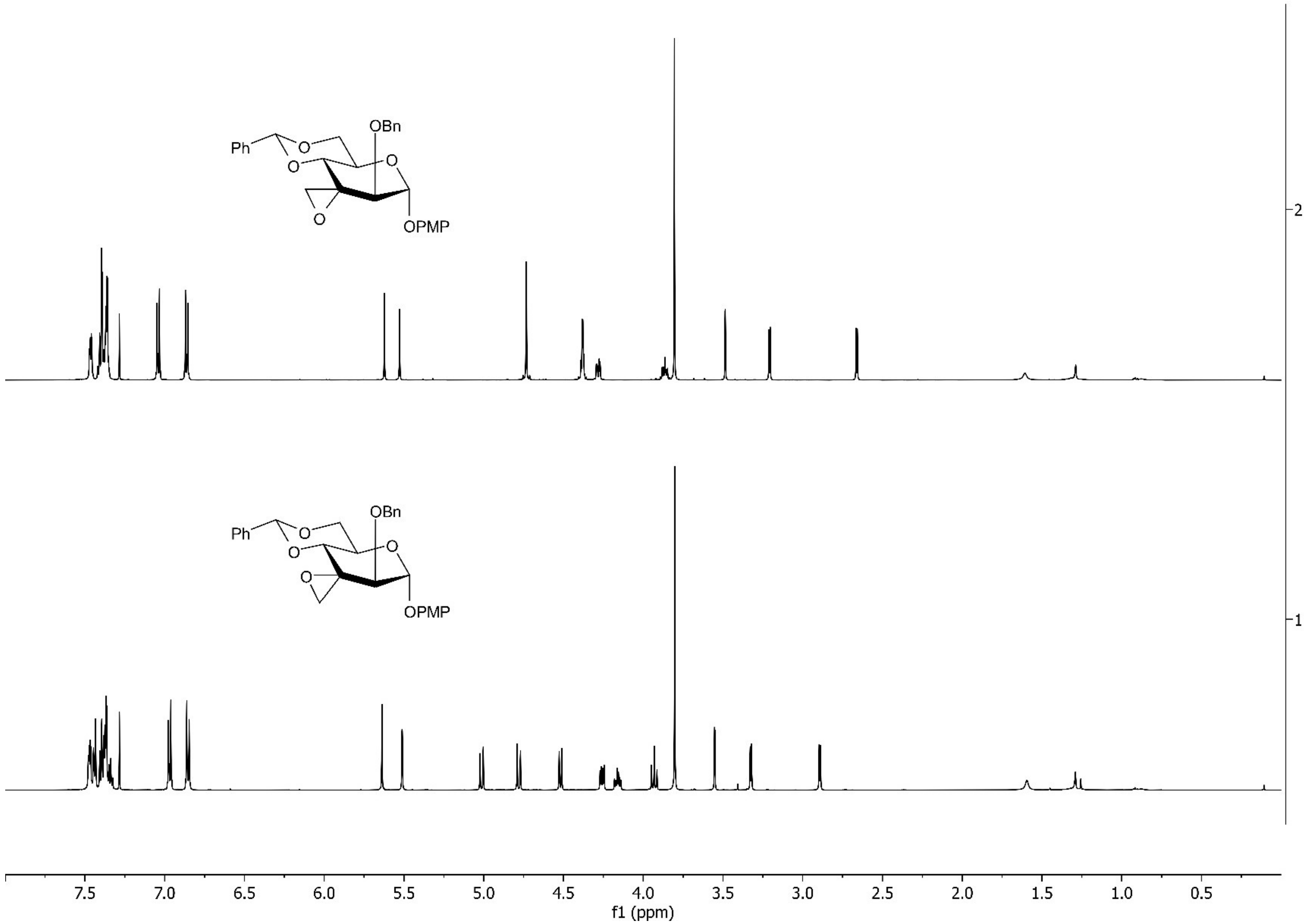

Figure S84. Comparison of $^{13}C\{H\}$ NMR (151 MHz, $CDCl_3$) spectra of *p*-methoxyphenyl 3,3'-anhydro-2-*O*-benzyl-4,6-*O*-benzylidene-3-*C*-hydroxymethyl-α-D-*manno*-hexopyranoside **27** and *p*-methoxyphenyl 3,3'-anhydro-2-*O*-benzyl-4,6-*O*-benzylidene-3-*C*-hydroxymethyl-α-D-*allo*-hexopyranoside **28**:

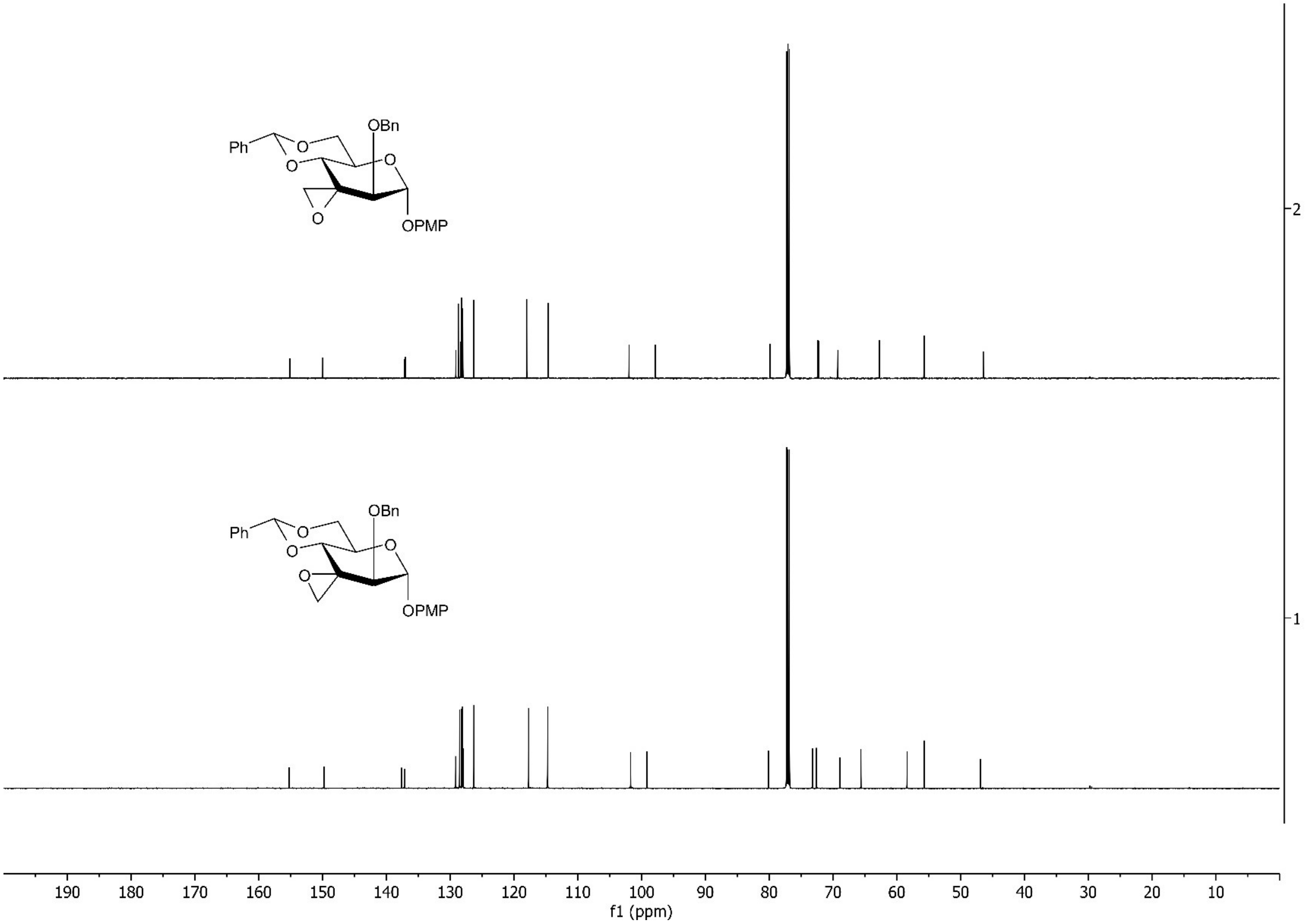

Figure S85. ^{1}H NMR (600 MHz, $CDCl_3$) spectrum of *p*-methoxyphenyl 2-*O*-benzoyl-4,6-*O*-benzylidene-3-*O*-*tert*-butyldimethylsilyl-3-*C*-methyl-α-D-mannopyranoside **29**:

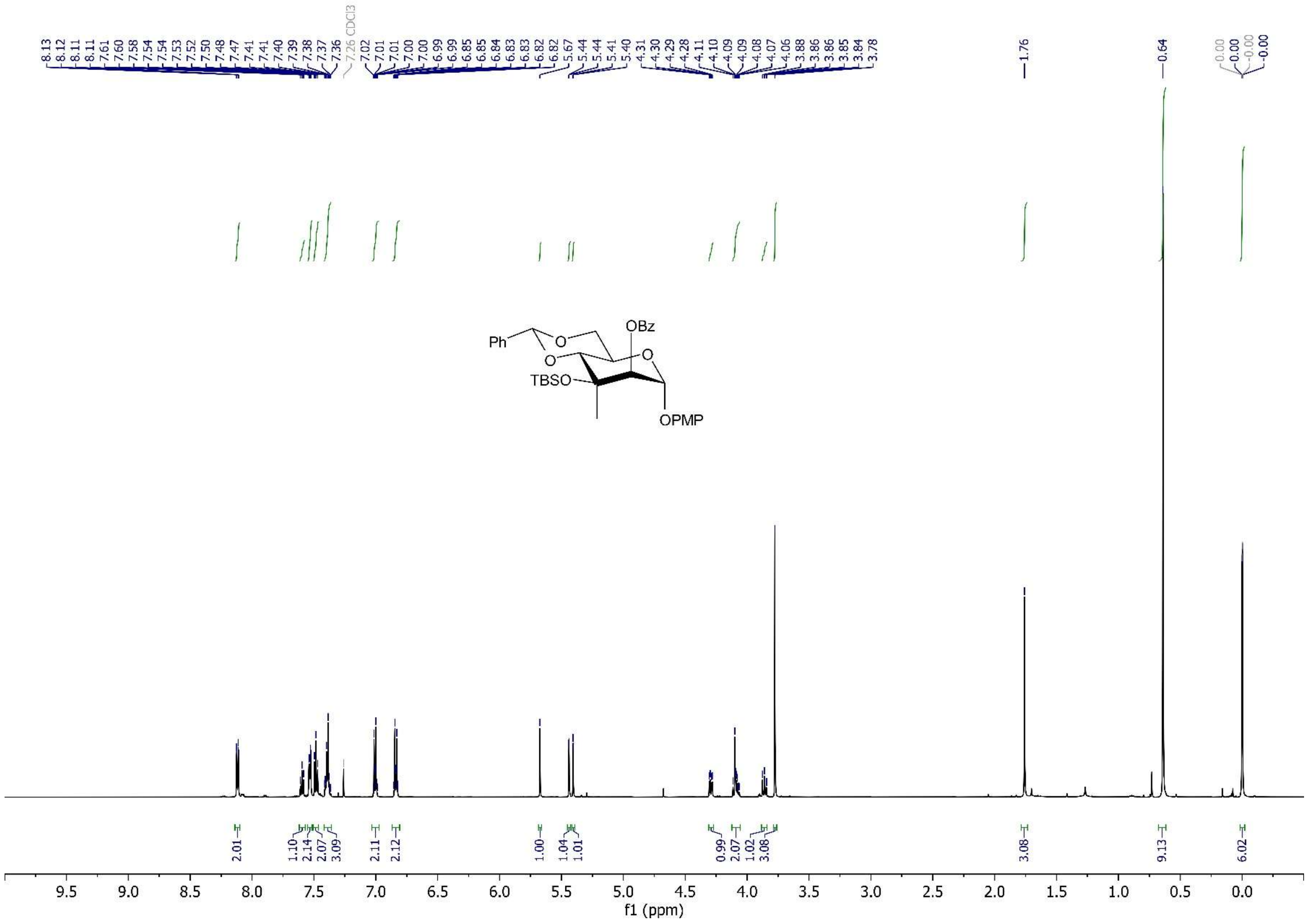

Figure S86. COSY NMR (600 MHz, $CDCl_3$) spectrum of *p*-methoxyphenyl 2-*O*-benzoyl-4,6-*O*-benzylidene-3-*O*-*tert*-butyldimethylsilyl-3-*C*-methyl-α-D-mannopyranoside **29**:

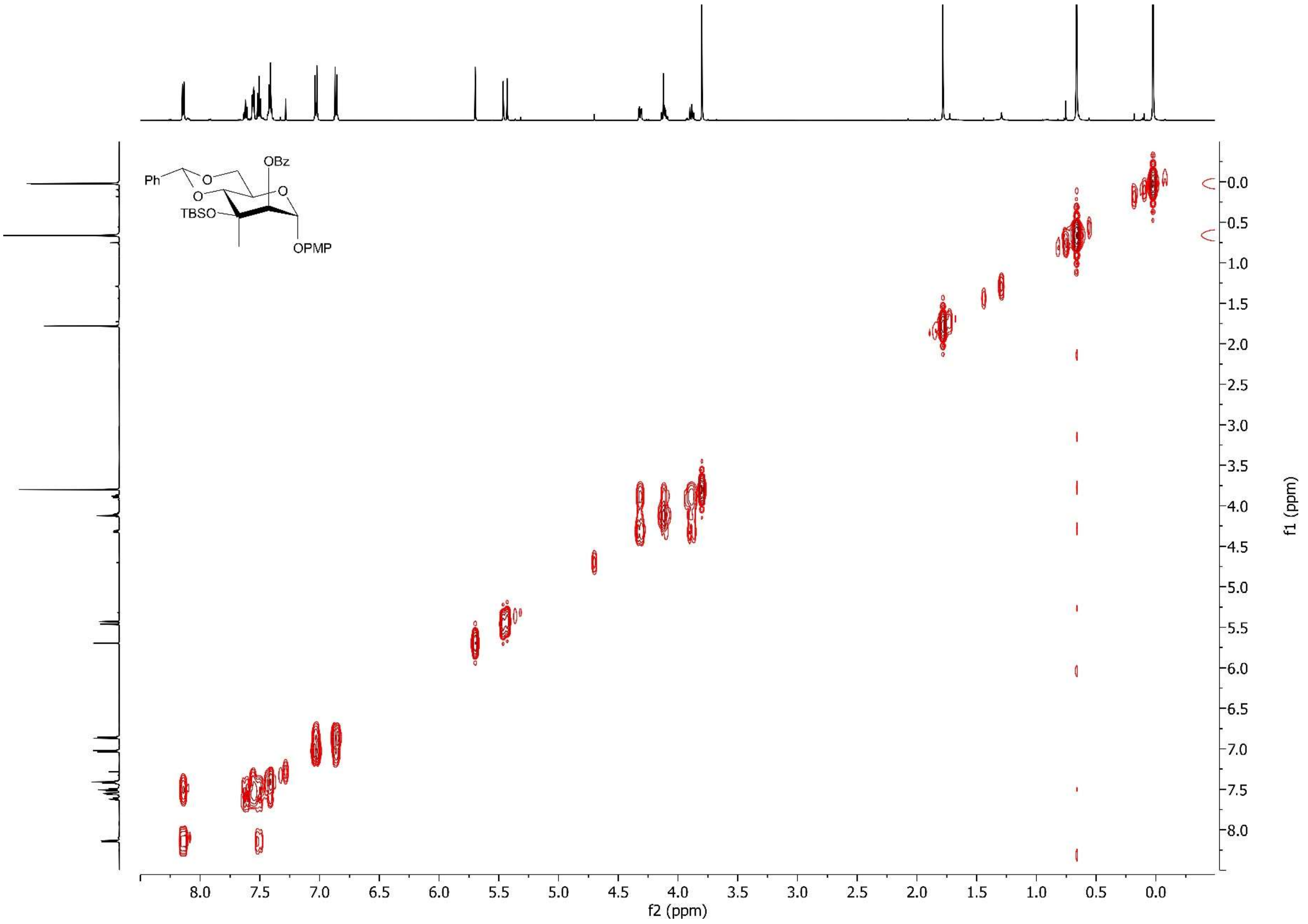

Figure S87. $^{13}C\{H\}$ NMR (151 MHz, $CDCl_3$) spectrum of *p*-methoxyphenyl 2-*O*-benzoyl-4,6-*O*-benzylidene-3-*O*-*tert*-butyldimethylsilyl-3-*C*-methyl-α-D-mannopyranoside **29**:

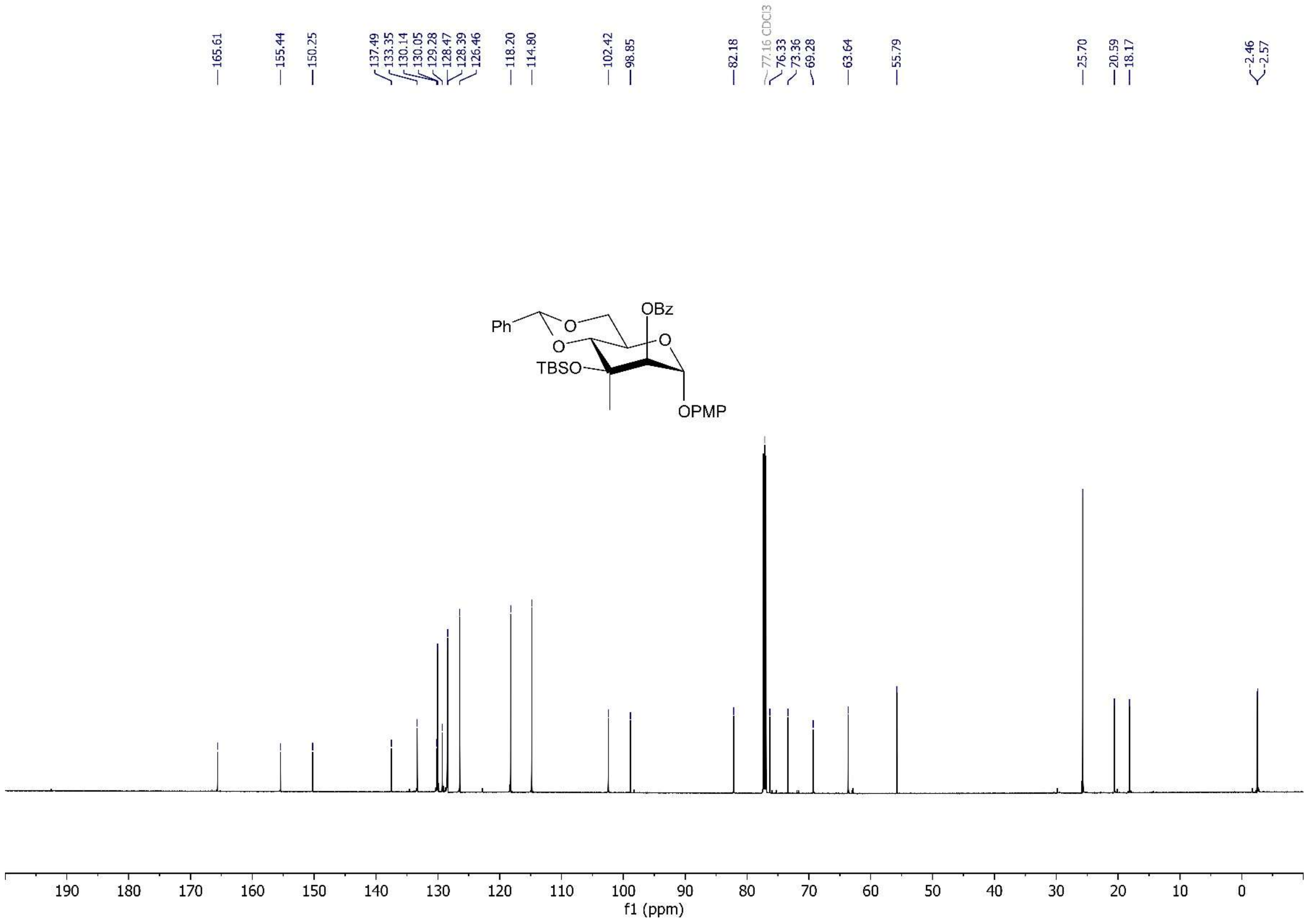

Figure S88. HSQC NMR (600 MHz, $CDCl_3$) spectrum of *p*-methoxyphenyl 2-*O*-benzoyl-4,6-*O*-benzylidene-3-*O*-*tert*-butyldimethylsilyl-3-*C*-methyl-α-D-mannopyranoside **29**:

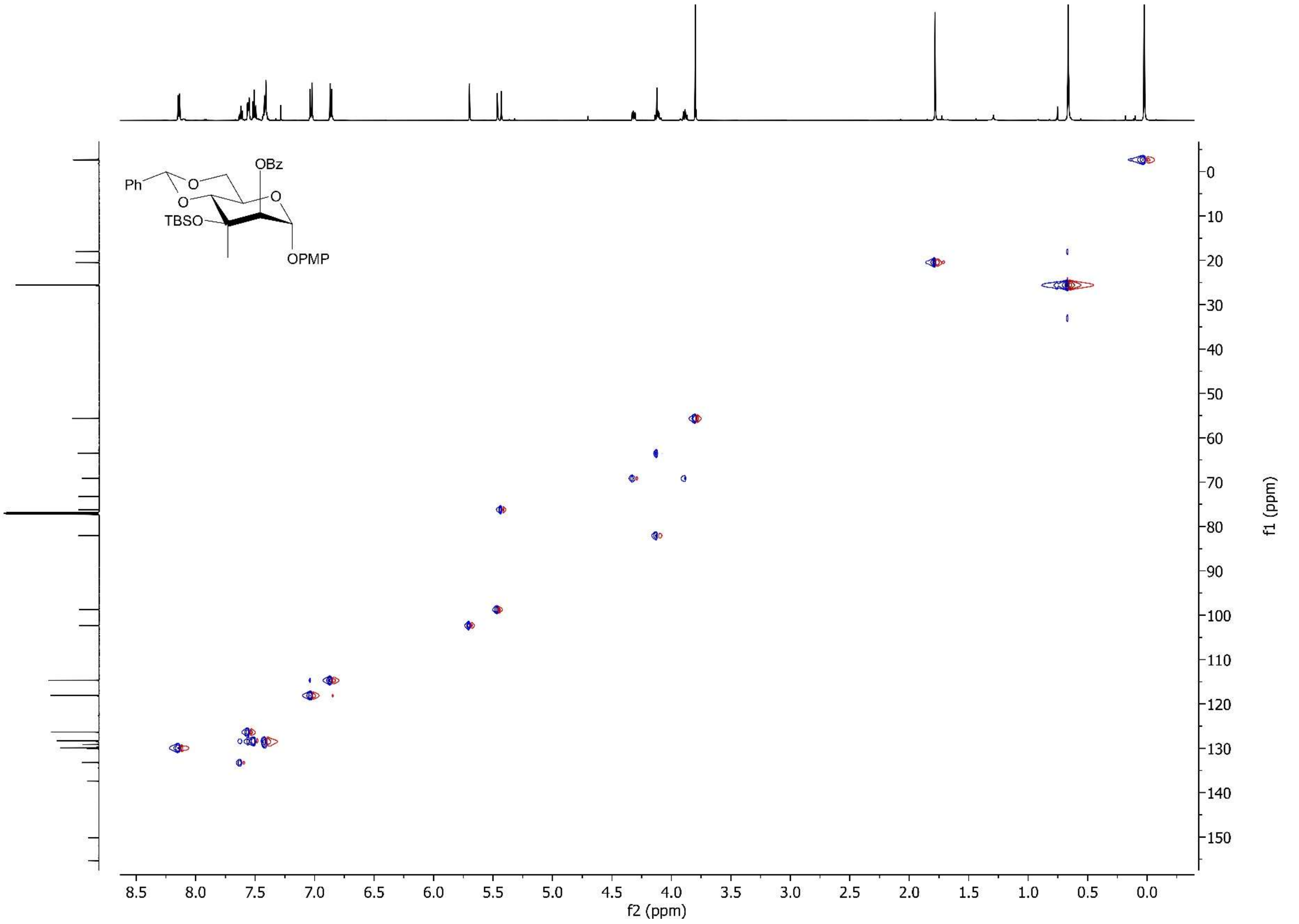

Figure S89. HMBC NMR (600 MHz, $CDCl_3$) spectrum of *p*-methoxyphenyl 2-*O*-benzoyl-4,6-*O*-benzylidene-3-*O*-*tert*-butyldimethylsilyl-3-*C*-methyl-α-D-mannopyranoside **29**:

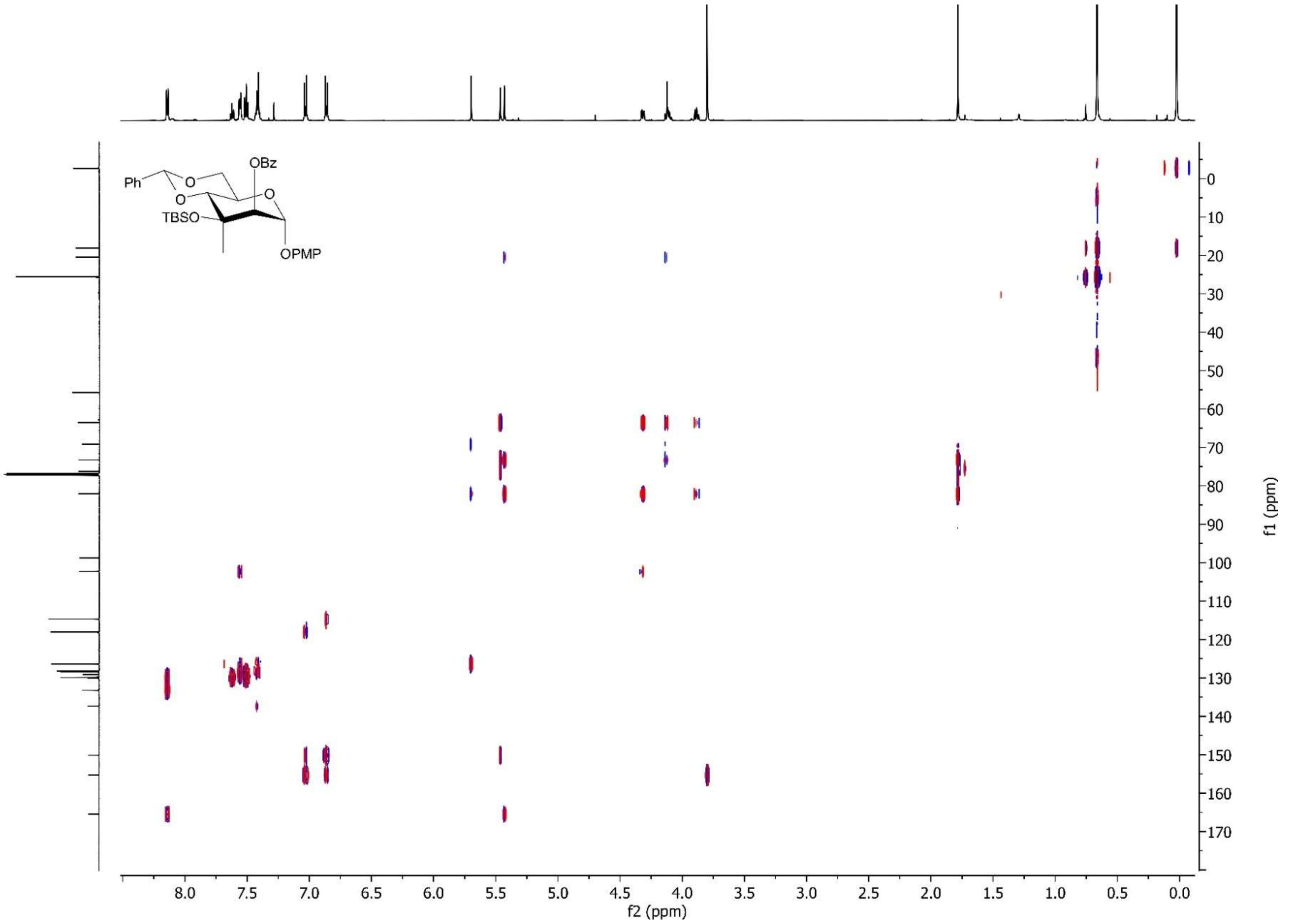

Figure S90. ^{1}H NMR (600 MHz, $CDCl_3$) spectrum of *p*-methoxyphenyl 3-*O*-(benzoyl-α-^{13}C)-2-*O*-benzyl-4,6-*O*-benzylidene-3-*C*-methyl-α-D-mannopyranoside **30**:

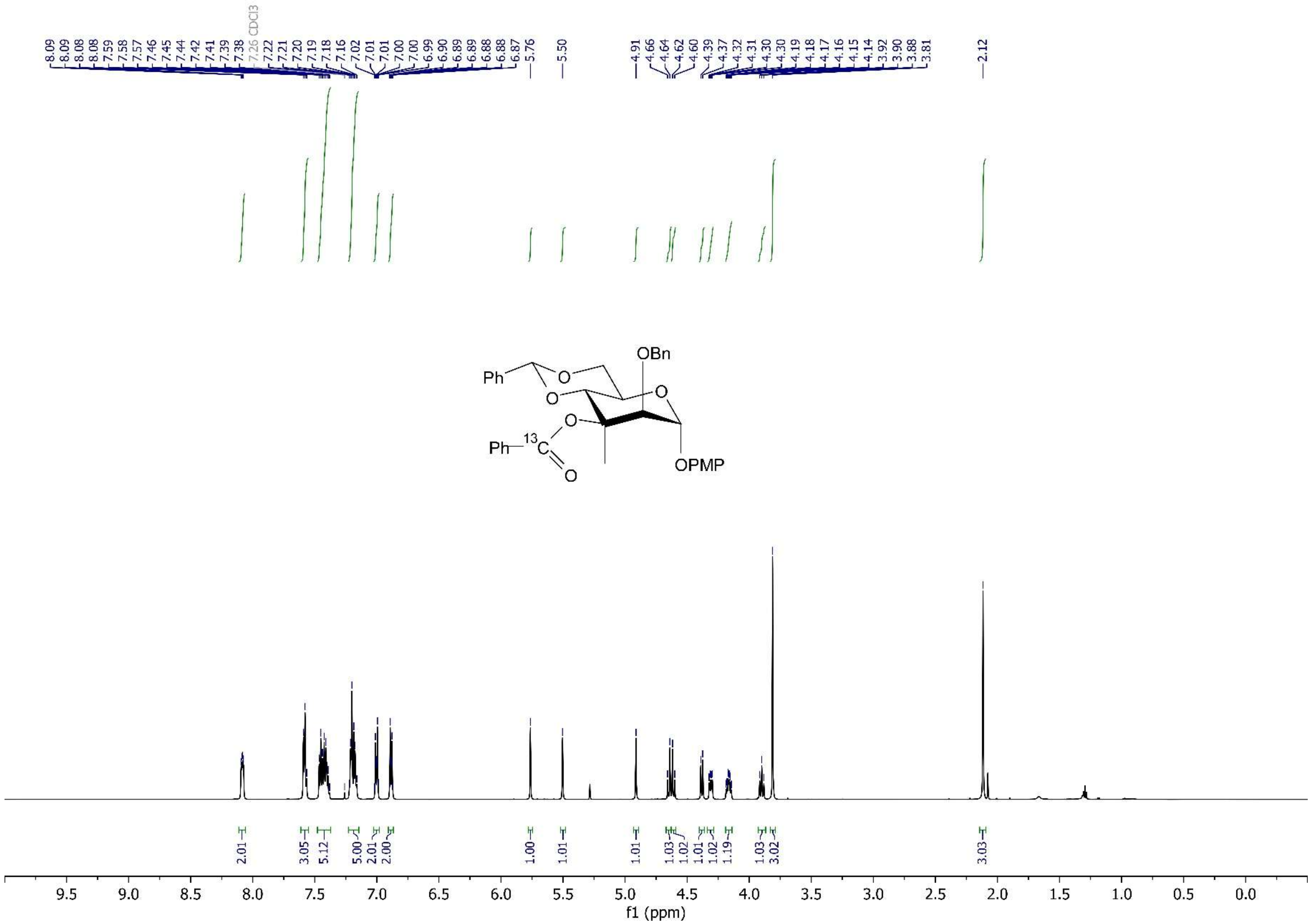

Figure S91. COSY NMR (600 MHz, $CDCl_3$) spectrum of *p*-methoxyphenyl 3-*O*-(benzoyl-α-^{13}C)-2-*O*-benzyl-4,6-*O*-benzylidene-3-*C*-methyl-α-D-mannopyranoside **30**:

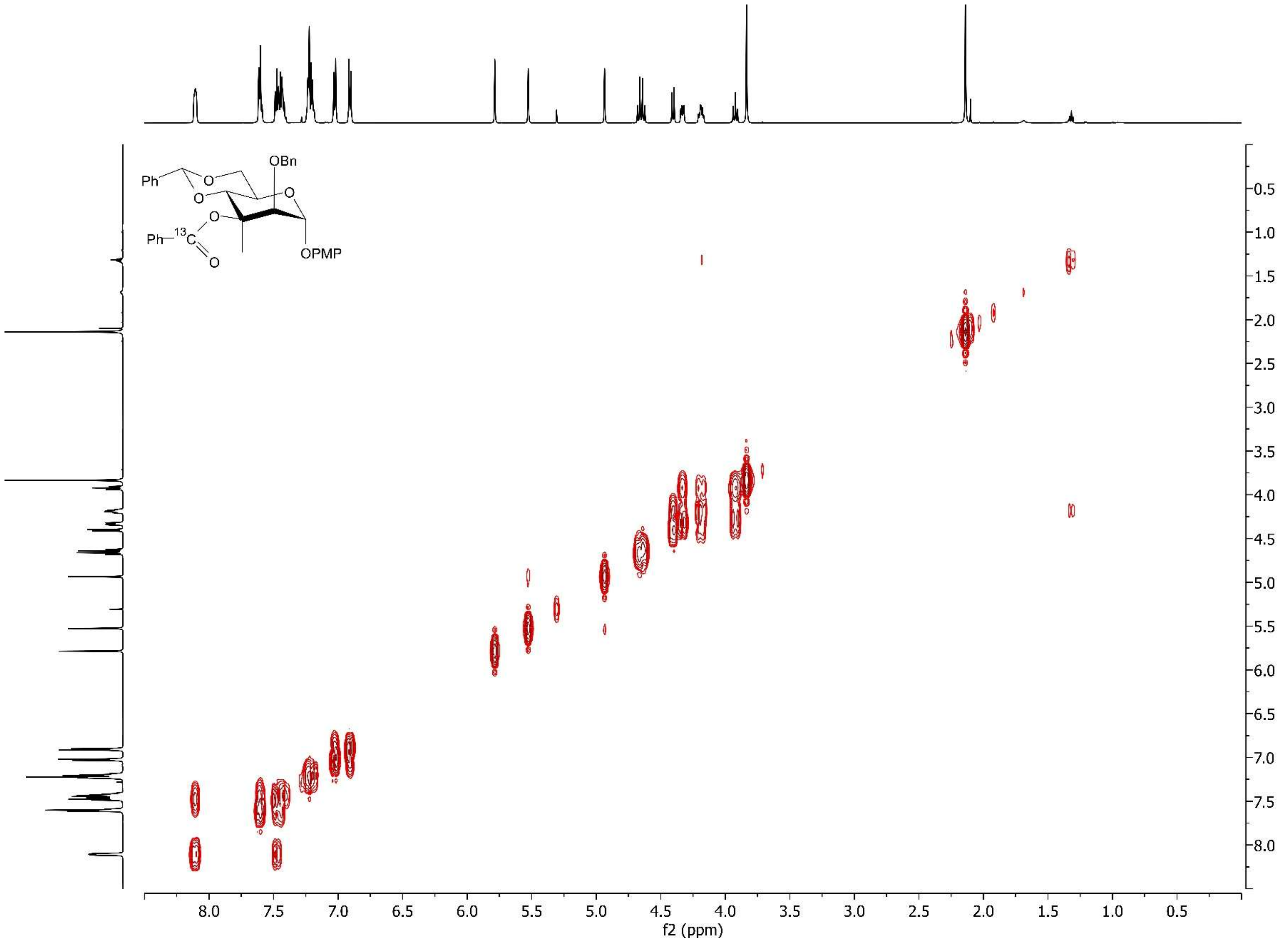

Figure S92. $^{13}C\{H\}$ NMR (151 MHz, $CDCl_3$) spectrum of *p*-methoxyphenyl 3-*O*-(benzoyl-α-^{13}C)-2-*O*-benzyl-4,6-*O*-benzylidene-3-*C*-methyl-α-D-mannopyranoside **30**:

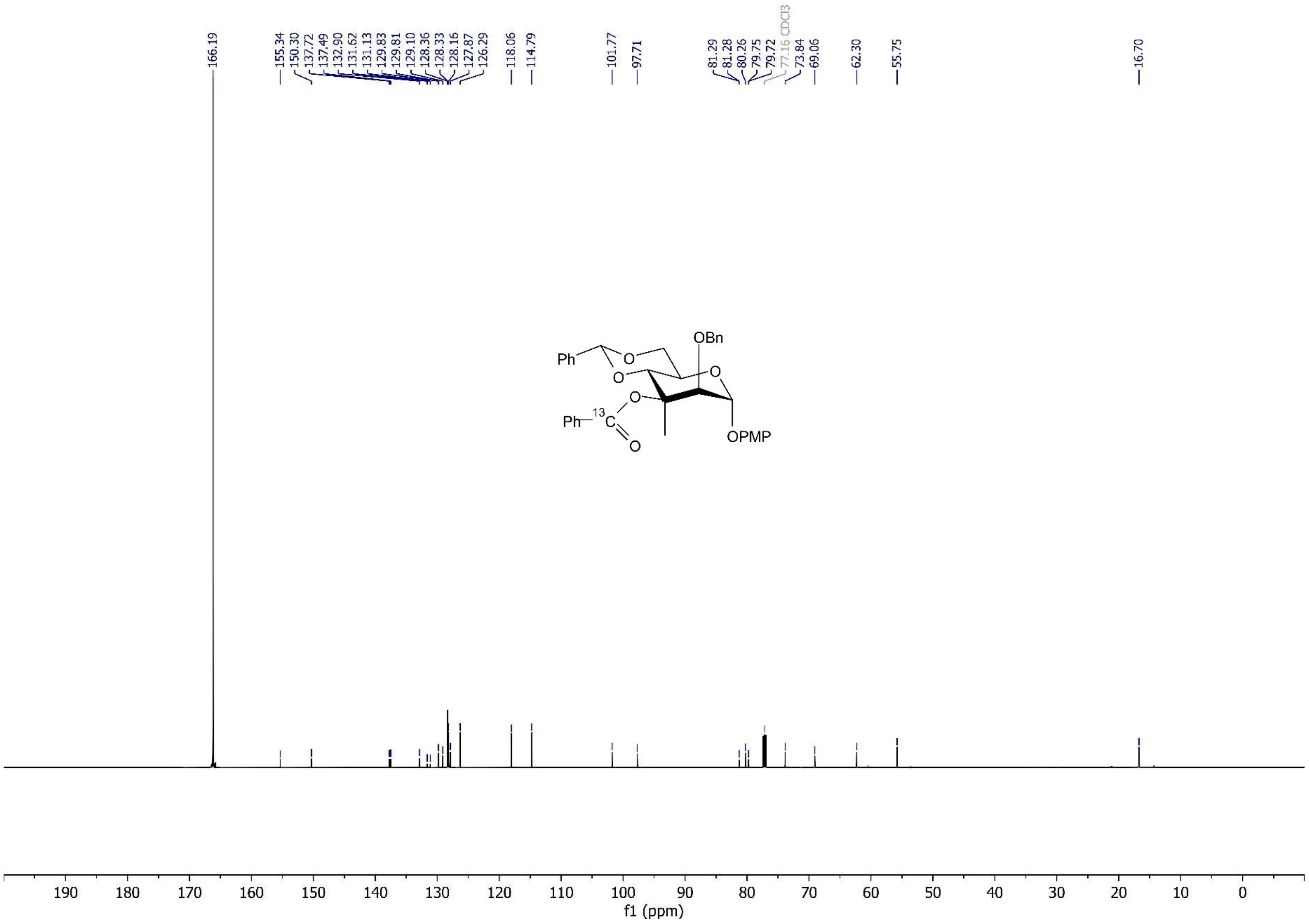

Figure S93. HSQC NMR (600 MHz, $CDCl_3$) spectrum of *p*-methoxyphenyl 3-*O*-(benzoyl-α-^{13}C)-2-*O*-benzyl-4,6-*O*-benzylidene-3-*C*-methyl-α-D-mannopyranoside **30**:

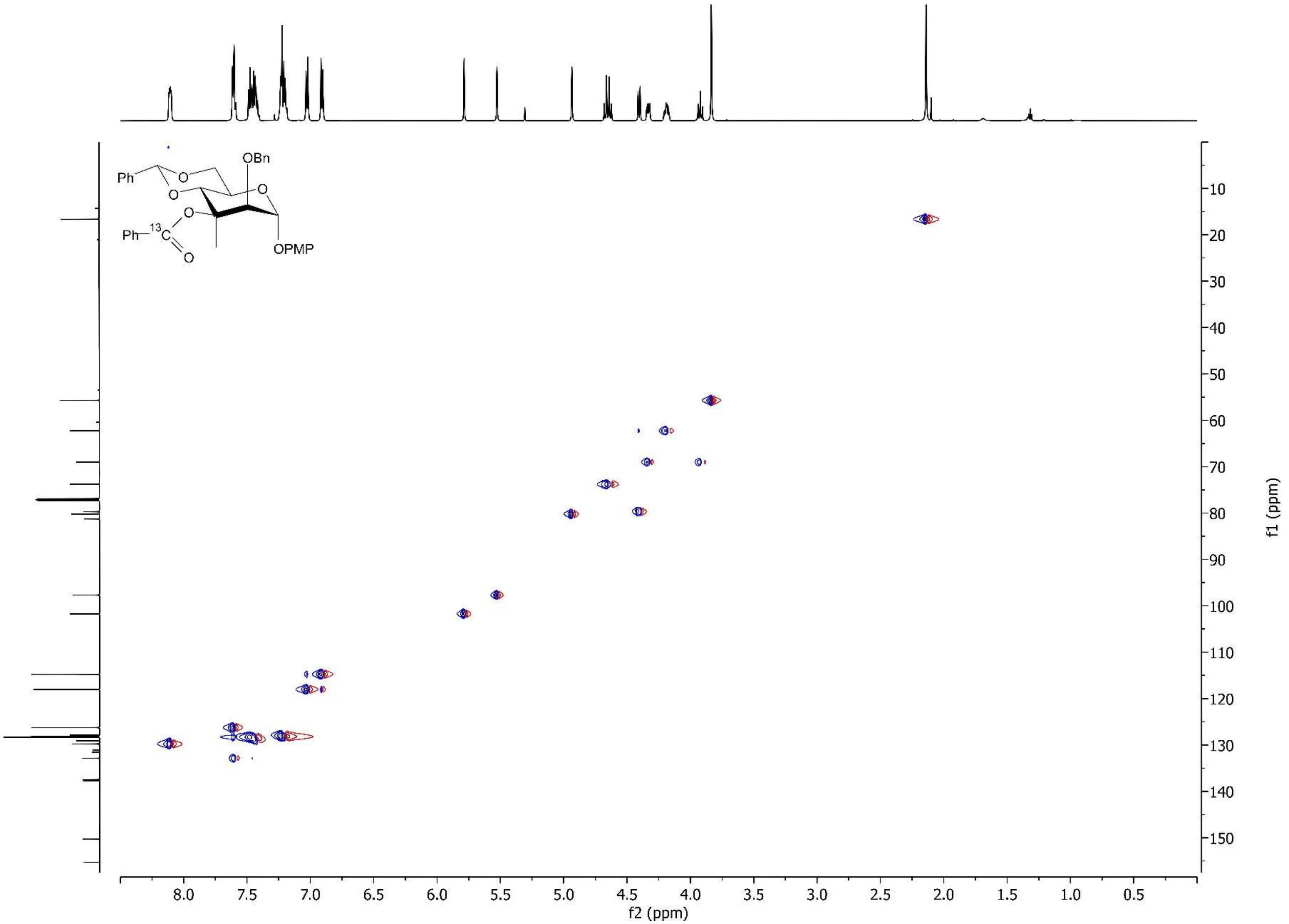

Figure S94. HMBC NMR (600 MHz, $CDCl_3$) spectrum of *p*-methoxyphenyl 3-*O*-(benzoyl-α-^{13}C)-2-*O*-benzyl-4,6-*O*-benzylidene-3-*C*-methyl-α-D-mannopyranoside **30**:

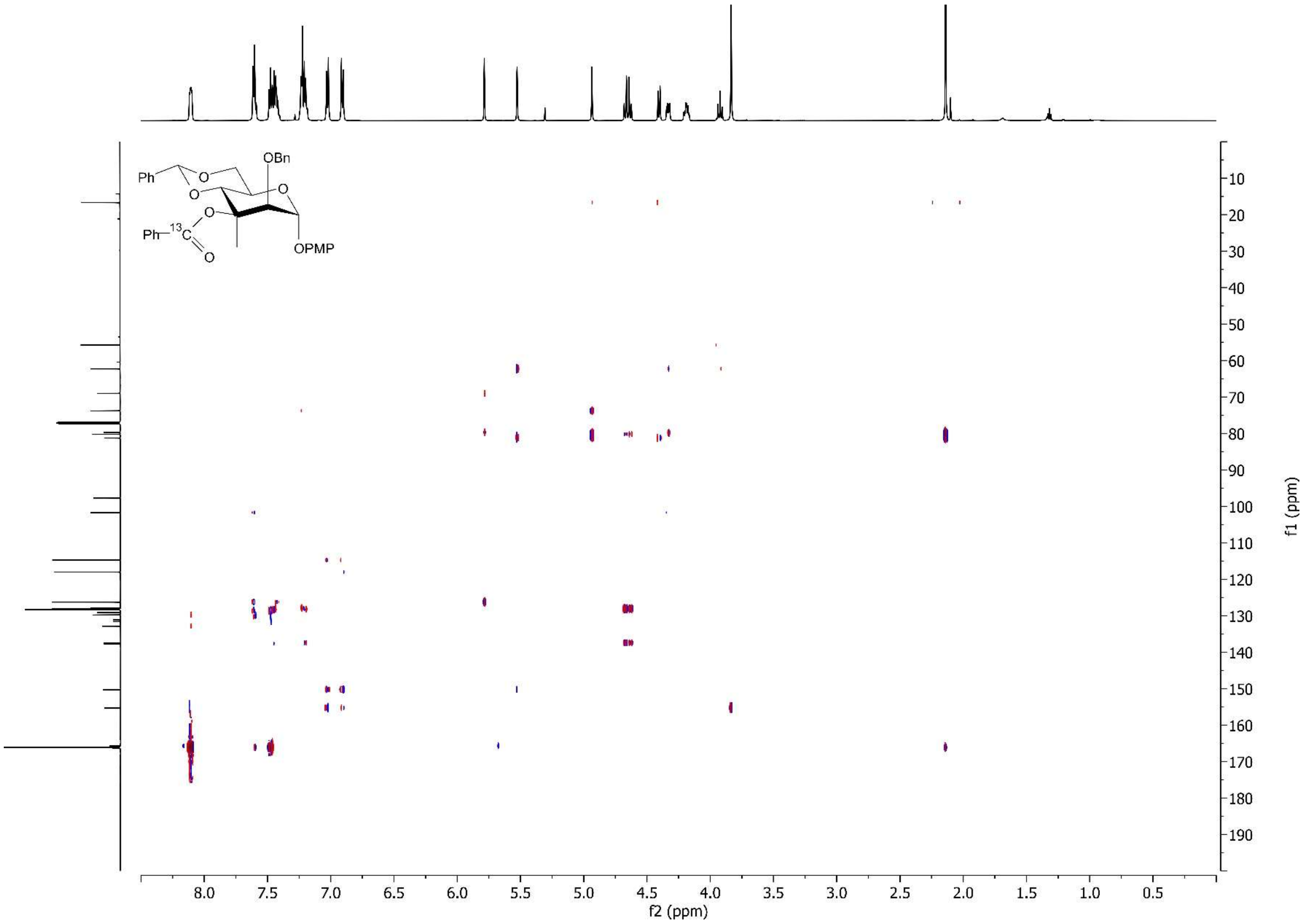

Figure S95. ^{1}H NMR (600 MHz, $CDCl_3$) spectrum of acetyl 2-*O*-benzyl-4,6-*O*-benzylidene-3-*C*-methyl-D-mannopyranose **32**:

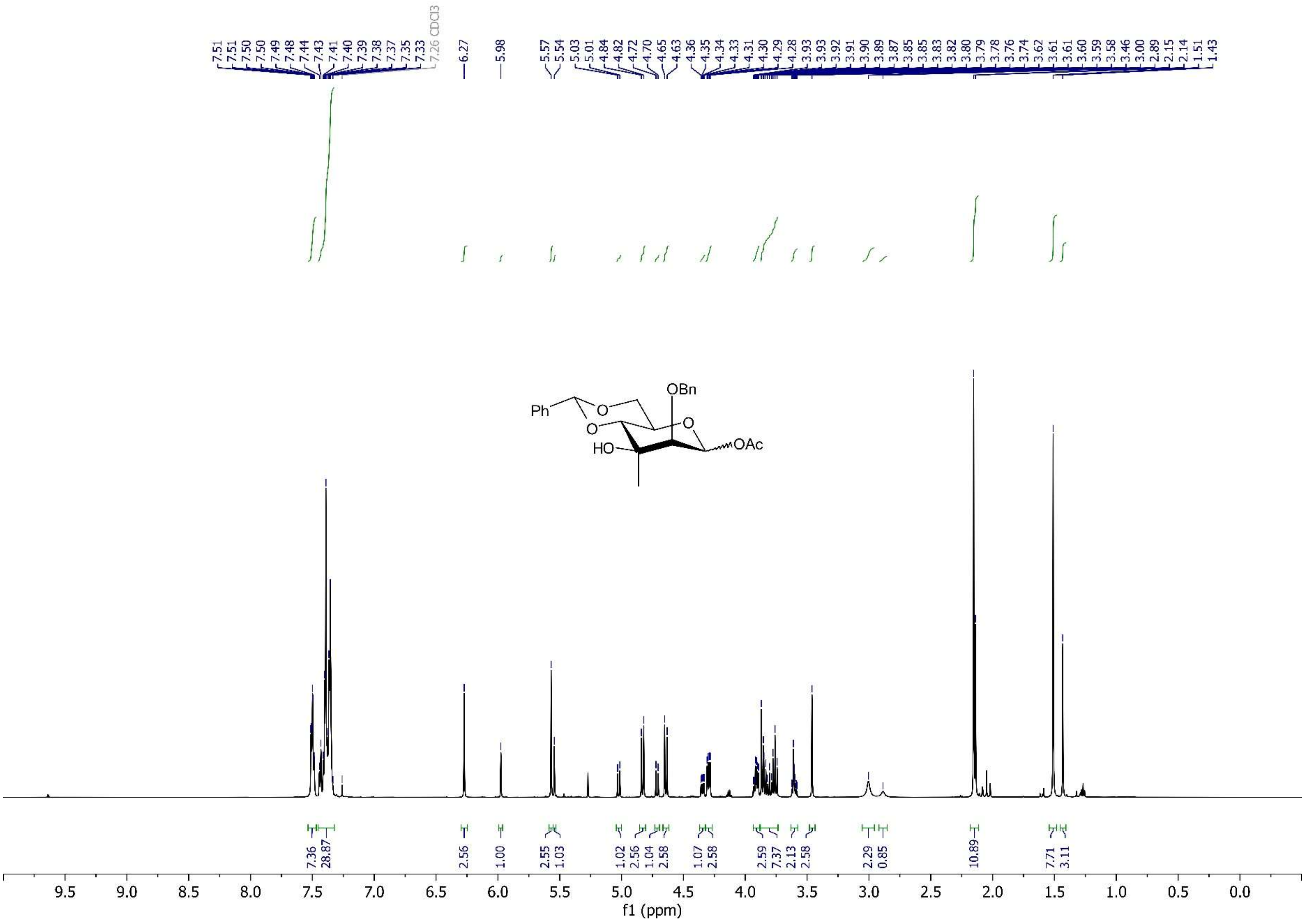

Figure S96. COSY NMR (600 MHz, $CDCl_3$) spectrum of acetyl 2-*O*-benzyl-4,6-*O*-benzylidene-3-*C*-methyl-D-mannopyranose **32**:

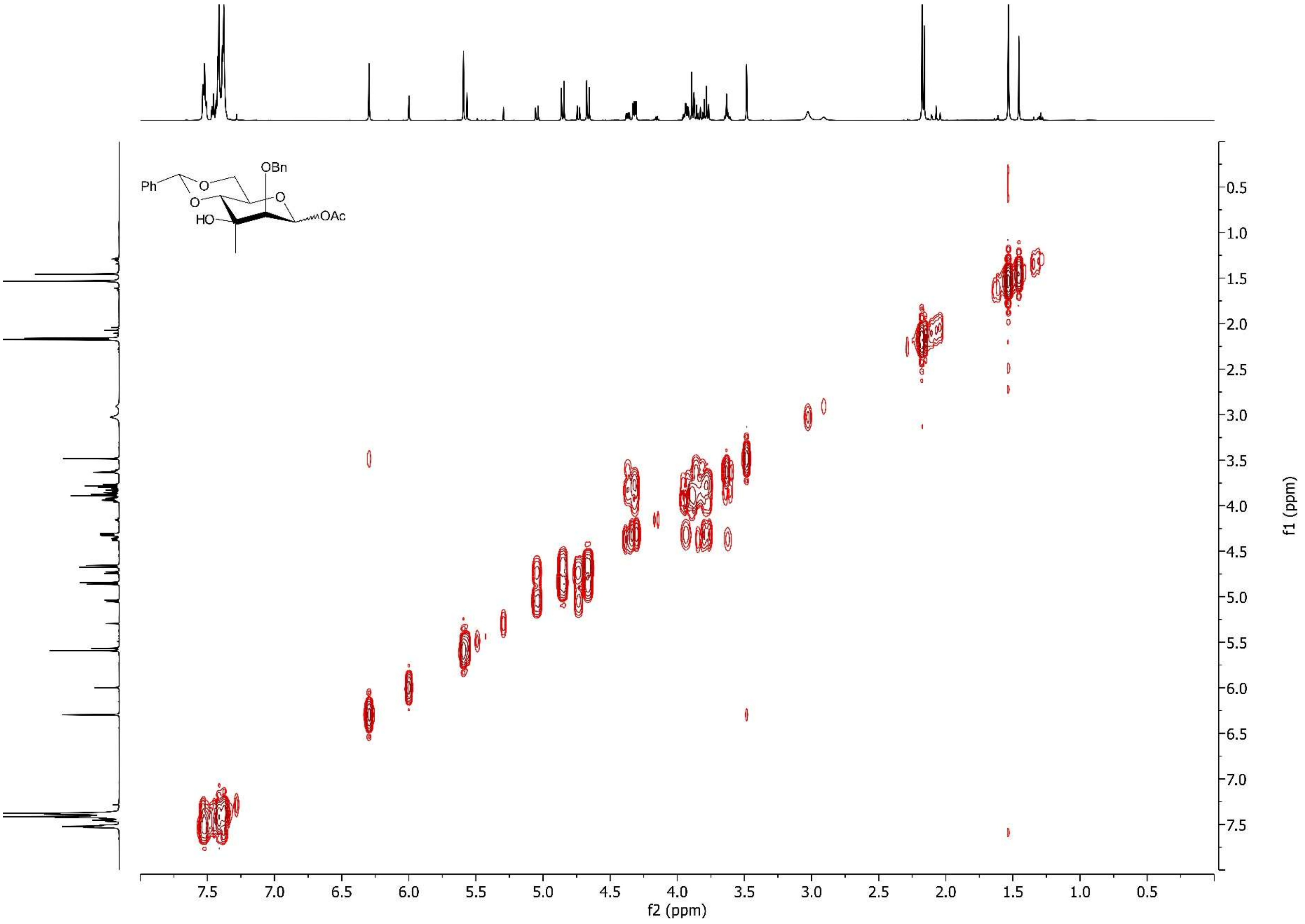

Figure S97. $^{13}C\{H\}$ NMR (151 MHz, $CDCl_3$) spectrum of acetyl 2-*O*-benzyl-4,6-*O*-benzylidene-3-*C*-methyl-D-mannopyranose **32**:

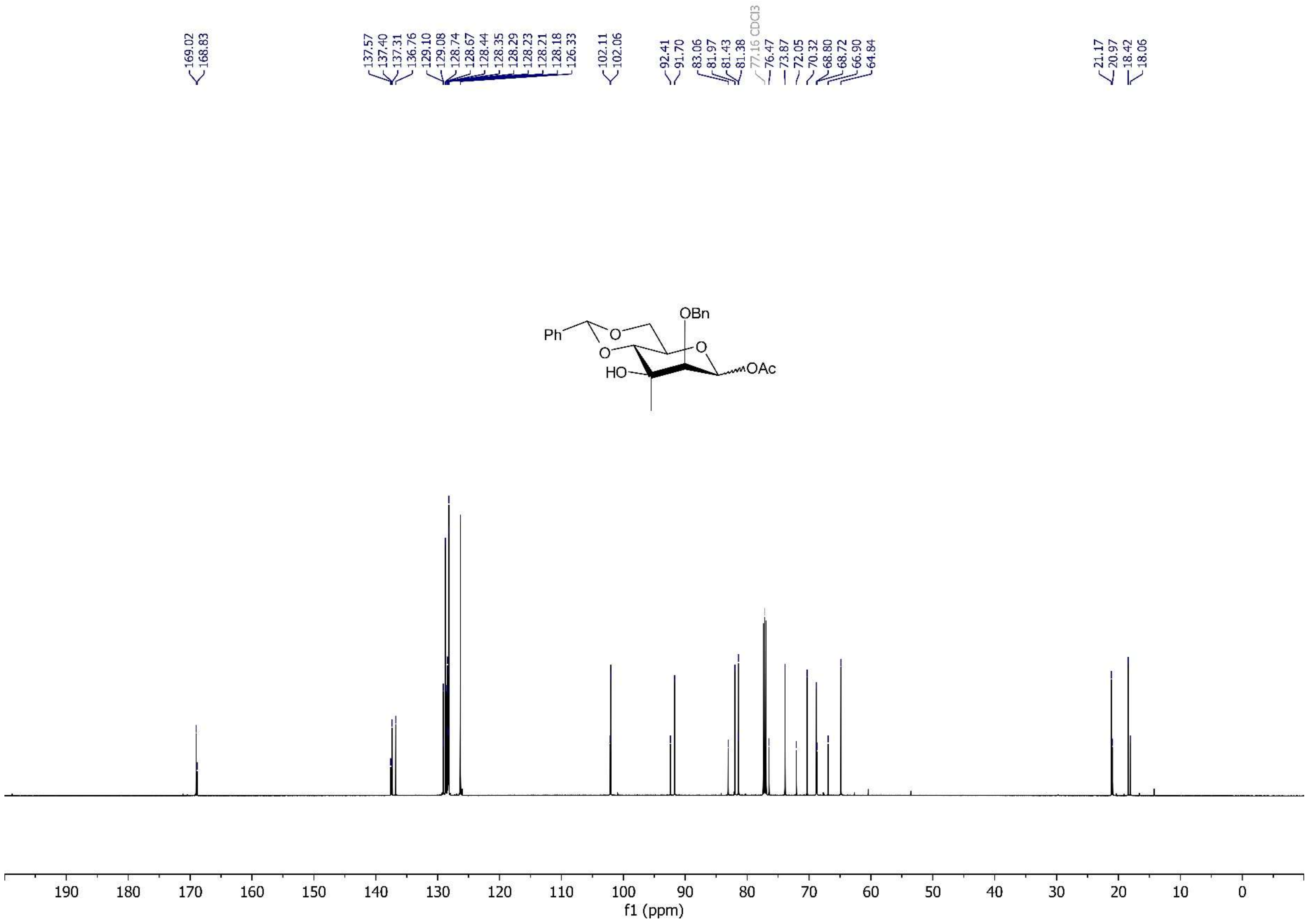

Figure S98. HSQC NMR (600 MHz, $CDCl_3$) spectrum of acetyl 2-*O*-benzyl-4,6-*O*-benzylidene-3-*C*-methyl-D-mannopyranose **32**:

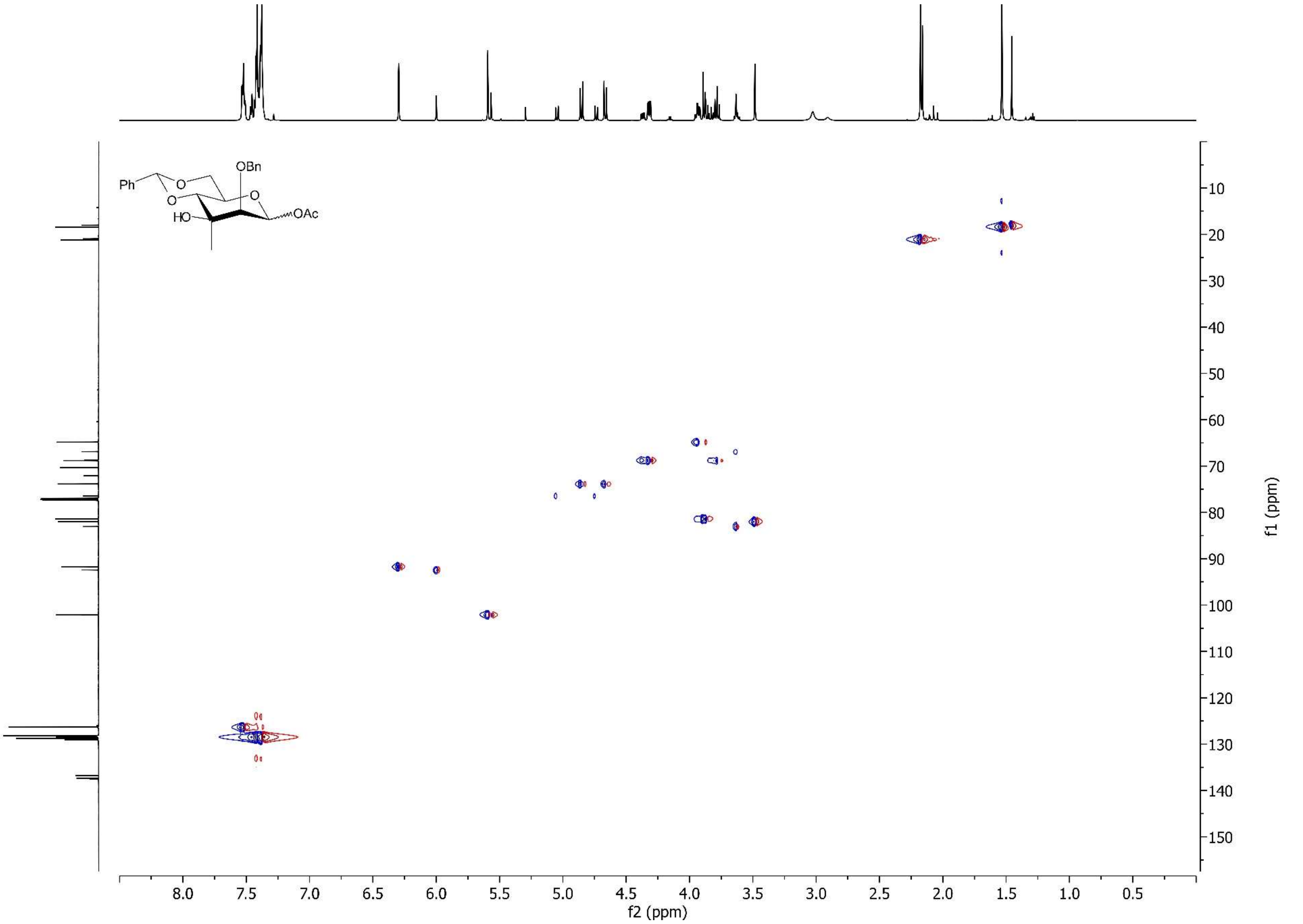

Figure S99. HMBC NMR (600 MHz, $CDCl_3$) spectrum of acetyl 2-*O*-benzyl-4,6-*O*-benzylidene-3-*C*-methyl-D-mannopyranose **32**:

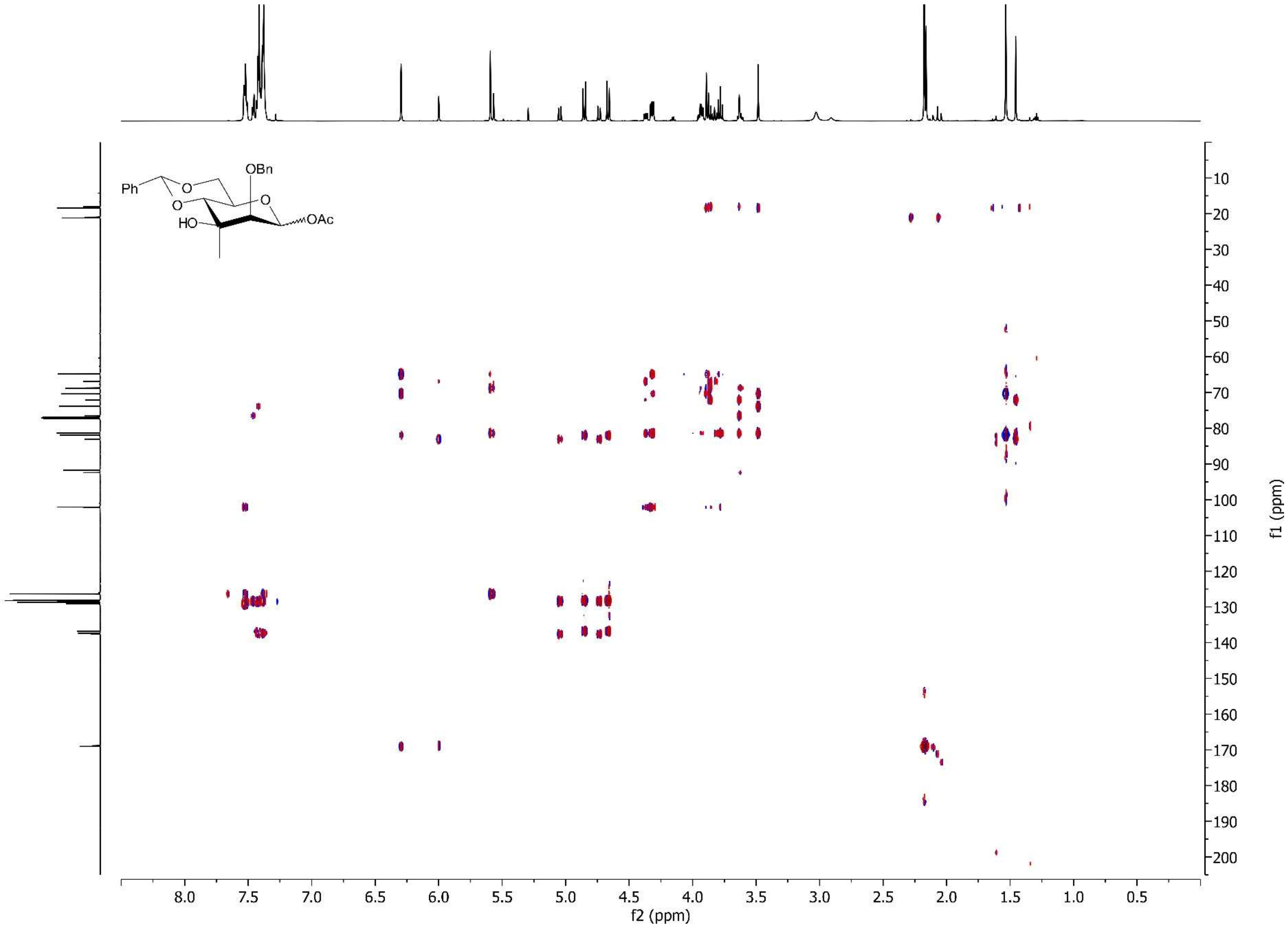

Figure S100. ^{1}H NMR (600 MHz, $CDCl_3$) spectrum of *p*-methylphenyl 2-*O*-benzyl-4,6-*O*-benzylidene-3-*C*-methyl-thio-α-D-mannopyranoside **33**:

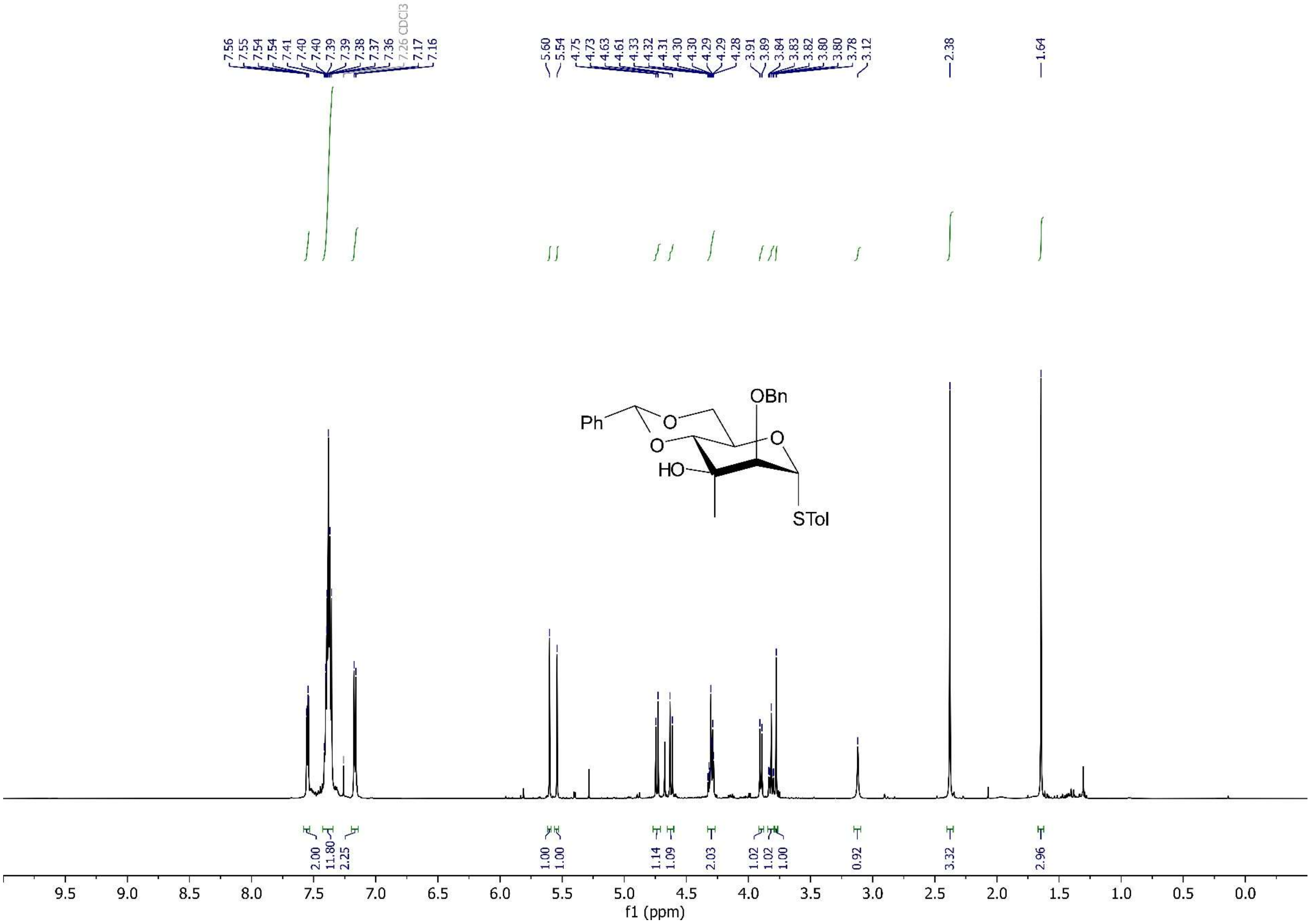

Figure S101. COSY NMR (600 MHz, $CDCl_3$) spectrum of *p*-methylphenyl 2-*O*-benzyl-4,6-*O*-benzylidene-3-*C*-methyl-thio-α-D-mannopyranoside **33**:

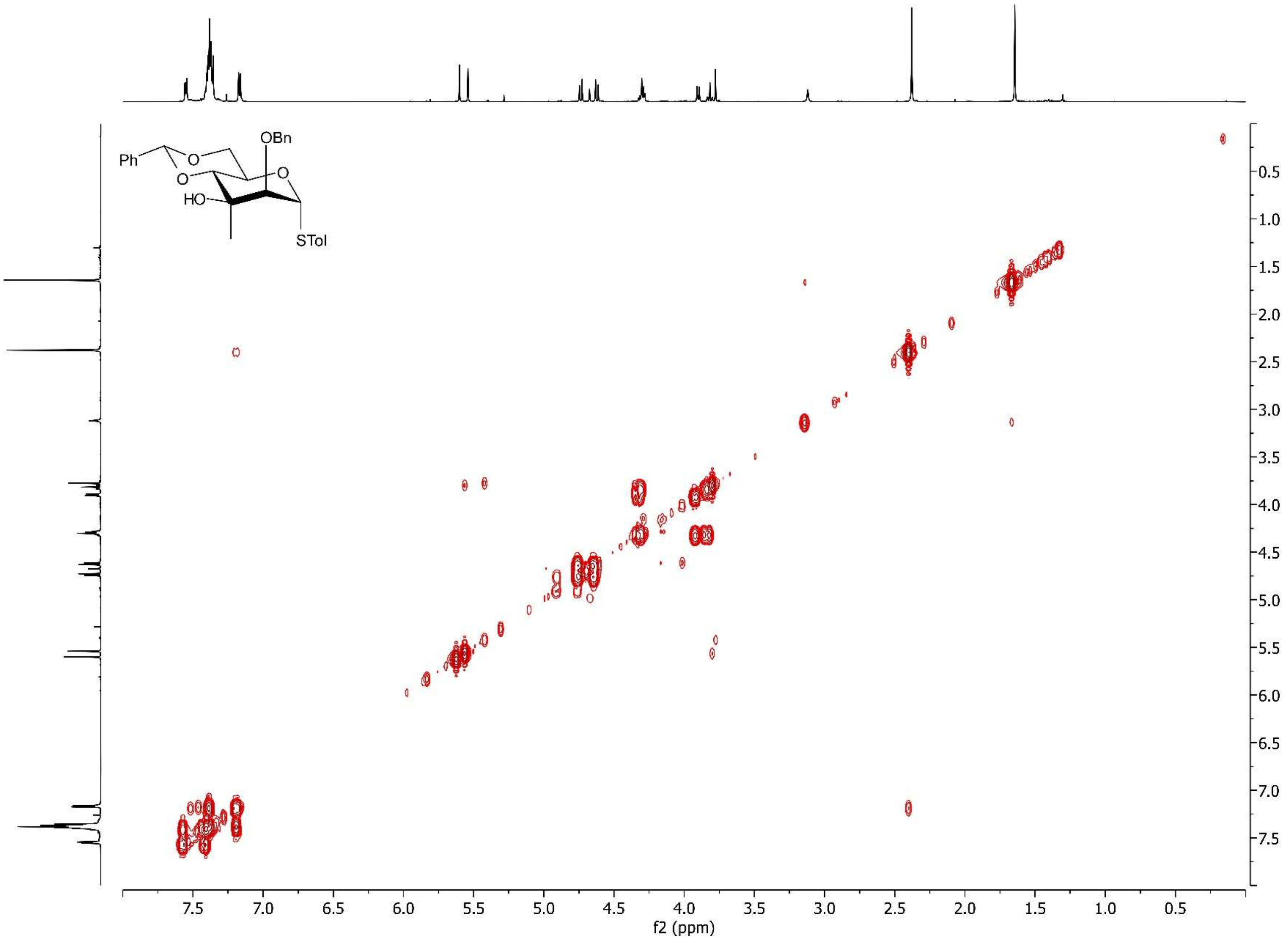

Figure S102. $^{13}C\{H\}$ NMR (151 MHz, $CDCl_3$) spectrum of *p*-methylphenyl 2-*O*-benzyl-4,6-*O*-benzylidene-3-*C*-methyl-thio-α-D-mannopyranoside **33**:

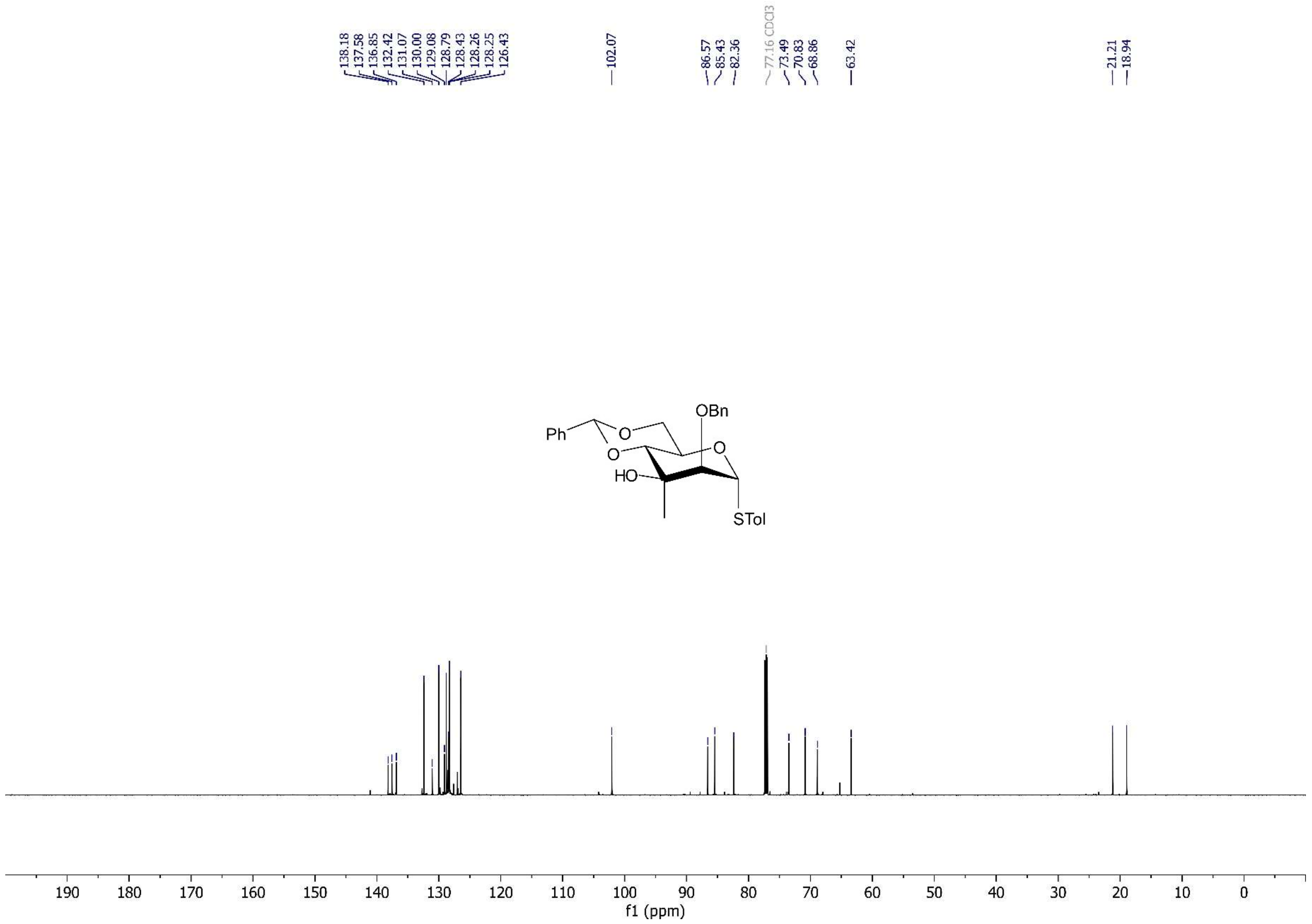

Figure S103. ^{13}C (C-H coupled) NMR (151 MHz, $CDCl_3$) spectrum of *p*-methylphenyl 2-*O*-benzyl-4,6-*O*-benzylidene-3-*C*-methyl-thio-α-D-mannopyranoside **33**:

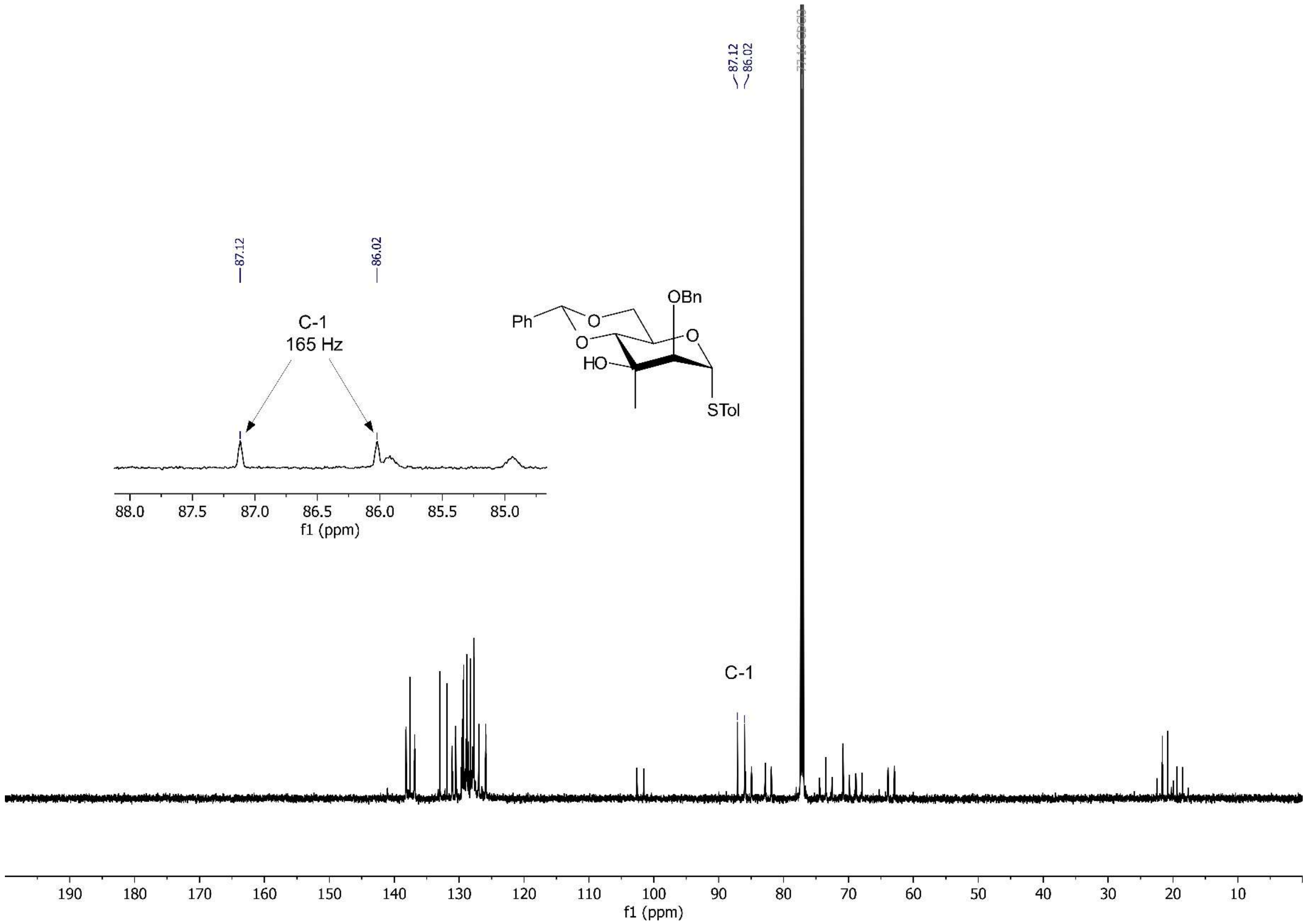

Figure S104. HSQC NMR (600 MHz, $CDCl_3$) spectrum of *p*-methylphenyl 2-*O*-benzyl-4,6-*O*-benzylidene-3-*C*-methyl-thio-α-D-mannopyranoside **33**:

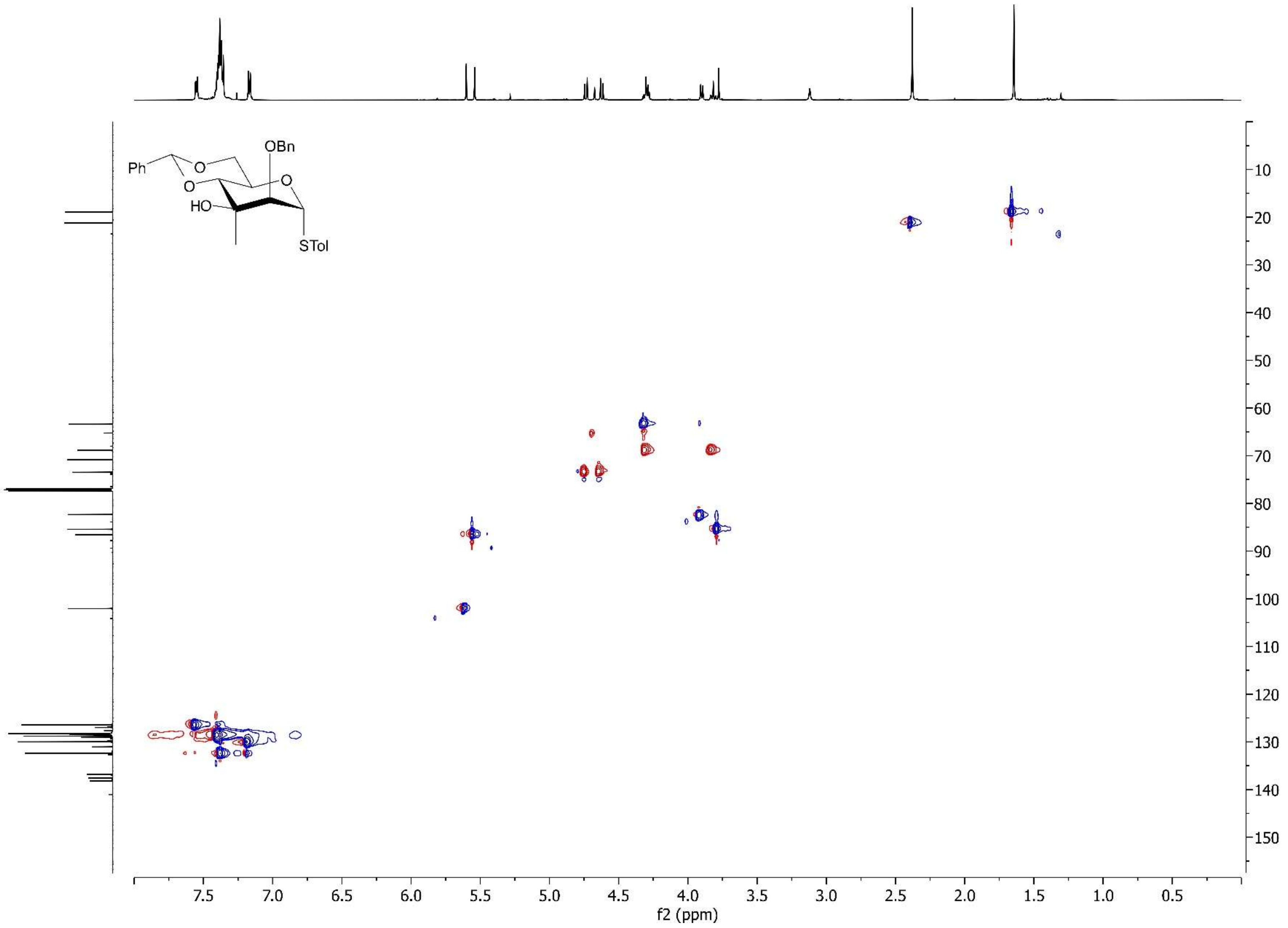

Figure S105. HMBC NMR (600 MHz, $CDCl_3$) spectrum of *p*-methylphenyl 2-*O*-benzyl-4,6-*O*-benzylidene-3-*C*-methyl-thio-α-D-mannopyranoside **33**:

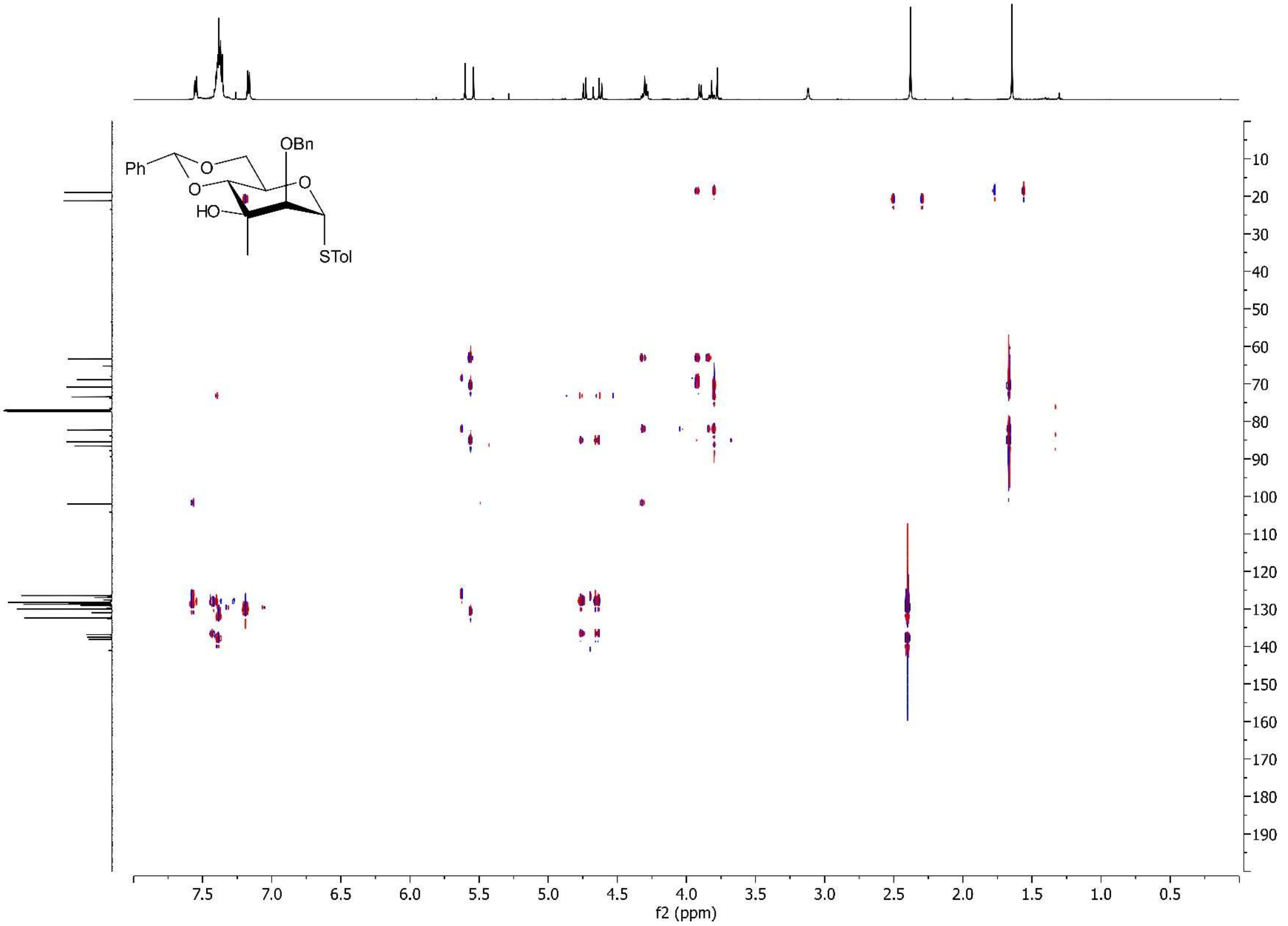

Supplement: Supplementary file 1 [file ao5c12799_si_001.pdf]
